# Global, regional and national burden of drug use disorders, 1990–2021: decomposition analysis, health inequality analysis and predictions to 2035

**DOI:** 10.3389/fpubh.2025.1588607

**Published:** 2025-10-21

**Authors:** Ruiying Jin, Shenyu Zhang, Jun Xiong, Baixi Liu

**Affiliations:** ^1^School of Basic Medical Sciences, Hangzhou Normal University, Hangzhou, China; ^2^School of Public Administration, Hangzhou Normal University, Hangzhou, China; ^3^Engineering Research Center of Mobile Health Management System, Ministry of Education, Hangzhou, China

**Keywords:** opioid use disorder, cocaine use disorder, amphetamine use disorder, cannabis use disorder, other drug use disorder, global burden of disease

## Abstract

**Background:**

Drug use disorders (DUDs), a significant public health issue worldwide, encompass disorders related to sedatives, cannabis, opioids, heroin, hallucinogens, club drugs, and inhalants. This study examines the changes in the global burden of DUDs from 1990 to 2021, aiming to provide a scientific foundation for strategies to mitigate the harms associated with substance abuse.

**Methods:**

The study utilized Global Burden of Disease (GBD) 2021 data to analyze trends in the incidence, prevalence, mortality, disability-adjusted life years (DALYs) associated with DUDs through Socio-demographic Index (SDI), attributable risk factors, and EAPC. Decomposition analysis was employed to assess the impact of age, gender, and SDI on the burden of DUDs, while the inequality distribution of DALYs was examined using the inequality slope index (SII) and the concentration index (CI).

**Results:**

Opioid use disorder accounted for the highest age-standardized disability rates (ASDR) and age-standardized mortality rates (ASMR) among the five drug use disorders, while cannabis use disorder is the leading cause of Age-standardized prevalence rates (ASPR). The Age-standardized incidence rates (ASIR) of opioid use disorder has demonstrated a downward trend, whereas both ASPR and ASMR have shown an increase; notably, the ASIR for opioid use disorder has declined the least among the five drug use disorders. The burden of DUDs in high SDI areas is significantly greater than that in low SDI areas, with income inequality exacerbating the uneven distribution of DALYs. The primary attributable risk factors are drug use and behavioral risks. While the ASIR of opioid use disorder is increasing, while others are experiencing a decline. The burden of DUDs varies among the five drug use disorders due to factors such as population aging, population changes, and age distribution.

**Conclusion:**

The findings reveal absolute and relative inequalities in DALYs associated with drug use disorders, which are concentrated in high-income regions.

## Introduction

Drug use disorders (DUDs), encompassing conditions such as substance abuse and dependence, arise from the consumption of psychoactive substances, including specific medications, on either a single occasion or through repeated use. These disorders are associated with fourteen distinct categories of psychoactive agents. Initial use is often characterized by rewarding psychoactive effects that reinforce continued consumption. However, persistent substance use diminishes control over intake, contributing significantly to the global burden of disease through elevated disability and mortality, alongside profound impairments in physical and mental health, social function, and occupational capacity (1–4). Commonly misused substances involve sedatives, cannabis, amphetamines, cocaine, as well as “club drugs” such as ecstasy, ketamine, and MDMA, in addition to solvents and inhalants (5). Drug use disorders constitute a substantial contributor to the global burden of disease and present a persistent public health challenge globally (6, 7). Data from the GBD 2021 study indicate that these disorders rank among the top 25 causes of years lived with disability (YLD), with their age-standardized YLD rate exhibiting a significant increase between 2010 and 2021 (6). Substance abuse exacts a dual toll, not only inflicting direct health damage but also propagating a spectrum of social problems, thereby forming a complex public health issue.

Furthermore, the World Drug Report 2024 indicates that the number of drug users rose to 292 million in 2022, reflecting a 20% increase over the past decade (6). Despite an estimated 64 million individuals worldwide suffering from drug use disorders, only one in eleven is receiving treatment (6). Notably, women face greater barriers to accessing treatment compared to men, with only one in eighteen women with drug use disorders receiving treatment, in contrast to one in seven men (6).

Previous studies have demonstrated significant regional disparities in the burden of drug use disorders (7, 8). However, comprehensive and up-to-date epidemiological data on these disorders remain limited. The GBD study provides a standardized framework for quantifying health loss, with incidence, prevalence, mortality, and DALYs serving as its core metrics for comprehensive burden assessment. We utilized these established measures to ensure comparability across time and with other diseases. Incidence rates inform on the risk of developing new disorders, crucial for guiding prevention efforts. Prevalence estimates depict the total number of individuals living with a disorder, indicating the immediate population-level healthcare need. Mortality rates directly quantify the fatal outcomes. Finally, DALYs combine years of life lost due to premature death (YLLs) and years lived with disability (YLDs), offering a holistic measure of total health loss that captures both fatal and non-fatal consequences. Together, these metrics provide a multifaceted view of the burden of DUDs, essential for strategic planning and policy formulation.

The GBD 2021 provides the latest dataset on the global burden of drug use disorders across 204 countries and regions from 1990 to 2021 (9, 10). Our study provides a novel contribution through a systematic and individualized analysis of the global burden associated with four common drug use disorders (opioid, cocaine, amphetamine, and cannabis), in addition to other drug use disorder. To our knowledge, this methodological approach, examining each category separately, has not been comprehensively pursued in earlier related research. This study primarily examines the incidence, prevalence, mortality, and DALYs of drug use disorders. It also explores the evolutionary trends from 1990 to 2021 and predicts the global burden of drug use disorders over the next 15 years.

## Method

### Data sources

The study utilized the GBD 2021 database, encompassing epidemiological data from 204 countries and territories across 21 geographic regions for the period 1990–2021 (9). The GBD 2021 study provides modeled estimates rather than raw data. The case definitions for the five drug use disorders are anchored in the International Classification of Diseases (ICD) diagnostic codes to ensure international comparability. Data on the disease burden of drug use disorders were sourced from the Global Health Data Exchange (GHDx) query tool^1^ (11). For example, if we want to get the global ASIR of opioid use disorder in 2021, we go to the website of the GHDx query tool and select ‘Cause of death or injury’ in the ‘GBD Estimate’ drop-down box,‘Incidence’ in ‘Measure’ drop-down box, ‘Rate’ in ‘Metric’ drop-down box,‘Opioid use disorder’ in ‘Cause’ drop-down box, ‘Global’ in the ‘Location’ drop-down box,‘Age-standardised’ in ‘Age’ drop-down box,‘Both’ in ‘Sex’ drop-down box, ‘2021’ in the ‘Year’ drop-down box and finally click ‘Sign in to search’. Through iterative application of the aforementioned data extraction protocol, we systematically compiled incidence, prevalence, mortality, and DALYs related to five distinct categories of drug use disorders. The dataset encompasses both sexes across 18 age groups, covering 204 countries and territories as well as 21 regions between 1990 and 2021. Furthermore, we obtained the SDI for each country from the GBD 2021 database and classified the countries into five SDI quintiles: low, low-middle, middle, high-middle, and high. Each country or territory was assigned an SDI value between 0 and 1, which corresponds to one of the following five categories: High (0.805129–1), High-middle (0.689504–0.805129), Middle (0.607679–0.689504), Low-middle (0.454743–0.607679), and Low (0–0.454743) (12, 13).

### Statistical analysis

This study is a secondary analysis of the latest available data from the GBD 2021. The GBD study generates estimates through its own internal process, which involves systematically aggregating a wide array of primary data sources (e.g., vital registration, surveys, scientific literature) and processing them through standardized statistical models (e.g., DisMod-MR 2.1, CODEm) to produce comparable and comprehensive estimates. The detailed methodology for this process is described in the core GBD publications (14). For this analysis, we utilized these final, model-based estimates as provided by GBD.

### Incidence, prevalence, mortality and disability-adjusted life years (DALYs)

Incidence, prevalence, mortality, and disability-adjusted life years (DALYs) are crucial indicators for assessing the burden of drug use disorders. To account for differences in age structure when analyzing disease burden, we also employed age-standardized incidence rates (ASIR), age-standardized prevalence rates (ASPR), age-standardized mortality rates (ASMR), and age-standardized DALYs rates (ASDR), thus making comparisons between time periods or geographical regions more representative. To ensure clarity, the key metrics obtained from the GHDx are defined as follows: incidence refers to the number of new cases of a disease occurring in a specified period; prevalence represents the total number of existing cases of a disease at a given point in time; mortality indicates the number of deaths caused by the disease and DALYs is a composite metric that quantifies the total burden of disease, representing the sum of Years of Life Lost (YLLs) due to premature mortality and Years Lived with Disability (YLDs) lived in less than full health. ASRs were used for all metrics to allow for comparison across populations with different age structures.

### Estimated annual percentage change (EAPC)

To analyze the trend of drug use disorder burden over a specific time period, we fitted each observed natural logarithm into a linear model based on a four-term age-standardized rate (ASR). We utilized the natural logarithm of the time-based regression model to calculate the 95% confidence interval (CI) for the estimated annual percentage change (EAPC), derived from the slope of this line.


y=α+βx

EAPC=100×(exp(β)−1)

In this context, x represents the year and y denotes log10 (ASR). If the estimated annual percentage changes (EAPCs) and the corresponding 95% confidence interval (CI) are greater than 0, it indicates that the indicator is increasing year over year. Conversely, if the EAPCs and 95% CI are less than 0, it signifies that the indicator is decreasing annually. Furthermore, a larger EAPC value corresponds to a more rapid change in the indicator (15).

### Socio-demographic index (SDI)

SDI is a comprehensive indicator used to assess the level of socio-economic development within a country or region. It evaluates socio-demographic status based on factors such as education level, fertility rate, and per capita income (10, 16).

### Prediction

Projections utilized the Bayesian Age-Period-Cohort (BAPC) model with three core assumptions: (a) continuation of 1990–2021 ASIR trends for opioid use disorders, (b) UN population growth projections (medium variant), and (c) age-structure shifts derived from GBD demographic forecasts. Hyperparameters included 10,000 MCMC iterations and a 20-year cohort effect window (17, 18).

### Decomposition analysis

To estimate the impact of population aging, changes in population size, and age-specific rate variations on DUD deaths, DALYs, and incidence between 1990 and 2021, we employed a credible decomposition method. Detailed information regarding the decomposition analysis has been provided in previous studies (19–21), and the foundational formula is as follows:

A = Ma + Iam + Ipa + Ipam. Here, A represents the primary effect of population aging. Ma denotes the effect of age-specific mortality, Iam signifies the interaction effect of aging and annual specific mortality, and Ipa represents the interaction effect of aging and population growth. Ipam is the joint interaction effect of the three factors.

Similarly, P = Np + Ipm + Ipa + Ipam, where P indicates the main effect of population growth, and Mp reflects the effect of daily population changes. Ipm represents the interaction effect of population dynamics and age rates, while Ipa is the interaction effect between population and aging, and Ipam is the joint interaction effect of these three factors.

Lastly, M = Mm + Ipm + Iam + Ipam, where M represents the primary effect of annual rate changes. Mm denotes the effect of age-specific rates, Ipm indicates the mutual interaction effect of age-specific rates and population, Iam signifies the interaction effect between age-specific rates and aging, and Ipam represents the joint interaction effect of all three.

### Health inequality analyses

Based on the impact of the SDI, geographical regions, and differences in age and gender on incidence, mortality, and DALYs, we conducted an in-depth analysis of health inequality. To achieve an accurate quantification of inequality, this study employed several measures for comprehensive evaluation (22, 23).

(a) Inequality Slope Index (SII): This index quantitatively measures the degree of health outcome inequality across the socio-economic status gradient through regression analysis. The SII value reflects the absolute difference between the highest and lowest socio-economic classes. A SII value of zero indicates no inequality, while a greater absolute value signifies a higher degree of inequality. For favorable indicators, positive values indicate concentration within the dominant group, whereas negative values suggest concentration within the disadvantaged group; conversely, for unfavorable indicators, the situation is reversed.
(b) Concentration Index (CI): This index evaluates the concentration of health outcomes across the entire socio-economic distribution. The closer the CI value is to zero, the more equitable the distribution. The CI can range from −1 to +1; negative values indicate that inequality tends to favor the poor, while positive values suggest that it favors the rich.

### Data visualization

Data visualization was performed using the R software package (version 4.2.3) and JD_GBDR (V2.36; Jingding Medical Technology Co., Ltd.) to visualize the global burden of drug use disorder. Specific R packages, including map, ggplot2, and dplyr, for example, were utilized in this analysis.

## Results

### Global incidence, prevalence, mortality and DALYs

**Figure 1:**
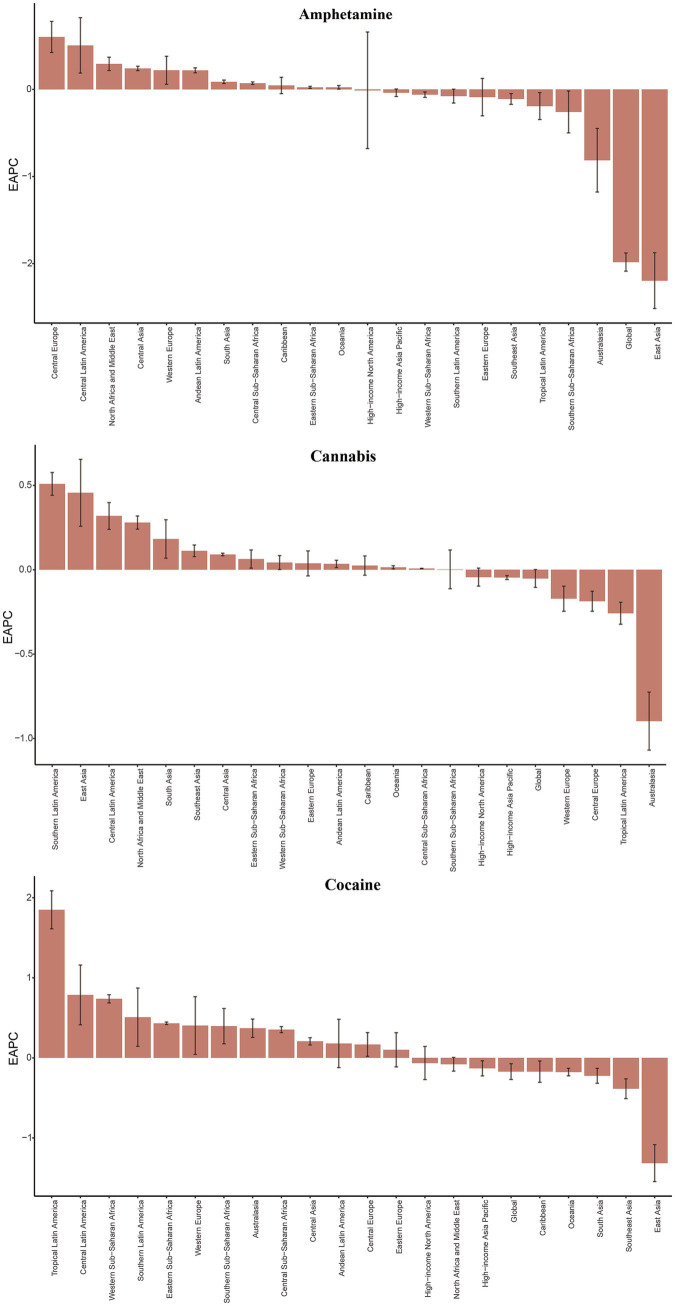
The EAPC of ASIR for amphetamine, cannabis and cocaine use disorders in global and 21 regions. ASIR age-standardized incidence rate, EAPC, estimated annual percentage change. *Y*-axis: EAPC of ASIR (% change/year); *X*-axis: 21 GBD regions; error bars: 95% CI.

**Figure 2:**
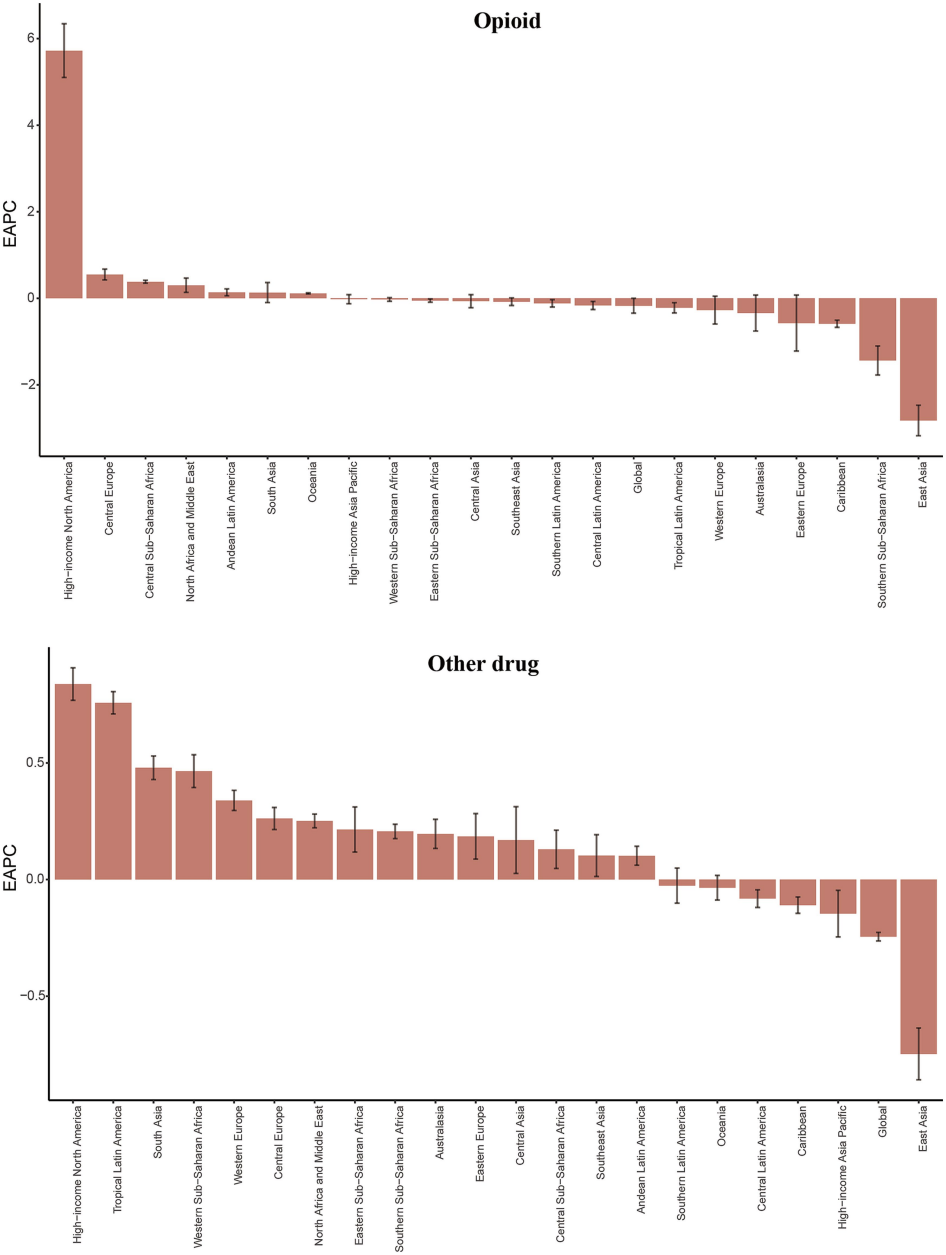
The EAPC of ASIR for opioid and other drug use disorders in global and 21 regions. ASIR age-standardized incidence rate, EAPC, estimated annual percentage change. *Y*-axis: EAPC of ASIR (% change/year); *X*-axis: 21 GBD regions; error bars: 95% CI.

In 2021, the global incidence of opioid use disorder was 24.54 × 10^5^(95%UI 20.74, 29.48), cocaine use disorder was 2.87 × 10^5^ (95%UI 2.06, 3.93) and amphetamine use disorder was 13.72 × 10^5^ (95%UI 9.70, 19.07). Additionally, cannabis use disorder affected 46.77 × 10^5^(95%UI 35.25, 61.17) individuals, while other drug use disorder accounted for 81.49 × 10^5^ (95%UI 62.45, 103.64) cases. The age-standardized incidence rate (ASIR) for other drug use disorder was the highest, at 114.28 per 100,000 people (95% UI 76.12, 169.02). Among the four common drug use disorders, the ASIR was highest for amphetamine use disorder, at 57.07 per 100,000 people (95% UI 30.05, 94.27) (Table 1). From 1990 to 2021, both the ASIR for the four common drug use disorders and other drug use disorder exhibited a downward trend. Notably, the ASIR for amphetamine use disorder showed a significant decline, with an estimated annual percentage change (EAPC) of −1.98 (95% CI −2.09, −1.88), while the ASIR for cannabis use disorder did not demonstrate a statistically significant trend. This divergence potentially linked to differing regulatory environments (Table 1, Figures 1, 2, and Supplementary Table S1).

**Table 1:** Global incidence, prevalence, mortality, and DALYs of 5 drug use disorders from 1990 to 2021.

Year	Opioid	Cocaine	Amphetamine	Cannabis	Other drug
1990					
Incidence					
(×10^5^, 95%UI)	23.37(19.58,28.48)	3.09(2.13,4.39)	22.70(15.92,31.75)	48.46(36.39,63.37)	86.69(65.72,111.42)
Prevalence					
(×10^5^, 95%UI)	154.59(131.06,181.26)	54.64(41.12,72.61)	186.84(136.71,248.87)	298.72(230.75,395.78)	18.65(14.97,22.95)
Mortality					
(×10^5^, 95%UI)	0.86(0.76,0.93)	0.07(0.06,0.09)	0.09(0.08,0.11)	___	0.24(0.19,0.33)
DALYs					
(×10^5^, 95%UI)	103.69(81.83,122.75)	10.91(7.86,14.84)	29.63(19.51,43.52)	8.63(5.10,13.25)	13.58(11.05,18.14)
ASIR					
(1/100,000, 95%UI)	32.77(21.56,47.03)	3.85(2.17,6.45)	31.83(19.86,47.39)	58.14(30.93,94.69)	121.56(79.84,181.78)
ASPR					
(1/100,000,95%UI)	216.80(164.98,281.71)	76.32(52.83,106.52)	262.02(179.94,369.21)	410.79(266.81,619.46)	26.16(17.60,37.80)
ASMR					
(1/100,000,95%UI)	1.20(1.06,1.34)	0.10(0.08,0.13)	0.13(0.10,0.16)	___	0.33(0.26,0.47)
ASDR					
(1/100,000,95%UI)	145.41(112.04,182.65)	15.26(10.72,21.79)	41.55(26.19,63.59)	11.86(6.39,20.29)	19.05(15.12,25.78)
2021					
Incidence					
(×10^5^, 95%UI)	24.54(20.74,29.48)	2.87(2.06,3.93)	13.72(9.70,19.07)	46.77(35.25,61.17)	81.49(62.45,103.64)
Prevalence					
(×10^5^, 95%UI)	198.49(173.42,227.22)	50.63(39.74,63.79)	115.99(84.63,153.55)	286.23(222.58,384.31)	18.17(14.82,22.12)
Mortality					
(×10^5^,95%UI)	1.19(1.12,1.29)	0.15(0.14,0.17)	0.12(0.11,0.13)	___	0.18(0.17,0.20)
DALYs					
(×10^5^,95%UI)	137.15(112.29,161.39)	13.88(11.18,17.52)	20.98(14.56,29.33)	8.27(4.90,12.86)	10.69(9.74,11.80)
ASIR					
(1/100,000,95%UI)	34.42(23.96,47.16)	3.56(2.14,5.82)	19.24(11.51,29.35)	57.07(30.05,94.27)	114.28(76.12,169.02)
ASPR					
(1/100,000,95%UI)	278.36(225.79,343.69)	70.71(51.74,94.86)	162.66(108.71,231.64)	394.36(256.54,599.80)	25.48(17.78,35.88)
ASMR					
(1/100,000,95%UI)	1.67(1.53,1.83)	0.21(0.19,0.25)	0.17(0.15,0.19)	___	0.26(0.24,0.28)
ASDR					
(1/100,000,95%UI)	192.33(153.27,234.10)	19.43(15.25,25.19)	29.42(19.48,43.43)	11.39(6.12,19.41)	14.99(13.35,17.06)
1990-2021					
ASIR (EAPC,95% CI)	-0.17(-0.34, -0.00)	-0.20(-0.29, -0.11)	-1.98(-2.09, -1.88)	-0.02(-0.08,0.05)	-0.24(-0.26, -0.23)
ASPR (EAPC,95% CI)	0.50(0.32,0.69)	-0.22(-0.30, -0.14)	-2.05(-2.21, -1.89)	-0.08(-0.15, -0.02)	-0.11(-0.13, -0.08)
ASMR (EAPC,95% CI)	0.52(0.25,0.78)	2.15(1.92,2.38)	0.02(-0.38,0.43)	___	-1.61(-1.99, -1.23)
ASDR (EAPC,95% CI)	0.54(0.04,1.04)	0.64(-0.04,1.33)	-1.77(-2.24, -1.29)	-0.10(-0.61,0.42)	-1.52(-2.06, -0.96)

DALYs, disability-adjusted life-years; ASIR, age-standardized incidence rate, ASPR, age-standardized prevalence rate; ASMR, age-standardized mortality rate; ASDR, age-standardized DALYs rate; EAPC, estimated annual percentage change; CI, confidence interval; UI, uncertainty intervals; —, negligible mortality.

In 2021, the global prevalence of four common drug use disorders was significantly higher than that of other drug use disorder, and notably, their prevalence exceeded the incidence rates. Among these, cannabis use disorder exhibited the highest prevalence, recorded at 286.23 × 10^5^ (95% UI 222.58, 384.31), with an age-standardized prevalence rate (ASPR) of 394.36 per 100,000 people (95% UI 256.54, 599.80) (Table 1). Between 1990 and 2021, the ASPR for amphetamine use disorder demonstrated a significant downward trend, with an estimated annual percentage change (EAPC) of −2.05 (95% CI -2.21, −1.89), whereas the ASPR for opioids exhibited an upward trend, with an EAPC of 0.50 (95% CI 0.32, 0.69) (Table 1, Supplementary Table S2, and Supplementary Figure S1).

It is noteworthy that the global mortality rate associated with cannabis use disorder has remained at zero. In 2021, approximately 1.19 × 10^5^ (95%UI 1.12, 1.29) cases of opioid use disorder resulted in death worldwide, indicating a significant mortality rate for this drug use disorder. Over recent decades, the age-standardized mortality rate (ASMR) for both opioid use disorder and cocaine use disorder has exhibited an upward trend, with the increase in cocaine use disorder being particularly pronounced [EAPC 2.15 (95% CI 1.92, 2.38)]. Conversely, other drug use disorder has shown a downward trend [EAPC −1.61 (95% UI −1.99, −1.23)], while no statistically significant trend was observed for amphetamine use disorder (Table 1, Supplementary Table S2, and Supplementary Figure S2).

In 2021, the global highest DALYs value for opioid use disorder was 137.15 × 10^5^ (95% UI 112.29, 161.39), with an age-standardized DALYs rate (ASDR) of 192.33 (95% UI 153.27, 234.10) per 100,000 people. Furthermore, it exhibited the highest positive Estimated Annual Percentage Change (EAPC) for ASDR at 0.54 (95% CI 0.04, 1.04). Additionally, between 1990 and 2021, amphetamine use disorder showed a significant downward trend with an ASDR of −1.77 (95% CI −2.24, −1.29), as did other drug use disorder with an ASDR of −1.52 (95% CI −2.06, −0.96) (Table 1, Supplementary Table S1, and Supplementary Figure S3).

### Regional incidence, prevalence, mortality, and DALYs

In 2021, High-income North America and Australia exhibited the highest incidence, prevalence, mortality, and DALYs associated with opioid use disorder, as determined through geographic region analysis. Specifically, High-income North America recorded the highest figures across all four indicators of opioid use disorder: an incidence rate of 123.28 × 10^5^ (95% UI 103.38, 148.55), a prevalence rate of 1862.41 × 10^5^ (95% UI 1644.13, 2112.86), a mortality rate of 15.72 × 10^5^ (95% UI 13.93, 17.79), and DALYs of 1504.74 × 10^5^ (95% UI 1244.10, 1740.26). Furthermore, High-income North America also reported the highest values for cocaine use disorder indicators, with an incidence rate of 18.72 × 10^5^ (95% UI 13.89, 26.38), a prevalence rate of 458.04 × 10^5^ (95% UI 364.74, 560.10), a mortality rate of 1.99 × 10^5^ (95% UI 1.78, 2.43), and DALYs of 150.77 × 10^5^ (95% UI 125.40, 186.20). Additionally, the region had the highest values for cannabis use disorder indicators, including an incidence rate of 122.64 × 10^5^ (95% UI 92.82, 156.76), a prevalence rate of 865.30 × 10^5^ (95% UI 677.13, 1120.15), and DALYs of 24.72 × 10^5^ (95% UI 15.03, 37.94). For amphetamine use disorder, High-income North America recorded the highest incidence rate of 36.24 × 10^5^ (95% UI 26.07, 48.86), a mortality rate of 1.29 × 10^5^ (95% UI 1.14, 1.57), and DALYs of 97.61 × 10^5^ (95% UI 79.87, 120.43), while the highest prevalence value of 478.93 × 10^5^ (95% UI 351.11, 629.69) was reported in Australia. Australia also exhibited the highest values for four indicators of other drug use disorder, with an incidence rate of 209.69 × 10^5^ (95% UI 166.25, 259.98), a prevalence rate of 111.73 × 10^5^ (95% UI 94.65, 128.15), a mortality rate of 2.71 × 10^5^ (95% UI 2.42, 3.04), and DALYs of 132.94 × 10^5^ (95% UI 118.38, 147.31) (Table 2; Supplementary Tables S3–S5).

**Table 2:** Regional incidence and ASIR of the 5 drug use disorders in 2021.

Location_name	Amphetamine		Cannabis		Cocaine		Opioid		Other drug	
	Incidence	ASIR	Incidence	ASIR	Incidence	ASIR	Incidence	ASIR	Incidence	ASIR
Global	13.55 (9.60, 18.79)	13.72 (9.70, 19.07)	45.94 (34.70, 60.03)	46.77 (35.25, 61.17)	2.78 (2.00, 3.79)	2.87 (2.06, 3.93)	24.62 (20.82, 29.51)	24.54 (20.74, 29.48)	85.58 (65.84, 108.86)	81.49 (62.45, 103.64)
East Asia	24.48 (17.38, 33.97)	32.31 (22.40, 45.25)	29.55 (21.83, 38.75)	36.16 (26.09, 49.11)	0.57 (0.37, 0.84)	0.72 (0.46, 1.05)	16.64 (13.78, 19.93)	16.71 (13.88, 20.27)	102.15 (77.41, 130.73)	88.03 (66.19, 112.56)
Southeast Asia	23.37 (15.69, 33.82)	22.65 (15.16, 32.80)	47.34 (34.83, 64.32)	46.30 (33.74, 63.67)	0.24 (0.15, 0.37)	0.24 (0.14, 0.36)	9.81 (8.13, 11.80)	9.36 (7.79, 11.23)	69.56 (52.03, 89.89)	62.94 (46.97, 81.27)
Oceania	18.76 (12.21, 27.49)	17.24 (11.45, 25.00)	79.80 (51.62, 114.15)	72.23 (47.14, 102.46)	0.30 (0.17, 0.46)	0.28 (0.16, 0.42)	12.47 (10.10, 15.47)	12.49 (10.20, 15.23)	66.81 (50.52, 85.13)	71.01 (54.14, 90.31)
Central Asia	16.34 (11.39, 22.89)	16.61 (11.63, 23.13)	32.24 (21.88, 47.39)	33.79 (22.62, 50.51)	2.01 (1.36, 2.88)	2.10 (1.42, 2.99)	36.61 (30.88, 43.65)	36.68 (30.96, 43.70)	85.93 (63.74, 110.17)	80.53 (60.00, 102.81)
Central Europe	18.01 (12.64, 24.91)	23.83 (16.19, 33.54)	38.92 (30.44, 48.98)	55.12 (42.33, 71.28)	2.23 (1.48, 3.20)	3.08 (2.00, 4.57)	14.31 (12.27, 16.86)	16.10 (13.62, 18.96)	95.66 (72.11, 124.17)	86.12 (63.91, 111.03)
Eastern Europe	19.63 (14.21, 26.22)	26.25 (18.74, 35.39)	40.87 (28.91, 55.93)	55.89 (37.85, 79.13)	3.08 (2.18, 4.25)	4.35 (3.13, 6.05)	62.48 (53.28, 74.26)	73.32 (61.90, 87.26)	130.17 (98.25, 164.92)	115.92 (87.05, 146.22)
High-income Asia Pacific	11.44 (7.94, 15.95)	15.15 (10.11, 21.23)	52.76 (38.38, 71.38)	78.59 (55.32, 109.81)	4.19 (2.89, 5.84)	6.50 (4.43, 9.50)	12.88 (10.48, 15.49)	14.92 (12.05, 18.42)	93.94 (70.02, 122.84)	89.22 (64.91, 117.30)
Australasia	47.12 (32.25, 66.21)	55.68 (38.08, 78.43)	91.63 (72.41, 115.10)	114.65 (90.13, 144.62)	9.24 (6.32, 13.51)	12.43 (8.34, 18.59)	41.91 (35.82, 48.48)	44.87 (38.68, 51.99)	209.69 (166.25, 259.98)	197.85 (155.75, 246.44)
Western Europe	19.80 (13.82, 27.26)	25.31 (17.31, 35.24)	67.82 (54.44, 83.47)	96.20 (76.54, 119.49)	6.65 (4.46, 9.82)	9.53 (6.20, 14.54)	20.76 (18.04, 23.93)	24.07 (20.69, 28.09)	149.88 (118.03, 187.47)	146.89 (114.29, 184.79)
Southern Latin America	10.82 (7.34, 15.23)	10.73 (7.23, 15.12)	55.12 (45.01, 66.99)	59.35 (48.47, 72.21)	13.36 (8.86, 20.38)	14.82 (9.74, 22.82)	18.30 (14.69, 22.53)	17.75 (14.11, 22.10)	102.04 (74.89, 132.50)	93.48 (68.39, 121.15)
High-income North America	36.24 (26.07, 48.86)	42.00 (29.89, 56.72)	122.64 (92.82, 156.76)	151.30 (114.29, 196.40)	18.72 (13.89, 26.38)	23.87 (17.69, 33.93)	123.28 (103.38, 148.55)	144.24 (120.13, 174.95)	162.06 (127.74, 205.75)	158.67 (122.50, 205.36)
Caribbean	7.08 (4.91,9.99)	6.95 (4.79,9.85)	72.87 (48.52,104.05)	74.97 (49.43,108.44)	7.48 (4.65,11.38)	7.82 (4.84,11.94)	16.07 (12.84,19.88)	15.61 (12.48,19.26)	78.65 (60.02,100.47)	74.74 (56.94,95.65)
Andean Latin America	10.04 (6.82, 14.25)	9.29 (6.33, 13.13)	41.74 (30.40, 55.82)	39.76 (29.10, 52.90)	5.40 (3.40, 8.27)	5.27 (3.32, 8.08)	18.72 (14.85, 23.39)	17.39 (13.80, 21.67)	78.72 (58.24, 101.77)	75.53 (55.90, 97.52)
Central Latin America	7.17 (4.87, 9.97)	6.75 (4.59, 9.38)	43.92 (33.87, 56.48)	42.34 (32.65, 54.46)	7.81 (5.00, 11.94)	7.56 (4.83, 11.58)	16.20 (12.95, 20.05)	15.25 (12.19, 18.85)	76.85 (57.62, 99.18)	72.15 (54.14, 93.22)
Tropical Latin America	19.39 (12.99, 27.48)	19.40 (12.91, 27.72)	61.50 (46.57, 79.49)	64.81 (48.52, 84.57)	10.68 (7.27, 15.92)	12.04 (8.21, 18.04)	16.51 (13.14, 20.59)	15.82 (12.43, 19.74)	77.49 (58.45, 100.25)	68.33 (51.33, 88.43)
North Africa and Middle East	4.97 (3.45, 6.98)	4.75 (3.30, 6.67)	23.89 (16.96, 32.99)	22.73 (16.10, 31.39)	1.69 (1.12, 2.44)	1.62 (1.07, 2.34)	39.37 (32.69, 47.57)	37.82 (31.50, 45.62)	82.60 (61.84, 108.11)	76.60 (57.60, 99.30)
South Asia	1.88 (1.30, 2.65)	1.73 (1.21, 2.43)	49.92 (36.39, 67.32)	44.47 (32.43, 59.54)	0.41 (0.27, 0.59)	0.37 (0.24, 0.54)	20.49 (16.77, 25.24)	18.90 (15.73, 23.12)	67.96 (51.31, 87.63)	65.94 (49.97, 84.78)
Central Sub-Saharan Africa	5.53 (3.64, 7.83)	5.31 (3.64, 7.43)	34.66 (23.22, 51.58)	30.72 (21.35, 43.62)	0.86 (0.56, 1.22)	0.80 (0.54, 1.14)	11.72 (9.31, 14.78)	12.49 (10.24, 15.37)	48.27 (36.14, 62.55)	60.73 (46.06, 77.73)
Eastern Sub-Saharan Africa	5.38 (3.50, 7.75)	4.98 (3.36, 7.00)	43.75 (29.38, 62.99)	36.35 (25.69, 50.62)	0.50 (0.34, 0.73)	0.48 (0.34, 0.68)	10.03 (8.06, 12.58)	10.77 (8.94, 12.99)	38.42 (28.46, 49.85)	48.51 (36.55, 62.36)
Southern Sub-Saharan Africa	12.48 (8.65,17.44)	11.74 (8.21,16.33)	49.91 (34.94,68.62)	46.14 (32.43,63.24)	5.84 (4.12,8.20)	5.51 (3.89,7.67)	23.95 (20.00,29.15)	23.31 (19.68,28.10)	77.61 (59.47,97.32)	74.81 (57.43,93.96)
Western Sub-Saharan Africa	4.77 (3.12, 6.82)	4.66 (3.16, 6.60)	23.73 (16.86, 32.78)	21.72 (15.81, 29.29)	0.58 (0.41, 0.80)	0.70 (0.53, 0.90)	10.18 (8.06, 12.89)	10.88 (8.95, 13.31)	42.16 (31.24, 54.16)	56.73 (43.19, 71.98)
High-middle SDI	18.76 (13.23, 26.15)	22.78 (15.75, 32.04)	36.23 (28.06, 46.29)	44.92 (34.37, 57.91)	2.46 (1.70, 3.42)	3.29 (2.23, 4.73)	25.75 (21.84, 30.59)	27.16 (23.02, 32.63)	105.83 (80.48, 136.52)	91.49 (69.02, 117.21)
High SDI	24.63 (17.79, 33.12)	30.22 (21.39, 41.49)	78.57 (60.59, 99.72)	106.41 (80.40, 136.64)	9.39 (6.97, 12.98)	13.52 (10.02, 18.91)	55.73 (47.40, 65.98)	68.52 (57.67, 82.33)	142.01 (111.30, 177.00)	132.22 (102.22, 166.22)
Low-middle SDI	5.81 (4.00, 8.24)	5.28 (3.66, 7.46)	42.90 (32.20, 57.57)	38.86 (29.16, 51.76)	1.22 (0.82, 1.68)	1.10 (0.75, 1.49)	19.31 (15.92, 23.73)	18.22 (15.29, 22.17)	66.41 (50.26, 85.32)	67.09 (51.04, 86.16)
Low SDI	4.40 (2.92, 6.30)	4.17 (2.85, 5.84)	39.92 (28.01, 55.00)	35.15 (25.65, 47.64)	0.67 (0.46, 0.95)	0.63 (0.45, 0.89)	13.89 (11.16, 17.39)	14.35 (11.93, 17.42)	44.96 (33.42, 57.61)	56.51 (42.82, 71.90)
Middle SDI	16.06 (11.33, 22.53)	16.65 (11.67, 23.46)	41.65 (31.22, 54.60)	42.89 (31.99, 56.76)	2.17 (1.49, 3.07)	2.33 (1.59, 3.33)	19.19 (16.08, 22.98)	18.82 (15.81, 22.76)	83.15 (62.90, 107.04)	74.50 (56.28, 95.40)

The unit of incidence is (×10^5^, 95%UI), the unit of ASIR is (1/100,000, 95%UI), ASIR, age-standardized incidence rate; UI, uncertainty interval.

In 2021, the highest values of the four standardized indicators for the five drug use disorders were observed in High-income North America and Australia. Specifically, among these indicators, opioid use disorder in High-income North America ranked first, with an ASIR of 144.24 per 100,000 people (95% UI 120.13, 174.95) and ASPR of 1890.26 per 100,000 people (95% UI 1659.84, 2156.24). The ASMR was 14.50 per 100,000 people (95% UI 12.92, 16.30), and ASDR was 1502.44 per 100,000 people (95% UI 1235.96, 1740.10). For cocaine use disorder, High-income North America also reported the highest values, with an ASIR of 23.87 per 100,000 people (95% UI 17.69, 33.93) and an ASPR of 479.97 per 100,000 people (95% UI 379.72, 592.54). The ASMR was 1.75 per 100,000 people (95% UI 1.57, 2.11), and the ASDR was 147.83 per 100,000 people (95% UI 121.82, 183.99). Additionally, the three indicators for cannabis use disorder in High-income North America were also the highest, with an ASIR of 151.30 per 100,000 people (95% UI 114.29, 196.40), an ASPR of 973.88 per 100,000 people (95% UI 752.87, 1275.29), and an ASDR of 27.88 per 100,000 people (95% UI 16.97, 42.78). In terms of two indicators, high-income North America exhibited the highest rates of amphetamine use disorder, with an ASMR of 1.15 per 100,000 people (95% UI 1.03, 1.39) and an ASDR of 98.53 per 100,000 people (95% UI 79.65, 122.87). Conversely, Australia recorded the highest ASIR at 55.68 per 100,000 people (95% UI 38.08, 78.43) and the highest ASPR at 513.42 per 100,000 people (95% UI 371.55, 682.47). Among the four key indicators for other drug use disorder, Australia also demonstrated the highest figures: ASIR at 197.85 per 100,000 people (95% UI 155.75, 246.44), ASPR at 102.72 per 100,000 people (95% UI 87.20, 118.37), ASMR at 2.36 per 100,000 people (95% UI 2.11, 2.64), and ASDR at 123.67 per 100,000 people (95% UI 109.73, 137.69). It is important to note that regional comparisons of ‘highest/lowest’ values should be interpreted with caution where uncertainty intervals overlap substantially, as this may indicate non-significant differences (Table 2; Supplementary Tables S3–S5).

From 1990 to 2021, the overall burden of the five drug use disorders exhibited an upward trend in more than half of the GBD regions. High-income North America experienced the most significant increase in the burden of opioid use disorder, while the burden of cocaine use increased the most in Tropical Latin America. Additionally, Central Europe and High-income North America saw the largest increases in amphetamine drug use disorder burden, whereas Southern Latin America experienced the most substantial rise in cannabis use disorder burden. Furthermore, High-income North America and Tropical Latin America also recorded the most significant increases in other drug use disorder. Notably, with the exception of the largest decline in cannabis use disorder burden observed in Australia, the other four drug use disorders experienced the most significant reductions in East Asia (Figures 1, 2; Supplementary Figures S1–S3).

### National incidence, prevalence, mortality, and DALYs

**Figure 3:**
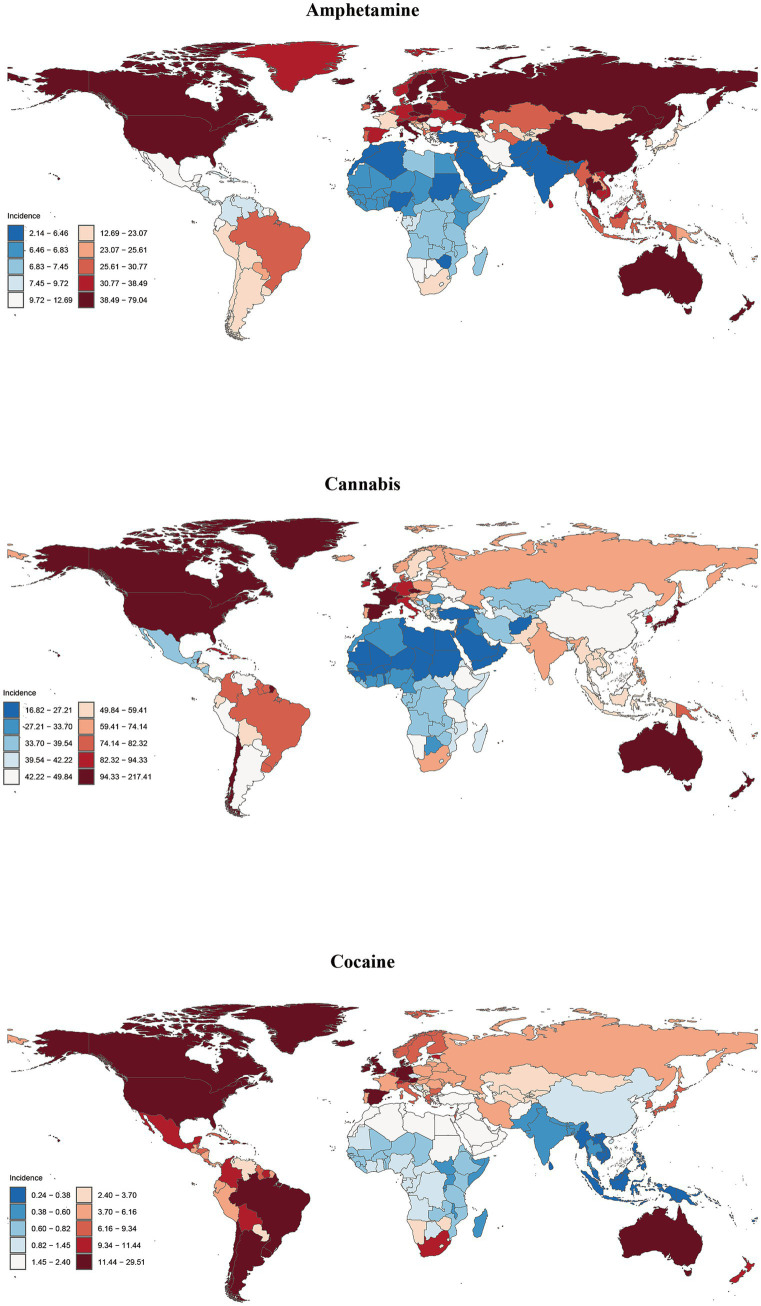
Global incidence of amphetamine, cannabis and cocaine use disorders in 204 countries or territories in 2021.

In 2021, Australia exhibited the highest ASIR and ASPR for amphetamine use disorder, reaching 78.25 and 723.99 per 100,000 people, respectively. In contrast, the United States of America had the highest ASMR at 1.71 and the highest ASDR at 145.72 (Supplementary Table S6 and Supplementary Figure S4). Data from 1990 to 2021 indicate that Sweden experienced the most rapid growth in ASIR and ASPR, with estimated annual percentage changes (EAPCs) of 2.01 (95% CI: 1.87, 2.15) and 2.35 (95% CI 2.16, 2.53), respectively. Furthermore, Mauritius demonstrated the fastest growth in ASMR at 17.25 (95% CI 12.59, 22.10), while the United States exhibited the highest growth in ASDR at 3.95 (95% CI 3.23, 4.68) (Figure 3, Supplementary Tables S7, S8, and Supplementary Figure S4).

New Zealand exhibits the highest ASIR, ASPR and ASDR for cannabis use disorder, with values of 215.26, 1516.12 and 43.54 per 100,000 people, respectively (Supplementary Table S6 and Supplementary Figure S4). Between 1990 and 2021, Chile experienced the most rapid increases in ASPR and ASDR, with estimated annual percentage changes (EAPCs) of 1.52 (95% CI 1.35, 1.68) and 1.51 (95% CI 1.35, 1.68), respectively. In contrast, Kenya reported the fastest growth in ASIR at 1.26 (95% CI 1.02, 1.49) (Supplementary Tables S7, S8).

The United States of America recorded the highest ASIR, ASPR, ASMR and ASDR for cocaine use disorder, with values of 29.22, 698.05, 2.59 and 216.76 per 100,000 people, respectively (Figure 3, Supplementary Figure S4, and Supplementary Table S6). From 1990 to 2021, the United Kingdom exhibited the most significant increases in ASIR and ASPR for cocaine use disorder, with EAPCs of 3.27 (95% CI 2.33, 4.23) and 2.99 (95% CI 2.19, 3.80), respectively. Furthermore, Mauritius demonstrated the fastest growth in ASMR and ASDR, with rates of 14.56 (95% CI 10.45, 18.83) and 9.77 (95% CI 7.44, 12.14), respectively (Supplementary Tables S7, S8).

**Figure 4:**
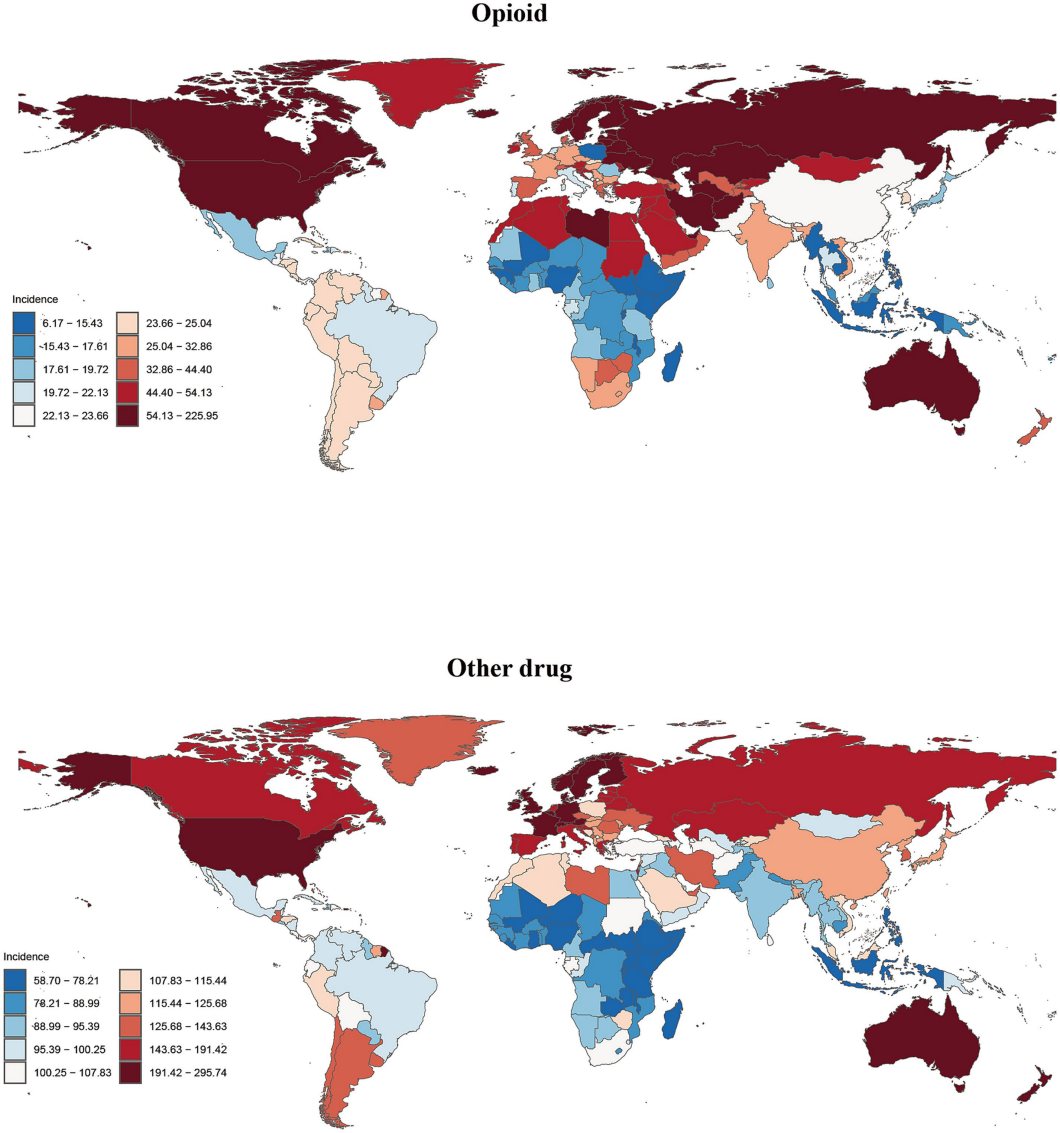
Global incidence of opioid and other drug use disorders in 204 countries or territories in 2021.

The highest ASIR values for opioid use disorder, at 223.71 per 100,000 people, are observed in the Republic of Estonia. In contrast, the highest ASPR, ASMR and ASDR values are found in the United States of America, with figures of 2825.30, 21.56, and 2236.30 per 100,000 people, respectively (Figure 4, Supplementary Figure S4, and Supplementary Table S6). Data indicate that from 1990 to 2021, the ASIR, ASPR, and ASDR for opioid use disorder in the United States have experienced the most rapid growth, with estimated annual percentage changes (EAPCs) of 6.05 (95% CI 5.44, 6.67), 6.73 (95% CI 6.21, 7.25), and 7.33 (95% CI 7.08, 7.58), respectively. The ASMR has shown the fastest increase in Mauritius, with an EAPC of 10.64 (95% CI 8.11, 13.22) (Supplementary Tables S7, S8).

The highest values of ASIR, ASPR, ASMR and ASDR for other drug use disorder were all recorded in Australia, at 292.81, 159.61, 3.69, and 192.93 per 100,000 people, respectively (Figure 4, Supplementary Figure S4, and Supplementary Table S6). From 1990 to 2021, Mauritius exhibited the most rapid increases in ASIR and ASPR, with Estimated Annual Percentage Changes (EAPCs) of 1.41(95%CI 1.27, 1.54) and 2.95 (95%CI 2.60, 3.30), respectively. The ASMR increased most significantly in Sao Tome and Principe, with an EAPC of 8.67 (95%CI 8.15, 9.20), while the ASDR saw the highest growth in Turkmenistan, with an EAPC of 6.12 (95% CI 5.37, 6.87) (Supplementary Tables S7, S8).

When analyzing the ASMR for drug use disorders in the GBD database, we observed zero ASMR values in most countries and territories. This phenomenon may stem from several factors. First, limitations in cause-of-death surveillance systems in certain regions can lead to the misclassification of drug-related fatalities; for instance, overdose deaths might be coded as “accidental poisoning” or “suicide” rather than being attributed to the underlying disorder. Second, coding practices under the International Classification of Diseases (ICD) system seldom list drug use disorders as the underlying cause of death, resulting in statistical underestimation. Additionally, geographical variations in the direct lethality of drug use disorders may exist, with some regions achieving reduced mortality risks through effective public health interventions. Given the potential for underestimation bias in ASMR data, we excluded the three countries and territories with the lowest ASMR values from our summary tables to avoid misleading interpretations. Future studies should integrate non-fatal burden metrics (e.g., ASIR, ASPR) and conduct subgroup analyses for a more comprehensive assessment.

### Burden of 5 drug use disorders by SDI

**Figure 5:**
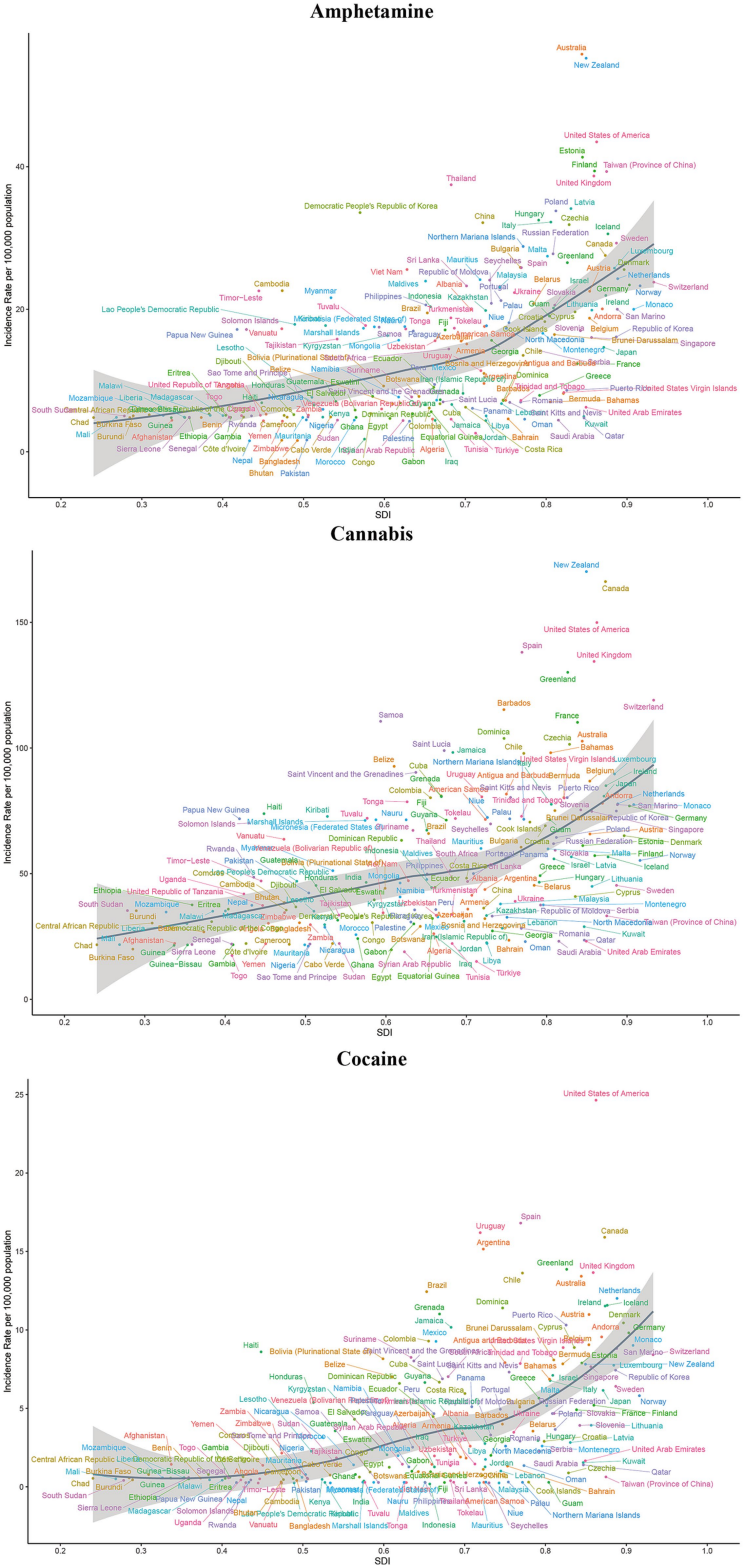
ASIR of amphetamine, cannabis and cocaine use disorders for 204 countries and territories by SDI. The colors of the country labels are used for visual distinction only and do not represent any categorical grouping. ASIR, age-standardized incidence rate; SDI, socio-demographic index.

**Figure 6:**
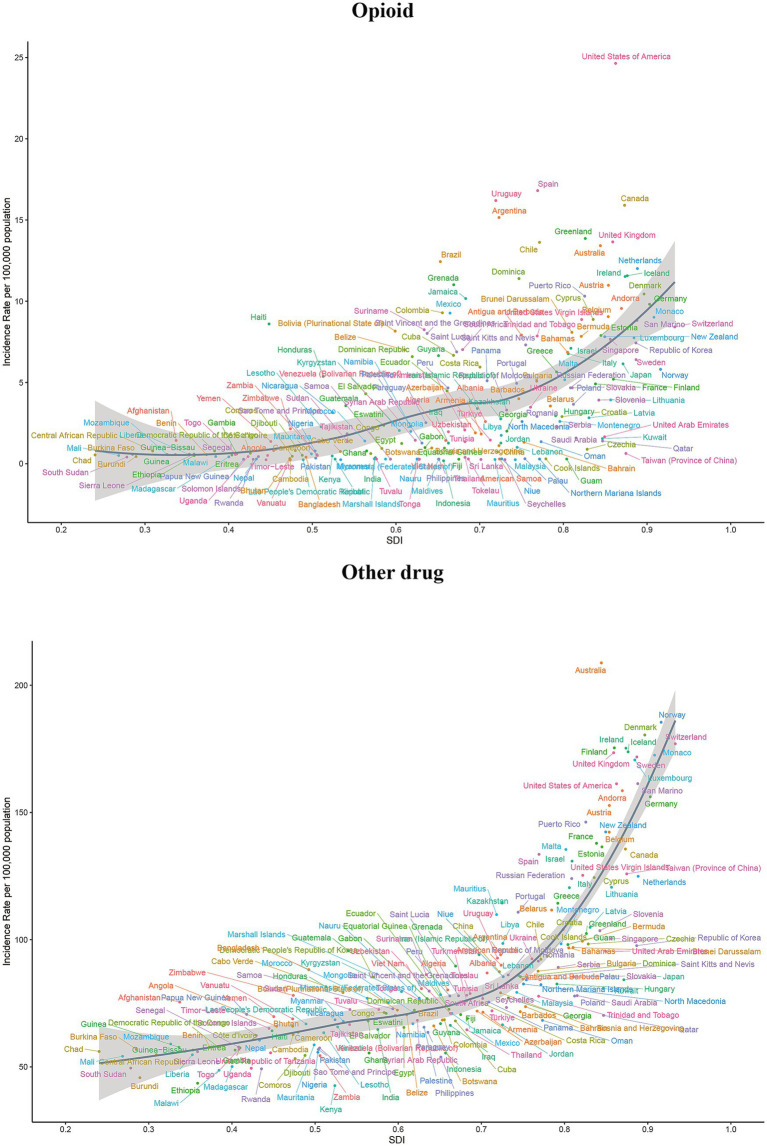
ASIR of opioid and other drug use disorders for 204 countries and territories by SDI. The colors of the country labels are used for visual distinction only and do not represent any categorical grouping. ASIR, age-standardized incidence rate; SDI, socio-demographic index.

In 2021, both the medium and high SDI quintiles for the five drug use disorders exhibited elevated incidence, prevalence, mortality, and DALY. At the national level, a positive correlation was observed in 2021 between the burden of these five drug use disorders and the ASIR and ASPR across 204 countries and regions. Furthermore, the SDI corresponding to the ASMR and ASDR also demonstrated a positive relationship with the other four drug use disorders, excluding cannabis (Figures 5, 6; Supplementary Figures S5–S7).

### Burden of 5 drug use disorders by age and sex

**Figure 7:**
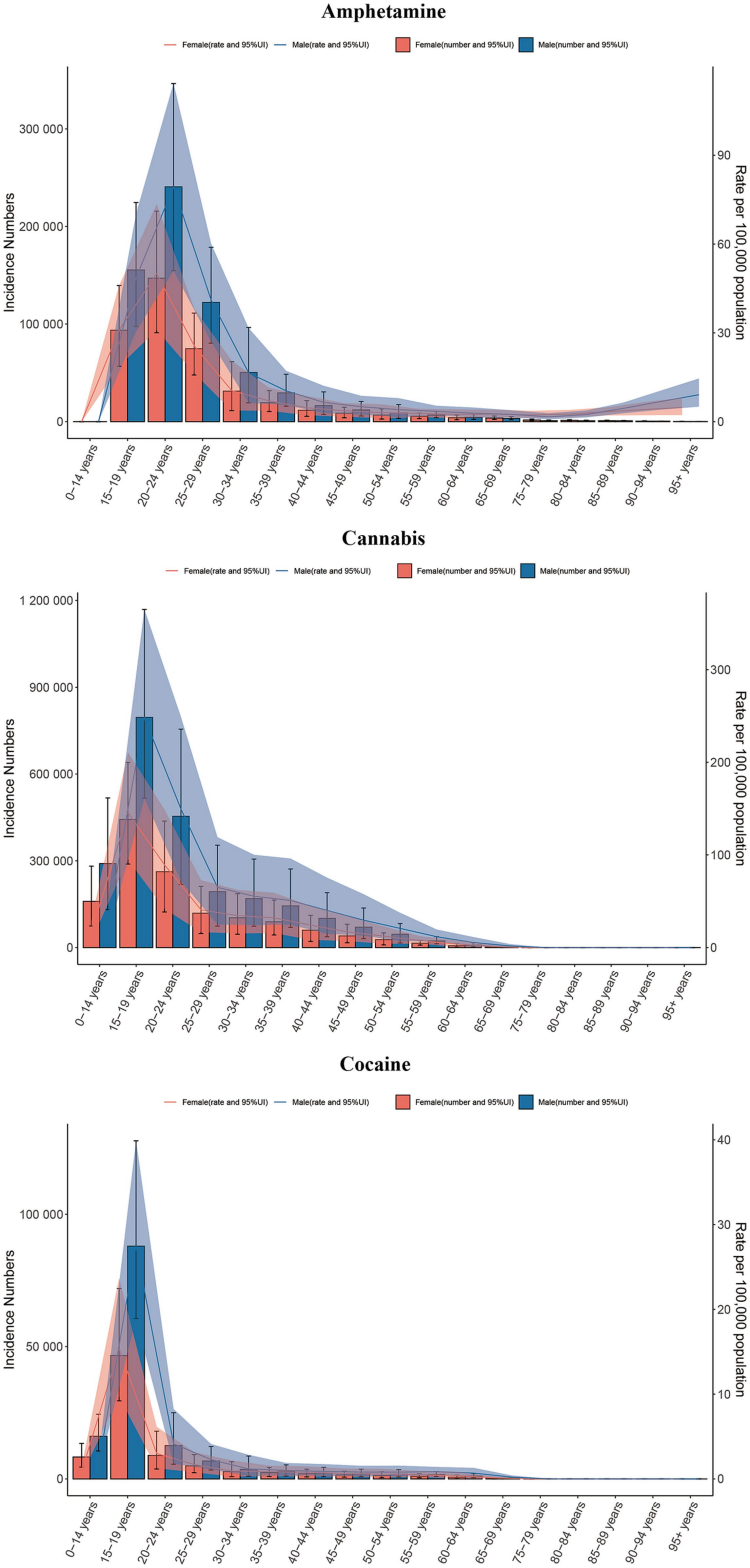
Global incidence of amphetamine, cannabis and cocaine use disorders by age and sex in 2021.

**Figure 8:**
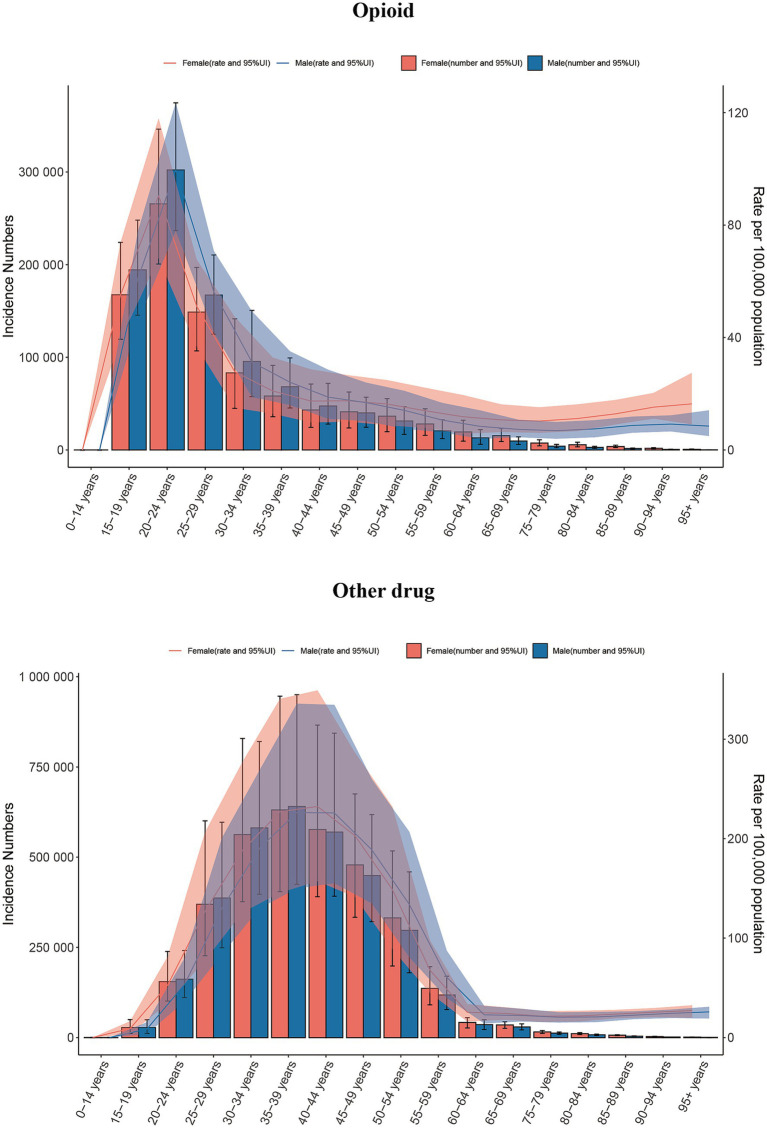
Global incidence of opioid and other drug use disorders by age and sex in 2021.

We analyzed the age and gender distribution maps of drug use disorders in 2021 and reached several conclusions. Specifically, the highest incidence of opioid use disorder occurs in the age group of 20–24 years, while the highest prevalence is observed in the age group of 25–29 years. Additionally, the highest mortality and DALYs are predominantly found in the age group of 25–39 years. For cocaine and cannabis use disorder, the highest incidence occurs in the age group of 15–19 years, the highest prevalence in 20–24 years, and the highest DALYs in 30–34 years. Notably, cocaine use disorder exhibits the highest mortality rate among individuals aged 30 to 39, whereas cannabis use disorder reports a mortality rate of zero. The highest incidence of amphetamine use disorder is also found in the age group of 20–24 years, with the highest prevalence and DALY values occurring in the age group of 25–29 years, and the highest mortality rates in 30–34 years. The incidence, prevalence, mortality, and DALYs of other drug use disorder predominantly affect the populations in the 30–34 and 35–39 age groups (Figures 7, 8; Supplementary Figures S8–S10). Furthermore, the age-specific incidence of the five drug use disorders indicates a trend towards younger populations. It is important to note that, with the exception of cannabis and cocaine use disorder in the youngest age group, none of the remaining three drug use disorders were present (Figures 7, 8; Supplementary Figures S8–S10).

In 2021, men represented the majority of individuals affected by five drug use disorders, exhibiting significantly higher rates of incidence, prevalence, mortality, and DALYs compared to women. Notably, the incidence and prevalence of opioid and other drug use disorder are comparable between men and women, with certain age groups even exceeding male rates. Specifically, the prevalence of opioid use disorder is highest among individuals aged 45–49 and in older age groups, while the prevalence of other drug use disorder peaks among those aged 40–44 and older. In these cases, females surpass males (Figures 7, 8; Supplementary Figures S8–S10).

### Attributable burden of 5 drug use disorders caused by risk factors

The risk factors associated with five drug use disorders primarily include drug use and behavioral risks, with both factors exhibiting consistent values in terms of DALYs and mortality rates, whether in 1990 or 2021. Notably, the DALYs attributed to these five drug use disorders resulting from both risk factors exceeded the corresponding mortality rates (Table 3).

**Table 3:** Percentage of 5 drug use disorders deaths and DALYs attributed to risk factors in 1990 and 2021.

Year	Amphetamine use disorders	Cocaine use disorders	Cannabis use disorders	Opioid use disorders	Other drug use disorders
1990					
DALYs	31.97 (20.83, 46.77)	10.94 (7.81, 15.02)	9.22 (5.39, 14.33)	101.52 (79.53, 120.73)	13.38 (10.89, 17.72)
Deaths	0.09 (0.08, 0.11)	0.07 (0.06, 0.08)	–	0.78 (0.69, 0.84)	0.22 (0.18, 0.31)
2021					
DALYs	21.26 (14.84, 29.70)	14.36 (11.62, 18.08)	8.29 (4.94, 12.89)	142.15 (116.40, 166.71)	11.13 (10.13, 12.31)
Deaths	0.13 (0.11, 0.14)	0.16 (0.14, 0.18)	–	1.26 (1.18, 1.37)	0.19 (0.18, 0.21)

The unit of deaths and DALYs is (×10^5^, 95%UI).

### Predicted ASIR of five drug use disorders from 2021 to 2035

**Figure 9:**
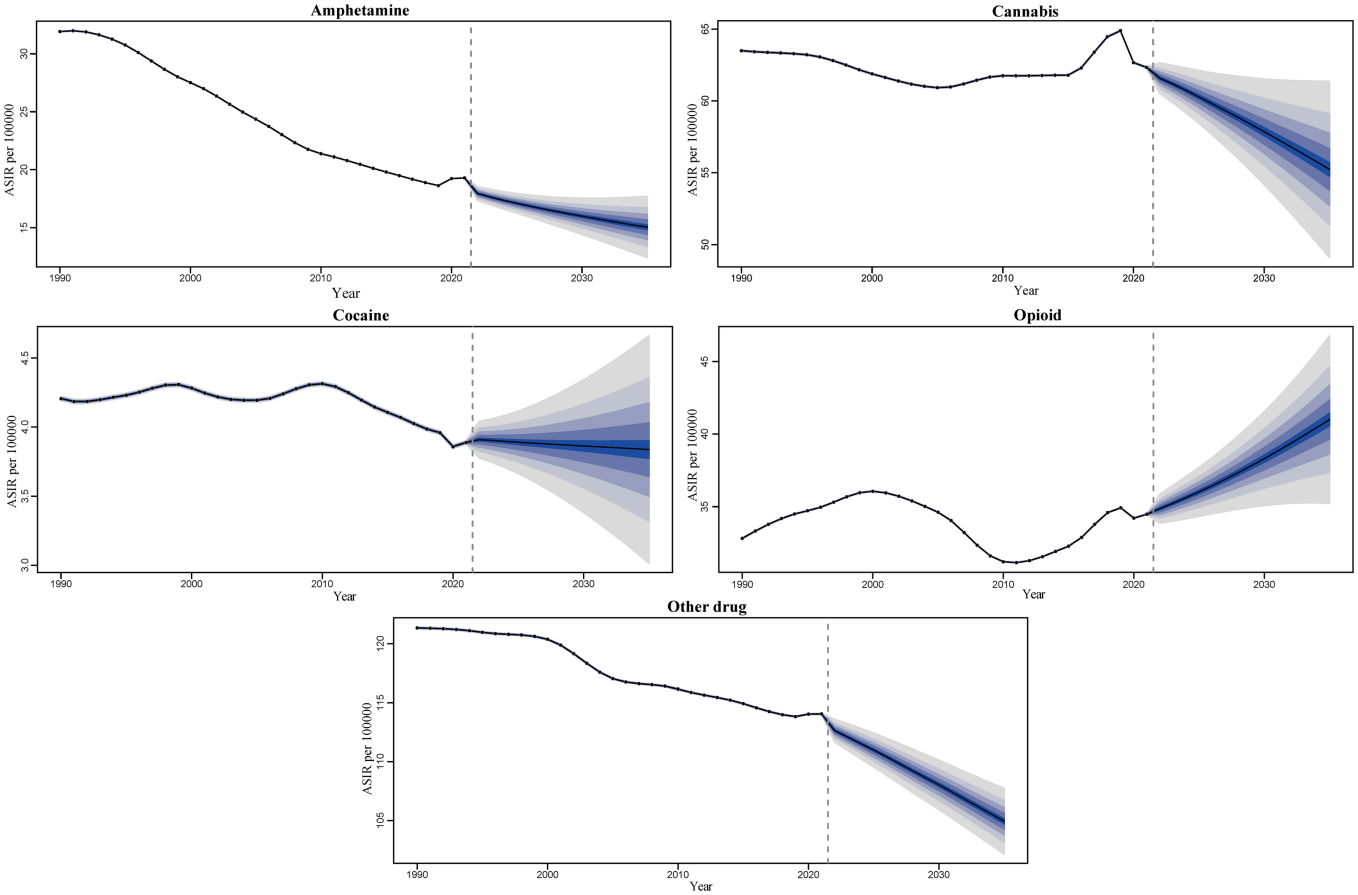
Trends and forecast rates of 5 drug use disorders ASIR worldwide change from 2021 to 2035.

Observations indicate that, among the five drug use disorders, only opioid use disorder is projected to rise over time, increasing from 34.48 per 100,000 people in 2021 to 41 per 100,000 by 2035. In contrast, the remaining four drug use disorders—amphetamine, cannabis, and other drug use disorder—exhibit a significant downward trend. It is estimated that by 2035, the ASIR for these three drug use disorders will decrease to 15.05, 55.23, and 104.94 per 100,000 cases worldwide, respectively. The ASIR for cocaine use disorder demonstrates a slight downward trend, projected to decline from 3.89 cases per 100,000 in 2021 to 3.84 cases per 100,000 by 2035 (Figure 9; Supplementary Table S10).

### Decomposition analysis of change in incidence

Figure 6 and Supplementary Table S11 present the results of the incidence breakdown analysis concerning changes in three population-level determinants: aging, population, and epidemiological changes, across five SDI categories and 21 regions worldwide. The decomposition analysis revealed that, on a global scale, the burden of amphetamine use disorder has decreased, with epidemiological changes accounting for the primary contribution to this decline (212.64%). Conversely, the burden associated with the remaining four drug use disorders has increased, primarily driven by changes in population dynamics.

**Figure 10:**
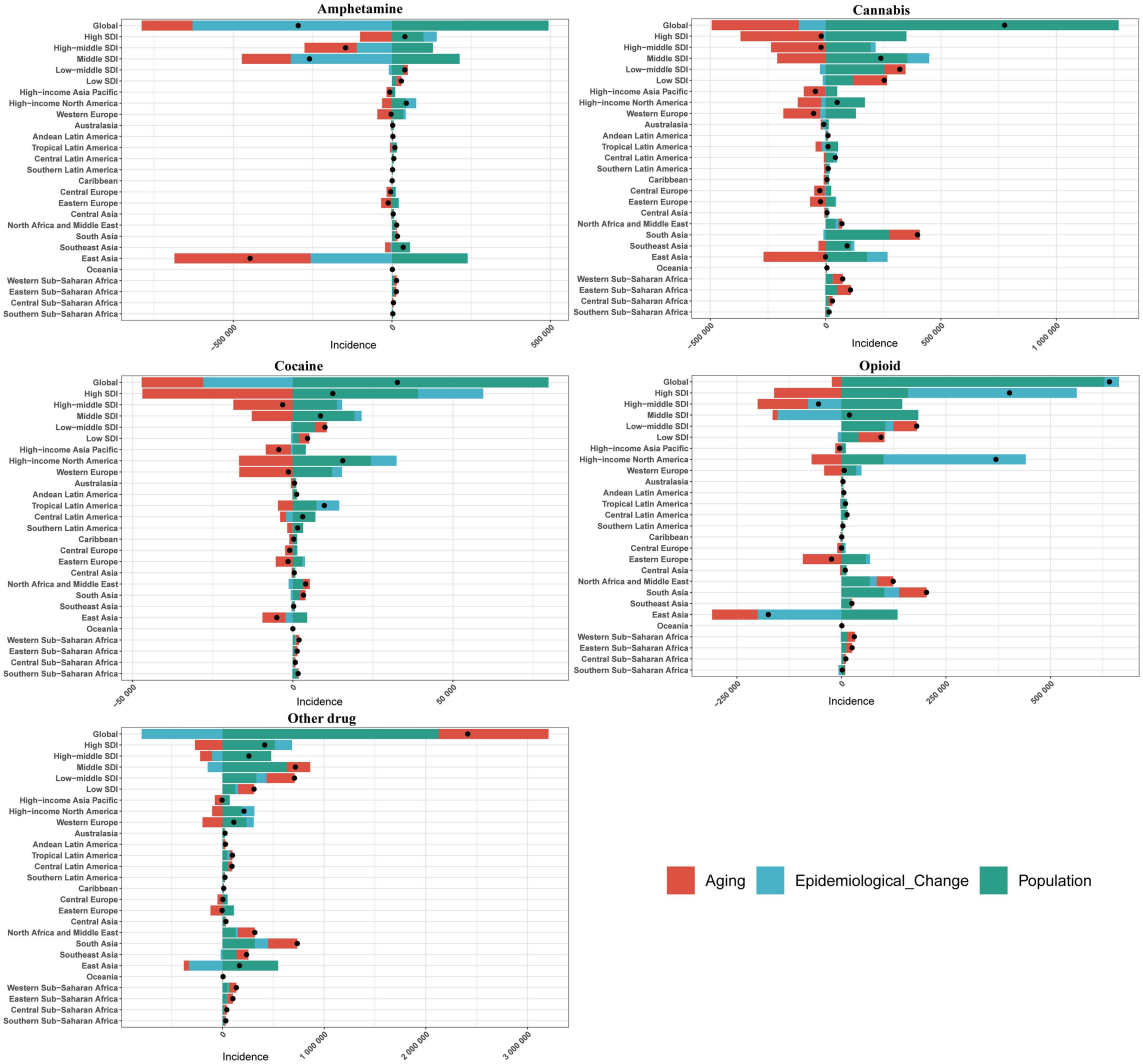
Change in incidence of 5 drug use disorders decomposed by three population-level determinants: aging, population and epidemiological change at the global level and various regions. The black dots indicate the total value of change attributable to all three components.

For amphetamine use disorder, among the various SDI regions, the High SDI region is the most significantly impacted by aging (−245.73%) and population changes (244.12%), while the Middle SDI region is most affected by epidemiological changes (122.71%). In contrast, aging has the least impact on the Low-middle SDI region (36.43%), and both population and epidemiological changes exert minimal influence on the Low-SDI region, with effects of 42.84 and 3.11%, respectively. This indicates that the contributions of the three determinants to incidence vary across regions. Aging has a positive contribution to incidence in all SDI regions except for the High SDI region (−245.73%). Similarly, populations in all regions, except for the High-middle SDI and Middle SDI regions, also contribute positively to incidence (−88.22% and −82%). Moreover, with the exception of the Low-middle SDI area, the contribution of epidemiological changes to incidence is positive across all SDI areas (−23.69%). Notably, in Western Europe, epidemiological changes (−295.43%), population changes (−1365.52%), and aging (1760.95%) are the largest contributors to overall incidence changes. Among the various regions, Oceania (0.18%), Central Sub-Saharan Africa (40.74%), and Central Latin America (−2.55%) are the least affected by epidemiological changes, population shifts, and aging (Figure 10; Supplementary Table S11).

In the context of cannabis use disorder, the High SDI region exhibits the greatest susceptibility to aging (1892.48%) and population changes (−1838.17%), as well as the most significant impact from epidemiological shifts (−106.09%). Conversely, aging has the least effect on Low-middle SDI areas (28.49%), while population and epidemiological changes exert minimal influence on Low-SDI regions, recording values of 46.72% and −4.78%, respectively. This analysis indicates varying contributions of the three determinants to incidence across different SDI regions. Notably, aging contributes positively to incidence in all SDI regions except for the Middle SDI regions (−87.69%). In addition, population changes positively influence incidence in all regions except for the High-middle SDI and High SDI areas, which show contributions of −982.44% and −1838.17%, respectively. Furthermore, the impact of epidemiological changes is negative in all SDI areas except for the High SDI and Middle SDI regions, with contributions of 45.68 and 39.34%, respectively. It is particularly noteworthy that the overall incidence changes are most significantly influenced by epidemiological changes (−14261.34%), population changes (−28718.79%), and aging (43080.13%). Among various regions, Central Sub-Saharan Africa, Western Sub-Saharan Africa, and Southern Sub-Saharan Africa experience the least impacts from epidemiological changes (0.21%), population (39.27%), and aging (−9.27%), respectively (Figure 10; Supplementary Table S11).

In the context of cocaine use disorder, among various SDI regions, the High-middle SDI region is the most significantly affected by aging (586.65%) and population dynamics (−436.45%), while the High SDI region experiences the greatest impact from epidemiological changes (162.76%). Conversely, both aging and epidemiological changes exert the least influence on Low-middle SDI areas (36.43% and −5.34%, respectively), and the population has the minimal impact on Low-SDI areas (42.18%). These findings indicate notable differences in the contributions of the three determinants to incidence. Aging has a positive contribution to incidence across all SDI regions, except for the High SDI and Middle SDI regions, which show negative contributions (−375.73% and −147.77%, respectively). With the exception of the High-middle SDI region, the population’s contribution to incidence is positive in all other SDI regions (−436.45%). Additionally, the contribution of epidemiological changes is negative in all SDI areas except for the High SDI and Middle SDI regions (162.76 and 25.22%, respectively). Notably, epidemiological changes (−225.88%), population dynamics (−890.27%), and aging (1216.15%) represent the largest contributors to overall incidence changes in Western Europe. Among the various regions, Eastern Sub-Saharan Africa (−4.89%), Western Sub-Saharan Africa (34.22%), and Andean Latin America (−8.44%) demonstrate the least impacts from epidemiological changes, population dynamics, and aging (Figure 10; Supplementary Table S11).

Opioid use disorder exhibits significant variability across different SDI regions. The Middle SDI region is the most affected by population changes, with an increase of 957.41%, and by epidemiological shifts, which show a decrease of 794.95%. Conversely, the High-middle SDI area experiences the greatest impact from aging, with a positive contribution of 219.43%. Aging has the least effect on Low-middle SDI areas (30.89%), while population changes exert minimal influence on High SDI areas (39.69%). Additionally, epidemiological changes have the smallest impact on Low SDI regions, recorded at −9.01%. These findings indicate notable differences in the contributions of the three determinants to incidence. Aging positively influences incidence across all SDI regions, except for the High SDI and Middle SDI regions, which show negative contributions of −40% and −62.46%, respectively. With the exception of the High-middle SDI region, the population’s contribution to incidence is positive across all SDI regions, totaling −264.68%. In contrast, epidemiological changes contribute positively to incidence rates in all SDI areas except for Low SDI and Middle SDI regions, which exhibit rates of −9.01% and −794.95%, respectively. Notably, in Central Europe, epidemiological changes (−20,727.75%), population changes (−45,442.72%), and aging (66,270.47%) represent the largest contributors to overall incidence changes. Among various regions, Eastern Sub-Saharan Africa (0.6%), High-income North America (27.26%), and Tropical Latin America (−7.57%) are identified as the areas least affected by epidemiological changes, population dynamics, and aging (Figure 10; Supplementary Table S11).

For other drug disorder, among various SDI regions, the High SDI region exhibits the most significant impact from aging (−64.97%) and epidemiological changes (40.78%), followed by the High-middle SDI area. Conversely, aging has the least effect on Middle SDI regions (32.18%), while demographic and epidemiological changes minimally influence Low SDI regions (40.14 and 8.66%, respectively). These findings indicate notable differences in the contributions of the three determinants to incidence. Aging positively contributes to incidence across all SDI regions, except for the High-middle SDI and High SDI regions, where it shows negative contributions (−45.15% and −64.97%, respectively). In contrast, the contribution of populations to incidence remains positive in all SDI regions. With the exception of the High-middle SDI and Middle SDI areas, epidemiological changes in the remaining SDI regions also positively affect the incidence rate (−39.01% and −20.29%). Notably, the contributions of population (−3078.29%) and aging (3351.85%) to the overall incidence change are the largest, whereas epidemiological change (211.08%) is most pronounced in Central Europe. Among the various regions, Oceania (0.06%), Central Sub-Saharan Africa (38.09%), and Australia (−0.52%) are the least affected by changes in epidemiological factors, population, and aging (Figure 10; Supplementary Table S11).

### Health inequality analysis of DALYs

**Figure 11:**
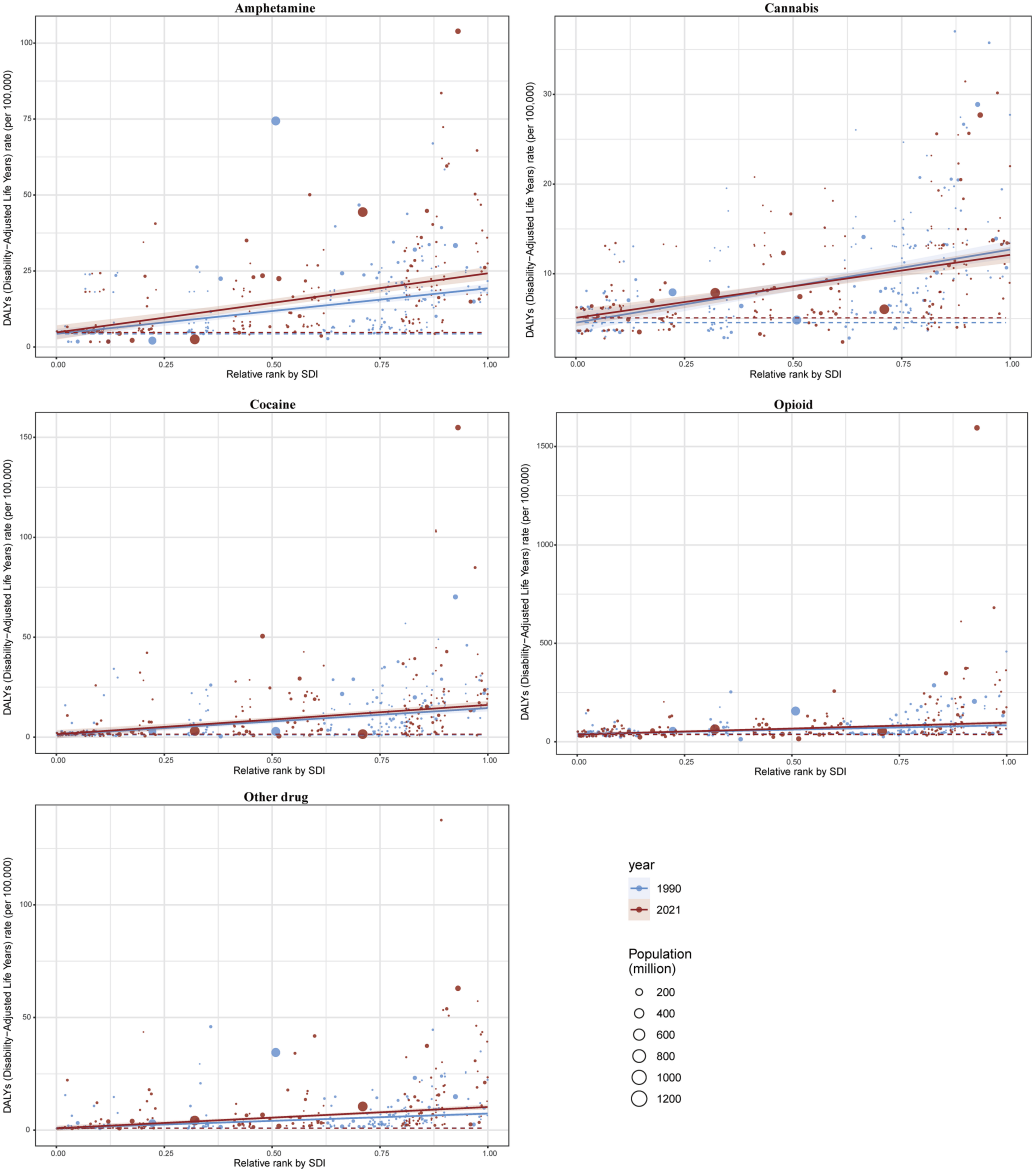
Health inequality regression curves for the incidence.

**Figure 12:**
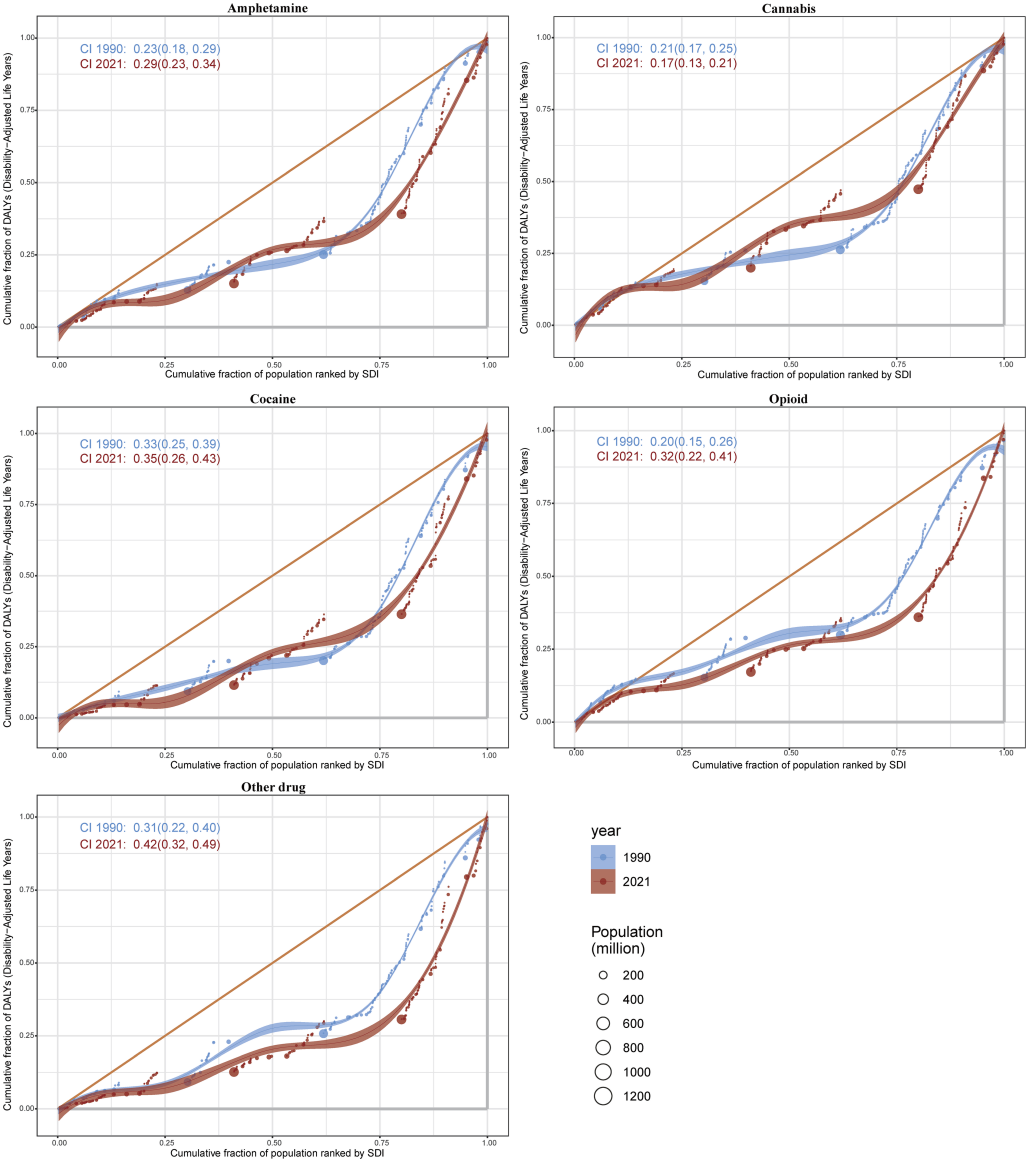
Health concentration curves for the incidence.

The results indicate significant absolute and relative income inequality in the burden of DALYs associated with drug use disorders, with this burden disproportionately concentrated in wealthier areas. A comparison of data from 1990 and 2021 reveals an increase in health inequality over this period. In 2021, the inequality slope index for the five drug use disorders showed a slight increase, with amphetamine use disorder experiencing the most substantial rise. The concentrated index for all five drug use disorders has significantly increased, with amphetamine use disorder rising from 0.23 (95% CI 0.18, 0.29) in 1990 to 0.29 (95% CI 0.23, 0.34) in 2021. Cannabis use disorder decreased from 0.21 (95% CI 0.17, 0.25) in 1990 to 0.17 (95% CI 0.13, 0.21) in 2021, while cocaine use disorder increased from 0.33 (95% CI 0.25, 0.39) in 1990 to 0.35 (95% CI 0.26, 0.43) in 2021. Opioid use disorder rose from 0.20 (95% CI 0.15, 0.26) in 1990 to 0.32 (95% CI 0.22, 0.41) in 2021, and other drug use disorder increased from 0.31 (95% CI 0.22, 0.40) in 1990 to 0.42 (95% CI 0.32, 0.49) in 2021. These findings collectively indicate an increased burden of inequality in DALYs for drug use disorders (Figures 11, 12).

## Discussion

This study examines the magnitude and temporal trends of drug use disorder-related burdens globally and across various countries over the past three decades, while also predicting the trajectory of these burdens over the next 15 years. Additionally, it presents the results of an incidence breakdown analysis that explores changes in three population-level determinants (aging, populations, and epidemiological changes) across five SDI categories and 21 regions worldwide. Through an analysis of health inequality concerning DALYs, the study quantifies the disparities in disease burden across socio-economic, geographical, and demographic dimensions. Overall, the burden associated with the five drug use disorders exhibited an upward trend in more than half of the GBD regions from 1990 to 2021. However, according to our predictive analysis, all drug use disorders, except for opioid use disorder, are anticipated to experience a downward trend over the next 15 years. This decline may be attributed to the strengthening of relevant policies and legal interventions, as strict regulations on prescription drugs, including illicit substances and opioids, in various countries have significantly reduced usage rates and curtailed abuse trends. Furthermore, advancements in treatment and rehabilitation strategies, such as the integration of pharmacological therapies with psychological interventions, may also contribute to this positive trend.

From a drug category perspective, cannabis remains the most widely used drug globally, with 228 million users among the five drug use disorders (6). However, its associated mortality and DALYs are not the highest. Opioid use has also reached 600,000 users (6), which may be related to listing it as a prescription drug. Prescription practices not only enhance the availability of opioids but also elevate the risk of both legal pain management and illegal transfers in non-medical settings (24). Notably, opioid use disorder has the most significant impact on human health, with studies indicating that opioid use increases the risk of major depression, anxiety, and stress-related disorders (25). Some researchers have suggested that mindful interventions and other strategies could help mitigate the prevalence of opioid use disorder (26). Furthermore, a substantial body of literature has demonstrated that drug-assisted treatments can enhance safety during use (27). However, in practice, public adherence to alternative treatments and other methods has been found to be low. Data indicates that in 2020, only 11% of individuals diagnosed with opioid use disorder in the United States received alternative treatment (28), despite the U.S. being the country most severely affected by opioid disorder. This phenomenon may be a significant factor contributing to the projected increase in the burden of opioid use disorder. We also note that in the GBD estimation results, the incidence rates of other drug use disorder are higher than their point prevalence. This seemingly counterintuitive phenomenon is a recognized feature within the GBD modeling framework. It arises from the high remission rates estimated by the DisMod-MR model for these disorders (29). Given that drug use disorders often follow a chronic, relapsing, and intermittent course, there are substantial instances of ‘natural recovery’ and short-term remission (30). This leads to a relatively short average duration in which individuals remain in a ‘disease state’ (31). Consequently, although the number of new cases (incidence) is high, the total number of cases present at any specific point in time (prevalence) remains relatively low. This methodological approach has been repeatedly clarified by the GBD research team and aligns with the clinical reality of these disorders.

In 2021, drug use disorders were found to be more severe in men than in women across most age groups. This disparity may be linked to the differences in metabolic processes between genders and the involvement of various metabolic pathways in the elimination of substances (32–34). Our study also revealed that the prevalence of opioid use disorder exceeded that of men in the age group of 45–49 and older, which may be due to differing risk factors for opioid use disorder between the sexes. For women, the risk factors associated with relapse after treatment for opioid use disorder include more significant drug use problems and withdrawal symptoms. In contrast, for men, younger age, behavioral disorders, and a history of multiple substance use disorders are the primary risk factors (35). Additionally, women may face more barriers in accessing medication, which can lead to delayed treatment (6).

Regardless of gender, the burden of drug use disorders is highest among individuals aged 15 to 45. The adolescent brain is often likened to a vehicle equipped with a relatively weak braking system; the accelerator, represented by the dopamine reward system, operates vigorously, while the brakes, associated with the prefrontal cortex, are insufficient. Individuals in this age group are frequently motivated to pursue happiness and avoid discomfort. However, their judgment and decision-making abilities are still maturing and relatively limited, which impairs their capacity to accurately assess risks and make prudent choices, particularly concerning drug use (36). Furthermore, the study revealed that the attributable risk factors for the five drug use disorders primarily fell into two categories: drug use and behavioral risks. Interventions should prioritize this life stage through school- and community-based programs focusing on cognitive behavioral training and emotional regulation to build resilience (37). Family screening and support systems are also crucial for early risk detection and support (38). Policy measures are needed to limit youth access to addictive substances and improve targeted health education to reduce DUDs risk.

Our results indicate that, in 2021, the EAPC of drug use disorders (DUDs) globally exhibited a negative correlation with the overall SDI. Furthermore, the burden of DALYs attributed to drug use disorders was disproportionately concentrated in wealthier regions. Populations residing in areas with high SDI generally experience elevated socio-economic levels, which may be linked to higher rates of substance abuse initiation (39). The concentrated burden in high-SDI regions may stem from several non-exclusive mechanisms: surveillance and diagnostic biases from better healthcare systems increase detection rates; greater purchasing power enhances substance availability; and high income inequality promotes drug use as a coping mechanism through stress and mental health impacts. Beyond SDI, unmeasured systemic factors such as drug policies, harm reduction services, cultural attitudes, and mental health treatment rates also contribute. Their interaction with economic development is complex. For instance, high-SDI countries with punitive policies show different burden patterns than those with health-centered approaches. Future research must disentangle these relationships to identify effective policy levers, incorporating political, cultural, and health-system determinants rather than economic indicators alone.

COVID-19 has significantly impacted the burden of drug use disorders. Recent evidence indicates that social isolation, economic distress, mental health challenges, and disruptions to treatment services caused by the pandemic have exacerbated the prevalence of drug use disorders (40–42). Some studies have suggested that biological factors associated with heightened stress, coupled with excessive immune system activity and resultant inflammation, may influence the functional status of the central nervous system (CNS), thereby increasing vulnerability to DUDs (42). Furthermore, the social isolation resulting from epidemic control measures has diminished opportunities for individuals to seek help (40).

According to the latest estimates from GBD 2021, this study provides a detailed analysis of the global burden of drug use disorders. However, it is not without limitations (9). Firstly, the analysis relies on an established GBD model, and the assumptions and parameter choices made could potentially influence the results. Secondly, data quality and availability are significant influencing factors. For instance, the disease surveillance systems in low- and middle-income countries are weak, with incomplete death and disease registration data, which may affect the reliability of our findings. The standards for data collection vary greatly among countries, and the heterogeneity of data sources could lead to systematic errors during integration. Thirdly, other factors also influence the burden of disease. For example, quantifying the impact of structural factors on health is challenging, leading to incomplete attribution analysis. Finally, the COVID-19 pandemic has introduced significant uncertainties into the estimation of all disease burdens, particularly in the regions most severely affected by the pandemic (40–42). Rankings based solely on point estimates can also be misleading when uncertainty intervals overlap considerably. Therefore, future research must enhance the accuracy and comprehensiveness of the results by supplementing them with additional data sources for cross-validation and employing other methods, such as formal statistical testing, to robustly determine the significance of observed spatial differences.

## Conclusion

Drug use disorders continue to represent a significant public health crisis globally, particularly among men and individuals aged 15 to 45 in high-income regions. Moving forward, it is essential to develop effective diagnostic screening tools, as well as high-quality prevention and treatment strategies, to address the prevention and treatment of drug use disorders.

## Data Availability

The datasets generated and analyzed during the current study are available in the GBD 2021 database, https://vizhub.healthdata.org/gbd-results/. The related data was contained in our articles and Additional files. Data visualization was performed using the R software package (version 4.2.3) and JD_GBDR (V2.36; Jingding Medical Technology Co., Ltd.) to visualize the global burden of pneumoconiosis through world maps. Specific R packages, including map, ggplot2, and dplyr, for example, were utilized in this analysis.

## Footnotes

^1^ http://ghdx.healthdata.org/gbd-results-tool
