# Supplementary material for: Global, regional and national burden of drug use disorders, 1990–2021: decomposition analysis, health inequality analysis and predictions to 2035
Source: Front Public Health. 2025 Oct 21;13:1588607. doi: 10.3389/fpubh.2025.1588607 (PMC12583096; doi:10.3389/fpubh.2025.1588607)

Additional file 1

Global, regional and national burden of drug use disorders, 1990-2021: decomposition analysis, health inequality analysis and predictions to 2035

Ruiying Jin[1, #a] , Shenyu Zhang[2, #a] , Jun Xiong[3*, #a], Baixi Liu[4, #a]

Hangzhou Normal University[#a],Hangzhou,Zhejiang,China

[*]Corresponding Author: Jun Xiong,20040044@hznu.edu.cn

Items

1 Search method for data from GBD 2021 Database.

2 Table S1: EAPCs of the ASIR and ASDR of 5 drug use disorders in global and 21 regions.

3 Table S2: EAPCs of the ASPR and ASMR of 5 drug use disorders in global and 21 regions.

4 Table S3: Regional DALYs and ASDR of the 5 drug use disorders in 2021.

5 Table S4: Regional prevalence and ASPR of the 5 drug use disorders in 2021.

6 Table S5: Regional deaths and ASMR of the 5 drug use disorders in 2021.

7 Table S6: Age-standardized incidence, prevalence, mortality, and DALY rates of the 5 drug use disorders among the top 3 and bottom 3 countries in 2021.

10 Table S7: EAPC of ASIR and ASDR for the 4 drug use disorders in 204 countries and territories from 1990 to 2021.

11 Table S8: EAPC of ASMR and ASPR for the 4 drug use disorders in 204 countries and territories from 1990 to 2021.

12 Table S9: EAPC of ASMR ,ASPR ,ASIR and ASDR for the amphetamine use disorders in 204 countries and territories from 1990 to 2021.

13 Table S10: Trends and forecast rates of 5 drug use disorders ASIR worldwide change from 2021 to 2035.

14 Table S11: Decomposition analysis of change in DALYs, incidence and deaths.

15 Figure S1: The EAPC of ASPR for 5 drug use disorders in global and 21 regions.

16 Figure S2: The EAPC of ASMR for 5 drug use disorders in global and 21 regions.

17 Figure S3: The EAPC of ASDR for 5 drug use disorders in global and 21 regions.

18 Figure S4: The global DALYs ,deaths and prevalence of 5 drug use disorders in 204 countries or territories in 2021.

19 Figure S5: ASPR of 5 drug use disorders for 204 countries and territories by SDI.

20 Figure S6: ASMR of 5 drug use disorders for 204 countries and territories by SDI.

21 Figure S7: ASDR of 5 drug use disorders for 204 countries and territories by SDI.

22 Figure S8: Global prevalence of 5 drug use disorders by age and sex in 2021.

23 Figure S9: Global deaths of 5 drug use disorders by age and sex in 2021.

24 Figure S10: Global DALYs of 5 drug use disorders by age and sex in 2021.

25 Figure S11: Change in DALYs of 5 drug use disorders decomposed by three population-level determinants: aging, population and epidemiological change at the global level and various regions.

26 Figure S12: Change in deaths of 5 drug use disorders decomposed by three population-level determinants: aging, population and epidemiological change at the global level and various regions.

Search method for data from GBD 2021 Database.

Data for this study were obtained from the Institute for Health Metrics and Evaluation (IHME) by accessing their official website (https://ghdx.healthdata.org/gbd-results). The specific search criteria in the "Search" interface were as follows: GBD Estimate (Cases of death or injury, risk factor), Measure (Incidence, Deaths, Prevalence, DALYs), Metric (Number, Percent, Rate), Cause (Opioid use disorders,Cocaine use disorders ,Amphetamine use disorders,Cannabis use disorders,Other drug use disorders), Location (Global, 21super regions, Different SDI regions, 204 countries and territories), Age (All ages, Age-standardized, 0–14 years, 15–19 years, 20–24 years, 25–29 years, 30–34 years, 35–39 years, 40–44 years, 45–49 years, 50–54 years, 55–59 years, 60–64 years, 65–69 years, 70–74 years, 75 – 79 years, 80–84 years, 85–89 years, 90–94 years, 95+ years), Sex (Both, male, female), Year(1990–2021, and each year from 1990 to 2021). Data we need for this study was all recorded from 1990 to 2021

Table S1. Table S1: EAPCs of the ASIR and ASDR of 5 drug use disorders in global and 21 regions.

	Amphetamine EAPCs		Cannabis EAPCs		Cocaine EAPCs		Opioid EAPCs		Other drug EAPCs	
	ASDR	ASIR	ASDR	ASIR	ASDR	ASIR	ASDR	ASIR	ASDR	ASIR
Global	-1.68(-1.88,-1.48)	-1.98(-2.09,-1.88)	-0.08(-0.14,-0.02)	-0.05(-0.10,0.00)	0.72(0.62,0.82)	-0.17(-0.27,-0.07)	0.50(0.30,0.71)	-0.17(-0.34,-0.00)	-1.44(-1.74,-1.15)	-0.24(-0.26,-0.23)
East Asia	-2.65(-3.06,-2.24)	-2.20(-2.52,-1.87)	0.49(0.28,0.70)	0.46(0.26,0.65)	-3.31(-3.84,-2.77)	-1.31(-1.55,-1.08)	-4.63(-5.11,-4.15)	-2.82(-3.17,-2.47)	-5.33(-6.05,-4.60)	-0.75(-0.86,-0.64)
Southeast Asia	-0.01(-0.07,0.05)	-0.11(-0.17,-0.05)	0.13(0.10,0.17)	0.11(0.08,0.15)	0.03(-0.10,0.17)	-0.38(-0.51,-0.26)	0.09(-0.01,0.19)	-0.08(-0.16,0.01)	0.24(0.10,0.37)	0.10(0.01,0.19)
Oceania	0.03(0.00,0.06)	0.02(0.00,0.04)	0.02(0.01,0.03)	0.01(0.00,0.02)	-0.84(-1.02,-0.67)	-0.18(-0.22,-0.13)	-0.27(-0.31,-0.22)	0.11(0.10,0.12)	-0.54(-0.68,-0.40)	-0.04(-0.09,0.02)
Central Asia	0.85(0.73,0.97)	0.24(0.22,0.27)	0.11(0.10,0.13)	0.09(0.08,0.10)	1.73(1.19,2.27)	0.21(0.16,0.25)	0.35(-0.08,0.78)	-0.07(-0.22,0.09)	1.50(0.67,2.33)	0.17(0.03,0.31)
Central Europe	0.84(0.79,0.88)	0.60(0.42,0.78)	-0.11(-0.17,-0.04)	-0.19(-0.25,-0.13)	-0.91(-1.01,-0.80)	0.17(0.02,0.32)	0.49(0.40,0.58)	0.55(0.42,0.67)	-0.28(-0.47,-0.09)	0.26(0.21,0.31)
Eastern Europe	0.87(0.32,1.41)	-0.09(-0.30,0.13)	0.05(-0.03,0.12)	0.04(-0.04,0.11)	-2.23(-2.77,-1.69)	0.10(-0.11,0.32)	-0.01(-0.88,0.88)	-0.57(-1.22,0.07)	0.29(-0.47,1.05)	0.19(0.09,0.28)
High-income Asia Pacific	0.03(-0.03,0.10)	-0.04(-0.08,0.00)	-0.02(-0.04,-0.01)	-0.05(-0.06,-0.03)	-0.09(-0.23,0.05)	-0.13(-0.22,-0.04)	-0.01(-0.21,0.19)	-0.02(-0.13,0.08)	-0.74(-1.09,-0.39)	-0.15(-0.25,-0.05)
Australasia	0.34(0.01,0.67)	-0.81(-1.18,-0.45)	-0.95(-1.15,-0.76)	-0.90(-1.07,-0.72)	0.56(0.42,0.71)	0.37(0.26,0.48)	-0.58(-1.02,-0.15)	-0.34(-0.76,0.07)	2.99(2.32,3.67)	0.20(0.13,0.26)
Western Europe	1.14(1.00,1.27)	0.22(0.06,0.38)	-0.06(-0.12,0.01)	-0.17(-0.25,-0.10)	0.66(0.33,0.99)	0.40(0.04,0.76)	0.25(-0.01,0.52)	-0.27(-0.59,0.05)	2.21(2.12,2.31)	0.34(0.30,0.38)
Southern Latin America	0.09(-0.02,0.20)	-0.08(-0.16,0.00)	0.70(0.63,0.77)	0.51(0.44,0.58)	0.40(0.18,0.61)	0.51(0.14,0.87)	0.02(-0.07,0.11)	-0.12(-0.20,-0.03)	0.29(0.14,0.43)	-0.03(-0.10,0.05)
High-income North America	3.84(3.15,4.52)	-0.01(-0.68,0.66)	-0.06(-0.11,-0.01)	-0.04(-0.10,0.01)	2.79(2.60,2.99)	-0.06(-0.27,0.14)	7.06(6.80,7.32)	5.72(5.10,6.34)	5.20(4.88,5.52)	0.84(0.77,0.91)
Caribbean	0.30(-0.05,0.64)	0.05(-0.05,0.14)	-0.01(-0.07,0.05)	0.02(-0.03,0.08)	-0.59(-1.47,0.30)	-0.17(-0.30,-0.04)	-1.20(-1.50,-0.89)	-0.59(-0.67,-0.51)	-0.06(-1.03,0.92)	-0.11(-0.14,-0.07)
Andean Latin America	0.70(0.63,0.76)	0.22(0.19,0.25)	0.04(0.01,0.06)	0.03(0.01,0.06)	0.94(0.64,1.24)	0.18(-0.12,0.48)	0.28(0.18,0.39)	0.14(0.06,0.22)	-0.53(-0.87,-0.19)	0.10(0.06,0.14)
Central Latin America	0.83(0.58,1.07)	0.50(0.19,0.82)	0.42(0.32,0.52)	0.32(0.24,0.40)	-0.16(-0.32,0.01)	0.79(0.41,1.16)	-0.25(-0.33,-0.17)	-0.17(-0.26,-0.07)	0.39(0.13,0.65)	-0.08(-0.12,-0.04)
Tropical Latin America	-0.14(-0.23,-0.05)	-0.19(-0.35,-0.04)	-0.36(-0.46,-0.27)	-0.26(-0.32,-0.19)	3.48(3.14,3.83)	1.85(1.61,2.09)	-0.15(-0.27,-0.04)	-0.22(-0.34,-0.10)	5.21(4.98,5.44)	0.76(0.71,0.81)
North Africa and Middle East	0.77(0.59,0.95)	0.29(0.22,0.37)	0.35(0.31,0.40)	0.28(0.24,0.32)	-0.53(-0.71,-0.34)	-0.08(-0.17,0.01)	0.34(0.13,0.55)	0.30(0.14,0.47)	0.34(-0.08,0.76)	0.25(0.22,0.28)
South Asia	0.34(0.24,0.44)	0.09(0.07,0.11)	0.24(0.10,0.37)	0.18(0.07,0.30)	-0.23(-0.43,-0.03)	-0.22(-0.32,-0.13)	0.03(-0.18,0.24)	0.13(-0.10,0.36)	0.76(0.59,0.92)	0.48(0.43,0.53)
Central Sub-Saharan Africa	0.20(0.18,0.23)	0.07(0.06,0.09)	0.04(0.03,0.05)	0.01(0.01,0.01)	0.44(0.29,0.59)	0.35(0.32,0.39)	0.52(0.44,0.61)	0.38(0.35,0.42)	0.38(0.23,0.54)	0.13(0.05,0.21)
Eastern Sub-Saharan Africa	0.16(0.15,0.17)	0.02(0.01,0.03)	0.08(0.02,0.13)	0.06(0.01,0.12)	-0.35(-0.47,-0.22)	0.43(0.42,0.45)	0.29(0.27,0.31)	-0.05(-0.09,-0.02)	0.63(0.50,0.76)	0.21(0.12,0.31)
Southern Sub-Saharan Africa	-0.18(-0.53,0.18)	-0.26(-0.50,-0.02)	0.02(-0.10,0.14)	0.00(-0.11,0.12)	0.28(-0.01,0.58)	0.40(0.18,0.62)	-1.32(-1.72,-0.92)	-1.44(-1.77,-1.10)	-0.20(-0.75,0.35)	0.21(0.18,0.24)
Western Sub-Saharan Africa	-0.02(-0.06,0.02)	-0.06(-0.09,-0.03)	0.05(0.01,0.09)	0.04(0.00,0.08)	0.60(0.48,0.72)	0.74(0.69,0.79)	-0.03(-0.07,0.02)	-0.03(-0.07,0.01)	0.70(0.61,0.80)	0.46(0.39,0.54)
High-middle SDI	-1.71(-1.91,-1.51)	-1.55(-1.70,-1.39)	0.06(-0.05,0.18)	0.08(-0.04,0.20)	-1.30(-1.67,-0.92)	-0.02(-0.23,0.18)	-1.75(-2.21,-1.29)	-1.36(-1.78,-0.95)	-2.71(-3.04,-2.38)	-0.36(-0.40,-0.32)
High SDI	2.01(1.69,2.32)	-0.01(-0.26,0.24)	-0.03(-0.06,0.00)	0.05(0.01,0.08)	2.17(2.02,2.32)	0.56(0.47,0.64)	4.98(4.72,5.24)	3.66(3.23,4.10)	3.40(3.22,3.58)	0.36(0.33,0.38)
Low-middle SDI	-0.24(-0.28,-0.21)	-0.36(-0.40,-0.32)	0.06(-0.03,0.15)	0.03(-0.05,0.11)	0.99(0.90,1.08)	0.30(0.15,0.45)	0.11(0.01,0.21)	0.09(-0.01,0.18)	0.84(0.75,0.94)	0.37(0.32,0.41)
Low SDI	0.13(0.11,0.15)	0.09(0.08,0.11)	0.02(-0.02,0.06)	-0.03(-0.06,0.00)	-0.40(-0.48,-0.31)	0.17(0.11,0.23)	0.21(0.16,0.27)	0.10(0.06,0.14)	0.97(0.88,1.07)	0.29(0.21,0.36)
Middle SDI	-2.72(-3.03,-2.41)	-2.34(-2.52,-2.17)	0.37(0.29,0.45)	0.33(0.26,0.40)	0.68(0.59,0.78)	0.78(0.54,1.02)	-2.50(-2.76,-2.24)	-1.25(-1.43,-1.08)	-3.71(-4.18,-3.24)	-0.42(-0.45,-0.39)

Table S2. EAPCs of the ASPR and ASMR of 5 drug use disorders in global and 21 regions.

	Amphetamine EAPCs		Cannabis EAPCs		Cocaine EAPCs		Opioid EAPCs		Other drugs EAPCs	
	ASPR	ASMR	ASPR	ASMR	ASPR	ASMR	ASPR	ASMR	ASPR	ASMR
Global	-2.05(-2.21,-1.89)	0.02(-0.38,0.43)	-0.09(-0.15,-0.03)	-	-0.22(-0.30,-0.14)	2.15(1.92,2.38)	0.50(0.32,0.69)	0.52(0.25,0.78)	-0.11(-0.13,-0.08)	-1.61(-1.99,-1.23)
East Asia	-2.31(-2.64,-1.97)	-4.04(-4.87,-3.21)	0.48(0.27,0.70)	-	-1.42(-1.67,-1.18)	-5.49(-6.48,-4.49)	-3.23(-3.62,-2.85)	-6.98(-7.80,-6.15)	-1.33(-1.50,-1.16)	-5.96(-6.81,-5.10)
Southeast Asia	-0.16(-0.23,-0.09)	3.23(3.01,3.46)	0.12(0.08,0.15)	-	-0.40(-0.53,-0.28)	0.21(0.02,0.40)	-0.03(-0.13,0.07)	0.41(0.30,0.51)	0.11(0.01,0.21)	0.42(0.30,0.54)
Oceania	-0.01(-0.03,0.02)	1.14(0.78,1.50)	0.01(0.00,0.02)	-	-0.22(-0.27,-0.16)	-1.05(-1.35,-0.74)	0.09(0.07,0.11)	-1.64(-1.93,-1.34)	-0.08(-0.15,-0.01)	-1.29(-1.55,-1.02)
Central Asia	0.35(0.32,0.37)	5.63(4.61,6.66)	0.11(0.10,0.12)	-	0.33(0.26,0.39)	2.93(2.07,3.80)	-0.02(-0.21,0.17)	1.73(0.71,2.76)	0.28(0.05,0.51)	1.88(0.89,2.87)
Central Europe	0.66(0.60,0.73)	2.78(2.60,2.96)	-0.11(-0.18,-0.05)	-	0.22(0.07,0.37)	-3.87(-4.60,-3.13)	0.60(0.50,0.69)	0.35(0.11,0.59)	0.46(0.39,0.54)	-0.64(-0.91,-0.37)
Eastern Europe	0.02(-0.20,0.25)	3.50(2.13,4.87)	0.03(-0.05,0.11)	-	0.30(0.05,0.55)	-4.07(-4.80,-3.32)	-0.36(-1.05,0.33)	0.32(-0.74,1.40)	0.49(0.33,0.64)	0.09(-0.66,0.84)
High-income Asia Pacific	-0.07(-0.12,-0.02)	4.39(3.29,5.50)	-0.03(-0.04,-0.02)	-	-0.13(-0.23,-0.04)	0.76(-0.13,1.65)	-0.10(-0.22,0.02)	0.76(-0.13,1.65)	-0.24(-0.41,-0.08)	-1.26(-2.12,-0.39)
Australasia	-0.33(-0.72,0.06)	8.72(7.49,9.96)	-0.96(-1.16,-0.77)	-	0.36(0.22,0.50)	7.22(5.76,8.69)	0.13(-0.19,0.44)	-1.02(-1.88,-0.15)	0.99(0.84,1.14)	3.70(2.97,4.43)
Western Europe	0.34(0.24,0.44)	5.97(5.38,6.56)	-0.06(-0.12,0.01)	-	0.44(0.06,0.82)	1.74(1.47,2.01)	0.42(0.12,0.71)	0.73(0.45,1.01)	0.93(0.88,0.98)	2.84(2.71,2.97)
Southern Latin America	-0.10(-0.22,0.02)	7.54(6.84,8.24)	0.70(0.64,0.77)	-	0.32(0.11,0.54)	4.25(3.69,4.81)	-0.30(-0.38,-0.22)	4.50(3.87,5.14)	-0.10(-0.23,0.03)	1.38(0.87,1.89)
High-income North America	-0.04(-0.93,0.85)	11.28(10.47,12.11)	-0.05(-0.10,0.00)	-	0.41(0.26,0.57)	7.03(6.43,7.63)	6.35(5.82,6.88)	7.98(7.73,8.23)	2.54(2.40,2.68)	5.93(5.53,6.32)
Caribbean	-0.16(-0.35,0.03)	4.03(2.32,5.78)	-0.01(-0.08,0.05)	-	-0.20(-0.36,-0.04)	-0.70(-2.51,1.15)	-0.89(-1.01,-0.77)	-2.69(-4.23,-1.12)	-0.31(-0.34,-0.28)	0.25(-1.16,1.69)
Andean Latin America	0.24(0.20,0.27)	4.96(4.39,5.53)	0.03(0.01,0.06)	-	0.20(-0.04,0.44)	1.57(1.21,1.93)	0.13(0.04,0.22)	1.73(1.35,2.11)	0.09(0.03,0.16)	-0.46(-0.86,-0.06)
Central Latin America	0.39(0.07,0.71)	3.66(3.33,3.99)	0.42(0.32,0.51)	-	-0.09(-0.24,0.05)	-0.37(-0.77,0.02)	-0.21(-0.31,-0.10)	-0.85(-1.11,-0.57)	-0.25(-0.29,-0.20)	0.62(0.25,1.00)
Tropical Latin America	-0.21(-0.30,-0.11)	4.68(3.68,5.68)	-0.37(-0.47,-0.28)	-	1.76(1.46,2.05)	7.47(6.51,8.44)	-0.27(-0.40,-0.13)	5.08(4.31,5.86)	1.03(0.96,1.09)	7.19(6.86,7.51)
North Africa and Middle East	0.44(0.33,0.55)	1.74(1.33,2.16)	0.35(0.31,0.39)	-	0.03(-0.04,0.09)	-0.96(-1.19,-0.73)	0.44(0.23,0.64)	0.08(-0.14,0.30)	0.39(0.34,0.44)	0.39(-0.05,0.83)
South Asia	0.12(0.09,0.14)	1.00(0.68,1.32)	0.22(0.09,0.36)	-	-0.23(-0.32,-0.13)	-0.08(-0.30,0.15)	0.14(-0.10,0.37)	-0.27(-0.48,-0.06)	0.61(0.53,0.68)	1.09(0.87,1.31)
Central Sub-Saharan Africa	0.07(0.06,0.09)	2.92(2.47,3.37)	0.01(0.01,0.01)	-	0.39(0.34,0.43)	0.52(0.21,0.83)	0.39(0.35,0.43)	0.58(0.36,0.80)	0.21(0.10,0.33)	1.20(0.77,1.63)
Eastern Sub-Saharan Africa	0.02(0.00,0.03)	1.90(1.61,2.18)	0.04(-0.01,0.10)	-	0.47(0.45,0.48)	-0.85(-1.06,-0.64)	-0.07(-0.11,-0.02)	0.21(0.13,0.29)	0.32(0.21,0.44)	1.26(1.05,1.47)
Southern Sub-Saharan Africa	-0.48(-0.82,-0.13)	2.62(1.95,3.28)	0.03(-0.10,0.15)	-	0.66(0.36,0.95)	-0.10(-0.45,0.26)	-1.70(-2.10,-1.29)	-0.22(-0.66,0.22)	0.29(0.24,0.33)	0.06(-0.45,0.57)
Western Sub-Saharan Africa	-0.08(-0.12,-0.04)	3.53(2.30,4.79)	0.03(-0.01,0.07)	-	1.00(0.95,1.05)	-0.03(-1.05,1.01)	-0.03(-0.08,0.02)	-0.62(-1.27,0.04)	0.69(0.59,0.78)	3.43(2.45,4.42)
High-middle SDI	-1.64(-1.83,-1.46)	-2.07(-2.45,-1.70)	0.05(-0.06,0.17)	-	-0.27(-0.68,0.13)	-3.44(-3.99,-2.90)	-1.43(-1.84,-1.02)	-2.42(-2.91,-1.92)	-0.57(-0.62,-0.52)	-3.20(-3.58,-2.82)
High SDI	0.04(-0.29,0.37)	8.80(8.25,9.36)	-0.02(-0.05,0.01)	-	0.49(0.40,0.58)	5.90(5.39,6.42)	4.50(4.09,4.90)	5.80(5.64,5.97)	1.44(1.37,1.52)	4.04(3.81,4.26)
Low-middle SDI	-0.37(-0.42,-0.32)	0.79(0.60,0.98)	0.05(-0.04,0.14)	-	0.83(0.60,1.05)	0.97(0.76,1.17)	0.09(0.00,0.18)	0.05(-0.10,0.19)	0.45(0.39,0.52)	1.21(1.07,1.35)
Low SDI	0.10(0.08,0.12)	-0.10(-0.32,0.13)	-0.00(-0.04,0.04)	-	0.31(0.28,0.35)	-1.01(-1.16,-0.85)	0.10(0.05,0.15)	0.20(0.11,0.29)	0.37(0.27,0.48)	1.49(1.34,1.64)
Middle SDI	-2.48(-2.71,-2.25)	-3.56(-4.31,-2.82)	0.36(0.28,0.44)	-	0.72(0.56,0.87)	0.40(0.13,0.68)	-1.48(-1.66,-1.30)	-4.30(-4.83,-3.77)	-0.85(-0.91,-0.79)	-4.06(-4.60,-3.50)

Table S3. Regional DALYs and ASDR of the 5 drug use disorders in 2021

location_name	Amphetamine		Cannabis		Cocaine		Opioid		Other drug	
	DALYs	ASDR	DALYs	ASDR	DALYs	ASDR	DALYs	ASDR	DALYs	ASDR
Global	21.26(14.84,29.70)	20.98(14.56,29.33)	8.29(4.94,12.89)	8.27(4.90,12.86)	14.37(11.63,18.10)	13.88(11.18,17.52)	142.16(116.44,166.76)	137.15(112.29,161.39)	11.14(10.14,12.31)	10.69(9.74,11.80)
East Asia	39.02(26.23,56.48)	44.67(29.64,65.46)	5.52(3.23,8.47)	6.02(3.44,9.65)	1.51(1.00,2.08)	1.51(0.99,2.13)	60.26(46.29,73.78)	54.37(40.97,67.47)	11.57(9.12,14.09)	10.66(8.40,12.98)
Southeast Asia	28.40(16.62,43.94)	26.84(15.71,41.66)	8.26(4.75,13.25)	7.85(4.52,12.65)	1.21(0.89,1.77)	1.13(0.84,1.66)	35.02(26.79,43.07)	32.90(25.23,40.51)	2.61(1.90,3.40)	2.49(1.81,3.22)
Oceania	19.63(10.61,32.22)	18.44(10.09,30.05)	14.07(7.48,24.08)	13.18(7.15,22.17)	0.90(0.63,1.39)	0.92(0.65,1.41)	33.05(23.47,43.82)	34.29(24.39,44.64)	1.85(1.25,2.70)	1.98(1.36,2.89)
Central Asia	20.96(13.49,30.28)	20.19(12.99,29.11)	5.72(3.02,9.51)	5.78(3.01,9.70)	8.44(6.53,10.62)	8.07(6.23,10.21)	123.04(93.45,151.15)	117.72(89.49,144.38)	7.36(6.08,8.72)	7.02(5.82,8.32)
Central Europe	22.48(14.16,33.98)	26.84(16.42,40.57)	7.84(4.91,11.83)	9.98(6.13,15.25)	6.07(4.16,8.84)	6.34(4.17,9.49)	65.03(53.80,75.89)	64.41(53.16,75.80)	6.28(5.35,7.39)	6.22(5.37,7.19)
Eastern Europe	34.14(25.39,45.63)	39.53(28.60,53.32)	7.79(4.52,12.23)	9.78(5.45,15.57)	12.60(10.21,15.86)	13.06(10.35,16.89)	318.09(268.62,370.59)	311.15(259.01,365.83)	30.78(27.83,33.91)	29.60(26.91,32.45)
High-income Asia Pacific	12.23(7.34,18.86)	15.20(8.70,23.86)	10.50(6.07,16.97)	13.53(7.70,22.38)	13.94(8.63,21.34)	14.74(8.79,23.21)	42.54(30.19,54.45)	43.74(30.90,57.08)	3.10(2.26,4.31)	2.85(2.06,3.91)
Australasia	76.99(51.06,110.06)	81.67(53.61,117.56)	18.30(11.94,27.50)	20.65(13.45,31.16)	31.42(19.59,48.28)	32.77(20.01,51.15)	210.99(173.02,246.41)	205.48(168.06,240.26)	132.94(118.38,147.31)	123.67(109.73,137.69)
Western Europe	27.61(18.98,39.00)	32.15(21.68,45.88)	13.55(8.76,20.00)	17.31(11.24,25.56)	23.07(15.99,31.87)	24.70(16.67,35.05)	178.08(150.41,205.26)	178.12(149.99,207.30)	23.59(21.30,26.21)	24.08(21.83,26.68)
Southern Latin America	10.47(6.22,16.56)	10.07(5.93,15.92)	10.56(7.08,15.40)	10.67(7.19,15.54)	36.55(22.06,55.46)	35.08(21.05,53.62)	55.26(38.69,72.10)	51.70(36.07,67.75)	3.04(2.16,4.21)	2.77(1.96,3.84)
High-income North America	97.61(79.87,120.43)	98.53(79.65,122.87)	24.72(15.03,37.94)	27.88(16.97,42.78)	150.77(125.40,186.20)	147.83(121.82,183.99)	1504.74(1244.10,1740.26)	1502.44(1235.96,1740.10)	61.15(54.08,70.15)	59.67(52.73,68.53)
Caribbean	8.81(5.59,12.98)	8.49(5.37,12.56)	13.90(7.63,22.85)	13.73(7.44,22.62)	25.27(18.79,34.45)	24.47(18.12,33.54)	42.63(29.53,55.91)	40.77(28.15,53.52)	5.07(4.21,6.12)	4.83(4.01,5.83)
Andean Latin America	11.94(7.38,17.83)	11.02(6.90,16.44)	7.32(4.28,11.74)	6.85(4.03,10.91)	22.51(17.43,28.86)	21.44(16.63,27.46)	49.23(34.63,66.29)	46.21(32.59,61.99)	6.32(5.01,7.99)	6.07(4.81,7.66)
Central Latin America	9.33(6.10,13.35)	8.76(5.72,12.52)	8.43(5.25,12.56)	7.96(4.96,11.83)	27.17(20.07,36.35)	25.64(18.93,34.27)	42.86(29.85,56.24)	40.33(28.12,52.90)	6.07(5.28,7.02)	5.71(4.97,6.60)
Tropical Latin America	24.35(13.95,37.79)	23.26(13.32,36.42)	12.34(7.48,19.24)	12.10(7.27,19.12)	51.65(40.54,65.99)	49.14(38.40,63.14)	41.93(28.13,55.62)	38.51(25.72,51.57)	7.11(6.40,7.98)	6.65(5.99,7.45)
North Africa and Middle East	7.21(4.75,9.99)	6.83(4.51,9.43)	4.10(2.38,6.55)	3.90(2.27,6.24)	7.39(5.67,9.65)	7.17(5.50,9.34)	135.18(104.39,165.60)	128.78(99.59,157.46)	16.20(13.73,19.53)	15.27(12.94,18.46)
South Asia	2.56(1.73,3.63)	2.39(1.63,3.36)	8.23(4.88,13.28)	7.48(4.47,11.97)	2.96(2.05,4.08)	2.91(2.02,4.03)	64.47(48.27,80.06)	61.71(46.68,75.92)	4.17(3.45,5.17)	4.19(3.49,5.16)
Central Sub-Saharan Africa	5.30(2.91,8.36)	5.41(3.00,8.36)	5.27(2.80,8.99)	5.21(2.80,8.54)	1.91(1.23,3.04)	2.25(1.47,3.63)	38.08(27.75,49.66)	45.14(32.46,58.02)	1.00(0.60,1.48)	1.26(0.76,1.87)
Eastern Sub-Saharan Africa	5.24(2.98,8.07)	5.24(3.06,8.03)	6.79(3.67,11.27)	6.27(3.62,10.11)	1.86(1.15,3.32)	2.35(1.45,4.26)	41.71(32.11,51.63)	51.62(40.04,62.98)	0.85(0.55,1.28)	1.11(0.72,1.66)
Southern Sub-Saharan Africa	18.67(11.91,26.94)	17.50(11.31,24.89)	9.01(5.04,14.60)	8.39(4.76,13.56)	17.22(12.26,23.52)	17.28(12.43,23.48)	89.35(71.46,106.13)	88.95(71.72,105.11)	11.26(9.89,12.80)	11.18(9.83,12.74)
Western Sub-Saharan Africa	4.41(2.39,7.04)	4.55(2.54,7.32)	3.55(2.00,5.82)	3.63(2.08,5.73)	0.88(0.60,1.27)	1.27(0.87,1.81)	22.40(14.84,30.21)	26.84(18.22,35.30)	0.70(0.42,1.06)	0.97(0.59,1.44)
High-middle SDI	27.20(18.11,39.87)	29.48(18.97,43.15)	6.88(4.18,10.24)	7.69(4.66,11.79)	7.94(5.67,10.98)	7.96(5.57,11.14)	105.63(84.98,126.49)	98.70(78.06,119.48)	11.06(9.84,12.32)	10.09(9.05,11.21)
High SDI	51.00(39.86,65.07)	52.96(40.45,69.35)	15.83(9.75,24.14)	18.89(11.53,28.83)	61.78(50.50,78.01)	60.50(48.28,77.26)	598.57(495.58,691.67)	587.41(484.84,680.69)	34.54(31.37,38.24)	32.84(29.88,36.31)
Low-middle SDI	6.63(4.01,9.88)	6.15(3.75,9.10)	7.05(4.12,11.22)	6.56(3.88,10.40)	5.43(4.18,6.89)	5.33(4.09,6.78)	59.38(44.86,73.20)	58.69(44.52,71.94)	4.46(3.72,5.43)	4.55(3.82,5.54)
Low SDI	4.31(2.46,6.63)	4.36(2.56,6.63)	6.19(3.50,10.11)	6.06(3.54,9.54)	2.17(1.40,3.40)	2.62(1.68,4.07)	43.01(32.60,52.96)	50.81(39.15,61.76)	2.12(1.55,3.40)	2.61(1.96,4.13)
Middle SDI	24.02(15.91,34.60)	23.63(15.53,34.41)	7.58(4.49,11.83)	7.49(4.41,11.73)	9.18(7.26,11.61)	8.82(6.96,11.21)	68.08(52.82,82.78)	63.60(49.05,77.63)	10.09(8.59,11.59)	9.36(7.99,10.74)

Table S4. Regional prevalence and ASPR of the 5 drug use disorders in 2021

location_name	Amphetamine		Cannabis		Cocaine		Opioid		Other drug	
	Prevalence	ASPR	Prevalence	ASPR	Prevalence	ASPR	Prevalence	ASPR	Prevalence	ASPR
Global	116.24(84.93,153.12)	115.99(84.63,153.55)	286.89(223.70,384.41)	286.23(222.58,384.31)	51.62(40.59,64.88)	50.63(39.74,63.79)	204.84(179.10,233.57)	198.49(173.42,227.22)	19.19(15.66,23.42)	18.17(14.82,22.12)
East Asia	228.27(169.93,300.21)	269.62(195.03,362.11)	189.19(145.07,251.06)	205.90(154.31,284.04)	5.78(3.99,7.92)	6.13(4.18,8.59)	104.11(86.78,122.54)	94.72(77.62,112.73)	18.64(14.76,23.40)	15.84(12.50,20.06)
Southeast Asia	200.53(138.25,277.08)	189.63(130.40,262.39)	284.39(207.88,400.06)	269.94(194.27,381.28)	2.10(1.28,3.09)	1.99(1.22,2.92)	58.09(48.61,68.34)	54.30(45.42,63.78)	11.36(8.90,14.61)	10.24(8.04,13.11)
Oceania	145.84(97.55,204.20)	136.57(92.19,189.06)	486.16(311.64,732.35)	455.60(298.54,672.23)	2.39(1.38,3.56)	2.30(1.35,3.42)	65.89(53.58,79.84)	68.86(56.55,82.59)	10.91(8.59,13.85)	11.67(9.17,14.79)
Central Asia	131.83(94.65,176.49)	127.60(91.05,172.64)	196.15(130.76,295.14)	198.04(129.47,302.45)	22.91(16.39,30.56)	22.51(16.26,30.00)	223.26(194.19,258.20)	213.65(185.05,247.50)	16.07(12.76,20.12)	15.04(11.97,18.87)
Central Europe	151.50(110.15,200.59)	181.72(129.43,246.17)	269.51(216.07,339.86)	342.62(264.57,440.97)	33.43(24.35,44.92)	35.91(25.34,49.44)	88.94(77.73,101.34)	89.23(77.04,104.10)	18.66(14.80,23.80)	16.52(13.06,21.12)
Eastern Europe	167.42(128.75,213.50)	203.57(153.65,264.85)	268.94(194.10,366.05)	336.82(230.68,483.34)	42.71(32.79,53.40)	48.53(36.71,62.33)	430.72(380.93,487.82)	431.53(379.31,493.25)	33.05(26.83,40.42)	29.41(23.88,35.65)
High-income Asia Pacific	87.79(61.86,119.32)	110.32(76.10,153.56)	362.21(269.69,502.39)	466.09(328.88,669.92)	95.85(72.21,126.79)	101.46(72.92,139.47)	87.65(71.42,104.66)	90.22(71.08,109.56)	18.22(13.49,23.85)	16.87(12.49,22.85)
Australasia	478.93(351.11,629.69)	513.42(371.55,682.47)	637.36(534.56,762.86)	718.11(599.67,860.36)	216.79(158.95,300.78)	225.80(164.31,314.59)	290.30(265.62,317.52)	284.21(259.19,311.84)	111.73(94.65,128.15)	102.72(87.20,118.37)
Western Europe	158.14(116.84,208.39)	187.82(135.17,251.47)	469.22(391.34,574.94)	598.62(494.10,743.63)	128.91(97.01,173.29)	138.33(100.60,190.17)	234.08(213.25,257.50)	237.54(213.94,263.02)	51.26(43.27,59.72)	49.29(41.46,57.13)
Southern Latin America	74.92(51.83,102.81)	72.35(49.93,99.31)	365.88(322.40,422.14)	369.24(325.22,426.04)	260.82(198.29,344.42)	250.22(188.83,332.21)	118.42(93.40,144.94)	110.90(86.57,136.22)	19.30(14.67,25.70)	17.59(13.28,23.51)
High-income North America	301.42(227.64,386.07)	334.25(249.54,432.38)	865.30(677.13,1120.15)	973.88(752.87,1275.29)	458.04(364.74,560.10)	479.97(379.72,592.54)	1862.41(1644.13,2112.86)	1890.26(1659.84,2156.24)	80.38(67.68,95.57)	76.38(64.09,90.25)
Caribbean	52.87(37.16,72.69)	51.23(35.87,70.57)	482.31(323.57,699.84)	476.43(315.55,696.65)	109.01(79.69,150.63)	106.38(77.47,147.92)	92.28(73.90,113.11)	88.28(70.52,108.35)	15.16(12.25,19.03)	14.34(11.57,17.95)
Andean Latin America	75.45(51.85,104.77)	68.86(47.60,95.21)	252.83(184.76,351.93)	236.64(172.76,327.74)	67.79(48.48,96.72)	64.08(46.11,90.87)	106.20(82.78,130.99)	99.23(77.66,122.43)	13.47(10.38,17.39)	12.96(10.02,16.73)
Central Latin America	53.69(37.37,73.85)	50.38(35.05,69.23)	291.10(234.05,367.24)	274.83(220.77,346.77)	112.41(80.93,157.51)	106.31(76.67,148.96)	93.15(73.70,114.22)	87.65(69.37,107.42)	12.96(10.10,16.59)	12.15(9.46,15.57)
Tropical Latin America	183.03(128.12,249.22)	174.84(121.64,239.06)	430.77(323.10,574.55)	421.92(314.68,564.89)	203.38(151.73,265.42)	195.30(144.63,256.82)	98.99(77.91,122.39)	90.82(71.67,113.42)	12.36(9.64,15.82)	10.86(8.44,13.92)
North Africa and Middle East	39.34(28.07,53.46)	37.14(26.45,50.55)	140.34(98.70,196.93)	133.59(93.62,187.44)	18.04(12.80,24.20)	17.26(12.39,23.08)	233.75(199.31,272.05)	222.34(190.30,258.56)	14.61(11.31,18.69)	13.53(10.54,17.30)
South Asia	12.62(8.82,17.39)	11.62(8.13,15.85)	286.35(217.05,386.29)	260.40(198.16,346.85)	3.38(2.28,4.80)	3.18(2.19,4.42)	112.03(91.22,135.24)	105.86(87.16,126.68)	11.15(8.87,13.96)	10.92(8.67,13.69)
Central Sub-Saharan Africa	38.24(25.55,54.51)	38.70(26.71,54.28)	182.69(119.24,283.02)	180.66(124.03,266.94)	7.08(5.06,9.65)	7.95(5.88,10.39)	59.25(47.18,73.07)	69.81(56.86,83.82)	7.56(6.03,9.52)	9.69(7.75,12.09)
Eastern Sub-Saharan Africa	37.00(24.74,53.14)	36.30(25.41,51.02)	234.42(155.92,345.13)	216.64(152.35,307.84)	4.29(3.18,5.65)	5.17(4.11,6.43)	50.42(40.32,61.57)	60.32(49.34,71.65)	5.63(4.34,7.20)	7.20(5.67,9.18)
Southern Sub-Saharan Africa	122.99(86.33,166.24)	113.54(80.78,152.22)	312.63(219.69,446.05)	291.16(205.32,413.03)	90.34(67.71,118.26)	89.26(67.95,114.91)	137.24(115.30,161.53)	134.96(114.30,157.31)	13.22(10.47,16.65)	12.85(10.24,16.05)
Western Sub-Saharan Africa	32.63(21.80,47.01)	33.73(23.57,47.81)	122.13(85.87,177.35)	124.85(91.97,173.12)	5.03(3.91,6.41)	7.80(6.52,9.37)	51.70(40.56,64.73)	61.75(49.74,74.58)	6.38(4.93,8.10)	8.81(7.03,11.16)
High-middle SDI	167.36(123.86,221.10)	185.54(133.58,250.49)	236.64(190.73,303.26)	264.53(208.24,342.36)	41.30(31.57,53.50)	42.67(31.64,56.57)	169.32(146.54,194.93)	159.87(135.83,186.50)	21.47(17.19,26.86)	18.38(14.55,22.89)
High SDI	209.85(158.17,267.45)	237.37(177.48,305.30)	550.92(434.71,706.62)	655.96(511.77,852.96)	215.68(171.12,263.88)	228.90(179.18,285.81)	760.20(677.88,854.76)	761.65(674.95,864.14)	54.06(45.75,63.27)	49.09(41.37,57.69)
Low-middle SDI	44.22(30.66,61.22)	40.67(28.60,55.91)	244.54(185.57,336.80)	227.75(174.78,308.98)	15.21(11.39,20.18)	14.55(11.04,19.07)	104.87(85.96,125.81)	102.41(85.01,121.39)	11.09(8.81,13.87)	11.32(9.07,14.14)
Low SDI	30.12(20.25,43.11)	30.09(21.03,42.28)	214.30(152.44,308.08)	209.94(155.68,290.75)	6.07(4.53,8.04)	6.86(5.34,8.62)	70.34(56.39,85.84)	80.76(66.91,96.27)	6.77(5.30,8.62)	8.65(6.89,10.93)
Middle SDI	142.99(103.84,190.78)	142.22(102.85,192.42)	261.88(203.79,349.07)	258.47(198.74,350.17)	33.16(25.26,43.74)	32.48(24.64,43.22)	115.69(97.06,136.24)	108.64(90.53,128.61)	14.41(11.33,18.13)	12.82(10.13,16.20)

Table S5. Regional death and ASMR of the 5 drug use disorders in 2021

location_name	Amphetamine		Cannabis		Cocaine		Opioid		Other drug	
	Death	ASMR	Death	ASMR	Death	ASMR	Death	ASMR	Death	ASMR
Global	0.13(0.11,0.14)	0.12(0.11,0.13)	-	-	0.16(0.14,0.18)	0.15(0.14,0.17)	1.26(1.18,1.37)	1.19(1.12,1.29)	0.19(0.18,0.21)	0.18(0.17,0.20)
East Asia	0.19(0.15,0.23)	0.17(0.14,0.21)	-	-	0.01(0.01,0.02)	0.01(0.01,0.02)	0.41(0.33,0.50)	0.33(0.26,0.40)	0.21(0.16,0.26)	0.18(0.14,0.22)
Southeast Asia	0.04(0.02,0.05)	0.03(0.02,0.05)	-	-	0.02(0.02,0.03)	0.02(0.02,0.03)	0.24(0.20,0.30)	0.25(0.20,0.30)	0.04(0.02,0.05)	0.04(0.03,0.06)
Oceania	0.01(0.00,0.01)	0.01(0.01,0.02)	-	-	0.01(0.01,0.02)	0.01(0.01,0.02)	0.11(0.08,0.14)	0.13(0.09,0.17)	0.01(0.01,0.02)	0.02(0.01,0.03)
Central Asia	0.07(0.06,0.09)	0.07(0.06,0.09)	-	-	0.11(0.08,0.13)	0.10(0.07,0.12)	0.63(0.52,0.74)	0.63(0.52,0.74)	0.12(0.09,0.14)	0.12(0.10,0.14)
Central Europe	0.05(0.04,0.05)	0.05(0.04,0.05)	-	-	0.03(0.03,0.04)	0.03(0.02,0.03)	0.68(0.62,0.73)	0.56(0.52,0.61)	0.09(0.09,0.10)	0.09(0.08,0.09)
Eastern Europe	0.22(0.19,0.25)	0.22(0.20,0.25)	-	-	0.14(0.12,0.15)	0.12(0.11,0.13)	2.91(2.64,3.19)	2.55(2.33,2.81)	0.56(0.51,0.62)	0.51(0.46,0.56)
High-income Asia Pacific	0.01(0.01,0.01)	0.01(0.01,0.01)	-	-	0.02(0.02,0.02)	0.02(0.02,0.02)	0.16(0.15,0.17)	0.12(0.11,0.13)	0.03(0.03,0.04)	0.02(0.02,0.02)
Australasia	0.28(0.23,0.32)	0.27(0.23,0.31)	-	-	0.04(0.04,0.05)	0.04(0.03,0.05)	1.88(1.64,2.16)	1.74(1.51,1.99)	2.71(2.42,3.04)	2.36(2.11,2.64)
Western Europe	0.14(0.13,0.14)	0.14(0.13,0.15)	-	-	0.12(0.11,0.12)	0.11(0.11,0.12)	2.05(1.93,2.15)	1.67(1.59,1.74)	0.39(0.36,0.41)	0.36(0.34,0.38)
Southern Latin America	0.01(0.01,0.02)	0.01(0.01,0.01)	-	-	0.03(0.03,0.04)	0.03(0.02,0.03)	0.16(0.14,0.18)	0.14(0.12,0.16)	0.03(0.02,0.03)	0.02(0.02,0.03)
High-income North America	1.29(1.14,1.57)	1.15(1.03,1.39)	-	-	1.99(1.78,2.43)	1.75(1.57,2.11)	15.72(13.93,17.79)	14.50(12.92,16.30)	1.11(0.98,1.29)	1.02(0.91,1.19)
Caribbean	0.04(0.03,0.05)	0.04(0.03,0.04)	-	-	0.21(0.17,0.25)	0.20(0.16,0.24)	0.10(0.08,0.12)	0.09(0.07,0.11)	0.08(0.07,0.09)	0.07(0.06,0.09)
Andean Latin America	0.04(0.03,0.05)	0.04(0.03,0.05)	-	-	0.27(0.22,0.32)	0.26(0.21,0.32)	0.12(0.09,0.15)	0.12(0.09,0.16)	0.10(0.08,0.13)	0.10(0.08,0.13)
Central Latin America	0.04(0.03,0.05)	0.04(0.03,0.05)	-	-	0.22(0.20,0.25)	0.21(0.19,0.24)	0.09(0.08,0.11)	0.09(0.08,0.10)	0.09(0.08,0.10)	0.08(0.07,0.09)
Tropical Latin America	0.01(0.01,0.01)	0.01(0.01,0.01)	-	-	0.46(0.43,0.50)	0.42(0.40,0.46)	0.04(0.03,0.04)	0.03(0.03,0.04)	0.11(0.10,0.12)	0.11(0.10,0.12)
North Africa and Middle East	0.04(0.03,0.06)	0.04(0.03,0.06)	-	-	0.11(0.08,0.14)	0.11(0.08,0.14)	0.79(0.66,0.91)	0.81(0.68,0.93)	0.28(0.24,0.34)	0.28(0.24,0.34)
South Asia	0.02(0.01,0.02)	0.02(0.01,0.02)	-	-	0.06(0.04,0.08)	0.06(0.04,0.09)	0.44(0.36,0.51)	0.48(0.40,0.55)	0.07(0.06,0.09)	0.09(0.07,0.11)
Central Sub-Saharan Africa	0.00(0.00,0.01)	0.01(0.00,0.01)	-	-	0.01(0.00,0.04)	0.02(0.00,0.05)	0.26(0.15,0.40)	0.35(0.20,0.53)	0.00(0.00,0.01)	0.00(0.00,0.01)
Eastern Sub-Saharan Africa	0.01(0.00,0.02)	0.01(0.01,0.02)	-	-	0.02(0.01,0.05)	0.03(0.01,0.07)	0.42(0.28,0.54)	0.62(0.43,0.79)	0.01(0.00,0.01)	0.01(0.01,0.02)
Southern Sub-Saharan Africa	0.06(0.05,0.08)	0.07(0.05,0.08)	-	-	0.12(0.09,0.15)	0.14(0.11,0.17)	0.77(0.68,0.88)	0.88(0.78,0.99)	0.21(0.18,0.24)	0.23(0.20,0.27)
Western Sub-Saharan Africa	0.00(0.00,0.00)	0.00(0.00,0.00)	-	-	0.00(0.00,0.00)	0.00(0.00,0.00)	0.02(0.02,0.03)	0.05(0.04,0.07)	0.00(0.00,0.00)	0.00(0.00,0.00)
High-middle SDI	0.10(0.09,0.12)	0.09(0.08,0.11)	-	-	0.05(0.04,0.05)	0.04(0.04,0.05)	0.79(0.73,0.84)	0.67(0.62,0.71)	0.19(0.17,0.20)	0.16(0.15,0.18)
High SDI	0.51(0.46,0.60)	0.44(0.41,0.52)	-	-	0.73(0.65,0.88)	0.62(0.56,0.73)	6.19(5.59,6.90)	5.47(4.97,6.06)	0.61(0.56,0.67)	0.54(0.50,0.60)
Low-middle SDI	0.01(0.01,0.02)	0.02(0.01,0.02)	-	-	0.07(0.05,0.09)	0.07(0.06,0.10)	0.37(0.31,0.42)	0.42(0.36,0.48)	0.07(0.06,0.09)	0.09(0.07,0.11)
Low SDI	0.01(0.00,0.01)	0.01(0.01,0.01)	-	-	0.02(0.01,0.04)	0.04(0.02,0.06)	0.29(0.22,0.35)	0.43(0.34,0.51)	0.03(0.02,0.05)	0.04(0.03,0.08)
Middle SDI	0.11(0.09,0.13)	0.10(0.08,0.12)	-	-	0.10(0.09,0.11)	0.09(0.08,0.10)	0.46(0.40,0.52)	0.42(0.36,0.47)	0.18(0.15,0.21)	0.17(0.14,0.19)

Table S6. Age-standardized incidence, prevalence, mortality, and DALY rates of the 5 drug use disorders among the top 3 and bottom 3 countries in 2021.

Measure	Type	Top3			Bottom3		
ASDR	Amphetamine	New Zealand	Australia	United States of America	Bhutan	Nepal	Bangladesh
		101.43(56.46,160.67)	117.15(70.48,181.70)	145.72(112.67,193.27)	2.35(1.19,4.12)	2.38(1.22,4.07)	2.47(1.29,4.25)
	Cannabis	New Zealand	Canada	United States of America	Turkey	Togo	Syrian Arab Republic
		43.54(21.83,76.91)	40.40(24.33,62.39)	37.75(20.59,64.27)	3.26(1.41,6.06)	3.86(1.65,7.51)	4.22(1.66,8.27)
	Cocaine	United States of America	United States Virgin Islands	Puerto Rico	Palau	Timor-Leste	Indonesia
		216.76(173.33,278.73)	145.11(78.59,248.03)	144.10(103.66,194.85)	0.57(0.26,1.10)	0.80(0.36,1.57)	0.81(0.49,1.35)
	Opioid	United States of America	Canada	Republic of Estonia	Republic of Indonesia	Federal Republic of Nigeria	Republic of the Philippines
		2236.30(1814.50,2654.92)	955.07(790.86,1131.50)	856.93(648.48,1075.10)	21.31(14.84,29.76)	33.20(20.91,49.15)	34.03(22.95,48.45)
	Other drugs	Australia	United States of America	Republic of Iceland	Federal Republic of Somalia	Federal Democratic Republic of Ethiopia	Republic of the Niger
		192.93(154.41,237.86)	88.27(76.33,104.62)	80.27(59.38,106.22)	1.08(0.34,2.38)	1.17(0.61,1.99)	1.18(0.35,2.41)
ASIR	Amphetamine	Australia	New Zealand	United States of America	Nepal	Bangladesh	Bhutan
		78.25(47.28,119.26)	77.46(45.18,120.06)	60.99(38.65,88.96)	2.16(0.97,3.77)	2.21(1.02,3.84)	2.26(1.05,3.93)
	Cannabis	New Zealand	United States of America	Canada	Turkey	Syrian Arab Republic	Togo
		215.26(109.35,352.82)	165.93(90.20,266.47)	156.02(111.26,212.41)	16.99(8.65,28.46)	22.18(10.36,39.76)	22.65(11.06,39.71)
	Cocaine	United States of America	Spain	Uruguay	Indonesia	Cambodia	Lao People's Democratic Republic
		29.22(19.67,45.10)	19.80(11.01,33.67)	19.11(11.16,32.09)	0.24(0.07,0.54)	0.32(0.10,0.68)	0.32(0.10,0.69)
	Opioid	Republic of Estonia	United States of America	Republic of Lithuania	Republic of Indonesia	Republic of Kenya	Republic of the Philippines
		223.71(173.79,283.87)	212.94(166.31,277.02)	118.50(88.88,153.03)	6.23(3.65,9.55)	12.01(7.32,17.90)	12.23(7.42,18.52)
	Other drugs	Australia	Kingdom of Norway	Kingdom of Denmark	Federal Republic of Somalia	Republic of Kenya	Federal Democratic Republic of Ethiopia
		292.81(199.80,409.01)	260.08(174.52,375.66)	253.07(170.63,353.91)	59.30(37.64,90.01)	59.81(37.88,91.10)	61.13(39.20,92.45)
ASMR	Amphetamine	United States of America	Canada	Finland	-	-	-
		1.71(1.46,2.18)	0.75(0.54,1.00)	0.51(0.36,0.71)	-	-	-
	Cannabis	-	-	-	-	-	-
		-	-	-	-	-	-
	Cocaine	United States of America	Puerto Rico	United States Virgin Islands	-	-	-
		2.59(2.25,3.29)	2.25(1.57,3.08)	2.07(0.98,3.77)	-	-	-
	Opioid	United States of America	Canada	United Kingdom of Great Britain and Northern Ireland	-	-	-
		21.56(18.90,24.48)	9.70(7.91,11.65)	5.24(4.95,5.56)	-	-	-

	Other drugs	Australia	United States of America	New Zealand	-	-	-
		3.69(2.90,4.62)	1.52(1.32,1.82)	1.38(1.12,1.69)	-	-	-
ASPR	Amphetamine	Australia	New Zealand	Taiwan (Province of China)	Nepal	Bangladesh	Bhutan
		723.99(480.34,1031.88)	698.95(449.71,1024.86)	544.84(390.70,736.21)	13.72(7.45,22.18)	14.11(7.69,22.58)	14.44(7.91,23.17)
	Cannabis	New Zealand	Canada	United States of America	Turkey	Togo	Syrian Arab Republic
		1516.12(918.00,2388.78)	1394.96(1136.66,1715.17)	1321.74(867.87,1965.16)	110.98(67.64,169.97)	132.22(78.60,215.34)	144.56(78.12,245.21)
	Cocaine	United States of America	Canada	Argentina	Indonesia	Cambodia	Timor-Leste
		698.05(529.84,904.64)	410.51(304.65,545.68)	369.69(259.21,516.77)	2.07(0.85,4.02)	2.81(1.17,5.20)	2.81(1.19,5.14)
	Opioid	United States of America	Republic of Estonia	Canada	Republic of Indonesia	Republic of Kenya	Republic of the Philippines
		2825.30(2365.17,3384.14)	1457.55(1288.41,1641.74)	1082.10(956.60,1223.19)	34.36(23.66,48.11)	66.75(47.03,91.36)	68.11(47.52,94.77)
	Other drugs	Australia	Kingdom of Norway	United States of America	Federal Republic of Somalia	Republic of Kenya	Federal Democratic Republic of Ethiopia
		159.61(119.69,207.67)	118.09(84.95,161.05)	111.51(79.73,151.50)	8.09(5.14,12.16)	8.53(5.38,13.08)	8.75(5.58,13.23)

Table S7. EAPC of ASIR and ASDR for the 4 drug use disorders in 204 countries and territories from 1990 to 2021

Location	Cannabis		Cocaine		Opioid		Other drug	
	EAPC of ASDR	EAPC of ASIR	EAPC of ASDR	EAPC of ASIR	EAPC of ASDR	EAPC of ASIR	EAPC of ASDR	EAPC of ASIR
Afghanistan	0.16(0.13,0.19)	0.08(0.06,0.10)	-1.25(-1.33,-1.17)	0.22(0.20,0.24)	0.56(0.36,0.76)	0.30(0.08,0.53)	0.89(0.74,1.05)	0.27(0.18,0.36)
Albania	-0.20(-0.30,-0.11)	-0.22(-0.30,-0.13)	-0.38(-0.55,-0.22)	0.10(0.08,0.12)	1.53(1.14,1.93)	2.08(1.87,2.28)	0.29(-0.08,0.66)	0.42(0.37,0.48)
Algeria	0.13(0.06,0.20)	0.12(0.05,0.19)	0.01(-0.12,0.14)	0.20(0.19,0.22)	0.79(0.71,0.86)	0.57(0.53,0.60)	0.43(0.17,0.69)	0.23(0.20,0.26)
American Samoa	-0.02(-0.03,-0.01)	-0.02(-0.02,-0.01)	0.90(0.09,1.72)	-0.10(-0.13,-0.07)	-0.02(-0.23,0.18)	0.10(0.09,0.12)	0.42(-0.06,0.91)	0.23(0.20,0.27)
Andorra	-0.02(-0.04,-0.01)	-0.00(-0.01,0.01)	0.34(0.30,0.39)	0.37(0.34,0.39)	0.41(0.26,0.56)	0.59(0.47,0.72)	0.53(0.46,0.60)	0.31(0.27,0.34)
Angola	-0.05(-0.07,-0.04)	-0.06(-0.08,-0.05)	0.59(0.48,0.69)	0.39(0.32,0.46)	0.45(0.39,0.51)	0.31(0.28,0.34)	0.83(0.72,0.95)	0.37(0.30,0.43)
Antigua and Barbuda	-0.01(-0.08,0.06)	-0.01(-0.08,0.06)	-0.10(-1.04,0.85)	0.13(0.05,0.20)	-0.43(-0.65,-0.21)	-0.14(-0.22,-0.06)	0.45(-0.41,1.33)	0.22(0.21,0.24)
Argentina	0.22(0.15,0.29)	0.23(0.17,0.29)	0.39(0.13,0.65)	0.54(0.14,0.94)	-0.10(-0.18,-0.01)	-0.13(-0.22,-0.04)	0.56(0.40,0.71)	-0.04(-0.11,0.04)
Armenia	0.11(0.06,0.16)	0.10(0.07,0.14)	0.51(0.12,0.90)	-0.15(-0.18,-0.12)	-0.33(-0.51,-0.15)	-0.43(-0.54,-0.33)	2.37(1.47,3.28)	0.03(-0.14,0.20)
Australia	-1.20(-1.46,-0.94)	-1.12(-1.35,-0.90)	0.55(0.41,0.70)	0.37(0.25,0.50)	-0.68(-1.14,-0.22)	-0.40(-0.84,0.04)	2.99(2.26,3.74)	0.16(0.09,0.24)
Austria	0.15(0.10,0.20)	0.15(0.10,0.21)	0.35(0.23,0.48)	0.20(0.02,0.38)	0.13(-0.28,0.54)	-0.96(-1.39,-0.53)	2.55(2.46,2.64)	0.86(0.70,1.03)
Azerbaijan	0.08(0.06,0.09)	0.05(0.04,0.06)	0.47(0.37,0.57)	0.00(-0.02,0.02)	-0.22(-0.34,-0.11)	-0.42(-0.49,-0.36)	1.12(0.75,1.48)	0.12(-0.06,0.30)
Bahamas	-0.04(-0.11,0.03)	-0.05(-0.12,0.03)	-0.74(-1.32,-0.15)	0.02(-0.01,0.04)	-0.06(-0.18,0.05)	-0.02(-0.12,0.08)	0.43(-0.29,1.15)	0.38(0.31,0.44)
Bahrain	0.16(0.14,0.19)	0.13(0.11,0.16)	-0.27(-0.54,-0.01)	-0.05(-0.09,0.00)	0.26(0.20,0.32)	0.29(0.23,0.36)	0.11(-0.36,0.58)	0.04(0.01,0.07)
Bangladesh	-0.11(-0.18,-0.04)	-0.13(-0.18,-0.08)	-0.18(-0.40,0.05)	0.12(0.07,0.18)	-0.36(-0.48,-0.23)	-0.29(-0.36,-0.21)	0.52(0.39,0.65)	0.24(0.21,0.27)
Barbados	-0.06(-0.14,0.03)	-0.09(-0.17,-0.01)	-0.16(-0.90,0.58)	0.05(-0.20,0.30)	-0.53(-1.21,0.16)	0.14(-0.39,0.66)	0.90(-0.05,1.86)	0.07(0.04,0.10)
Belarus	0.05(0.04,0.06)	0.02(0.02,0.02)	0.04(-0.33,0.42)	-0.06(-0.13,0.01)	1.38(1.14,1.61)	0.70(0.37,1.02)	-0.18(-0.89,0.54)	0.20(0.12,0.28)
Belgium	-0.46(-0.56,-0.37)	-0.43(-0.51,-0.35)	0.58(0.38,0.79)	0.15(-0.04,0.35)	-0.30(-0.39,-0.21)	-0.18(-0.23,-0.13)	1.95(1.77,2.13)	0.30(0.25,0.34)
Belize	-0.30(-0.38,-0.21)	-0.32(-0.42,-0.23)	-0.03(-0.59,0.55)	0.02(-0.02,0.07)	0.12(0.05,0.18)	0.12(0.06,0.18)	0.50(-0.27,1.29)	0.14(0.12,0.16)
Benin	0.19(0.12,0.26)	0.14(0.08,0.20)	0.06(-0.09,0.22)	0.41(0.38,0.44)	-0.96(-1.47,-0.45)	-0.15(-0.27,-0.03)	0.54(0.45,0.62)	0.35(0.29,0.41)
Bermuda	-0.00(-0.01,0.01)	-0.00(-0.02,0.01)	-0.90(-2.19,0.41)	-0.16(-0.24,-0.09)	0.29(0.23,0.35)	0.15(0.09,0.20)	-0.07(-1.43,1.31)	0.32(0.27,0.38)
Bhutan	0.01(-0.00,0.02)	-0.02(-0.02,-0.01)	-0.74(-0.91,-0.57)	-0.08(-0.12,-0.05)	-0.47(-0.57,-0.37)	-0.37(-0.45,-0.30)	1.24(1.18,1.30)	0.38(0.33,0.44)
Bolivia (Plurinational State of)	0.23(0.18,0.28)	0.17(0.13,0.22)	0.47(0.28,0.67)	0.27(-0.12,0.65)	1.07(0.77,1.36)	1.13(0.84,1.42)	0.04(-0.12,0.20)	0.19(0.15,0.23)
Bosnia and Herzegovina	-0.66(-0.87,-0.44)	-0.57(-0.74,-0.40)	0.44(0.36,0.51)	0.12(0.06,0.19)	0.01(-0.09,0.11)	0.17(0.14,0.19)	0.45(0.36,0.55)	0.24(0.16,0.32)
Botswana	0.07(0.05,0.09)	0.06(0.05,0.07)	-0.73(-1.09,-0.36)	0.35(0.25,0.46)	-0.15(-0.27,-0.04)	-0.22(-0.34,-0.10)	0.48(0.29,0.68)	0.24(0.17,0.30)
Brazil	-0.35(-0.45,-0.25)	-0.24(-0.31,-0.18)	3.52(3.17,3.86)	1.90(1.66,2.14)	-1.41(-1.58,-1.25)	-0.95(-1.07,-0.84)	5.26(5.03,5.50)	0.78(0.73,0.82)
Brunei Darussalam	0.01(0.00,0.02)	0.01(0.00,0.01)	-0.68(-0.80,-0.56)	-0.33(-0.39,-0.27)	1.51(1.22,1.80)	1.22(1.12,1.33)	-1.27(-1.54,-1.00)	-0.34(-0.39,-0.29)
Bulgaria	0.26(0.14,0.39)	0.30(0.16,0.45)	-0.33(-0.60,-0.05)	0.17(-0.22,0.57)	0.28(0.24,0.32)	0.26(0.23,0.29)	0.30(0.03,0.58)	0.41(0.35,0.47)
Burkina Faso	0.02(-0.02,0.05)	-0.02(-0.05,0.01)	0.03(-0.10,0.17)	0.31(0.28,0.33)	0.07(0.00,0.14)	-0.07(-0.11,-0.03)	0.62(0.52,0.72)	0.40(0.33,0.47)
Burundi	0.06(0.04,0.08)	0.03(0.02,0.04)	-1.13(-1.33,-0.93)	0.19(0.15,0.23)	0.08(0.04,0.11)	0.13(0.07,0.19)	-0.23(-0.40,-0.07)	-0.18(-0.27,-0.08)
Cabo Verde	0.16(0.14,0.18)	0.12(0.11,0.13)	1.38(1.28,1.48)	1.59(1.50,1.67)	0.00(-0.04,0.05)	0.02(-0.01,0.05)	1.83(1.72,1.94)	1.09(1.01,1.18)
Cambodia	-0.33(-0.44,-0.22)	-0.30(-0.39,-0.20)	-0.71(-0.81,-0.61)	-0.25(-0.33,-0.18)	3.59(3.12,4.07)	1.85(1.38,2.32)	-0.46(-0.63,-0.29)	0.12(0.03,0.21)
Cameroon	0.05(0.04,0.06)	0.04(0.04,0.04)	1.00(0.71,1.29)	1.56(1.48,1.63)	-0.05(-0.08,-0.02)	-0.01(-0.05,0.02)	0.80(0.74,0.86)	0.49(0.44,0.53)
Canada	0.05(-0.17,0.28)	0.01(-0.21,0.23)	2.17(1.97,2.36)	0.05(-0.13,0.24)	-0.19(-0.24,-0.14)	-0.17(-0.19,-0.14)	3.83(3.58,4.09)	0.37(0.32,0.41)

Location	Cannabis		Cocaine		Opioid		Other drug	
	EAPC of ASDR	EAPC of ASIR	EAPC of ASDR	EAPC of ASIR	EAPC of ASDR	EAPC of ASIR	EAPC of ASDR	EAPC of ASIR
Central African Republic	0.02(-0.00,0.04)	-0.02(-0.03,-0.01)	0.07(0.02,0.13)	0.28(0.27,0.29)	0.32(0.21,0.44)	-0.03(-0.12,0.05)	0.17(0.13,0.20)	0.03(0.01,0.06)
Chad	0.03(0.02,0.04)	0.03(0.03,0.03)	0.20(0.07,0.33)	0.32(0.26,0.38)	-4.73(-5.21,-4.24)	-2.90(-3.25,-2.54)	0.55(0.44,0.65)	0.41(0.34,0.48)
Chile	1.51(1.35,1.68)	1.14(1.00,1.27)	0.44(0.04,0.84)	0.36(-0.09,0.80)	-0.17(-0.23,-0.11)	-0.06(-0.14,0.02)	-0.32(-0.54,-0.09)	0.01(-0.07,0.09)
China	0.51(0.29,0.73)	0.47(0.26,0.68)	-3.23(-3.76,-2.70)	-1.35(-1.58,-1.11)	-0.34(-0.52,-0.16)	-0.20(-0.29,-0.10)	-5.47(-6.20,-4.72)	-0.78(-0.89,-0.66)
Colombia	1.40(1.04,1.76)	1.05(0.76,1.34)	-0.23(-0.39,-0.06)	0.31(-0.32,0.95)	0.48(0.27,0.68)	0.30(0.25,0.35)	0.12(-0.11,0.34)	-0.22(-0.31,-0.14)
Comoros	0.05(0.04,0.06)	0.03(0.03,0.03)	-0.02(-0.28,0.25)	0.41(0.39,0.43)	0.28(0.23,0.33)	0.27(0.25,0.30)	0.74(0.62,0.87)	0.19(0.12,0.25)
Congo	0.01(0.00,0.02)	-0.00(-0.01,0.00)	0.31(0.17,0.46)	0.72(0.62,0.82)	-0.04(-0.08,-0.00)	-0.05(-0.10,0.01)	0.51(0.44,0.58)	0.33(0.28,0.38)
Cook Islands	-0.08(-0.09,-0.06)	-0.04(-0.06,-0.03)	1.16(0.79,1.54)	-0.09(-0.11,-0.06)	0.09(0.00,0.17)	0.10(0.02,0.18)	0.76(0.62,0.91)	0.26(0.23,0.28)
Costa Rica	0.10(0.05,0.16)	0.10(0.05,0.16)	-0.06(-0.36,0.25)	0.22(-0.42,0.87)	1.82(1.07,2.57)	1.58(1.07,2.10)	0.67(0.36,0.98)	0.17(0.13,0.20)
Croatia	-0.18(-0.26,-0.10)	-0.20(-0.29,-0.11)	-1.30(-1.48,-1.11)	0.11(0.02,0.21)	-0.81(-0.87,-0.75)	-0.54(-0.60,-0.49)	-0.51(-0.75,-0.27)	0.48(0.44,0.52)
Cuba	0.03(0.02,0.03)	0.01(0.01,0.01)	-0.90(-0.97,-0.84)	-0.04(-0.05,-0.02)	-0.15(-0.31,0.01)	0.39(0.19,0.59)	-1.54(-1.71,-1.37)	-0.06(-0.15,0.02)
Cyprus	-0.51(-0.77,-0.26)	-0.49(-0.72,-0.26)	-0.00(-0.23,0.23)	-0.10(-0.37,0.17)	2.54(2.27,2.80)	2.60(2.23,2.97)	0.28(-0.02,0.58)	0.09(0.06,0.12)
Czechia	0.12(-0.05,0.29)	-0.25(-0.37,-0.12)	-2.54(-3.10,-1.98)	-0.43(-0.95,0.09)	-0.92(-1.01,-0.82)	-0.32(-0.34,-0.30)	0.16(-0.05,0.38)	0.70(0.64,0.76)
Côte d'Ivoire	0.04(0.03,0.06)	0.02(0.02,0.02)	0.32(0.15,0.49)	0.65(0.62,0.68)	0.62(0.50,0.74)	0.44(0.39,0.49)	0.62(0.54,0.70)	0.36(0.30,0.42)
Democratic People's Republic of Korea	0.06(0.04,0.08)	0.05(0.04,0.06)	-0.60(-0.73,-0.46)	0.01(-0.01,0.04)	0.16(0.02,0.30)	0.68(0.43,0.92)	-1.70(-1.92,-1.48)	-0.19(-0.24,-0.13)
Democratic Republic of the Congo	0.07(0.06,0.09)	0.03(0.02,0.03)	0.39(0.20,0.59)	0.29(0.27,0.32)	0.51(0.37,0.65)	0.49(0.42,0.57)	0.13(-0.07,0.34)	-0.00(-0.11,0.10)
Denmark	-0.70(-0.80,-0.60)	-0.72(-0.83,-0.62)	-0.18(-0.37,0.01)	0.15(-0.03,0.33)	-0.07(-0.18,0.05)	-0.13(-0.21,-0.04)	0.52(0.33,0.72)	0.09(0.04,0.13)
Djibouti	0.04(0.02,0.06)	0.06(0.05,0.08)	0.33(0.26,0.41)	0.66(0.62,0.70)	0.36(0.25,0.47)	0.37(0.28,0.47)	0.75(0.71,0.79)	0.23(0.17,0.29)
Dominica	-0.74(-0.92,-0.55)	-0.81(-1.01,-0.60)	0.99(0.43,1.55)	0.48(0.31,0.66)	0.39(0.28,0.50)	0.10(0.01,0.19)	1.96(1.38,2.53)	0.57(0.52,0.62)
Dominican Republic	0.64(0.40,0.88)	0.58(0.36,0.81)	-0.07(-0.13,-0.02)	0.03(0.01,0.05)	0.64(0.60,0.68)	0.44(0.42,0.46)	1.32(0.88,1.76)	0.22(0.16,0.27)
Ecuador	-0.01(-0.04,0.02)	-0.02(-0.04,0.01)	2.01(1.72,2.31)	0.19(-0.07,0.44)	-0.15(-0.24,-0.05)	0.03(-0.05,0.11)	1.31(0.48,2.15)	0.27(0.22,0.32)
Egypt	0.32(0.17,0.46)	0.27(0.15,0.39)	-0.03(-0.10,0.04)	-0.14(-0.19,-0.09)	0.73(0.68,0.78)	0.49(0.42,0.55)	1.74(1.59,1.89)	0.01(-0.06,0.09)
El Salvador	-0.07(-0.28,0.15)	-0.07(-0.25,0.12)	-0.93(-1.19,-0.67)	0.22(0.17,0.26)	0.74(0.69,0.79)	-0.00(-0.04,0.04)	-0.34(-0.60,-0.09)	-0.01(-0.07,0.06)
Equatorial Guinea	0.30(0.28,0.33)	0.28(0.26,0.30)	1.22(1.14,1.30)	1.00(0.87,1.12)	3.28(2.65,3.91)	3.12(2.67,3.57)	2.07(1.86,2.28)	0.98(0.82,1.15)
Eritrea	0.12(0.11,0.14)	0.04(0.03,0.04)	0.66(0.61,0.72)	0.80(0.78,0.82)	-0.27(-0.32,-0.21)	-0.28(-0.33,-0.23)	1.05(0.97,1.13)	0.34(0.33,0.36)
Estonia	-0.25(-0.32,-0.19)	-0.27(-0.35,-0.19)	-1.38(-1.70,-1.05)	1.44(1.28,1.59)	-0.35(-0.41,-0.29)	0.07(0.04,0.10)	-0.45(-1.02,0.13)	0.63(0.59,0.67)
Eswatini	0.14(0.08,0.20)	0.16(0.10,0.22)	-0.02(-0.24,0.20)	0.16(0.14,0.18)	-0.97(-1.15,-0.79)	-0.16(-0.19,-0.14)	1.59(1.28,1.91)	0.30(0.26,0.34)
Ethiopia	0.06(0.05,0.07)	0.03(0.02,0.03)	-1.94(-2.21,-1.66)	0.27(0.25,0.29)	2.41(2.22,2.60)	2.28(2.10,2.45)	0.10(-0.11,0.31)	0.17(0.01,0.33)
Fiji	0.02(0.01,0.02)	0.00(-0.00,0.00)	-2.32(-2.92,-1.72)	-0.18(-0.23,-0.14)	1.75(1.23,2.28)	1.34(0.83,1.85)	-1.58(-1.96,-1.20)	-0.03(-0.08,0.03)
Finland	-0.03(-0.07,0.01)	-0.03(-0.06,0.01)	0.88(0.68,1.08)	0.65(0.43,0.87)	-0.07(-0.12,-0.02)	0.08(0.06,0.09)	2.72(2.49,2.95)	0.34(0.30,0.38)
France	0.03(-0.03,0.09)	-0.12(-0.19,-0.05)	0.80(0.69,0.90)	0.42(0.28,0.55)	0.25(-0.36,0.86)	-0.18(-0.60,0.24)	1.82(1.71,1.92)	0.35(0.31,0.39)
Gabon	-0.08(-0.10,-0.07)	-0.08(-0.08,-0.07)	0.41(0.33,0.50)	0.89(0.83,0.96)	-0.15(-0.30,-0.01)	-0.39(-0.61,-0.18)	0.55(0.51,0.59)	0.22(0.20,0.24)
Gambia	-0.04(-0.06,-0.03)	-0.02(-0.03,-0.02)	0.22(0.07,0.37)	0.47(0.45,0.49)	0.03(-0.03,0.09)	0.04(-0.01,0.09)	0.42(0.35,0.49)	0.32(0.26,0.37)
Georgia	0.48(0.28,0.68)	0.42(0.25,0.58)	0.77(0.16,1.38)	0.08(0.05,0.11)	1.09(0.23,1.97)	-0.19(-0.86,0.48)	1.75(1.09,2.41)	0.40(0.24,0.55)
Germany	0.55(0.46,0.63)	0.33(0.26,0.40)	-0.32(-0.56,-0.07)	-1.09(-1.50,-0.68)	1.11(0.95,1.26)	0.26(0.14,0.39)	2.36(2.14,2.59)	0.40(0.33,0.47)

Location	Cannabis		Cocaine		Opioid		Other drug	
	EAPC of ASDR	EAPC of ASIR	EAPC of ASDR	EAPC of ASIR	EAPC of ASDR	EAPC of ASIR	EAPC of ASDR	EAPC of ASIR
Ghana	0.30(0.20,0.40)	0.26(0.17,0.34)	0.72(0.57,0.86)	1.69(1.59,1.78)	-0.36(-0.60,-0.12)	-0.27(-0.35,-0.20)	0.31(0.18,0.44)	0.22(0.13,0.31)
Greece	-0.27(-0.53,-0.02)	-0.28(-0.51,-0.05)	0.79(0.73,0.84)	0.20(0.13,0.26)	-1.03(-1.22,-0.83)	-0.04(-0.08,-0.00)	3.25(2.99,3.51)	-0.03(-0.08,0.02)
Greenland	0.13(0.01,0.26)	0.00(-0.11,0.11)	-0.28(-0.42,-0.14)	-0.49(-0.60,-0.37)	-0.51(-0.76,-0.26)	-0.14(-0.17,-0.11)	0.72(0.55,0.88)	0.02(-0.11,0.14)
Grenada	-0.03(-0.12,0.06)	-0.04(-0.13,0.06)	0.81(-0.06,1.68)	1.02(0.85,1.18)	-0.15(-0.18,-0.12)	-0.13(-0.16,-0.11)	0.97(-0.06,2.01)	0.41(0.37,0.45)
Guam	-0.02(-0.04,-0.00)	0.00(-0.01,0.01)	-4.04(-4.87,-3.19)	0.01(-0.03,0.05)	-0.06(-0.11,-0.01)	-0.00(-0.02,0.02)	-1.87(-2.38,-1.35)	0.39(0.36,0.42)
Guatemala	-0.07(-0.32,0.18)	-0.05(-0.27,0.17)	0.74(-0.17,1.65)	0.85(0.74,0.95)	-0.30(-0.37,-0.23)	-0.28(-0.32,-0.23)	-0.42(-0.78,-0.06)	-0.65(-0.90,-0.40)
Guinea	-0.02(-0.04,-0.00)	-0.03(-0.03,-0.02)	0.15(-0.02,0.33)	0.39(0.36,0.42)	-0.05(-0.14,0.05)	-0.18(-0.24,-0.11)	0.55(0.47,0.62)	0.36(0.31,0.42)
Guinea-Bissau	0.02(0.00,0.03)	0.01(0.01,0.01)	0.11(-0.05,0.28)	0.46(0.43,0.49)	-0.03(-0.06,-0.01)	0.06(0.03,0.08)	0.49(0.44,0.55)	0.29(0.25,0.34)
Guyana	0.09(0.06,0.13)	0.11(0.07,0.15)	0.38(0.10,0.65)	0.06(0.03,0.09)	0.99(0.88,1.10)	0.78(0.76,0.80)	0.92(0.44,1.40)	0.05(-0.04,0.14)
Haiti	0.14(0.04,0.23)	0.11(0.02,0.20)	0.75(0.68,0.83)	0.27(0.22,0.31)	2.36(2.17,2.55)	2.07(1.84,2.31)	1.70(1.51,1.89)	0.08(0.03,0.13)
Honduras	0.01(-0.06,0.08)	0.03(-0.04,0.09)	0.05(-0.04,0.13)	0.47(0.41,0.52)	-0.02(-0.36,0.32)	0.11(-0.31,0.53)	0.30(0.11,0.50)	0.20(0.18,0.23)
Hungary	-0.36(-0.46,-0.27)	-0.37(-0.48,-0.26)	-0.43(-0.55,-0.31)	0.23(0.02,0.44)	-0.15(-0.52,0.22)	-1.23(-1.87,-0.58)	-0.55(-0.76,-0.33)	0.18(0.12,0.24)
Iceland	-0.27(-0.33,-0.21)	-0.27(-0.33,-0.22)	1.21(0.97,1.46)	0.51(0.37,0.64)	0.14(0.01,0.27)	0.12(0.04,0.21)	2.81(2.52,3.10)	0.62(0.54,0.70)
India	0.30(0.14,0.46)	0.23(0.09,0.37)	-0.31(-0.54,-0.07)	-0.29(-0.41,-0.17)	2.39(1.85,2.93)	1.16(0.68,1.65)	0.69(0.50,0.88)	0.54(0.48,0.59)
Indonesia	0.40(0.30,0.49)	0.33(0.24,0.43)	0.91(0.78,1.04)	-0.54(-0.73,-0.35)	-0.02(-0.41,0.38)	0.61(0.25,0.97)	0.94(0.76,1.12)	0.17(0.02,0.33)
Iran (Islamic Republic of)	1.37(1.13,1.61)	1.20(0.99,1.41)	-1.08(-1.33,-0.84)	0.03(-0.07,0.12)	0.16(-0.21,0.53)	0.23(0.14,0.32)	-0.49(-1.01,0.03)	0.27(0.24,0.31)
Iraq	0.28(0.19,0.37)	0.26(0.18,0.35)	-0.76(-1.01,-0.51)	-0.17(-0.21,-0.12)	-4.17(-4.51,-3.84)	-2.73(-3.12,-2.35)	1.34(0.98,1.70)	0.03(-0.04,0.11)
Ireland	-0.76(-0.86,-0.65)	-0.76(-0.87,-0.65)	0.59(0.33,0.85)	0.21(-0.04,0.46)	-0.26(-0.35,-0.17)	-0.15(-0.23,-0.07)	2.52(2.03,3.02)	0.66(0.57,0.75)
Israel	0.37(0.18,0.56)	0.33(0.15,0.50)	-0.01(-0.22,0.19)	-0.24(-0.30,-0.19)	-0.01(-0.25,0.24)	-0.23(-0.34,-0.12)	1.25(0.53,1.97)	-0.34(-0.40,-0.29)
Italy	-1.11(-1.29,-0.92)	-1.02(-1.19,-0.85)	-0.15(-0.50,0.20)	-0.07(-0.44,0.30)	0.09(-0.02,0.20)	0.15(0.07,0.24)	-0.19(-0.57,0.19)	0.12(0.07,0.16)
Jamaica	0.15(0.05,0.26)	0.17(0.06,0.28)	-0.72(-1.04,-0.40)	-0.66(-0.97,-0.34)	1.09(0.29,1.89)	0.65(0.31,1.00)	-0.28(-0.81,0.25)	0.01(-0.05,0.07)
Japan	-0.03(-0.04,-0.01)	-0.03(-0.05,-0.02)	-0.02(-0.18,0.14)	-0.07(-0.15,0.01)	0.07(-0.02,0.16)	-0.32(-0.45,-0.19)	-0.42(-0.73,-0.11)	-0.25(-0.36,-0.14)
Jordan	0.05(0.02,0.07)	0.03(0.01,0.04)	-1.09(-1.28,-0.89)	-0.18(-0.22,-0.15)	0.31(0.21,0.41)	0.20(0.16,0.25)	-2.67(-3.18,-2.15)	0.02(-0.03,0.07)
Kazakhstan	-0.00(-0.02,0.01)	-0.00(-0.01,0.00)	2.69(1.68,3.72)	0.82(0.63,1.00)	0.50(0.36,0.64)	0.32(0.23,0.40)	1.71(0.29,3.15)	0.58(0.49,0.67)
Kenya	1.35(1.09,1.60)	1.26(1.02,1.49)	0.49(0.40,0.57)	0.44(0.38,0.50)	0.72(0.49,0.95)	0.32(0.22,0.41)	0.56(0.42,0.70)	0.04(-0.10,0.19)
Kiribati	0.21(0.15,0.27)	0.15(0.10,0.20)	1.82(1.63,2.01)	-0.14(-0.20,-0.09)	0.17(-0.56,0.90)	-0.38(-0.61,-0.15)	1.26(1.13,1.39)	-0.03(-0.09,0.03)
Kuwait	0.40(0.19,0.61)	0.49(0.30,0.67)	-0.58(-1.54,0.38)	0.14(0.08,0.20)	-0.09(-0.13,-0.05)	0.06(-0.01,0.12)	0.43(-1.50,2.40)	0.27(0.21,0.33)
Kyrgyzstan	0.02(0.01,0.03)	0.01(0.01,0.01)	0.01(-0.83,0.86)	0.03(-0.04,0.10)	-0.18(-0.59,0.24)	-0.30(-0.54,-0.06)	1.29(0.33,2.26)	-0.01(-0.15,0.12)
Lao People's Democratic Republic	0.06(0.05,0.06)	0.01(0.01,0.02)	-0.83(-0.92,-0.75)	-0.27(-0.34,-0.20)	1.19(1.13,1.24)	1.26(1.21,1.31)	-0.32(-0.44,-0.19)	0.17(0.09,0.24)
Latvia	-0.28(-0.37,-0.18)	-0.21(-0.30,-0.12)	-3.64(-4.22,-3.06)	-0.02(-0.14,0.09)	0.68(0.59,0.77)	0.28(0.25,0.31)	-0.79(-1.28,-0.31)	0.12(0.04,0.20)
Lebanon	0.60(0.51,0.69)	0.54(0.46,0.63)	0.03(-0.13,0.19)	0.44(0.39,0.50)	-0.09(-0.14,-0.03)	-0.10(-0.16,-0.05)	-0.28(-0.44,-0.11)	0.24(0.21,0.27)
Lesotho	0.19(0.18,0.21)	0.17(0.16,0.18)	0.68(0.50,0.87)	0.15(0.12,0.17)	2.06(1.93,2.19)	1.26(1.19,1.32)	2.54(2.25,2.82)	0.32(0.26,0.38)
Liberia	0.35(0.23,0.47)	0.27(0.16,0.38)	0.10(-0.08,0.29)	0.33(0.29,0.36)	2.35(2.10,2.61)	2.04(1.40,2.67)	0.40(0.29,0.51)	0.26(0.17,0.35)
Libya	0.01(-0.01,0.04)	0.02(0.01,0.04)	1.44(1.25,1.64)	0.79(0.72,0.85)	-1.38(-1.83,-0.93)	-0.94(-1.35,-0.53)	2.03(1.75,2.32)	0.39(0.28,0.50)
Lithuania	-0.03(-0.14,0.08)	-0.01(-0.11,0.09)	-1.96(-2.38,-1.54)	0.44(0.32,0.57)	0.19(0.13,0.26)	0.10(0.02,0.18)	0.26(-0.15,0.67)	0.36(0.31,0.42)

Location	Cannabis		Cocaine		Opioid		Other drug	
	EAPC of ASDR	EAPC of ASIR	EAPC of ASDR	EAPC of ASIR	EAPC of ASDR	EAPC of ASIR	EAPC of ASDR	EAPC of ASIR
Luxembourg	0.04(0.02,0.06)	0.04(0.02,0.05)	-0.73(-0.95,-0.50)	0.08(0.03,0.13)	0.66(0.61,0.70)	-0.00(-0.02,0.01)	-0.23(-0.40,-0.06)	0.38(0.33,0.43)
Madagascar	0.02(0.01,0.03)	-0.02(-0.02,-0.01)	-0.34(-0.42,-0.26)	0.04(0.01,0.08)	0.01(-0.04,0.07)	0.17(0.10,0.25)	0.31(0.15,0.47)	0.04(-0.05,0.13)
Malawi	0.04(0.02,0.05)	-0.01(-0.02,-0.00)	0.18(0.07,0.29)	0.29(0.26,0.31)	0.89(0.83,0.94)	0.40(0.35,0.45)	0.74(0.65,0.83)	0.10(0.03,0.18)
Malaysia	-0.29(-0.38,-0.20)	-0.27(-0.36,-0.19)	0.22(0.11,0.32)	-0.17(-0.23,-0.10)	-0.14(-0.19,-0.10)	-0.12(-0.15,-0.09)	-0.68(-0.82,-0.53)	-0.01(-0.05,0.03)
Maldives	0.17(0.14,0.20)	0.07(0.06,0.08)	2.70(2.56,2.83)	0.03(0.01,0.06)	1.39(0.81,1.98)	1.29(0.58,2.00)	0.64(0.54,0.74)	0.11(0.05,0.16)
Mali	0.04(0.02,0.06)	0.01(0.01,0.02)	-0.12(-0.23,-0.02)	-0.01(-0.08,0.05)	-0.14(-0.22,-0.07)	0.26(0.23,0.30)	0.33(0.21,0.44)	0.25(0.17,0.33)
Malta	0.43(0.24,0.63)	0.35(0.19,0.51)	0.97(0.79,1.16)	0.16(0.04,0.29)	-0.04(-0.09,-0.00)	-0.00(-0.04,0.04)	2.94(2.75,3.13)	0.37(0.33,0.40)
Marshall Islands	0.01(-0.00,0.01)	-0.01(-0.01,-0.00)	-0.49(-0.63,-0.35)	-0.04(-0.08,-0.01)	2.54(2.34,2.74)	0.69(0.51,0.87)	-0.28(-0.36,-0.19)	0.17(0.14,0.20)
Mauritania	0.01(-0.01,0.02)	-0.01(-0.02,-0.01)	0.36(0.18,0.54)	0.60(0.57,0.64)	-0.27(-0.39,-0.14)	-0.20(-0.33,-0.08)	0.55(0.46,0.64)	0.37(0.31,0.43)
Mauritius	0.57(0.40,0.74)	0.56(0.40,0.73)	9.77(7.44,12.14)	-0.09(-0.15,-0.04)	0.71(0.43,0.99)	0.01(-0.08,0.10)	1.65(-0.51,3.85)	1.41(1.27,1.54)
Mexico	-0.02(-0.09,0.05)	0.09(0.04,0.14)	-0.26(-0.55,0.03)	1.20(0.69,1.71)	0.31(0.23,0.39)	0.34(0.26,0.42)	0.32(-0.13,0.78)	-0.06(-0.10,-0.03)
Micronesia (Federated States of)	0.02(0.02,0.03)	0.00(-0.00,0.00)	-0.75(-0.95,-0.55)	-0.13(-0.16,-0.10)	0.45(0.38,0.51)	0.13(0.11,0.16)	-0.76(-0.88,-0.63)	-0.01(-0.03,0.01)
Monaco	-0.01(-0.02,-0.00)	-0.01(-0.02,-0.00)	0.37(0.35,0.40)	0.24(0.21,0.26)	1.44(1.39,1.49)	0.67(0.64,0.71)	2.24(1.93,2.55)	0.53(0.48,0.58)
Mongolia	0.60(0.43,0.78)	0.57(0.41,0.74)	1.60(1.19,2.02)	0.04(0.03,0.05)	-0.55(-0.60,-0.50)	-0.37(-0.41,-0.33)	2.57(2.02,3.13)	0.22(0.08,0.36)
Montenegro	-0.47(-0.60,-0.34)	-0.46(-0.58,-0.33)	-0.05(-0.10,-0.00)	-0.00(-0.02,0.01)	0.04(0.01,0.06)	0.06(0.02,0.10)	0.36(0.24,0.49)	0.11(0.05,0.17)
Morocco	0.19(0.11,0.27)	0.19(0.11,0.27)	0.29(0.11,0.47)	0.16(0.15,0.16)	-0.03(-0.07,0.01)	-0.23(-0.29,-0.17)	0.97(0.70,1.24)	0.34(0.32,0.37)
Mozambique	0.06(0.05,0.07)	0.02(0.01,0.03)	1.26(1.10,1.42)	1.32(1.24,1.41)	0.76(0.27,1.25)	-0.13(-0.73,0.47)	1.65(1.57,1.73)	0.76(0.72,0.79)
Myanmar	-0.02(-0.07,0.02)	-0.04(-0.08,0.00)	-0.87(-0.95,-0.80)	-0.29(-0.35,-0.24)	0.53(0.29,0.78)	0.18(0.06,0.30)	-0.68(-0.81,-0.54)	0.26(0.17,0.34)
Namibia	0.59(0.42,0.77)	0.59(0.42,0.76)	-0.41(-0.46,-0.37)	0.04(0.02,0.06)	-0.47(-0.53,-0.41)	-0.39(-0.43,-0.35)	0.56(0.47,0.64)	0.20(0.12,0.27)
Nauru	0.01(-0.00,0.02)	0.01(0.01,0.01)	-1.04(-1.21,-0.87)	-0.10(-0.12,-0.08)	0.09(0.01,0.18)	0.09(-0.00,0.18)	-1.12(-1.41,-0.83)	-0.10(-0.24,0.04)
Nepal	-0.03(-0.04,-0.02)	-0.04(-0.05,-0.04)	-0.19(-0.54,0.16)	-0.22(-0.29,-0.16)	-0.07(-0.13,-0.01)	-0.08(-0.12,-0.04)	1.13(0.92,1.34)	0.28(0.18,0.37)
Netherlands	-0.89(-0.97,-0.80)	-0.88(-1.01,-0.76)	0.45(0.24,0.66)	-0.13(-0.39,0.12)	-0.75(-0.88,-0.61)	-0.08(-0.16,0.01)	2.05(1.91,2.19)	0.19(0.15,0.23)
New Zealand	0.20(0.07,0.33)	0.07(-0.04,0.18)	0.66(0.56,0.77)	0.40(0.31,0.50)	0.09(-0.88,1.06)	0.74(0.17,1.31)	3.06(2.67,3.44)	0.39(0.38,0.40)
Nicaragua	-0.42(-0.57,-0.27)	-0.38(-0.51,-0.26)	-0.52(-0.74,-0.29)	0.03(-0.06,0.12)	-0.00(-0.11,0.10)	0.00(-0.09,0.10)	-0.11(-0.35,0.14)	-0.20(-0.27,-0.13)
Niger	0.02(0.01,0.04)	0.01(0.01,0.01)	-0.42(-0.53,-0.31)	-0.22(-0.29,-0.15)	-0.00(-0.48,0.48)	-0.07(-0.66,0.53)	0.13(0.01,0.26)	0.10(0.01,0.19)
Nigeria	-0.01(-0.10,0.09)	0.02(-0.08,0.11)	0.85(0.75,0.95)	0.75(0.66,0.84)	-0.24(-0.33,-0.14)	-0.19(-0.28,-0.11)	0.91(0.80,1.03)	0.61(0.53,0.69)
Niue	-0.00(-0.02,0.01)	0.02(0.00,0.04)	-0.94(-1.10,-0.77)	-0.09(-0.11,-0.06)	0.02(-0.05,0.09)	0.05(-0.00,0.11)	-0.51(-0.61,-0.41)	0.23(0.20,0.26)
North Macedonia	-0.20(-0.25,-0.15)	-0.20(-0.24,-0.16)	-0.41(-0.52,-0.30)	0.08(0.05,0.10)	-0.09(-0.12,-0.05)	0.21(0.19,0.23)	0.36(0.05,0.67)	0.25(0.18,0.33)
Northern Mariana Islands	0.11(0.06,0.16)	0.08(0.04,0.11)	-2.78(-3.56,-1.98)	0.00(-0.03,0.03)	-0.26(-0.32,-0.20)	-0.28(-0.33,-0.23)	-1.70(-2.16,-1.23)	0.02(-0.00,0.05)
Norway	-0.25(-0.30,-0.21)	-0.20(-0.24,-0.16)	2.41(1.49,3.34)	0.90(0.76,1.04)	0.46(0.33,0.59)	0.34(0.26,0.43)	4.83(3.60,6.08)	0.46(0.40,0.52)
Oman	0.02(-0.02,0.07)	-0.00(-0.03,0.03)	-0.49(-0.78,-0.20)	0.02(-0.01,0.04)	-0.15(-0.25,-0.05)	-0.16(-0.27,-0.05)	-0.42(-1.31,0.47)	0.12(0.09,0.15)
Pakistan	-0.03(-0.03,-0.02)	-0.02(-0.02,-0.02)	0.30(0.07,0.53)	-0.08(-0.15,-0.01)	-0.42(-0.50,-0.35)	-0.46(-0.53,-0.39)	1.37(1.16,1.58)	0.34(0.28,0.40)
Palau	0.07(0.05,0.10)	0.04(0.02,0.05)	0.03(0.01,0.05)	0.01(-0.02,0.04)	-1.00(-1.93,-0.07)	-1.55(-2.51,-0.58)	0.38(0.35,0.41)	0.14(0.12,0.16)
Palestine	0.25(0.10,0.40)	0.24(0.09,0.39)	-0.10(-0.16,-0.05)	-0.10(-0.14,-0.05)	-0.83(-1.15,-0.51)	0.08(-0.04,0.20)	-0.10(-0.19,-0.02)	0.02(-0.06,0.09)
Panama	-0.51(-0.74,-0.29)	-0.52(-0.75,-0.29)	-0.27(-0.60,0.06)	-0.19(-0.30,-0.09)	0.32(0.19,0.46)	0.30(0.19,0.41)	0.33(0.07,0.59)	-0.03(-0.06,0.00)

Location	Cannabis		Cocaine		Opioid		Other drug	
	EAPC of ASDR	EAPC of ASIR	EAPC of ASDR	EAPC of ASIR	EAPC of ASDR	EAPC of ASIR	EAPC of ASDR	EAPC of ASIR
Papua New Guinea	0.02(0.02,0.03)	0.01(0.01,0.01)	-0.91(-1.06,-0.76)	-0.18(-0.23,-0.13)	-2.95(-3.98,-1.92)	-2.09(-2.46,-1.72)	-0.41(-0.55,-0.27)	-0.02(-0.08,0.04)
Paraguay	0.10(0.06,0.15)	0.11(0.06,0.16)	2.68(2.46,2.89)	0.07(-0.09,0.24)	-0.09(-0.20,0.03)	-0.02(-0.12,0.08)	2.90(2.75,3.06)	0.08(0.00,0.15)
Peru	-0.05(-0.09,-0.01)	-0.03(-0.07,0.00)	0.62(0.24,1.01)	0.01(-0.23,0.24)	-0.15(-0.17,-0.12)	-0.09(-0.12,-0.06)	-1.38(-1.72,-1.05)	0.02(-0.02,0.06)
Philippines	0.03(0.03,0.04)	0.01(0.01,0.02)	-0.10(-0.24,0.03)	-0.64(-0.71,-0.56)	-0.06(-0.11,-0.00)	-0.04(-0.10,0.02)	-0.63(-0.77,-0.49)	-0.61(-0.73,-0.50)
Poland	-0.33(-0.41,-0.24)	-0.31(-0.39,-0.22)	-0.85(-0.99,-0.72)	0.19(0.05,0.33)	-0.14(-0.31,0.03)	0.28(0.17,0.39)	-0.15(-0.58,0.29)	0.23(0.17,0.28)
Portugal	-0.69(-0.81,-0.56)	-0.64(-0.76,-0.51)	-0.20(-0.46,0.06)	0.03(-0.13,0.19)	-0.23(-0.50,0.05)	-0.49(-0.64,-0.34)	0.14(-0.16,0.45)	-0.11(-0.19,-0.04)
Puerto Rico	0.03(0.02,0.04)	0.01(0.01,0.02)	-0.35(-2.42,1.77)	-1.29(-1.89,-0.69)	-0.23(-0.29,-0.17)	0.24(0.21,0.26)	0.63(-1.14,2.44)	0.01(-0.06,0.08)
Qatar	0.19(0.11,0.27)	0.11(0.05,0.17)	-0.29(-0.64,0.05)	0.14(0.08,0.20)	-0.41(-0.46,-0.37)	0.07(0.06,0.08)	-0.90(-1.79,0.01)	0.18(0.15,0.21)
Republic of Korea	0.02(0.01,0.02)	0.01(0.01,0.01)	-0.38(-0.49,-0.26)	-0.21(-0.27,-0.15)	0.08(0.05,0.11)	0.12(0.08,0.15)	-1.53(-1.99,-1.07)	-0.05(-0.12,0.02)
Republic of Moldova	-0.38(-0.50,-0.26)	-0.35(-0.45,-0.24)	-2.43(-2.79,-2.07)	-0.22(-0.28,-0.15)	0.28(0.13,0.43)	0.41(0.29,0.52)	-0.24(-0.56,0.09)	-0.24(-0.39,-0.09)
Romania	0.02(-0.04,0.08)	0.01(-0.05,0.07)	-1.57(-1.89,-1.25)	0.19(0.09,0.29)	-0.30(-0.32,-0.28)	-0.22(-0.24,-0.19)	-1.10(-1.40,-0.81)	0.18(0.13,0.23)
Russian Federation	0.08(-0.02,0.18)	0.06(-0.03,0.16)	-2.55(-3.16,-1.94)	0.01(-0.26,0.28)	-0.53(-0.78,-0.28)	-0.16(-0.52,0.20)	0.31(-0.48,1.11)	0.20(0.10,0.30)
Rwanda	0.80(0.60,0.99)	0.77(0.56,0.99)	-1.68(-2.03,-1.34)	0.21(0.15,0.27)	-0.10(-1.09,0.90)	-1.01(-1.77,-0.24)	-0.28(-0.55,-0.02)	0.04(-0.08,0.16)
Saint Kitts and Nevis	0.01(0.01,0.02)	0.00(0.00,0.01)	-0.88(-1.41,-0.36)	-0.08(-0.18,0.01)	-0.11(-0.24,0.02)	0.16(0.12,0.19)	-0.35(-1.01,0.31)	0.32(0.29,0.35)
Saint Lucia	-0.11(-0.32,0.09)	-0.14(-0.36,0.08)	-0.21(-0.94,0.53)	0.02(-0.01,0.05)	-0.21(-0.31,-0.10)	-0.06(-0.12,0.01)	0.52(-0.51,1.56)	0.34(0.32,0.37)
Saint Vincent and the Grenadines	0.15(0.07,0.24)	0.15(0.07,0.23)	-0.32(-0.94,0.30)	0.30(0.19,0.41)	-0.27(-0.38,-0.16)	-0.14(-0.22,-0.07)	0.01(-0.68,0.71)	0.23(0.21,0.25)
Samoa	0.44(0.25,0.62)	0.50(0.30,0.70)	-0.40(-0.64,-0.17)	-0.19(-0.23,-0.15)	-0.23(-0.35,-0.10)	-0.18(-0.25,-0.11)	-0.59(-0.77,-0.42)	-0.04(-0.07,-0.01)
San Marino	0.01(-0.00,0.03)	0.02(0.02,0.03)	0.28(0.26,0.30)	0.27(0.25,0.29)	-0.29(-0.37,-0.22)	0.13(0.10,0.16)	0.94(0.79,1.10)	0.44(0.36,0.52)
Sao Tome and Principe	0.06(0.05,0.08)	0.04(0.03,0.04)	1.18(1.09,1.26)	0.34(0.30,0.38)	0.36(0.30,0.43)	0.09(0.03,0.15)	0.52(0.39,0.66)	0.31(0.23,0.40)
Saudi Arabia	0.01(-0.02,0.04)	0.04(0.02,0.06)	0.70(0.64,0.77)	0.43(0.39,0.48)	0.28(0.17,0.39)	0.12(0.04,0.19)	1.05(0.96,1.13)	0.36(0.32,0.40)
Senegal	0.05(0.04,0.07)	0.05(0.05,0.06)	0.20(0.03,0.38)	0.38(0.35,0.40)	-0.35(-0.37,-0.33)	-0.32(-0.36,-0.28)	0.51(0.42,0.60)	0.32(0.25,0.38)
Serbia	-0.63(-0.81,-0.45)	-0.48(-0.62,-0.34)	-0.49(-0.62,-0.35)	0.15(0.11,0.19)	1.27(0.79,1.75)	1.28(0.98,1.59)	-0.20(-0.34,-0.06)	0.29(0.21,0.36)
Seychelles	-0.18(-0.43,0.08)	-0.25(-0.52,0.02)	0.83(0.24,1.43)	-0.22(-0.29,-0.16)	0.41(0.11,0.72)	0.13(0.07,0.20)	0.19(-0.23,0.60)	-0.03(-0.08,0.03)
Sierra Leone	0.08(0.06,0.11)	0.06(0.04,0.07)	0.29(0.12,0.45)	0.51(0.47,0.54)	-0.36(-0.39,-0.33)	-0.31(-0.35,-0.27)	0.39(0.28,0.51)	0.27(0.18,0.36)
Singapore	0.01(-0.00,0.02)	-0.01(-0.01,-0.00)	-0.06(-0.15,0.02)	-0.04(-0.08,0.00)	-0.28(-0.47,-0.09)	0.02(-0.07,0.11)	-0.95(-1.24,-0.67)	-0.07(-0.14,0.01)
Slovakia	-0.15(-0.21,-0.10)	-0.21(-0.30,-0.11)	-0.69(-0.86,-0.53)	-0.17(-0.39,0.06)	-0.36(-0.71,-0.02)	-0.64(-1.01,-0.27)	-0.45(-0.57,-0.34)	-0.26(-0.29,-0.24)
Slovenia	-0.63(-0.75,-0.50)	-0.59(-0.74,-0.45)	-0.89(-1.04,-0.74)	0.40(0.27,0.52)	3.07(2.38,3.76)	3.45(2.84,4.07)	-0.45(-0.83,-0.07)	0.66(0.61,0.71)
Solomon Islands	0.14(0.10,0.19)	0.13(0.09,0.17)	-0.56(-0.74,-0.38)	-0.22(-0.29,-0.16)	1.17(0.94,1.40)	1.34(1.02,1.66)	-0.53(-0.73,-0.33)	-0.10(-0.19,-0.02)
Somalia	0.02(0.00,0.04)	-0.01(-0.02,-0.00)	-0.44(-0.58,-0.29)	0.03(-0.04,0.10)	-0.14(-0.24,-0.05)	0.18(0.12,0.23)	-0.21(-0.36,-0.07)	-0.21(-0.34,-0.08)
South Africa	-0.01(-0.17,0.15)	-0.03(-0.19,0.13)	0.26(-0.05,0.59)	0.49(0.27,0.71)	0.03(-0.02,0.07)	-0.06(-0.10,-0.03)	-0.30(-0.89,0.30)	0.26(0.23,0.29)
South Sudan	-0.10(-0.12,-0.09)	-0.06(-0.08,-0.05)	0.07(-0.13,0.26)	-0.15(-0.21,-0.09)	-1.64(-2.12,-1.15)	-1.84(-2.28,-1.41)	0.24(0.17,0.32)	-0.00(-0.03,0.03)
Spain	0.65(0.56,0.74)	0.38(0.32,0.44)	-1.51(-2.72,-0.30)	-1.22(-2.32,-0.11)	0.04(-0.03,0.11)	-0.05(-0.09,-0.01)	-0.28(-0.53,-0.03)	-0.02(-0.06,0.02)
Sri Lanka	-0.01(-0.02,-0.00)	-0.00(-0.01,0.00)	-2.96(-3.64,-2.28)	-0.15(-0.20,-0.10)	-2.61(-3.09,-2.12)	-1.29(-1.75,-0.83)	-2.80(-3.44,-2.15)	-0.18(-0.29,-0.07)
Sudan	0.04(0.02,0.06)	0.04(0.03,0.05)	-0.03(-0.13,0.08)	0.10(0.09,0.12)	-2.37(-2.71,-2.03)	-1.07(-1.20,-0.93)	1.63(1.47,1.79)	0.29(0.25,0.33)
Suriname	-0.10(-0.14,-0.06)	-0.08(-0.12,-0.04)	0.79(0.02,1.56)	0.26(0.17,0.35)	0.79(0.74,0.84)	0.52(0.49,0.55)	1.71(0.69,2.73)	0.47(0.42,0.52)

Location	Cannabis		Cocaine		Opioid		Other drug	
	EAPC of ASDR	EAPC of ASIR	EAPC of ASDR	EAPC of ASIR	EAPC of ASDR	EAPC of ASIR	EAPC of ASDR	EAPC of ASIR
Sweden	-0.43(-0.62,-0.23)	-0.36(-0.53,-0.18)	3.23(2.93,3.54)	0.98(0.84,1.12)	-0.04(-0.18,0.10)	0.05(-0.01,0.11)	5.45(4.89,6.02)	0.74(0.69,0.79)
Switzerland	-0.61(-0.74,-0.48)	-0.68(-0.82,-0.54)	-0.60(-0.74,-0.47)	-0.18(-0.30,-0.06)	5.09(4.69,5.49)	5.10(4.86,5.33)	0.45(0.25,0.65)	0.06(-0.00,0.12)
Syrian Arab Republic	0.12(0.04,0.21)	0.16(0.07,0.24)	0.42(0.18,0.65)	0.21(0.19,0.23)	-3.78(-4.05,-3.51)	-4.30(-4.76,-3.84)	1.08(0.85,1.32)	0.13(0.08,0.18)
Taiwan (Province of China)	-0.00(-0.01,0.01)	0.01(0.01,0.01)	-7.85(-9.25,-6.43)	0.06(0.04,0.08)	0.73(0.63,0.84)	0.42(0.39,0.45)	0.72(0.45,1.00)	0.33(0.28,0.38)
Tajikistan	0.04(0.03,0.05)	0.03(0.02,0.03)	0.31(0.27,0.34)	-0.13(-0.15,-0.10)	-1.07(-1.68,-0.46)	0.06(-0.39,0.52)	-0.66(-0.95,-0.36)	-0.12(-0.31,0.08)
Thailand	0.12(0.08,0.16)	0.10(0.07,0.13)	0.59(0.46,0.71)	0.21(-0.05,0.48)	-0.28(-0.38,-0.19)	-0.41(-0.50,-0.32)	0.27(0.07,0.46)	0.18(0.10,0.26)
Timor-Leste	0.01(-0.00,0.03)	-0.03(-0.04,-0.02)	-0.23(-0.43,-0.03)	-0.28(-0.36,-0.21)	0.15(-0.16,0.45)	0.04(-0.24,0.32)	0.27(0.06,0.48)	0.28(0.21,0.35)
Togo	-0.05(-0.12,0.01)	-0.05(-0.10,0.00)	0.24(0.03,0.45)	0.59(0.55,0.64)	1.39(0.94,1.85)	1.31(0.93,1.70)	0.53(0.45,0.61)	0.30(0.24,0.35)
Tokelau	0.05(0.02,0.07)	0.04(0.01,0.06)	-0.48(-0.71,-0.24)	-0.03(-0.07,0.00)	0.01(-0.05,0.06)	0.03(-0.05,0.10)	-0.32(-0.49,-0.15)	0.18(0.15,0.21)
Tonga	0.10(0.04,0.17)	0.11(0.04,0.17)	-0.03(-0.13,0.07)	-0.12(-0.17,-0.07)	0.36(0.29,0.43)	0.35(0.30,0.40)	-0.11(-0.19,-0.02)	0.15(0.12,0.18)
Trinidad and Tobago	0.10(0.07,0.14)	0.10(0.07,0.12)	1.29(0.56,2.02)	0.53(0.46,0.59)	-0.33(-0.39,-0.27)	0.10(0.10,0.11)	1.85(1.03,2.67)	0.42(0.39,0.46)
Tunisia	-0.03(-0.04,-0.01)	-0.00(-0.01,0.00)	0.37(0.13,0.61)	0.11(0.09,0.12)	-0.05(-0.07,-0.04)	0.24(0.21,0.26)	1.17(0.85,1.50)	0.34(0.31,0.36)
Turkey	0.07(0.06,0.08)	0.04(0.03,0.05)	0.33(0.28,0.38)	0.35(0.19,0.51)	-0.12(-0.25,0.02)	-0.14(-0.21,-0.08)	-0.11(-0.22,-0.00)	0.22(0.18,0.26)
Turkmenistan	0.11(0.10,0.12)	0.04(0.03,0.05)	5.80(5.16,6.45)	0.26(0.25,0.27)	0.79(0.70,0.89)	0.66(0.60,0.73)	6.12(5.37,6.87)	0.32(0.15,0.48)
Tuvalu	-0.61(-0.78,-0.43)	-0.54(-0.70,-0.39)	-0.38(-0.57,-0.19)	-0.06(-0.10,-0.02)	0.11(0.05,0.16)	-0.05(-0.13,0.02)	-0.37(-0.51,-0.24)	0.12(0.09,0.15)
Uganda	-0.17(-0.26,-0.07)	-0.21(-0.31,-0.11)	0.42(0.19,0.65)	0.30(0.28,0.32)	1.68(1.38,1.98)	0.58(0.53,0.63)	1.00(0.90,1.10)	0.19(0.11,0.28)
Ukraine	-0.26(-0.33,-0.20)	-0.27(-0.33,-0.21)	-0.62(-1.00,-0.24)	0.31(0.29,0.33)	-0.16(-0.21,-0.10)	0.18(0.16,0.19)	-0.54(-1.20,0.12)	0.05(-0.06,0.16)
United Arab Emirates	0.01(-0.09,0.11)	-0.00(-0.07,0.07)	0.46(0.25,0.68)	0.39(0.36,0.42)	0.53(0.47,0.59)	-0.02(-0.07,0.03)	0.57(0.14,1.00)	0.06(-0.00,0.11)
United Kingdom	-0.12(-0.18,-0.06)	-0.26(-0.34,-0.18)	3.17(2.53,3.82)	3.27(2.33,4.23)	0.03(-0.34,0.40)	0.95(0.81,1.08)	3.10(2.69,3.51)	0.68(0.62,0.74)
United Republic of Tanzania	-0.36(-0.55,-0.17)	-0.38(-0.55,-0.21)	0.44(0.36,0.52)	0.77(0.73,0.82)	1.67(1.55,1.79)	1.40(1.28,1.52)	1.11(1.01,1.21)	0.43(0.36,0.50)
United States of America	-0.07(-0.10,-0.04)	-0.04(-0.08,0.00)	2.84(2.64,3.04)	-0.08(-0.31,0.14)	2.67(2.07,3.28)	0.74(-0.02,1.51)	5.29(4.96,5.61)	0.89(0.81,0.96)
United States Virgin Islands	0.02(0.00,0.05)	0.03(0.01,0.04)	1.98(1.70,2.25)	0.18(0.11,0.26)	0.38(0.33,0.43)	0.01(-0.07,0.10)	2.24(2.05,2.44)	0.55(0.47,0.63)
Uruguay	0.04(-0.01,0.09)	0.08(0.01,0.14)	0.29(0.02,0.57)	0.39(0.01,0.77)	7.33(7.08,7.58)	6.05(5.44,6.67)	0.56(0.32,0.81)	0.02(-0.07,0.11)
Uzbekistan	0.02(0.01,0.03)	0.02(0.02,0.02)	1.37(1.14,1.60)	0.11(0.08,0.13)	-0.36(-0.56,-0.15)	-0.57(-0.66,-0.48)	2.06(1.74,2.39)	0.14(-0.01,0.30)
Vanuatu	-0.03(-0.05,-0.02)	-0.05(-0.07,-0.04)	-0.60(-0.77,-0.43)	-0.19(-0.24,-0.14)	-0.13(-0.20,-0.06)	-0.36(-0.44,-0.28)	-0.45(-0.60,-0.30)	0.01(-0.04,0.06)
Venezuela (Bolivarian Republic of)	-0.11(-0.17,-0.04)	-0.07(-0.12,-0.02)	-0.10(-0.48,0.28)	0.20(0.02,0.38)	0.19(0.00,0.37)	-0.20(-0.29,-0.10)	1.37(1.17,1.58)	0.04(-0.02,0.10)
Viet Nam	0.05(0.04,0.06)	0.02(0.02,0.02)	1.10(0.98,1.22)	0.05(0.01,0.08)	-0.19(-0.24,-0.15)	0.19(0.18,0.20)	1.18(1.10,1.27)	0.51(0.47,0.56)
Yemen	-0.01(-0.03,0.01)	-0.03(-0.04,-0.03)	-0.36(-0.49,-0.22)	0.04(0.03,0.06)	0.80(0.67,0.93)	0.50(0.46,0.54)	2.01(1.66,2.36)	0.18(0.13,0.22)
Zambia	-0.59(-0.78,-0.41)	-0.51(-0.67,-0.35)	-0.02(-0.14,0.09)	0.77(0.73,0.82)	0.45(0.39,0.50)	-0.19(-0.22,-0.16)	1.13(0.98,1.27)	0.35(0.27,0.43)
Zimbabwe	-0.02(-0.02,-0.01)	-0.01(-0.01,-0.00)	0.39(0.27,0.51)	-0.21(-0.25,-0.17)	-0.11(-0.18,-0.03)	-0.21(-0.24,-0.18)	0.43(0.13,0.72)	-0.08(-0.14,-0.02)

Table S8. EAPC of ASMR and ASPR for the 4 drug use disorders in 204 countries and territories from 1990 to 2021

Location	Cannabis		Cocaine		Opioid		Other drug	
	EAPC of ASMR	EAPC of ASPR	EAPC of ASMR	EAPC of ASPR	EAPC of ASMR	EAPC of ASPR	EAPC of ASMR	EAPC of ASPR
Afghanistan	-	0.13(0.10,0.16)	-1.75(-1.88,-1.61)	0.30(0.28,0.31)	0.96(0.80,1.12)	0.29(0.04,0.54)	1.07(0.88,1.25)	0.41(0.27,0.56)
Albania	-	-0.21(-0.30,-0.13)	-0.78(-1.12,-0.45)	0.09(0.06,0.11)	0.88(0.28,1.48)	2.11(1.89,2.33)	0.38(-0.03,0.79)	0.70(0.59,0.80)
Algeria	-	0.13(0.05,0.20)	-0.19(-0.35,-0.04)	0.27(0.26,0.28)	1.02(0.87,1.16)	0.64(0.59,0.68)	0.50(0.22,0.77)	0.35(0.30,0.41)
American Samoa	-	-0.01(-0.02,-0.01)	1.22(0.02,2.43)	-0.13(-0.16,-0.10)	-0.13(-1.75,1.51)	0.09(0.07,0.11)	0.52(-0.96,2.01)	0.32(0.26,0.38)
Andorra	-	-0.02(-0.03,-0.01)	0.38(0.21,0.55)	0.36(0.32,0.40)	-0.05(-0.26,0.16)	0.45(0.30,0.61)	0.18(-0.05,0.42)	0.58(0.51,0.65)
Angola	-	-0.08(-0.10,-0.07)	0.66(0.46,0.87)	0.45(0.40,0.50)	0.57(0.41,0.73)	0.32(0.28,0.35)	2.26(1.98,2.54)	0.63(0.53,0.72)
Antigua and Barbuda	-	-0.01(-0.08,0.06)	0.35(-1.88,2.64)	0.07(-0.06,0.19)	-2.04(-3.72,-0.33)	-0.18(-0.27,-0.09)	1.12(-0.68,2.95)	0.40(0.36,0.44)
Argentina	-	0.22(0.15,0.29)	5.37(4.55,6.19)	0.33(0.07,0.59)	3.92(3.41,4.44)	-0.30(-0.38,-0.22)	2.61(1.87,3.35)	-0.11(-0.23,0.01)
Armenia	-	0.11(0.06,0.16)	4.27(2.19,6.40)	-0.20(-0.24,-0.16)	2.81(1.46,4.18)	-0.51(-0.63,-0.39)	4.27(2.96,5.60)	0.04(-0.22,0.30)
Australia	-	-1.21(-1.47,-0.95)	7.16(5.62,8.72)	0.35(0.21,0.49)	-1.17(-2.06,-0.27)	0.11(-0.23,0.44)	3.72(2.91,4.53)	0.98(0.81,1.14)
Austria	-	0.14(0.09,0.20)	1.62(1.21,2.03)	0.24(0.10,0.38)	0.62(-0.08,1.33)	0.12(-0.20,0.44)	2.86(2.63,3.08)	2.11(1.81,2.40)
Azerbaijan	-	0.06(0.05,0.07)	3.43(2.76,4.10)	0.03(-0.00,0.06)	1.34(0.91,1.77)	-0.49(-0.57,-0.41)	3.22(2.40,4.04)	0.18(-0.10,0.47)
Bahrain	-	-0.04(-0.10,0.03)	-2.07(-3.65,-0.47)	-0.03(-0.06,0.01)	-0.39(-0.97,0.19)	-0.03(-0.14,0.07)	0.38(-0.77,1.55)	0.63(0.53,0.74)
Bangladesh	-	0.18(0.16,0.19)	-0.92(-1.56,-0.27)	-0.09(-0.14,-0.04)	-0.02(-0.11,0.07)	0.34(0.26,0.42)	-0.15(-1.03,0.73)	0.06(0.00,0.12)
Barbados	-	-0.13(-0.19,-0.07)	-0.25(-0.49,-0.01)	0.15(0.08,0.21)	1.25(-0.36,2.89)	-0.37(-0.46,-0.28)	1.36(1.17,1.55)	0.12(0.04,0.20)
Belarus	-	-0.05(-0.13,0.04)	0.33(-1.62,2.32)	-0.03(-0.24,0.17)	-0.83(-1.59,-0.06)	0.18(-0.42,0.77)	2.31(0.54,4.10)	0.07(0.04,0.10)
Belgium	-	0.03(0.03,0.03)	0.46(-0.30,1.21)	-0.02(-0.13,0.08)	1.67(1.16,2.18)	1.53(1.20,1.86)	0.00(-0.86,0.88)	0.16(0.02,0.29)
Belize	-	-0.47(-0.56,-0.37)	1.66(1.19,2.14)	0.36(0.19,0.52)	-1.53(-3.35,0.33)	-0.22(-0.28,-0.15)	2.68(2.43,2.92)	0.59(0.55,0.63)
Benin	-	-0.29(-0.38,-0.21)	1.46(-1.14,4.13)	-0.04(-0.11,0.03)	-1.18(-1.86,-0.50)	0.13(0.05,0.21)	1.29(-0.42,3.03)	0.21(0.17,0.25)
Bermuda	-	0.18(0.11,0.24)	1.74(0.21,3.30)	0.29(0.24,0.34)	-2.96(-4.56,-1.34)	-0.25(-0.40,-0.10)	4.37(3.34,5.42)	0.53(0.46,0.60)
Bhutan	-	-0.01(-0.02,0.00)	-1.04(-3.03,1.00)	-0.25(-0.40,-0.11)	0.56(0.52,0.60)	0.16(0.09,0.22)	-0.12(-1.92,1.71)	0.50(0.42,0.58)
Bolivarian Republic of Venezuela	-	-0.01(-0.02,-0.00)	-0.93(-1.20,-0.65)	-0.07(-0.11,-0.02)	-0.39(-0.89,0.11)	-0.49(-0.57,-0.40)	2.26(2.17,2.34)	0.52(0.44,0.60)
Bosnia and Herzegovina	-	0.22(0.17,0.27)	0.72(0.69,0.75)	0.39(0.07,0.70)	0.36(0.19,0.52)	1.19(0.86,1.52)	0.23(0.06,0.40)	0.23(0.16,0.29)
Botswana	-	-0.69(-0.89,-0.48)	1.39(1.20,1.58)	0.14(0.08,0.20)	-0.44(-0.73,-0.14)	0.14(0.11,0.17)	1.03(0.80,1.27)	0.35(0.21,0.48)
Brazil	-	0.07(0.05,0.08)	-1.71(-2.32,-1.10)	0.37(0.26,0.48)	5.07(4.29,5.85)	-0.27(-0.41,-0.13)	0.55(0.27,0.83)	0.37(0.28,0.45)
Brunei Darussalam	-	-0.36(-0.46,-0.26)	7.49(6.53,8.47)	1.79(1.50,2.09)	-1.47(-1.78,-1.16)	-1.35(-1.45,-1.24)	7.22(6.89,7.54)	1.05(0.98,1.12)
Bulgaria	-	0.01(0.00,0.01)	-0.82(-1.21,-0.43)	-0.65(-0.74,-0.55)	2.14(1.54,2.74)	1.22(1.12,1.33)	-1.35(-1.72,-0.98)	-0.67(-0.75,-0.60)
Burkina Faso	-	0.26(0.13,0.38)	-3.30(-3.95,-2.64)	0.17(-0.19,0.54)	-0.87(-1.56,-0.18)	0.29(0.24,0.33)	0.17(-0.20,0.54)	0.68(0.57,0.80)
Burundi	-	-0.01(-0.04,0.02)	1.08(-0.44,2.62)	0.30(0.26,0.34)	0.04(-0.09,0.18)	-0.11(-0.15,-0.06)	5.11(3.96,6.27)	0.59(0.50,0.69)
Cambodia	-	0.06(0.05,0.07)	-2.11(-2.43,-1.78)	0.20(0.18,0.23)	-0.45(-0.60,-0.29)	0.13(0.06,0.19)	-0.13(-0.44,0.18)	-0.30(-0.43,-0.17)

Location	Cannabis		Cocaine		Opioid		Other drug	
	EAPC of ASMR	EAPC of ASPR	EAPC of ASMR	EAPC of ASPR	EAPC of ASMR	EAPC of ASPR	EAPC of ASMR	EAPC of ASPR
Cameroon	-	0.16(0.15,0.17)	3.23(3.08,3.39)	1.69(1.59,1.79)	-0.96(-1.66,-0.25)	-0.00(-0.04,0.04)	3.92(3.63,4.21)	1.83(1.72,1.94)
Canada	-	-0.35(-0.46,-0.24)	-0.87(-1.13,-0.62)	-0.25(-0.34,-0.17)	6.15(5.69,6.61)	1.89(1.53,2.26)	-0.91(-1.14,-0.69)	0.13(-0.01,0.27)
Central African Republic	-	0.04(0.04,0.04)	1.42(-0.08,2.95)	1.63(1.50,1.76)	-0.13(-0.21,-0.05)	-0.06(-0.10,-0.02)	3.99(3.02,4.97)	0.78(0.73,0.83)
Chad	-	0.06(-0.17,0.28)	6.61(6.09,7.14)	0.09(-0.07,0.24)	0.00(-0.60,0.61)	-0.22(-0.25,-0.19)	5.02(4.68,5.35)	0.80(0.74,0.86)
Chile	-	-0.02(-0.03,-0.00)	-0.27(-0.40,-0.14)	0.26(0.25,0.27)	5.12(4.27,5.97)	-0.22(-0.31,-0.13)	0.81(0.71,0.91)	0.02(-0.01,0.06)
China	-	0.04(0.04,0.04)	1.83(0.38,3.30)	0.30(0.25,0.34)	-7.11(-7.94,-6.28)	-3.31(-3.70,-2.92)	5.51(4.42,6.61)	0.57(0.47,0.67)
Colombia	-	1.52(1.35,1.68)	3.12(2.73,3.51)	0.34(-0.07,0.75)	-2.37(-3.03,-1.71)	-0.07(-0.16,0.03)	-0.76(-1.19,-0.32)	-0.05(-0.19,0.09)
Commonwealth of the Bahamas	-	0.50(0.27,0.72)	-5.34(-6.30,-4.36)	-1.46(-1.71,-1.21)	-1.03(-2.37,0.33)	-0.26(-0.37,-0.15)	-6.10(-6.97,-5.23)	-1.40(-1.58,-1.23)
Comoros	-	1.39(1.02,1.75)	-1.41(-2.24,-0.57)	0.14(-0.32,0.61)	0.36(0.05,0.67)	0.33(0.27,0.40)	0.32(0.04,0.60)	-0.37(-0.51,-0.23)
Congo	-	0.03(0.03,0.03)	-0.23(-0.64,0.18)	0.36(0.34,0.37)	0.26(0.13,0.39)	0.26(0.24,0.29)	1.59(1.33,1.85)	0.26(0.19,0.33)
Cook Islands	-	-0.00(-0.01,0.00)	0.11(-0.13,0.35)	0.75(0.66,0.84)	0.14(-0.46,0.74)	-0.07(-0.14,-0.01)	0.50(0.19,0.81)	0.49(0.43,0.56)
Costa Rica	-	-0.07(-0.09,-0.06)	1.79(1.19,2.39)	-0.11(-0.14,-0.09)	-0.19(-0.78,0.40)	0.10(0.01,0.20)	1.46(0.82,2.09)	0.45(0.43,0.48)
Croatia	-	0.11(0.05,0.16)	-0.25(-1.00,0.52)	-0.12(-0.55,0.31)	1.76(0.93,2.59)	1.95(1.24,2.65)	0.77(0.25,1.31)	0.27(0.21,0.33)
Cuba	-	-0.19(-0.27,-0.12)	-4.76(-5.32,-4.19)	0.16(0.05,0.27)	-5.77(-6.25,-5.28)	-0.67(-0.73,-0.60)	-1.24(-1.57,-0.91)	0.97(0.92,1.02)
Cyprus	-	0.02(0.02,0.02)	-4.56(-4.99,-4.13)	-0.08(-0.11,-0.05)	-0.69(-0.85,-0.54)	0.27(0.04,0.51)	-3.20(-3.51,-2.88)	-0.14(-0.28,0.00)
Czech Republic	-	-0.51(-0.77,-0.26)	0.44(0.17,0.71)	-0.07(-0.30,0.15)	1.97(1.65,2.30)	2.91(2.46,3.36)	0.34(-0.03,0.70)	0.16(0.12,0.19)
Democratic People's Republic of Korea	-	0.12(-0.05,0.29)	-4.21(-5.00,-3.41)	-0.43(-0.97,0.11)	-1.81(-2.06,-1.56)	-0.46(-0.48,-0.44)	-0.57(-0.95,-0.20)	1.25(1.16,1.35)
Democratic Republic of the Congo	-	0.01(0.01,0.01)	1.06(-0.34,2.48)	0.63(0.57,0.68)	0.68(0.39,0.96)	0.46(0.41,0.52)	3.69(2.88,4.50)	0.58(0.51,0.66)
Denmark	-	0.06(0.06,0.07)	-1.60(-1.97,-1.24)	-0.02(-0.04,0.01)	0.02(-0.18,0.23)	0.78(0.58,0.98)	-1.88(-2.10,-1.66)	-0.44(-0.55,-0.33)
Djibouti	-	0.04(0.04,0.05)	0.53(0.11,0.96)	0.31(0.27,0.35)	0.38(0.22,0.55)	0.55(0.46,0.64)	0.88(0.29,1.48)	-0.03(-0.18,0.13)
Dominica	-	-0.70(-0.80,-0.61)	-0.70(-1.15,-0.25)	0.19(0.05,0.33)	2.03(1.03,3.04)	-0.19(-0.28,-0.10)	0.82(0.59,1.04)	0.14(0.09,0.18)
Dominican Republic	-	0.04(0.02,0.05)	0.28(0.17,0.39)	0.54(0.50,0.58)	-1.64(-2.34,-0.94)	0.41(0.29,0.52)	1.53(1.40,1.66)	0.33(0.25,0.41)
Ecuador	-	-0.73(-0.91,-0.54)	1.99(0.70,3.30)	0.58(0.32,0.84)	4.07(3.58,4.57)	0.09(-0.01,0.19)	2.88(1.74,4.04)	0.89(0.82,0.96)
Egypt	-	0.63(0.39,0.87)	-1.06(-1.42,-0.69)	0.07(0.04,0.09)	3.73(3.21,4.25)	0.52(0.49,0.54)	2.14(1.28,3.00)	0.34(0.25,0.43)
El Salvador	-	-0.02(-0.05,0.01)	3.37(2.90,3.84)	0.34(0.17,0.52)	-1.93(-2.27,-1.60)	0.03(-0.06,0.13)	1.99(0.97,3.01)	0.39(0.31,0.46)
Equatorial Guinea	-	0.31(0.17,0.44)	3.87(3.27,4.48)	-0.18(-0.24,-0.12)	0.86(0.74,0.98)	0.54(0.46,0.62)	4.65(4.17,5.12)	-0.01(-0.13,0.11)
Eritrea	-	-0.10(-0.31,0.11)	-1.46(-1.83,-1.09)	0.32(0.29,0.35)	1.06(1.00,1.12)	-0.01(-0.07,0.04)	-0.40(-0.71,-0.09)	-0.03(-0.13,0.06)
Estonia	-	0.27(0.24,0.29)	1.46(1.30,1.62)	1.09(0.99,1.19)	1.84(0.76,2.93)	3.91(3.58,4.23)	4.17(4.00,4.34)	1.63(1.35,1.92)
Ethiopia	-	0.06(0.06,0.07)	0.52(0.43,0.61)	0.77(0.75,0.79)	-0.20(-0.31,-0.09)	-0.31(-0.37,-0.26)	2.17(1.91,2.43)	0.51(0.48,0.54)
Federated States of Micronesia	-	-0.27(-0.33,-0.20)	-5.70(-6.51,-4.89)	1.81(1.62,1.99)	-2.08(-2.35,-1.82)	0.01(-0.02,0.04)	-0.77(-1.36,-0.17)	1.68(1.60,1.76)
Fiji	-	0.16(0.10,0.23)	-0.41(-0.94,0.12)	0.21(0.17,0.25)	-5.43(-6.69,-4.15)	-0.21(-0.25,-0.17)	2.15(1.65,2.64)	0.50(0.46,0.54)

Location	Cannabis		Cocaine		Opioid		Other drug	
	EAPC of ASMR	EAPC of ASPR	EAPC of ASMR	EAPC of ASPR	EAPC of ASMR	EAPC of ASPR	EAPC of ASMR	EAPC of ASPR
Finland	-	0.03(0.02,0.04)	-3.95(-4.36,-3.54)	0.38(0.36,0.41)	2.08(1.77,2.38)	2.62(2.51,2.74)	0.13(-0.14,0.41)	0.22(0.02,0.42)
France	-	0.00(-0.00,0.01)	-3.23(-4.20,-2.25)	-0.20(-0.25,-0.15)	2.65(2.39,2.92)	1.89(1.15,2.63)	-4.27(-5.35,-3.18)	-0.07(-0.14,0.01)
Gabon	-	-0.03(-0.07,0.01)	0.85(0.48,1.21)	0.66(0.48,0.84)	-0.20(-0.31,-0.09)	0.03(0.01,0.05)	3.05(2.80,3.31)	0.88(0.82,0.95)
Georgia	-	0.03(-0.02,0.08)	1.62(1.42,1.82)	0.51(0.35,0.68)	4.17(2.75,5.60)	-0.23(-0.71,0.26)	2.18(2.06,2.30)	0.66(0.63,0.69)
Germany	-	-0.08(-0.09,-0.07)	0.24(0.08,0.41)	0.96(0.88,1.04)	-0.24(-0.50,0.02)	0.63(0.48,0.78)	1.30(1.13,1.46)	0.39(0.33,0.44)
Ghana	-	-0.04(-0.04,-0.03)	1.70(0.38,3.04)	0.49(0.44,0.54)	-0.83(-1.36,-0.29)	0.03(-0.04,0.10)	5.30(4.30,6.31)	0.43(0.36,0.49)
Greece	-	0.48(0.29,0.68)	1.95(0.58,3.35)	0.17(0.14,0.20)	1.92(0.72,3.12)	0.82(0.28,1.37)	2.77(1.82,3.73)	0.57(0.33,0.82)
Greenland	-	0.55(0.46,0.64)	1.91(1.62,2.21)	-0.68(-0.92,-0.44)	2.12(1.73,2.52)	0.55(0.49,0.60)	3.19(2.92,3.46)	1.30(1.19,1.41)
Grenada	-	0.29(0.20,0.38)	-2.70(-3.19,-2.20)	2.04(1.93,2.15)	-0.00(-1.49,1.51)	-0.33(-0.42,-0.25)	-9.72(-11.58,-7.81)	0.30(0.17,0.42)
Guam	-	-0.28(-0.55,-0.02)	3.47(3.09,3.85)	0.33(0.31,0.36)	-5.11(-6.51,-3.68)	-0.08(-0.13,-0.02)	5.36(5.07,5.66)	-0.04(-0.14,0.06)
Guatemala	-	0.13(0.01,0.25)	1.18(0.98,1.38)	-0.67(-0.82,-0.51)	-1.51(-2.44,-0.57)	-0.21(-0.25,-0.17)	0.98(0.72,1.24)	-0.09(-0.24,0.06)
Guinea	-	-0.03(-0.12,0.06)	0.49(-1.43,2.45)	1.42(1.21,1.64)	-0.41(-1.09,0.28)	-0.17(-0.20,-0.14)	1.32(-0.06,2.73)	0.67(0.62,0.73)
Guinea-Bissau	-	-0.02(-0.04,0.00)	-4.50(-5.56,-3.42)	-0.01(-0.05,0.03)	-1.03(-1.73,-0.32)	-0.02(-0.04,0.01)	-4.82(-5.99,-3.64)	0.80(0.71,0.88)
Guyana	-	-0.09(-0.34,0.17)	0.18(-0.85,1.21)	1.12(0.98,1.27)	1.75(0.17,3.36)	-0.32(-0.38,-0.27)	-0.35(-0.92,0.23)	-1.73(-2.26,-1.19)
Haiti	-	-0.02(-0.03,-0.02)	1.96(0.44,3.51)	0.37(0.31,0.43)	1.04(0.71,1.36)	-0.20(-0.28,-0.13)	5.54(4.51,6.58)	0.55(0.48,0.62)
Honduras	-	0.01(0.00,0.01)	1.33(-0.08,2.76)	0.35(0.30,0.39)	-0.34(-0.41,-0.26)	0.10(0.08,0.13)	4.53(3.42,5.66)	0.46(0.41,0.52)
Hungary	-	0.08(0.05,0.11)	4.17(1.97,6.41)	-0.05(-0.10,0.00)	1.58(1.18,1.99)	0.77(0.74,0.80)	1.70(0.80,2.60)	0.05(-0.08,0.19)
Iceland	-	0.11(0.01,0.21)	1.52(1.31,1.73)	0.36(0.34,0.37)	2.71(2.50,2.92)	2.17(1.98,2.36)	2.41(2.06,2.76)	0.09(0.01,0.17)
India	-	0.01(-0.05,0.08)	0.17(0.07,0.27)	0.72(0.66,0.78)	-0.35(-0.59,-0.11)	0.11(-0.32,0.54)	0.81(0.61,1.01)	0.35(0.33,0.38)
Indonesia	-	-0.38(-0.48,-0.28)	-4.63(-5.36,-3.90)	0.31(0.11,0.51)	2.54(2.30,2.77)	-1.30(-1.97,-0.63)	-1.11(-1.44,-0.77)	0.28(0.18,0.39)
Iraq	-	-0.28(-0.34,-0.22)	2.08(1.56,2.61)	0.72(0.58,0.85)	0.17(-0.01,0.34)	0.10(-0.01,0.21)	2.97(2.58,3.35)	1.66(1.49,1.83)
Ireland	-	0.28(0.12,0.44)	-0.11(-0.36,0.14)	-0.30(-0.42,-0.18)	2.78(2.00,3.56)	2.22(1.84,2.61)	0.99(0.75,1.23)	0.71(0.62,0.79)
Islamic Republic of Iran	-	0.38(0.28,0.47)	2.26(2.03,2.50)	-0.55(-0.74,-0.35)	-1.12(-1.43,-0.80)	0.64(0.16,1.12)	2.34(2.14,2.54)	0.19(0.00,0.38)
Israel	-	1.37(1.13,1.60)	-1.65(-1.97,-1.32)	-0.16(-0.25,-0.07)	0.33(-0.34,1.01)	0.12(-0.04,0.28)	-0.64(-1.18,-0.10)	0.38(0.34,0.43)
Italy	-	0.28(0.19,0.37)	-0.82(-1.08,-0.55)	-0.19(-0.24,-0.14)	-5.32(-5.85,-4.79)	-3.02(-3.52,-2.51)	1.47(1.11,1.82)	0.02(-0.10,0.15)
Jamaica	-	-0.76(-0.86,-0.65)	2.01(1.27,2.76)	0.25(0.08,0.42)	-1.97(-2.71,-1.23)	-0.19(-0.27,-0.10)	2.77(2.26,3.28)	1.46(1.32,1.60)
Japan	-	0.38(0.19,0.56)	0.99(0.20,1.80)	-0.37(-0.44,-0.30)	2.50(1.36,3.65)	-0.34(-0.47,-0.20)	1.63(0.85,2.42)	-0.51(-0.59,-0.44)
Jordan	-	-1.12(-1.30,-0.93)	-1.65(-1.91,-1.39)	0.11(-0.26,0.49)	-1.10(-1.45,-0.76)	0.17(0.06,0.27)	-0.24(-0.81,0.34)	0.19(0.14,0.24)
Kazakhstan	-	0.15(0.05,0.25)	-2.10(-3.36,-0.82)	-0.53(-0.75,-0.30)	1.57(0.25,2.89)	0.93(0.52,1.34)	-0.34(-1.15,0.47)	0.01(-0.08,0.10)
Kenya	-	-0.03(-0.04,-0.02)	2.09(1.01,3.18)	-0.13(-0.25,-0.02)	-0.20(-0.51,0.11)	-0.36(-0.51,-0.22)	-0.17(-1.03,0.68)	-0.46(-0.65,-0.27)
Kingdom of Eswatini	-	0.03(0.01,0.05)	-3.74(-4.42,-3.06)	-0.20(-0.25,-0.16)	0.76(0.49,1.02)	0.18(0.13,0.24)	-4.32(-5.18,-3.44)	0.01(-0.09,0.10)

Location	Cannabis		Cocaine		Opioid		Other drug	
	EAPC of ASMR	EAPC of ASPR	EAPC of ASMR	EAPC of ASPR	EAPC of ASMR	EAPC of ASPR	EAPC of ASMR	EAPC of ASPR
Kiribati	-	-0.01(-0.01,-0.00)	3.37(2.17,4.57)	1.16(0.93,1.39)	0.55(0.39,0.72)	0.31(0.22,0.39)	1.82(0.35,3.32)	0.91(0.75,1.06)
Kuwait	-	1.34(1.09,1.59)	1.13(1.02,1.24)	0.36(0.31,0.40)	2.75(1.35,4.16)	0.41(0.30,0.53)	1.51(1.14,1.88)	0.05(-0.13,0.23)
Kyrgyzstan	-	0.18(0.12,0.23)	1.78(1.58,1.98)	-0.13(-0.18,-0.08)	1.74(0.25,3.25)	-0.49(-0.79,-0.19)	1.22(1.09,1.35)	-0.08(-0.16,0.01)
Lao People's Democratic Republic	-	0.39(0.18,0.61)	-0.65(-2.14,0.86)	0.12(0.04,0.19)	-0.83(-0.97,-0.70)	0.04(-0.03,0.11)	0.69(-1.45,2.88)	0.44(0.35,0.54)
Latvia	-	0.00(0.00,0.01)	-0.03(-1.13,1.07)	0.04(-0.08,0.15)	0.07(-0.69,0.84)	-0.34(-0.60,-0.08)	1.61(0.56,2.66)	-0.04(-0.25,0.17)
Lebanon	-	0.03(0.03,0.04)	-1.10(-1.29,-0.91)	-0.27(-0.34,-0.19)	-0.47(-0.57,-0.37)	1.41(1.36,1.46)	-0.94(-1.09,-0.79)	0.18(0.07,0.30)
Lesotho	-	-0.30(-0.39,-0.20)	-5.88(-6.85,-4.91)	-0.02(-0.16,0.11)	2.10(1.76,2.43)	0.28(0.25,0.32)	-0.91(-1.50,-0.33)	0.31(0.18,0.45)
Liberia	-	0.60(0.51,0.69)	-0.33(-0.50,-0.15)	0.46(0.40,0.52)	-0.88(-1.59,-0.16)	-0.12(-0.19,-0.05)	-0.62(-0.82,-0.43)	0.40(0.34,0.46)
Libya	-	0.22(0.21,0.23)	1.73(1.11,2.35)	0.21(0.18,0.24)	2.76(2.42,3.09)	1.59(1.51,1.67)	3.81(3.30,4.31)	0.48(0.40,0.56)
Lithuania	-	0.32(0.19,0.44)	2.37(0.71,4.06)	0.22(0.16,0.28)	2.11(1.47,2.74)	2.32(1.56,3.09)	4.40(3.24,5.57)	0.39(0.28,0.51)
Luxembourg	-	-0.00(-0.02,0.02)	1.54(1.30,1.78)	1.08(0.98,1.18)	-1.16(-1.61,-0.71)	-1.13(-1.57,-0.68)	2.11(1.81,2.42)	0.65(0.43,0.87)
Madagascar	-	-0.04(-0.16,0.07)	-4.60(-5.30,-3.89)	0.85(0.76,0.94)	-0.07(-0.22,0.08)	0.10(0.00,0.19)	0.12(-0.34,0.58)	1.00(0.90,1.10)
Malawi	-	0.04(0.03,0.06)	-1.63(-2.07,-1.18)	0.16(0.11,0.21)	0.76(0.68,0.83)	-0.02(-0.03,-0.00)	-0.21(-0.39,-0.03)	0.85(0.77,0.93)
Malaysia	-	-0.01(-0.02,-0.01)	-0.52(-0.64,-0.40)	0.03(0.01,0.06)	-0.69(-0.85,-0.54)	0.17(0.09,0.26)	0.55(0.21,0.89)	0.03(-0.08,0.15)
Maldives	-	-0.02(-0.03,-0.01)	0.02(-0.15,0.20)	0.31(0.29,0.32)	1.65(1.51,1.79)	0.42(0.36,0.47)	1.99(1.85,2.13)	0.12(0.02,0.22)
Mali	-	-0.28(-0.38,-0.19)	0.37(0.21,0.53)	-0.16(-0.23,-0.09)	-0.63(-1.29,0.04)	-0.15(-0.18,-0.11)	-1.80(-2.27,-1.33)	-0.20(-0.28,-0.12)
Malta	-	0.15(0.12,0.18)	2.78(2.64,2.92)	0.08(0.06,0.11)	1.87(1.40,2.35)	1.24(0.52,1.96)	1.44(1.23,1.65)	0.08(0.01,0.15)
Marshall Islands	-	0.02(0.01,0.02)	1.13(-0.62,2.91)	-0.05(-0.10,-0.00)	-1.73(-1.94,-1.52)	0.26(0.22,0.30)	4.70(3.47,5.93)	0.31(0.20,0.42)
Mauritania	-	0.44(0.25,0.64)	2.37(1.87,2.88)	0.32(0.25,0.38)	-0.58(-1.29,0.13)	-0.02(-0.07,0.03)	3.73(3.49,3.96)	0.91(0.89,0.93)
Mauritius	-	0.01(0.01,0.01)	-0.39(-0.60,-0.18)	-0.06(-0.10,-0.02)	10.64(8.11,13.22)	0.78(0.54,1.02)	-1.02(-1.25,-0.78)	0.27(0.25,0.28)
Mexico	-	-0.01(-0.02,-0.01)	0.18(-1.30,1.68)	0.73(0.66,0.80)	-0.56(-0.87,-0.24)	-0.23(-0.37,-0.09)	3.04(1.97,4.11)	0.54(0.46,0.63)
Mongolia	-	0.57(0.41,0.74)	14.56(10.45,18.83)	-0.02(-0.09,0.05)	3.92(2.92,4.92)	-0.02(-0.12,0.09)	1.45(-1.47,4.46)	2.95(2.60,3.30)
Montenegro	-	-0.02(-0.09,0.05)	-0.52(-1.21,0.16)	-0.09(-0.20,0.02)	0.48(0.23,0.72)	0.29(0.21,0.38)	0.58(-0.06,1.22)	-0.10(-0.15,-0.05)
Morocco	-	0.02(0.02,0.02)	-0.80(-1.11,-0.49)	-0.15(-0.18,-0.12)	1.06(0.89,1.23)	0.16(0.13,0.19)	-1.70(-2.01,-1.40)	-0.08(-0.09,-0.06)
Mozambique	-	-0.01(-0.01,0.00)	4.91(3.97,5.86)	0.34(0.32,0.36)	2.26(2.18,2.34)	0.81(0.77,0.84)	4.88(3.92,5.85)	1.15(1.07,1.23)
Myanmar	-	0.61(0.43,0.78)	4.15(3.15,5.17)	0.06(0.04,0.08)	-1.15(-1.32,-0.99)	-0.46(-0.51,-0.41)	4.42(3.38,5.47)	0.36(0.14,0.58)
Namibia	-	-0.47(-0.60,-0.34)	-0.44(-0.86,-0.01)	-0.00(-0.02,0.01)	0.03(-0.05,0.12)	0.03(-0.03,0.08)	0.63(0.43,0.83)	0.11(0.02,0.21)
Nepal	-	0.19(0.11,0.27)	0.23(0.02,0.44)	0.20(0.19,0.21)	0.53(0.40,0.66)	-0.26(-0.33,-0.18)	1.20(0.93,1.47)	0.53(0.49,0.58)
Netherlands	-	0.04(0.03,0.05)	1.33(1.10,1.55)	1.41(1.30,1.52)	2.30(1.99,2.61)	-0.07(-0.69,0.54)	3.91(3.66,4.17)	1.26(1.20,1.32)
New Zealand	-	-0.04(-0.08,-0.00)	-1.03(-1.19,-0.87)	-0.30(-0.36,-0.23)	1.45(0.79,2.13)	0.20(0.07,0.32)	-1.67(-1.89,-1.45)	0.35(0.24,0.47)
Nicaragua	-	0.59(0.42,0.76)	-1.61(-1.78,-1.44)	0.05(0.02,0.07)	-1.01(-1.41,-0.60)	-0.47(-0.52,-0.42)	0.72(0.57,0.88)	0.31(0.21,0.41)

Location	Cannabis		Cocaine		Opioid		Other drug	
	EAPC of ASMR	EAPC of ASPR	EAPC of ASMR	EAPC of ASPR	EAPC of ASMR	EAPC of ASPR	EAPC of ASMR	EAPC of ASPR
Niger	-	-0.00(-0.00,0.00)	-1.37(-1.63,-1.11)	-0.13(-0.16,-0.11)	-0.93(-1.54,-0.32)	0.11(-0.00,0.22)	-2.19(-2.56,-1.82)	-0.22(-0.45,0.01)
Nigeria	-	-0.06(-0.07,-0.05)	-0.20(-0.73,0.33)	-0.25(-0.33,-0.18)	-0.42(-1.06,0.22)	-0.08(-0.13,-0.02)	2.05(1.76,2.34)	0.32(0.18,0.47)
Northern Mariana Islands	-	-0.88(-0.97,-0.80)	2.01(1.51,2.51)	0.32(0.06,0.59)	-3.78(-5.06,-2.49)	-0.12(-0.22,-0.02)	3.41(3.14,3.67)	0.36(0.31,0.41)
Norway	-	0.19(0.06,0.33)	8.60(7.10,10.13)	0.42(0.32,0.51)	0.55(-0.70,1.81)	0.07(-0.66,0.81)	3.60(3.17,4.04)	1.10(1.04,1.16)
Oman	-	-0.46(-0.60,-0.31)	-0.99(-1.42,-0.56)	-0.03(-0.12,0.06)	-0.13(-0.36,0.11)	0.00(-0.12,0.13)	0.12(-0.29,0.54)	-0.34(-0.45,-0.22)
Pakistan	-	-0.00(-0.01,-0.00)	0.05(-1.57,1.70)	-0.33(-0.39,-0.28)	0.15(0.02,0.28)	-0.11(-0.75,0.53)	4.30(3.22,5.40)	0.11(-0.01,0.24)
Palestine	-	-0.04(-0.13,0.06)	1.61(0.19,3.05)	1.20(1.11,1.30)	0.19(-0.09,0.47)	-0.23(-0.33,-0.13)	4.40(3.30,5.51)	0.90(0.79,1.01)
Panama	-	-0.00(-0.02,0.01)	-1.12(-1.35,-0.89)	-0.10(-0.13,-0.07)	-0.19(-0.67,0.29)	0.05(-0.02,0.11)	-1.71(-1.96,-1.45)	0.37(0.33,0.41)
Papua New Guinea	-	-0.22(-0.27,-0.18)	-2.34(-2.84,-1.83)	0.07(0.04,0.10)	-1.46(-1.80,-1.12)	0.21(0.18,0.24)	0.42(-0.04,0.87)	0.40(0.27,0.53)
Paraguay	-	0.11(0.05,0.16)	-3.20(-4.58,-1.79)	-0.06(-0.09,-0.03)	5.88(5.32,6.45)	-0.36(-0.42,-0.30)	-3.54(-5.19,-1.87)	-0.02(-0.08,0.03)
Peru	-	-0.26(-0.31,-0.21)	5.62(3.18,8.13)	1.19(1.04,1.34)	1.14(0.62,1.67)	0.37(0.27,0.46)	6.49(4.80,8.20)	1.58(1.48,1.68)
Philippines	-	0.02(-0.03,0.06)	-1.40(-2.00,-0.80)	-0.01(-0.03,0.02)	-0.00(-0.16,0.16)	-0.19(-0.31,-0.07)	-0.78(-2.13,0.58)	0.18(0.12,0.23)
Plurinational State of Bolivia	-	-0.03(-0.03,-0.02)	0.38(0.14,0.62)	-0.10(-0.17,-0.03)	0.73(0.69,0.78)	-0.57(-0.64,-0.49)	1.78(1.48,2.07)	0.37(0.28,0.47)
Poland	-	0.08(0.05,0.10)	0.04(-0.24,0.32)	0.00(-0.03,0.04)	-0.52(-1.38,0.34)	-1.63(-2.61,-0.65)	1.25(1.02,1.49)	0.26(0.24,0.28)
Portugal	-	0.24(0.09,0.39)	0.37(-0.18,0.92)	-0.11(-0.16,-0.06)	-1.23(-1.92,-0.54)	-0.03(-0.17,0.11)	-0.36(-0.97,0.25)	-0.02(-0.14,0.10)
Principality of Monaco	-	-0.51(-0.74,-0.29)	-0.30(-0.86,0.26)	-0.32(-0.48,-0.16)	4.39(3.47,5.32)	0.13(0.01,0.25)	0.56(0.13,0.98)	-0.09(-0.16,-0.03)
Puerto Rico	-	0.01(0.01,0.01)	-1.32(-1.69,-0.95)	-0.23(-0.28,-0.17)	-1.96(-4.13,0.26)	-3.04(-3.55,-2.52)	-1.56(-1.88,-1.24)	-0.05(-0.14,0.03)
Qatar	-	0.10(0.05,0.14)	7.11(6.56,7.66)	0.07(-0.05,0.20)	-0.64(-1.21,-0.07)	-0.04(-0.16,0.07)	5.93(5.57,6.29)	0.07(-0.05,0.18)
Republic of Cabo Verde	-	-0.05(-0.09,-0.01)	1.12(0.63,1.61)	-0.13(-0.32,0.06)	-0.23(-0.52,0.06)	-0.16(-0.20,-0.11)	-1.53(-1.93,-1.13)	-0.04(-0.10,0.01)
Republic of C 么 te d'Ivoire	-	0.01(0.01,0.01)	0.43(0.22,0.64)	-0.67(-0.74,-0.60)	-1.03(-1.70,-0.36)	-0.06(-0.14,0.02)	0.05(-0.11,0.22)	-0.94(-1.09,-0.80)
Republic of Korea	-	-0.34(-0.42,-0.25)	-4.09(-4.95,-3.22)	0.29(0.13,0.44)	-2.78(-3.59,-1.98)	0.22(0.10,0.35)	-0.43(-0.97,0.12)	0.31(0.23,0.40)
Republic of Moldova	-	-0.69(-0.82,-0.57)	-0.33(-0.85,0.20)	0.17(0.07,0.28)	0.71(0.04,1.38)	-0.53(-0.69,-0.37)	0.64(0.29,0.98)	-0.29(-0.44,-0.15)
Republic of Nauru	-	0.02(0.02,0.02)	0.89(-1.94,3.81)	-1.51(-2.22,-0.79)	-2.32(-2.51,-2.12)	0.22(0.20,0.24)	1.21(-1.09,3.56)	0.16(0.06,0.26)
Republic of Niue	-	0.19(0.11,0.27)	-1.23(-2.19,-0.26)	0.13(0.08,0.19)	-2.45(-2.68,-2.21)	0.03(0.02,0.05)	-2.03(-3.60,-0.45)	0.26(0.21,0.32)
Republic of Palau	-	0.01(0.01,0.01)	-1.78(-2.52,-1.03)	-0.31(-0.39,-0.23)	1.84(1.59,2.10)	0.09(0.05,0.12)	-3.10(-4.11,-2.07)	-0.13(-0.26,-0.01)
Republic of San Marino	-	-0.39(-0.51,-0.27)	-5.24(-6.15,-4.33)	-0.27(-0.36,-0.17)	1.14(0.82,1.46)	0.22(0.07,0.37)	-0.16(-0.58,0.27)	-0.38(-0.63,-0.13)
Republic of the Gambia	-	0.00(-0.06,0.07)	-4.69(-5.65,-3.72)	0.25(0.12,0.37)	-0.72(-1.42,-0.02)	-0.28(-0.32,-0.24)	-1.82(-2.26,-1.38)	0.40(0.32,0.48)
Romania	-	0.06(-0.04,0.16)	-4.32(-5.11,-3.53)	0.27(-0.05,0.59)	-0.74(-0.96,-0.51)	-0.26(-0.66,0.13)	0.15(-0.62,0.92)	0.53(0.38,0.69)
Russian Federation	-	0.76(0.56,0.97)	-2.59(-3.11,-2.06)	0.20(0.16,0.23)	0.74(-0.43,1.92)	-0.73(-1.55,0.09)	-0.69(-1.17,-0.21)	0.03(-0.12,0.19)
Rwanda	-	0.01(0.01,0.02)	-2.48(-3.74,-1.21)	-0.06(-0.22,0.10)	-0.48(-0.69,-0.26)	0.17(0.13,0.22)	-0.43(-1.60,0.76)	0.55(0.50,0.61)
Saint Kitts and Nevis	-	-0.12(-0.32,0.08)	0.10(-2.04,2.29)	0.00(-0.04,0.04)	-2.88(-4.30,-1.44)	-0.10(-0.18,-0.02)	0.82(-1.08,2.75)	0.52(0.49,0.55)

Location	Cannabis		Cocaine		Opioid		Other drug	
	EAPC of ASMR	EAPC of ASPR	EAPC of ASMR	EAPC of ASPR	EAPC of ASMR	EAPC of ASPR	EAPC of ASMR	EAPC of ASPR
Saint Lucia	-	0.15(0.07,0.24)	-1.97(-3.93,0.02)	0.37(0.18,0.57)	-2.25(-3.83,-0.64)	-0.19(-0.27,-0.11)	0.03(-1.90,2.00)	0.46(0.44,0.49)
Saint Vincent and the Grenadines	-	0.44(0.26,0.62)	-0.38(-0.78,0.03)	-0.21(-0.26,-0.16)	3.02(-0.23,6.37)	-0.24(-0.31,-0.16)	-1.53(-1.97,-1.09)	-0.09(-0.13,-0.05)
Samoa	-	0.01(-0.00,0.02)	1.66(1.31,2.01)	0.27(0.26,0.29)	-2.00(-2.33,-1.67)	0.12(0.09,0.14)	1.62(1.22,2.03)	0.95(0.79,1.10)
Sao Tome and Principe	-	0.05(0.05,0.06)	4.37(4.05,4.70)	0.25(0.23,0.27)	1.98(1.87,2.10)	-0.00(-0.07,0.06)	8.67(8.15,9.20)	0.47(0.35,0.59)
Saudi Arabia	-	0.01(-0.01,0.03)	1.87(1.73,2.02)	0.62(0.54,0.69)	3.07(2.49,3.66)	0.12(0.03,0.22)	2.65(2.37,2.94)	0.58(0.52,0.64)
Senegal	-	0.04(0.04,0.04)	2.52(0.84,4.22)	0.36(0.32,0.41)	-0.50(-1.22,0.21)	-0.39(-0.45,-0.34)	5.08(3.94,6.23)	0.48(0.39,0.57)
Serbia	-	-0.63(-0.81,-0.45)	-1.99(-2.45,-1.52)	0.18(0.14,0.23)	1.11(0.51,1.71)	1.38(1.02,1.74)	-0.44(-0.63,-0.24)	0.53(0.40,0.65)
Seychelles	-	-0.18(-0.43,0.08)	1.10(0.37,1.83)	-0.23(-0.29,-0.16)	1.19(0.44,1.93)	0.11(0.02,0.19)	0.44(-0.09,0.98)	-0.14(-0.22,-0.06)
Sierra Leone	-	0.07(0.05,0.08)	2.50(0.70,4.33)	0.46(0.40,0.53)	-0.80(-1.56,-0.03)	-0.38(-0.44,-0.32)	4.64(3.33,5.97)	0.36(0.24,0.48)
Singapore	-	-0.00(-0.01,0.00)	-1.57(-2.65,-0.47)	-0.01(-0.07,0.05)	-1.30(-2.30,-0.28)	-0.12(-0.22,-0.02)	-3.53(-4.53,-2.51)	-0.19(-0.33,-0.05)
Slovakia	-	-0.16(-0.21,-0.11)	-2.88(-3.37,-2.39)	-0.22(-0.47,0.04)	0.52(0.28,0.77)	-0.64(-1.05,-0.23)	-0.39(-0.55,-0.23)	-0.50(-0.55,-0.44)
Slovenia	-	-0.63(-0.75,-0.50)	-4.72(-5.35,-4.10)	0.54(0.40,0.68)	1.39(0.86,1.92)	3.90(3.05,4.75)	-0.91(-1.36,-0.46)	1.36(1.28,1.44)
Socialist Republic of Viet Nam	-	0.15(0.11,0.19)	-0.68(-1.05,-0.30)	-0.27(-0.34,-0.20)	0.80(0.72,0.89)	1.56(1.20,1.93)	-1.27(-1.60,-0.93)	-0.21(-0.34,-0.08)
Solomon Islands	-	0.01(0.00,0.02)	-0.72(-0.92,-0.52)	0.06(0.02,0.09)	-1.65(-1.95,-1.34)	0.15(0.10,0.20)	0.09(-0.02,0.19)	-0.32(-0.49,-0.15)
Somalia	-	-0.00(-0.17,0.16)	-0.26(-0.66,0.15)	0.70(0.39,1.01)	-0.24(-0.36,-0.12)	-0.09(-0.12,-0.05)	-0.06(-0.60,0.48)	0.36(0.32,0.39)
South Africa	-	-0.11(-0.12,-0.09)	-0.23(-0.56,0.09)	-0.11(-0.17,-0.05)	-0.37(-0.87,0.13)	-2.10(-2.60,-1.60)	1.50(1.35,1.65)	-0.06(-0.10,-0.02)
South Sudan	-	0.66(0.56,0.75)	-1.53(-2.09,-0.96)	-1.45(-2.72,-0.15)	-0.02(-0.12,0.08)	-0.06(-0.11,-0.02)	0.18(-0.15,0.51)	-0.10(-0.17,-0.04)
Spain	-	-0.01(-0.01,-0.01)	-2.82(-3.48,-2.15)	-0.15(-0.21,-0.10)	-2.37(-2.80,-1.95)	-2.00(-2.64,-1.37)	-3.66(-4.45,-2.88)	-0.56(-0.76,-0.36)
Sri Lanka	-	0.04(0.02,0.05)	-0.47(-0.61,-0.32)	0.13(0.12,0.14)	-3.20(-3.74,-2.65)	-1.45(-1.63,-1.27)	1.75(1.58,1.93)	0.44(0.39,0.50)
Sudan	-	-0.09(-0.13,-0.06)	2.04(0.29,3.81)	0.31(0.16,0.46)	1.02(0.87,1.16)	0.57(0.53,0.60)	2.32(0.85,3.82)	0.84(0.76,0.91)
Suriname	-	-0.43(-0.62,-0.23)	6.74(5.79,7.69)	1.25(1.09,1.41)	0.17(-1.42,1.78)	0.03(-0.04,0.10)	6.28(5.59,6.97)	2.06(1.92,2.20)
Sweden	-	-0.62(-0.74,-0.49)	-1.37(-1.67,-1.06)	-0.17(-0.28,-0.05)	4.49(3.87,5.11)	5.81(5.55,6.07)	0.76(0.47,1.05)	0.28(0.24,0.31)
Switzerland	-	0.14(0.06,0.23)	0.88(0.48,1.29)	0.34(0.31,0.37)	-3.20(-3.49,-2.90)	-3.58(-3.92,-3.25)	1.97(1.62,2.32)	0.13(0.06,0.21)
Syrian Arab Republic	-	0.00(-0.00,0.00)	-14.71(-17.15,-12.20)	0.02(0.01,0.04)	1.75(1.39,2.11)	0.42(0.40,0.44)	0.78(0.44,1.13)	0.74(0.70,0.79)
Taiwan (Province of China)	-	0.03(0.02,0.03)	1.91(1.78,2.05)	-0.17(-0.22,-0.12)	-2.65(-3.37,-1.92)	0.15(-0.39,0.69)	-0.61(-0.83,-0.40)	-0.17(-0.47,0.14)
Tajikistan	-	0.12(0.08,0.15)	0.88(0.65,1.10)	0.17(-0.10,0.44)	1.14(0.98,1.31)	-0.47(-0.57,-0.37)	0.12(-0.28,0.52)	0.21(0.10,0.31)
Thailand	-	-0.03(-0.04,-0.03)	-0.16(-0.70,0.37)	-0.30(-0.39,-0.22)	0.72(0.49,0.95)	0.03(-0.30,0.35)	0.04(-0.40,0.49)	0.36(0.27,0.45)
The former Yugoslav Republic of Macedonia	-	-0.07(-0.13,-0.02)	1.78(0.26,3.32)	0.45(0.37,0.53)	1.46(0.88,2.04)	1.39(0.92,1.86)	3.67(2.63,4.71)	0.48(0.41,0.56)
Timor-Leste	-	0.04(0.02,0.07)	-0.61(-0.91,-0.31)	-0.05(-0.09,-0.02)	-0.48(-0.81,-0.16)	0.02(-0.07,0.11)	-1.36(-1.72,-0.99)	0.30(0.26,0.34)
Togo	-	0.11(0.04,0.18)	-0.01(-0.21,0.19)	-0.15(-0.20,-0.09)	-1.14(-1.78,-0.49)	0.39(0.33,0.44)	-0.96(-1.21,-0.70)	0.18(0.13,0.22)

Location	Cannabis		Cocaine		Opioid		Other drug	
	EAPC of ASMR	EAPC of ASPR	EAPC of ASMR	EAPC of ASPR	EAPC of ASMR	EAPC of ASPR	EAPC of ASMR	EAPC of ASPR
Tokelau	-	0.12(0.09,0.15)	2.29(0.37,4.25)	1.02(0.91,1.13)	-2.09(-2.40,-1.78)	0.08(0.07,0.09)	2.71(1.15,4.29)	0.79(0.73,0.85)
Tonga	-	-0.02(-0.02,-0.01)	0.35(0.05,0.65)	0.15(0.14,0.17)	-1.54(-1.71,-1.38)	0.22(0.19,0.26)	1.13(0.78,1.49)	0.53(0.49,0.57)
Trinidad and Tobago	-	0.07(0.06,0.08)	-0.13(-0.26,-0.00)	0.43(0.31,0.55)	2.14(0.31,4.00)	-0.20(-0.28,-0.13)	-0.42(-0.57,-0.27)	0.26(0.18,0.34)
Tunisia	-	0.09(0.08,0.10)	8.91(7.77,10.05)	0.32(0.30,0.34)	0.79(0.63,0.95)	0.75(0.67,0.82)	7.41(6.44,8.38)	0.49(0.24,0.75)
Turkey	-	-0.62(-0.80,-0.44)	-0.37(-0.66,-0.08)	-0.06(-0.10,-0.01)	0.80(0.46,1.14)	-0.09(-0.18,0.00)	-1.10(-1.38,-0.82)	0.15(0.11,0.19)
Turkmenistan	-	-0.21(-0.31,-0.12)	0.28(-0.09,0.65)	0.39(0.38,0.40)	6.08(4.93,7.24)	0.44(0.38,0.50)	3.08(2.98,3.19)	0.24(0.13,0.35)
Tuvalu	-	-0.26(-0.32,-0.21)	-2.56(-3.41,-1.70)	0.31(0.29,0.33)	-1.55(-1.77,-1.33)	0.14(0.13,0.16)	-1.27(-2.04,-0.50)	0.11(-0.07,0.29)
Uganda	-	-0.01(-0.10,0.09)	0.60(0.18,1.02)	0.59(0.56,0.62)	0.63(0.54,0.73)	-0.04(-0.10,0.02)	1.02(0.39,1.66)	0.09(-0.01,0.19)
Ukraine	-	-0.12(-0.18,-0.06)	3.74(3.31,4.19)	2.99(2.19,3.80)	-1.88(-2.70,-1.06)	0.84(0.67,1.02)	3.78(3.22,4.33)	1.70(1.56,1.85)
United Arab Emirates	-	-0.41(-0.60,-0.22)	0.37(0.23,0.52)	0.73(0.69,0.78)	0.30(0.08,0.52)	1.95(1.81,2.08)	1.61(1.41,1.80)	0.66(0.58,0.75)
United Kingdom of Great Britain and Northern Ireland	-	-0.05(-0.08,-0.02)	7.06(6.46,7.66)	0.44(0.26,0.61)	4.14(3.35,4.94)	1.94(1.49,2.39)	5.98(5.58,6.38)	2.70(2.55,2.85)
United Republic of Tanzania	-	0.03(0.01,0.05)	1.93(1.61,2.25)	0.32(0.20,0.43)	0.31(0.27,0.36)	-0.01(-0.11,0.09)	1.89(1.64,2.13)	1.08(0.91,1.25)
United States of America	-	0.04(-0.00,0.09)	3.84(3.33,4.34)	0.18(-0.11,0.46)	8.10(7.83,8.38)	6.73(6.21,7.25)	1.78(1.06,2.50)	-0.04(-0.19,0.12)
United States Virgin Islands	-	0.02(0.01,0.02)	4.21(3.50,4.93)	0.16(0.13,0.18)	-0.20(-0.90,0.50)	-0.74(-0.85,-0.63)	4.18(3.48,4.88)	0.23(-0.01,0.48)
Uruguay	-	-0.04(-0.05,-0.03)	-0.73(-1.05,-0.41)	-0.22(-0.28,-0.17)	4.70(4.09,5.31)	-0.63(-0.70,-0.57)	-1.38(-1.70,-1.05)	-0.04(-0.10,0.02)
Uzbekistan	-	-0.10(-0.16,-0.04)	-0.76(-1.45,-0.06)	0.22(0.08,0.37)	3.15(2.47,3.84)	-0.25(-0.36,-0.15)	1.98(1.61,2.35)	0.07(-0.02,0.15)
Vanuatu	-	0.03(0.03,0.04)	0.99(0.90,1.08)	0.06(0.02,0.09)	-1.89(-2.17,-1.60)	0.18(0.16,0.19)	1.12(1.08,1.16)	0.76(0.73,0.80)
Yemen	-	-0.02(-0.03,-0.01)	-1.26(-1.39,-1.12)	0.09(0.07,0.11)	1.17(0.89,1.45)	0.54(0.50,0.59)	2.34(1.96,2.73)	0.28(0.21,0.35)
Zambia	-	-0.59(-0.78,-0.40)	-0.09(-0.27,0.08)	0.68(0.66,0.71)	0.47(0.39,0.55)	-0.23(-0.26,-0.19)	1.64(1.39,1.90)	0.54(0.43,0.66)
Zimbabwe	-	-0.03(-0.03,-0.03)	1.00(0.73,1.27)	-0.30(-0.36,-0.23)	0.33(0.16,0.50)	-0.32(-0.35,-0.29)	0.56(0.13,0.99)	0.04(-0.03,0.12)

Table S9. EAPC of ASMR ,ASPR ,ASIR and ASDR for the amphetamine use disorders in 204 countries and territories from 1990 to 2021

Location	Amphatamine			
	EAPC of ASDR	EAPC of ASIR	EAPC of ASMR	EAPC of ASPR
American Samoa	0.52(0.38,0.65)	0.09(0.08,0.10)	10.89(7.98,13.88)	0.08(0.06,0.09)
Antigua and Barbuda	0.73(0.44,1.02)	0.30(0.25,0.35)	4.44(2.45,6.47)	0.34(0.26,0.42)
Arab Republic of Egypt	0.26(0.24,0.29)	0.18(0.15,0.20)	5.24(4.77,5.70)	0.18(0.16,0.20)
Argentine Republic	0.12(-0.03,0.28)	-0.01(-0.12,0.10)	9.52(8.41,10.64)	-0.03(-0.20,0.13)
Australia	0.44(0.03,0.85)	-0.82(-1.26,-0.38)	8.63(7.34,9.94)	-0.25(-0.74,0.24)
Barbados	0.36(0.17,0.54)	0.08(-0.03,0.18)	6.33(4.33,8.37)	0.08(-0.03,0.18)
Belize	0.30(0.15,0.45)	0.14(0.10,0.19)	4.27(2.15,6.43)	0.14(0.09,0.19)
Bermuda	1.17(0.50,1.84)	0.27(0.15,0.38)	4.12(2.05,6.22)	0.36(0.15,0.57)
Bolivarian Republic of Venezuela	0.21(0.13,0.29)	-0.01(-0.03,0.01)	3.48(2.57,4.40)	-0.02(-0.04,0.00)
Bosnia and Herzegovina	0.29(0.23,0.35)	0.16(0.10,0.22)	3.19(2.86,3.52)	0.16(0.10,0.23)
Brunei Darussalam	-0.04(-0.08,0.01)	-0.06(-0.09,-0.03)	1.19(0.90,1.48)	-0.11(-0.14,-0.07)
Burkina Faso	0.00(-0.02,0.03)	-0.03(-0.05,-0.01)	4.29(2.61,6.00)	-0.03(-0.06,-0.01)
Canada	2.48(2.23,2.72)	-0.02(-0.12,0.08)	10.65(10.01,11.28)	-0.07(-0.17,0.04)
Central African Republic	0.10(0.06,0.13)	0.02(-0.00,0.04)	1.47(1.38,1.56)	0.01(-0.02,0.03)
Commonwealth of Dominica	0.65(0.54,0.76)	0.24(0.19,0.29)	4.52(3.56,5.49)	0.28(0.22,0.34)
Commonwealth of the Bahamas	0.62(0.38,0.85)	0.21(0.16,0.26)	5.01(2.94,7.12)	0.25(0.19,0.31)
Cook Islands	0.21(0.18,0.23)	-0.02(-0.05,0.01)	6.86(6.03,7.69)	-0.05(-0.09,-0.02)
Czech Republic	1.20(0.93,1.47)	1.25(0.50,2.00)	3.52(3.14,3.90)	1.06(0.77,1.36)
Democratic People's Republic of Korea	0.11(0.06,0.16)	0.26(0.23,0.29)	-1.62(-1.84,-1.40)	0.26(0.22,0.29)
Democratic Republic of Sao Tome and Principe	0.40(0.36,0.43)	0.01(-0.01,0.03)	8.58(8.20,8.96)	0.00(-0.02,0.03)
Democratic Republic of the Congo	0.17(0.14,0.20)	0.08(0.07,0.10)	1.86(1.33,2.39)	0.09(0.07,0.10)
Democratic Republic of Timor-Leste	0.05(0.01,0.09)	-0.03(-0.05,-0.01)	2.13(1.62,2.65)	-0.05(-0.08,-0.02)
Democratic Socialist Republic of Sri Lanka	-0.30(-0.41,-0.19)	-0.05(-0.07,-0.03)	-2.19(-3.20,-1.18)	-0.11(-0.13,-0.09)
Dominican Republic	0.57(0.51,0.63)	0.32(0.29,0.36)	6.32(4.98,7.68)	0.38(0.34,0.41)
Eastern Republic of Uruguay	0.45(0.41,0.50)	0.14(0.10,0.17)	8.41(7.59,9.24)	0.16(0.12,0.20)
Federal Democratic Republic of Ethiopia	-0.08(-0.09,-0.06)	-0.02(-0.04,-0.00)	-1.70(-2.05,-1.35)	-0.04(-0.06,-0.01)
Federal Democratic Republic of Nepal	-0.08(-0.12,-0.03)	-0.10(-0.12,-0.08)	-0.07(-0.52,0.38)	-0.11(-0.13,-0.08)
Federal Republic of Germany	1.70(1.47,1.94)	0.76(0.59,0.93)	6.59(5.78,7.40)	1.04(0.81,1.27)
Federal Republic of Nigeria	-0.06(-0.10,-0.01)	-0.10(-0.15,-0.06)	5.26(3.67,6.88)	-0.14(-0.19,-0.10)
Federal Republic of Somalia	0.04(0.00,0.07)	-0.00(-0.02,0.02)	0.51(0.41,0.61)	0.00(-0.02,0.02)

Federated States of Micronesia	0.14(0.11,0.16)	0.11(0.09,0.12)	1.68(1.45,1.91)	0.09(0.07,0.12)
Federative Republic of Brazil	-0.14(-0.23,-0.05)	-0.19(-0.35,-0.03)	4.58(3.57,5.59)	-0.21(-0.31,-0.11)
French Republic	1.95(1.74,2.16)	0.45(0.22,0.68)	6.38(5.73,7.04)	0.52(0.25,0.79)
Gabonese Republic	0.65(0.59,0.71)	0.08(0.07,0.09)	5.11(4.86,5.37)	0.08(0.07,0.09)
Georgia	0.47(0.31,0.62)	0.09(0.06,0.12)	6.69(4.88,8.52)	0.08(0.04,0.11)
Grand Duchy of Luxembourg	0.75(0.57,0.93)	0.13(0.05,0.22)	3.02(2.39,3.65)	0.16(0.06,0.26)
Greenland	0.59(0.48,0.71)	-0.25(-0.29,-0.20)	6.02(5.27,6.77)	-0.33(-0.38,-0.27)
Grenada	1.12(0.77,1.47)	0.25(0.18,0.32)	7.55(5.26,9.89)	0.26(0.15,0.37)
Guam	0.37(0.24,0.50)	0.31(0.24,0.38)	1.50(-0.19,3.23)	0.32(0.26,0.39)
Hashemite Kingdom of Jordan	-0.03(-0.08,0.02)	-0.02(-0.04,-0.00)	-0.03(-0.60,0.54)	-0.04(-0.06,-0.02)
Hellenic Republic	2.28(1.96,2.61)	0.82(0.58,1.05)	7.77(7.06,8.48)	0.93(0.66,1.21)
Hungary	0.89(0.58,1.20)	0.71(0.40,1.03)	2.77(2.35,3.20)	0.81(0.49,1.13)
Independent State of Papua New Guinea	0.03(-0.01,0.06)	0.02(-0.01,0.04)	1.46(1.13,1.79)	-0.01(-0.04,0.02)
Independent State of Samoa	0.03(0.02,0.05)	0.01(0.00,0.02)	1.78(1.28,2.28)	-0.01(-0.02,0.01)
Ireland	3.14(2.96,3.33)	1.25(1.01,1.48)	7.07(6.33,7.82)	1.49(1.21,1.78)
Islamic Republic of Afghanistan	-0.33(-0.54,-0.11)	0.13(0.11,0.16)	-1.05(-1.52,-0.58)	0.16(0.12,0.20)
Islamic Republic of Iran	1.19(0.86,1.51)	0.85(0.73,0.97)	1.40(0.87,1.93)	0.98(0.78,1.17)
Islamic Republic of Mauritania	-0.02(-0.05,0.01)	-0.04(-0.07,-0.02)	3.12(1.59,4.67)	-0.05(-0.08,-0.02)
Islamic Republic of Pakistan	0.34(0.22,0.46)	-0.12(-0.22,-0.01)	1.61(1.37,1.86)	-0.13(-0.25,-0.00)
Jamaica	0.35(0.27,0.43)	0.17(0.13,0.21)	3.87(2.88,4.88)	0.17(0.13,0.21)
Japan	0.02(-0.06,0.10)	-0.08(-0.13,-0.04)	5.31(4.05,6.58)	-0.11(-0.17,-0.05)
Kingdom of Bahrain	0.27(0.16,0.38)	0.09(0.06,0.12)	1.86(0.92,2.82)	0.08(0.04,0.13)
Kingdom of Belgium	1.17(1.00,1.33)	0.20(0.05,0.35)	6.17(5.42,6.92)	0.25(0.09,0.41)
Kingdom of Bhutan	-0.06(-0.08,-0.04)	-0.01(-0.03,0.00)	-0.58(-0.89,-0.27)	-0.02(-0.03,0.00)
Kingdom of Cambodia	0.09(0.06,0.12)	0.01(-0.01,0.03)	0.91(0.65,1.18)	0.01(-0.01,0.04)
Kingdom of Denmark	1.26(1.13,1.39)	-0.01(-0.09,0.08)	4.55(3.99,5.11)	0.06(-0.02,0.15)
Kingdom of Eswatini	0.32(0.27,0.37)	0.21(0.20,0.22)	1.97(1.38,2.57)	0.23(0.21,0.24)
Kingdom of Lesotho	0.39(0.34,0.44)	0.20(0.19,0.22)	3.42(2.74,4.11)	0.21(0.20,0.23)
Kingdom of Morocco	0.50(0.47,0.54)	0.07(0.06,0.09)	2.00(1.81,2.19)	0.06(0.04,0.08)
Kingdom of Norway	3.40(2.57,4.23)	1.53(1.26,1.81)	9.04(6.68,11.46)	1.70(1.38,2.01)
Kingdom of Saudi Arabia	-0.05(-0.09,-0.01)	-0.05(-0.08,-0.02)	4.49(4.18,4.80)	-0.09(-0.12,-0.05)
Kingdom of Spain	0.38(0.14,0.61)	-0.09(-0.65,0.49)	3.31(2.79,3.82)	0.05(-0.18,0.28)
Kingdom of Sweden	3.21(3.01,3.41)	2.01(1.87,2.15)	4.90(4.27,5.54)	2.35(2.16,2.53)

Kingdom of Thailand	0.21(-0.01,0.43)	0.10(-0.18,0.38)	3.96(3.63,4.30)	0.13(-0.10,0.35)
Kingdom of the Netherlands	0.92(0.67,1.17)	0.31(0.06,0.56)	6.34(5.74,6.95)	0.56(0.28,0.84)
Kingdom of Tonga	0.06(0.03,0.09)	0.03(0.01,0.05)	2.69(2.52,2.86)	0.01(-0.03,0.04)
Kyrgyz Republic	0.82(0.45,1.19)	0.06(0.03,0.09)	4.17(2.64,5.72)	0.05(0.01,0.08)
Lao People's Democratic Republic	-0.10(-0.36,0.15)	-0.13(-0.40,0.13)	1.37(1.12,1.62)	-0.16(-0.43,0.10)
Lebanese Republic	0.44(0.34,0.55)	0.04(-0.05,0.12)	3.89(3.38,4.39)	0.07(-0.02,0.16)
Malaysia	0.09(0.07,0.12)	0.03(0.02,0.05)	3.16(2.76,3.58)	0.04(0.02,0.06)
Mongolia	0.44(0.33,0.54)	0.12(0.08,0.17)	6.34(5.21,7.47)	0.10(0.06,0.15)
Montenegro	0.21(0.15,0.27)	0.03(-0.02,0.08)	3.95(3.70,4.21)	0.01(-0.05,0.07)
New Zealand	-0.36(-0.78,0.07)	-0.75(-1.19,-0.31)	10.18(9.12,11.25)	-0.84(-1.29,-0.38)
North Macedonia	0.54(0.47,0.61)	0.19(0.14,0.23)	3.76(3.26,4.26)	0.20(0.14,0.25)
Northern Mariana Islands	1.29(0.92,1.67)	1.11(0.84,1.37)	3.51(0.72,6.37)	1.15(0.83,1.47)
Palestine	-0.07(-0.10,-0.03)	-0.05(-0.08,-0.02)	4.66(4.09,5.23)	-0.08(-0.11,-0.05)
People's Democratic Republic of Algeria	0.64(0.58,0.69)	0.09(0.08,0.10)	2.16(1.90,2.42)	0.09(0.08,0.10)
People's Republic of Bangladesh	-0.03(-0.09,0.03)	-0.09(-0.10,-0.08)	0.10(-0.26,0.45)	-0.09(-0.10,-0.08)
People's Republic of China	-2.74(-3.16,-2.33)	-2.27(-2.60,-1.94)	-4.15(-4.99,-3.31)	-2.40(-2.74,-2.05)
Plurinational State of Bolivia	0.35(0.33,0.37)	0.18(0.17,0.20)	2.35(2.27,2.42)	0.20(0.19,0.22)
Portuguese Republic	0.01(-0.41,0.42)	-0.38(-0.83,0.06)	3.71(3.32,4.10)	-0.43(-0.94,0.09)
Principality of Andorra	-0.11(-0.14,-0.08)	-0.11(-0.13,-0.09)	3.33(2.96,3.70)	-0.14(-0.17,-0.11)
Principality of Monaco	-0.09(-0.13,-0.05)	-0.09(-0.12,-0.06)	5.88(4.93,6.84)	-0.12(-0.15,-0.09)
Puerto Rico	0.86(-0.31,2.04)	-0.88(-1.46,-0.30)	6.42(3.98,8.93)	-1.60(-2.54,-0.65)
Republic of Albania	0.93(0.88,0.99)	0.63(0.59,0.68)	3.31(2.78,3.84)	0.68(0.64,0.73)
Republic of Angola	0.18(0.14,0.22)	0.02(0.01,0.04)	4.16(3.76,4.56)	0.02(0.00,0.04)
Republic of Armenia	0.14(0.05,0.24)	-0.00(-0.04,0.03)	7.73(5.78,9.72)	-0.03(-0.08,0.01)
Republic of Austria	1.08(1.01,1.16)	0.53(0.44,0.62)	6.00(5.39,6.62)	0.61(0.50,0.71)
Republic of Azerbaijan	0.20(0.15,0.26)	0.11(0.07,0.15)	6.12(5.14,7.11)	0.09(0.05,0.14)
Republic of Belarus	0.21(0.09,0.33)	-0.07(-0.12,-0.02)	1.75(1.23,2.26)	-0.08(-0.14,-0.01)
Republic of Benin	0.04(0.01,0.07)	0.00(-0.02,0.03)	4.40(2.80,6.03)	0.01(-0.02,0.03)
Republic of Botswana	0.27(0.21,0.33)	0.18(0.17,0.20)	1.26(0.60,1.92)	0.20(0.18,0.21)
Republic of Bulgaria	0.80(0.73,0.88)	0.52(0.46,0.58)	3.95(3.35,4.55)	0.54(0.47,0.61)
Republic of Burundi	0.01(-0.02,0.04)	0.01(-0.00,0.03)	-0.10(-0.35,0.15)	0.01(-0.01,0.02)
Republic of Cabo Verde	0.10(0.07,0.13)	0.04(0.01,0.07)	7.84(7.61,8.07)	0.04(0.01,0.07)
Republic of Cameroon	0.06(0.02,0.09)	-0.00(-0.02,0.02)	4.60(3.14,6.09)	-0.01(-0.03,0.02)

Republic of Chad	-0.02(-0.06,0.02)	-0.03(-0.05,-0.01)	3.93(2.30,5.58)	-0.04(-0.06,-0.01)
Republic of Chile	0.08(-0.06,0.22)	-0.15(-0.26,-0.05)	6.10(5.62,6.59)	-0.16(-0.29,-0.03)
Republic of Colombia	0.11(0.01,0.20)	-0.06(-0.08,-0.04)	1.58(1.13,2.04)	-0.08(-0.10,-0.05)
Republic of Costa Rica	0.78(0.69,0.86)	0.11(0.10,0.12)	5.36(4.72,6.02)	0.12(0.11,0.13)
Republic of Croatia	0.57(0.44,0.70)	0.26(0.22,0.31)	2.38(1.74,3.03)	0.28(0.23,0.33)
Republic of Cuba	0.16(0.11,0.20)	0.16(0.12,0.19)	0.03(-0.63,0.69)	0.17(0.13,0.20)
Republic of Cyprus	0.28(0.21,0.36)	-0.03(-0.15,0.09)	2.42(1.76,3.08)	-0.03(-0.17,0.12)
Republic of C 么 te d'Ivoire	0.02(-0.02,0.05)	-0.03(-0.05,-0.02)	3.45(2.26,4.67)	-0.05(-0.08,-0.02)
Republic of Djibouti	0.27(0.25,0.29)	0.06(0.04,0.08)	3.72(3.56,3.88)	0.03(0.02,0.05)
Republic of Ecuador	0.90(0.81,0.99)	0.17(0.15,0.18)	7.30(6.45,8.16)	0.19(0.17,0.21)
Republic of El Salvador	0.70(0.62,0.79)	-0.06(-0.08,-0.04)	4.67(4.12,5.23)	-0.07(-0.10,-0.05)
Republic of Equatorial Guinea	1.12(0.98,1.26)	0.32(0.29,0.34)	9.02(8.47,9.58)	0.37(0.34,0.40)
Republic of Estonia	2.25(1.84,2.67)	1.93(1.86,2.01)	2.34(1.15,3.54)	2.19(2.10,2.27)
Republic of Fiji	-0.04(-0.07,-0.01)	0.01(-0.01,0.04)	-1.98(-3.16,-0.79)	-0.01(-0.04,0.02)
Republic of Finland	2.04(1.79,2.30)	-0.45(-1.01,0.12)	7.05(6.26,7.84)	0.71(0.48,0.93)
Republic of Ghana	-0.05(-0.08,-0.02)	-0.04(-0.07,-0.02)	-0.90(-1.43,-0.37)	-0.04(-0.07,-0.01)
Republic of Guatemala	1.66(1.42,1.90)	0.34(0.23,0.44)	3.28(2.83,3.73)	0.39(0.26,0.52)
Republic of Guinea	0.01(-0.03,0.04)	-0.05(-0.07,-0.03)	5.40(3.86,6.96)	-0.05(-0.08,-0.02)
Republic of Guinea-Bissau	-0.00(-0.03,0.03)	-0.03(-0.05,-0.02)	3.92(2.54,5.32)	-0.04(-0.07,-0.02)
Republic of Guyana	0.50(0.29,0.71)	0.24(0.08,0.41)	8.26(5.86,10.72)	0.28(0.09,0.47)
Republic of Haiti	0.46(0.42,0.50)	0.27(0.24,0.31)	2.46(2.24,2.68)	0.30(0.27,0.34)
Republic of Honduras	0.63(0.60,0.66)	0.11(0.10,0.11)	3.76(3.63,3.89)	0.14(0.12,0.15)
Republic of Iceland	2.50(2.37,2.64)	0.98(0.88,1.07)	6.27(5.72,6.83)	1.19(1.08,1.31)
Republic of India	0.38(0.27,0.49)	0.14(0.13,0.15)	1.00(0.64,1.36)	0.18(0.16,0.19)
Republic of Indonesia	0.04(-0.00,0.09)	-0.03(-0.06,0.01)	4.97(4.70,5.23)	-0.07(-0.11,-0.02)
Republic of Iraq	0.33(0.29,0.36)	0.00(-0.02,0.02)	3.69(3.48,3.91)	-0.02(-0.04,0.01)
Republic of Italy	-0.03(-0.54,0.49)	-0.04(-0.66,0.58)	2.26(1.42,3.10)	-0.16(-0.67,0.37)
Republic of Kazakhstan	1.98(1.69,2.27)	0.66(0.60,0.72)	5.85(4.62,7.10)	1.11(1.06,1.15)
Republic of Kenya	0.25(0.23,0.27)	0.03(0.02,0.05)	4.15(4.04,4.27)	0.02(0.00,0.04)
Republic of Kiribati	1.10(1.02,1.18)	0.10(0.09,0.11)	2.73(2.54,2.92)	0.08(0.07,0.10)
Republic of Korea	0.03(-0.02,0.07)	0.00(-0.03,0.04)	2.29(1.45,3.13)	-0.03(-0.07,0.01)
Republic of Latvia	0.60(0.29,0.91)	0.41(0.19,0.64)	2.59(1.63,3.55)	0.33(0.09,0.57)
Republic of Liberia	0.12(0.07,0.16)	0.02(-0.01,0.05)	4.68(2.88,6.51)	0.02(-0.01,0.06)

Republic of Lithuania	1.92(1.66,2.19)	1.09(0.74,1.44)	3.52(2.48,4.57)	1.31(0.89,1.72)
Republic of Madagascar	0.11(0.08,0.14)	0.01(-0.00,0.02)	1.48(1.19,1.78)	0.00(-0.01,0.01)
Republic of Malawi	0.22(0.20,0.24)	0.03(0.02,0.04)	2.43(2.27,2.60)	0.02(0.01,0.03)
Republic of Maldives	0.46(0.40,0.52)	0.22(0.18,0.26)	8.73(8.46,9.00)	0.27(0.22,0.32)
Republic of Mali	-0.00(-0.03,0.03)	-0.02(-0.04,-0.00)	3.68(1.69,5.70)	-0.03(-0.06,-0.00)
Republic of Malta	1.93(1.85,2.02)	0.90(0.83,0.97)	6.93(6.29,7.58)	1.05(0.97,1.14)
Republic of Mauritius	0.88(0.81,0.96)	0.23(0.20,0.27)	17.25(12.59,22.10)	0.34(0.29,0.39)
Republic of Moldova	0.15(0.06,0.24)	-0.10(-0.14,-0.06)	3.31(2.57,4.06)	-0.13(-0.18,-0.08)
Republic of Mozambique	0.25(0.22,0.27)	0.11(0.10,0.12)	2.99(2.72,3.26)	0.12(0.11,0.13)
Republic of Namibia	0.15(0.13,0.17)	0.10(0.09,0.11)	0.53(0.34,0.71)	0.10(0.09,0.11)
Republic of Nauru	0.11(0.09,0.12)	0.09(0.07,0.10)	1.45(0.88,2.02)	0.06(0.04,0.07)
Republic of Nicaragua	0.24(0.19,0.29)	-0.06(-0.08,-0.04)	3.52(3.01,4.02)	-0.08(-0.10,-0.05)
Republic of Niue	0.16(0.13,0.19)	0.08(0.06,0.11)	1.86(1.63,2.09)	0.05(0.02,0.09)
Republic of Palau	0.20(0.17,0.22)	0.16(0.14,0.18)	0.49(0.31,0.67)	0.19(0.17,0.22)
Republic of Panama	0.59(0.51,0.68)	0.01(-0.01,0.03)	4.50(3.89,5.12)	-0.00(-0.03,0.02)
Republic of Paraguay	0.31(0.28,0.34)	0.17(0.15,0.19)	8.77(8.25,9.29)	0.18(0.16,0.20)
Republic of Peru	0.71(0.61,0.81)	0.26(0.21,0.31)	4.57(3.95,5.20)	0.27(0.21,0.34)
Republic of Poland	0.44(0.35,0.52)	0.28(0.17,0.39)	2.41(1.74,3.08)	0.29(0.19,0.39)
Republic of Rwanda	0.05(-0.01,0.11)	0.03(0.00,0.06)	-0.10(-0.65,0.46)	0.02(-0.01,0.05)
Republic of San Marino	-0.13(-0.16,-0.10)	-0.09(-0.12,-0.07)	2.30(1.81,2.79)	-0.13(-0.16,-0.10)
Republic of Senegal	0.05(0.01,0.08)	-0.00(-0.03,0.02)	5.42(3.70,7.16)	-0.02(-0.05,0.01)
Republic of Serbia	0.79(0.72,0.87)	0.33(0.25,0.40)	3.39(3.08,3.71)	0.39(0.30,0.47)
Republic of Seychelles	0.67(0.53,0.82)	0.13(0.11,0.14)	4.00(2.73,5.30)	0.21(0.19,0.22)
Republic of Sierra Leone	0.05(0.02,0.08)	0.01(-0.01,0.04)	4.91(2.87,6.99)	0.01(-0.02,0.04)
Republic of Singapore	-0.06(-0.12,0.01)	-0.07(-0.11,-0.04)	2.12(1.19,3.05)	-0.08(-0.13,-0.03)
Republic of Slovenia	1.36(1.21,1.51)	1.00(0.91,1.09)	2.39(1.73,3.05)	1.13(1.02,1.23)
Republic of South Africa	-0.22(-0.60,0.17)	-0.29(-0.55,-0.02)	2.64(1.93,3.35)	-0.52(-0.89,-0.15)
Republic of South Sudan	-0.03(-0.05,-0.01)	-0.08(-0.10,-0.07)	2.62(2.08,3.16)	-0.11(-0.13,-0.09)
Republic of Sudan	0.26(0.23,0.28)	0.10(0.09,0.11)	0.68(0.59,0.77)	0.08(0.07,0.09)
Republic of Suriname	1.65(1.32,1.99)	0.39(0.31,0.46)	6.76(5.01,8.53)	0.55(0.44,0.66)
Republic of Tajikistan	0.20(0.15,0.24)	0.10(0.07,0.14)	3.57(2.89,4.26)	0.10(0.07,0.14)
Republic of the Congo	0.39(0.34,0.45)	0.08(0.06,0.09)	3.42(3.06,3.79)	0.07(0.06,0.08)
Republic of the Gambia	-0.03(-0.05,-0.00)	-0.05(-0.07,-0.03)	5.04(3.72,6.38)	-0.06(-0.08,-0.04)

Republic of the Marshall Islands	0.08(0.06,0.10)	0.05(0.03,0.07)	2.07(1.84,2.29)	0.04(0.01,0.07)
Republic of the Niger	0.00(-0.03,0.03)	-0.03(-0.05,-0.00)	2.03(0.39,3.71)	-0.04(-0.07,-0.01)
Republic of the Philippines	-0.13(-0.16,-0.10)	-0.09(-0.11,-0.07)	-0.27(-0.94,0.41)	-0.15(-0.17,-0.12)
Republic of the Union of Myanmar	-0.11(-0.24,0.03)	-0.13(-0.26,0.01)	1.18(0.92,1.44)	-0.15(-0.29,-0.01)
Republic of Trinidad and Tobago	1.22(1.10,1.34)	0.44(0.40,0.47)	7.80(6.05,9.58)	0.56(0.52,0.60)
Republic of Tunisia	0.83(0.76,0.89)	0.11(0.10,0.13)	2.75(2.43,3.08)	0.11(0.10,0.12)
Republic of Turkey	0.95(0.85,1.05)	0.18(0.17,0.20)	3.56(3.29,3.83)	0.17(0.16,0.19)
Republic of Uganda	0.26(0.24,0.28)	0.02(0.01,0.03)	4.01(3.74,4.28)	0.01(-0.00,0.02)
Republic of Uzbekistan	0.37(0.30,0.44)	0.10(0.07,0.14)	7.66(6.70,8.62)	0.09(0.05,0.12)
Republic of Vanuatu	0.03(0.00,0.06)	0.04(0.02,0.06)	2.26(1.85,2.66)	0.00(-0.03,0.03)
Republic of Yemen	0.13(0.07,0.19)	0.01(-0.01,0.03)	0.40(0.22,0.59)	0.00(-0.03,0.03)
Republic of Zambia	0.46(0.39,0.52)	0.07(0.06,0.08)	3.46(2.99,3.93)	0.08(0.07,0.09)
Republic of Zimbabwe	0.04(-0.12,0.20)	-0.15(-0.31,0.00)	0.95(0.40,1.50)	-0.20(-0.37,-0.04)
Romania	0.58(0.51,0.66)	0.11(0.03,0.18)	2.11(1.91,2.30)	0.09(0.01,0.18)
Russian Federation	0.84(0.19,1.50)	-0.29(-0.58,-0.00)	3.55(2.10,5.01)	-0.14(-0.45,0.17)
Saint Kitts and Nevis	-0.03(-0.23,0.18)	0.15(0.09,0.20)	-0.53(-1.48,0.43)	0.13(0.07,0.19)
Saint Lucia	0.48(0.35,0.61)	0.25(0.21,0.29)	4.69(2.76,6.66)	0.29(0.24,0.34)
Saint Vincent and the Grenadines	0.50(0.32,0.69)	0.18(0.13,0.23)	11.07(6.68,15.63)	0.19(0.12,0.25)
Slovak Republic	-0.15(-0.29,-0.01)	-0.21(-0.46,0.03)	3.12(2.80,3.44)	-0.38(-0.54,-0.22)
Socialist Republic of Viet Nam	0.92(0.82,1.03)	0.32(0.27,0.38)	4.80(4.64,4.96)	0.41(0.34,0.48)
Solomon Islands	0.02(-0.00,0.04)	0.02(0.00,0.03)	2.64(2.29,2.98)	-0.01(-0.04,0.01)
State of Eritrea	0.39(0.36,0.42)	0.05(0.04,0.06)	3.22(3.10,3.35)	0.07(0.05,0.08)
State of Israel	0.60(0.47,0.74)	-0.13(-0.18,-0.08)	6.02(5.00,7.05)	-0.15(-0.22,-0.09)
State of Kuwait	0.71(0.17,1.24)	-0.03(-0.10,0.04)	2.78(1.24,4.36)	-0.08(-0.17,0.01)
State of Libya	1.87(1.66,2.07)	0.43(0.39,0.46)	3.90(3.28,4.53)	0.54(0.50,0.58)
State of Qatar	0.25(0.09,0.40)	0.13(0.05,0.20)	1.67(0.41,2.93)	0.11(0.03,0.19)
Sultanate of Oman	0.48(0.30,0.66)	0.01(-0.04,0.05)	4.36(2.73,6.02)	-0.01(-0.06,0.04)
Swiss Confederation	0.96(0.85,1.08)	0.25(0.22,0.28)	4.11(3.35,4.88)	0.33(0.30,0.37)
Syrian Arab Republic	0.63(0.48,0.78)	-0.01(-0.06,0.04)	3.31(2.81,3.82)	-0.04(-0.11,0.02)
Taiwan (Province of China)	1.41(1.23,1.59)	0.34(0.19,0.49)	5.06(4.22,5.90)	0.88(0.66,1.10)
Togolese Republic	-0.01(-0.03,0.01)	-0.02(-0.03,-0.00)	3.34(1.88,4.82)	-0.04(-0.05,-0.02)
Tokelau	0.15(0.12,0.18)	0.07(0.04,0.10)	2.71(2.37,3.06)	0.06(0.03,0.10)
Turkmenistan	1.85(1.67,2.03)	0.69(0.61,0.76)	10.67(9.61,11.74)	0.79(0.70,0.88)

Tuvalu	0.21(0.18,0.25)	0.13(0.10,0.15)	2.92(2.70,3.14)	0.14(0.11,0.17)
Ukraine	0.80(0.69,0.92)	0.49(0.38,0.60)	2.63(1.90,3.36)	0.48(0.36,0.59)
Union of the Comoros	0.32(0.29,0.36)	0.02(0.01,0.03)	3.50(3.17,3.84)	0.03(0.01,0.04)
United Arab Emirates	1.71(1.53,1.88)	0.75(0.69,0.82)	4.26(3.20,5.33)	1.20(1.13,1.27)
United Kingdom of Great Britain and Northern Ireland	1.16(1.07,1.25)	-0.07(-0.23,0.09)	6.98(6.29,7.68)	0.03(-0.13,0.19)
United Mexican States	1.05(0.66,1.44)	0.90(0.33,1.48)	4.06(3.48,4.64)	0.66(0.13,1.19)
United Republic of Tanzania	0.39(0.35,0.42)	0.07(0.06,0.08)	3.35(3.03,3.68)	0.08(0.07,0.09)
United States of America	3.95(3.23,4.68)	-0.02(-0.75,0.73)	11.32(10.50,12.16)	-0.05(-1.02,0.93)
United States Virgin Islands	1.48(1.27,1.70)	-0.18(-0.33,-0.03)	4.08(3.62,4.53)	-0.37(-0.64,-0.10)

Table S10. Prediction of 5 drug use disorders-related burden from 2022 to 2035 in the global

Cause	Val	Sd	Time	Group	Low_50	Up_50	Low_60	Up_60	Low_70	Up_70	Low_80	Up_80	Low_95	Up_95
Amphetamine	31.92352	0.027376	1990	ASR	31.90507	31.94197	31.90049	31.94654	31.89516	31.95188	31.88842	31.95861	31.86986	31.97717
Amphetamine	31.9911	0.027208	1991	ASR	31.97277	32.00944	31.96822	32.01399	31.96292	32.01929	31.95622	32.02599	31.93778	32.04443
Amphetamine	31.88932	0.027023	1992	ASR	31.87111	31.90753	31.86659	31.91205	31.86132	31.91732	31.85468	31.92396	31.83635	31.94228
Amphetamine	31.6409	0.026801	1993	ASR	31.62284	31.65897	31.61836	31.66344	31.61314	31.66867	31.60654	31.67526	31.58837	31.69343
Amphetamine	31.2683	0.026543	1994	ASR	31.25041	31.28619	31.24598	31.29063	31.24081	31.2958	31.23428	31.30233	31.21628	31.32033
Amphetamine	30.76157	0.026232	1995	ASR	30.74389	30.77925	30.73951	30.78363	30.7344	30.78875	30.72794	30.7952	30.71016	30.81299
Amphetamine	30.11348	0.025859	1996	ASR	30.09605	30.13091	30.09173	30.13523	30.08669	30.14027	30.08033	30.14663	30.0628	30.16416
Amphetamine	29.38493	0.025448	1997	ASR	29.36777	29.40208	29.36352	29.40633	29.35856	29.41129	29.3523	29.41755	29.33505	29.4348
Amphetamine	28.66283	0.025022	1998	ASR	28.64597	28.6797	28.64179	28.68388	28.63691	28.68876	28.63076	28.69491	28.61379	28.71188
Amphetamine	28.02405	0.02461	1999	ASR	28.00746	28.04063	28.00335	28.04474	27.99855	28.04954	27.9925	28.0556	27.97581	28.07228
Amphetamine	27.51052	0.024231	2000	ASR	27.49419	27.52685	27.49014	27.5309	27.48542	27.53562	27.47946	27.54159	27.46303	27.55801
Amphetamine	26.99126	0.023835	2001	ASR	26.97519	27.00732	26.97121	27.0113	26.96656	27.01595	26.9607	27.02181	26.94454	27.03797
Amphetamine	26.34925	0.023377	2002	ASR	26.33349	26.36501	26.32959	26.36891	26.32503	26.37347	26.31928	26.37922	26.30343	26.39507
Amphetamine	25.65265	0.022892	2003	ASR	25.63722	25.66808	25.63339	25.6719	25.62893	25.67636	25.6233	25.68199	25.60778	25.69751
Amphetamine	24.9698	0.022412	2004	ASR	24.9547	24.98491	24.95096	24.98865	24.94659	24.99302	24.94107	24.99854	24.92588	25.01373
Amphetamine	24.35897	0.021968	2005	ASR	24.34416	24.37378	24.3405	24.37745	24.33621	24.38173	24.33081	24.38714	24.31591	24.40203
Amphetamine	23.73175	0.021525	2006	ASR	23.71724	23.74626	23.71364	23.74985	23.70945	23.75405	23.70415	23.75934	23.68956	23.77394
Amphetamine	23.02005	0.021056	2007	ASR	23.00586	23.03424	23.00234	23.03776	22.99823	23.04186	22.99306	23.04704	22.97878	23.06132
Amphetamine	22.32537	0.020605	2008	ASR	22.31148	22.33925	22.30804	22.34269	22.30402	22.34671	22.29895	22.35178	22.28498	22.36575
Amphetamine	21.75121	0.020227	2009	ASR	21.73758	21.76485	21.7342	21.76822	21.73026	21.77217	21.72528	21.77714	21.71157	21.79086
Amphetamine	21.37741	0.019967	2010	ASR	21.36395	21.39087	21.36062	21.3942	21.35672	21.39809	21.35181	21.40301	21.33827	21.41654
Amphetamine	21.09746	0.019775	2011	ASR	21.08414	21.11079	21.08083	21.11409	21.07698	21.11795	21.07211	21.12281	21.05871	21.13622
Amphetamine	20.78433	0.01958	2012	ASR	20.77113	20.79752	20.76786	20.80079	20.76404	20.80461	20.75922	20.80943	20.74595	20.8227
Amphetamine	20.45285	0.019387	2013	ASR	20.43978	20.46591	20.43654	20.46915	20.43276	20.47293	20.42799	20.4777	20.41485	20.49084
Amphetamine	20.11454	0.019201	2014	ASR	20.1016	20.12748	20.09839	20.13069	20.09465	20.13443	20.08992	20.13915	20.0769	20.15217
Amphetamine	19.79381	0.019026	2015	ASR	19.78099	19.80664	19.77781	19.80981	19.7741	19.81352	19.76942	19.8182	19.75652	19.8311
Amphetamine	19.48476	0.018857	2016	ASR	19.47205	19.49747	19.4689	19.50062	19.46523	19.5043	19.46059	19.50893	19.4478	19.52172
Amphetamine	19.17258	0.018682	2017	ASR	19.15999	19.18517	19.15687	19.18829	19.15323	19.19193	19.14863	19.19653	19.13596	19.2092
Amphetamine	18.87587	0.018511	2018	ASR	18.8634	18.88835	18.86031	18.89144	18.8567	18.89505	18.85214	18.89961	18.83959	18.91216
Amphetamine	18.62191	0.018357	2019	ASR	18.60954	18.63428	18.60647	18.63735	18.60289	18.64093	18.59838	18.64544	18.58593	18.65789

Cause	Val	Sd	Time	Group	Low_50	Up_50	Low_60	Up_60	Low_70	Up_70	Low_80	Up_80	Low_95	Up_95
Amphetamine	19.22683	0.018626	2020	ASR	19.21427	19.23938	19.21116	19.24249	19.20753	19.24612	19.20295	19.25071	19.19032	19.26333
Amphetamine	19.28327	0.01863	2021	ASR	19.27071	19.29583	19.2676	19.29894	19.26397	19.30257	19.25939	19.30715	19.24676	19.31978
Amphetamine	17.93519	0.365703	2022	ASR	17.68871	18.18167	17.62763	18.24275	17.55632	18.31406	17.46636	18.40402	17.21841	18.65197
Amphetamine	17.62528	0.408934	2023	ASR	17.34966	17.9009	17.28137	17.96919	17.20162	18.04893	17.10103	18.14953	16.82377	18.42679
Amphetamine	17.34508	0.457507	2024	ASR	17.03672	17.65344	16.96031	17.72984	16.8711	17.81905	16.75855	17.9316	16.44836	18.24179
Amphetamine	17.08954	0.51389	2025	ASR	16.74317	17.4359	16.65736	17.52172	16.55715	17.62193	16.43073	17.74834	16.08231	18.09676
Amphetamine	16.84793	0.580061	2026	ASR	16.45697	17.23889	16.3601	17.33576	16.24699	17.44887	16.10429	17.59157	15.71101	17.98485
Amphetamine	16.61453	0.657048	2027	ASR	16.17168	17.05739	16.06196	17.16711	15.93383	17.29524	15.7722	17.45687	15.32672	17.90235
Amphetamine	16.39239	0.744409	2028	ASR	15.89066	16.89412	15.76634	17.01844	15.62118	17.1636	15.43806	17.34672	14.93335	17.85143
Amphetamine	16.18199	0.84173	2029	ASR	15.61466	16.74931	15.47409	16.88988	15.30995	17.05402	15.10289	17.26108	14.5322	17.83178
Amphetamine	15.98142	0.948668	2030	ASR	15.34202	16.62082	15.18359	16.77925	14.9986	16.96424	14.76523	17.19761	14.12203	17.84081
Amphetamine	15.78632	1.064809	2031	ASR	15.06864	16.504	14.89081	16.68182	14.68318	16.88946	14.42123	17.1514	13.69929	17.87334
Amphetamine	15.59448	1.189537	2032	ASR	14.79274	16.39623	14.59408	16.59489	14.36212	16.82685	14.0695	17.11947	13.26299	17.92598
Amphetamine	15.40752	1.321829	2033	ASR	14.51661	16.29844	14.29587	16.51918	14.03811	16.77694	13.71294	17.10211	12.81674	17.99831
Amphetamine	15.2259	1.460852	2034	ASR	14.24129	16.21052	13.99733	16.45448	13.71246	16.73935	13.35309	17.09872	12.36263	18.08917
Amphetamine	15.04902	1.605935	2035	ASR	13.96662	16.13142	13.69843	16.39962	13.38528	16.71277	12.99022	17.10783	11.90139	18.19666
Cannabis	63.49546	0.040803	1990	ASR	63.46796	63.52296	63.46115	63.52978	63.45319	63.53773	63.44315	63.54777	63.41549	63.57544
Cannabis	63.42707	0.040474	1991	ASR	63.39979	63.45435	63.39303	63.46111	63.38514	63.469	63.37518	63.47895	63.34774	63.5064
Cannabis	63.37807	0.040255	1992	ASR	63.35094	63.4052	63.34421	63.41192	63.33636	63.41977	63.32646	63.42967	63.29917	63.45697
Cannabis	63.33848	0.040048	1993	ASR	63.31149	63.36547	63.3048	63.37216	63.29699	63.37997	63.28714	63.38982	63.25998	63.41697
Cannabis	63.29101	0.039833	1994	ASR	63.26416	63.31786	63.25751	63.32451	63.24974	63.33228	63.23994	63.34207	63.21294	63.36908
Cannabis	63.21617	0.03959	1995	ASR	63.18948	63.24285	63.18287	63.24946	63.17515	63.25718	63.16541	63.26692	63.13857	63.29376
Cannabis	63.06431	0.03931	1996	ASR	63.03782	63.09081	63.03126	63.09737	63.02359	63.10504	63.01392	63.11471	62.98727	63.14136
Cannabis	62.80993	0.038984	1997	ASR	62.78365	62.8362	62.77714	62.84271	62.76954	62.85031	62.75995	62.8599	62.73352	62.88634
Cannabis	62.49966	0.038619	1998	ASR	62.47363	62.52569	62.46718	62.53214	62.45965	62.53967	62.45015	62.54917	62.42397	62.57536
Cannabis	62.16968	0.038227	1999	ASR	62.14391	62.19544	62.13753	62.20182	62.13007	62.20928	62.12067	62.21868	62.09475	62.2446
Cannabis	61.8798	0.037826	2000	ASR	61.8543	61.90529	61.84799	61.91161	61.84061	61.91899	61.8313	61.92829	61.80566	61.95394
Cannabis	61.63149	0.037424	2001	ASR	61.60627	61.65671	61.60002	61.66296	61.59272	61.67026	61.58351	61.67947	61.55814	61.70484
Cannabis	61.38399	0.037025	2002	ASR	61.35904	61.40895	61.35286	61.41513	61.34564	61.42235	61.33653	61.43146	61.31143	61.45656
Cannabis	61.16781	0.036649	2003	ASR	61.1431	61.19251	61.13698	61.19863	61.12984	61.20577	61.12082	61.21479	61.09597	61.23964
Cannabis	61.01298	0.036318	2004	ASR	60.9885	61.03746	60.98244	61.04352	60.97536	61.05061	60.96642	61.05954	60.9418	61.08416
Cannabis	60.9185	0.036035	2005	ASR	60.89421	60.94278	60.88819	60.9488	60.88116	60.95583	60.8723	60.96469	60.84787	60.98913

Cause	Val	Sd	Time	Group	Low_50	Up_50	Low_60	Up_60	Low_70	Up_70	Low_80	Up_80	Low_95	Up_95
Cannabis	60.96961	0.035839	2006	ASR	60.94546	60.99377	60.93947	60.99975	60.93248	61.00674	60.92367	61.01556	60.89937	61.03985
Cannabis	61.17807	0.035727	2007	ASR	61.15399	61.20215	61.14803	61.20812	61.14106	61.21509	61.13227	61.22388	61.10805	61.2481
Cannabis	61.4378	0.03566	2008	ASR	61.41376	61.46183	61.40781	61.46779	61.40085	61.47474	61.39208	61.48351	61.3679	61.50769
Cannabis	61.66081	0.035613	2009	ASR	61.63681	61.68481	61.63086	61.69076	61.62392	61.69771	61.61516	61.70647	61.59101	61.73061
Cannabis	61.75096	0.035561	2010	ASR	61.72699	61.77493	61.72105	61.78087	61.71412	61.7878	61.70537	61.79655	61.68126	61.82066
Cannabis	61.74778	0.0355	2011	ASR	61.72385	61.77171	61.71793	61.77764	61.711	61.78456	61.70227	61.79329	61.6782	61.81736
Cannabis	61.75278	0.035444	2012	ASR	61.72889	61.77667	61.72297	61.78259	61.71606	61.7895	61.70734	61.79822	61.68331	61.82225
Cannabis	61.76751	0.035395	2013	ASR	61.74365	61.79137	61.73774	61.79728	61.73084	61.80418	61.72213	61.81289	61.69814	61.83688
Cannabis	61.78694	0.035351	2014	ASR	61.76312	61.81077	61.75721	61.81667	61.75032	61.82357	61.74163	61.83226	61.71766	61.85623
Cannabis	61.79109	0.035298	2015	ASR	61.7673	61.81488	61.7614	61.82077	61.75452	61.82766	61.74583	61.83634	61.7219	61.86027
Cannabis	62.296	0.03538	2016	ASR	62.27216	62.31985	62.26625	62.32576	62.25935	62.33266	62.25065	62.34136	62.22666	62.36535
Cannabis	63.39541	0.035621	2017	ASR	63.3714	63.41942	63.36545	63.42537	63.35851	63.43231	63.34974	63.44108	63.32559	63.46523
Cannabis	64.46307	0.035841	2018	ASR	64.43891	64.48723	64.43293	64.49321	64.42594	64.5002	64.41712	64.50902	64.39282	64.53332
Cannabis	64.88553	0.035863	2019	ASR	64.86136	64.9097	64.85537	64.91569	64.84838	64.92269	64.83956	64.93151	64.81524	64.95582
Cannabis	62.66854	0.035123	2020	ASR	62.64487	62.69221	62.639	62.69808	62.63215	62.70493	62.62351	62.71357	62.5997	62.73738
Cannabis	62.3319	0.034944	2021	ASR	62.30835	62.35545	62.30251	62.36129	62.2957	62.3681	62.2871	62.3767	62.26341	62.40039
Cannabis	61.58562	0.6452	2022	ASR	61.15076	62.02049	61.04301	62.12823	60.91719	62.25405	60.75848	62.41277	60.32103	62.85021
Cannabis	61.17742	0.792669	2023	ASR	60.64316	61.71168	60.51078	61.84405	60.35621	61.99862	60.16121	62.19362	59.62379	62.73105
Cannabis	60.74164	0.949213	2024	ASR	60.10187	61.38141	59.94335	61.53992	59.75825	61.72502	59.52475	61.95853	58.88118	62.60209
Cannabis	60.27799	1.119734	2025	ASR	59.52329	61.03269	59.3363	61.21969	59.11795	61.43804	58.84249	61.71349	58.08331	62.47267
Cannabis	59.80678	1.308045	2026	ASR	58.92516	60.6884	58.70672	60.90685	58.45165	61.16192	58.12987	61.4837	57.24301	62.37055
Cannabis	59.33229	1.515914	2027	ASR	58.31057	60.35402	58.05741	60.60718	57.76181	60.90278	57.38889	61.2757	56.3611	62.30349
Cannabis	58.85246	1.741367	2028	ASR	57.67878	60.02614	57.38797	60.31695	57.0484	60.65652	56.62003	61.08489	55.43938	62.26554
Cannabis	58.35923	1.982994	2029	ASR	57.02269	59.69577	56.69153	60.02693	56.30485	60.41361	55.81703	60.90143	54.47256	62.2459
Cannabis	57.85013	2.239988	2030	ASR	56.34038	59.35988	55.9663	59.73396	55.5295	60.17075	54.97846	60.72179	53.45975	62.2405
Cannabis	57.33613	2.511983	2031	ASR	55.64306	59.02921	55.22356	59.44871	54.73372	59.93855	54.11577	60.55649	52.41265	62.25962
Cannabis	56.81943	2.79839	2032	ASR	54.93331	58.70554	54.46598	59.17287	53.9203	59.71856	53.23189	60.40696	51.33458	62.30427
Cannabis	56.29903	3.097679	2033	ASR	54.2112	58.38687	53.69389	58.90418	53.08984	59.50823	52.32781	60.27026	50.22758	62.37048
Cannabis	55.7687	3.40878	2034	ASR	53.47118	58.06622	52.90192	58.63549	52.23721	59.3002	51.39865	60.13876	49.08749	62.44991
Cannabis	55.22624	3.731073	2035	ASR	52.7115	57.74099	52.08841	58.36408	51.36085	59.09164	50.44301	60.00948	47.91334	62.53915
Cocaine	4.206629	0.010063	1990	ASR	4.199847	4.213411	4.198166	4.215092	4.196204	4.217054	4.193729	4.219529	4.186906	4.226352
Cocaine	4.184938	0.00993	1991	ASR	4.178245	4.191631	4.176586	4.193289	4.17465	4.195226	4.172207	4.197669	4.165474	4.204402

Cause	Val	Sd	Time	Group	Low_50	Up_50	Low_60	Up_60	Low_70	Up_70	Low_80	Up_80	Low_95	Up_95
Cocaine	4.185277	0.009895	1992	ASR	4.178608	4.191947	4.176956	4.193599	4.175026	4.195529	4.172592	4.197963	4.165883	4.204672
Cocaine	4.197987	0.009887	1993	ASR	4.191324	4.204651	4.189672	4.206302	4.187745	4.20823	4.185312	4.210662	4.178609	4.217365
Cocaine	4.215066	0.009879	1994	ASR	4.208408	4.221725	4.206758	4.223374	4.204832	4.225301	4.202402	4.227731	4.195704	4.234429
Cocaine	4.230503	0.009856	1995	ASR	4.22386	4.237147	4.222214	4.238793	4.220292	4.240715	4.217867	4.243139	4.211185	4.249822
Cocaine	4.252732	0.009832	1996	ASR	4.246106	4.259359	4.244464	4.261001	4.242547	4.262918	4.240128	4.265336	4.233463	4.272002
Cocaine	4.280715	0.009805	1997	ASR	4.274106	4.287323	4.272469	4.288961	4.270557	4.290872	4.268145	4.293284	4.261497	4.299932
Cocaine	4.303478	0.009762	1998	ASR	4.296898	4.310058	4.295268	4.311688	4.293365	4.313592	4.290963	4.315993	4.284344	4.322612
Cocaine	4.306566	0.009688	1999	ASR	4.300036	4.313096	4.298418	4.314714	4.296529	4.316603	4.294146	4.318986	4.287577	4.325555
Cocaine	4.281607	0.009575	2000	ASR	4.275154	4.28806	4.273555	4.289659	4.271687	4.291526	4.269332	4.293882	4.26284	4.300373
Cocaine	4.245907	0.009443	2001	ASR	4.239542	4.252272	4.237965	4.253849	4.236124	4.25569	4.233801	4.258013	4.227399	4.264415
Cocaine	4.217898	0.009322	2002	ASR	4.211615	4.224181	4.210058	4.225737	4.20824	4.227555	4.205947	4.229848	4.199627	4.236168
Cocaine	4.20016	0.009221	2003	ASR	4.193945	4.206375	4.192406	4.207915	4.190608	4.209713	4.188339	4.211981	4.182088	4.218233
Cocaine	4.194114	0.009146	2004	ASR	4.187949	4.200278	4.186422	4.201806	4.184638	4.203589	4.182388	4.205839	4.176187	4.21204
Cocaine	4.194248	0.009095	2005	ASR	4.188118	4.200378	4.186599	4.201897	4.184825	4.20367	4.182588	4.205908	4.176421	4.212074
Cocaine	4.207851	0.009079	2006	ASR	4.201731	4.21397	4.200215	4.215487	4.198444	4.217257	4.196211	4.219491	4.190055	4.225646
Cocaine	4.24048	0.009102	2007	ASR	4.234346	4.246615	4.232826	4.248135	4.231051	4.24991	4.228812	4.252149	4.222641	4.25832
Cocaine	4.277057	0.00914	2008	ASR	4.270897	4.283218	4.26937	4.284744	4.267588	4.286527	4.265339	4.288775	4.259142	4.294973
Cocaine	4.305302	0.009179	2009	ASR	4.299116	4.311489	4.297583	4.313022	4.295793	4.314811	4.293535	4.317069	4.287312	4.323293
Cocaine	4.313246	0.009201	2010	ASR	4.307044	4.319447	4.305508	4.320983	4.303713	4.322778	4.30145	4.325041	4.295212	4.331279
Cocaine	4.292187	0.009187	2011	ASR	4.285995	4.298379	4.284461	4.299913	4.28267	4.301704	4.28041	4.303964	4.274181	4.310193
Cocaine	4.247997	0.009139	2012	ASR	4.241837	4.254157	4.240311	4.255683	4.238529	4.257466	4.236281	4.259714	4.230084	4.26591
Cocaine	4.194744	0.009079	2013	ASR	4.188625	4.200863	4.187109	4.202379	4.185339	4.20415	4.183106	4.206383	4.17695	4.212538
Cocaine	4.144552	0.00902	2014	ASR	4.138473	4.150632	4.136967	4.152138	4.135208	4.153897	4.132989	4.156116	4.126874	4.162231
Cocaine	4.105656	0.00897	2015	ASR	4.099609	4.111702	4.098111	4.1132	4.096362	4.114949	4.094155	4.117156	4.088074	4.123237
Cocaine	4.068229	0.00892	2016	ASR	4.062217	4.074241	4.060728	4.07573	4.058988	4.07747	4.056794	4.079664	4.050747	4.085712
Cocaine	4.024635	0.008858	2017	ASR	4.018665	4.030605	4.017186	4.032084	4.015458	4.033811	4.01328	4.03599	4.007274	4.041996
Cocaine	3.98541	0.008796	2018	ASR	3.979482	3.991339	3.978013	3.992808	3.976298	3.994523	3.974134	3.996687	3.96817	4.00265
Cocaine	3.958948	0.008745	2019	ASR	3.953053	3.964842	3.951593	3.966303	3.949888	3.968008	3.947736	3.970159	3.941807	3.976089
Cocaine	3.858573	0.008616	2020	ASR	3.852766	3.86438	3.851327	3.865818	3.849647	3.867498	3.847528	3.869618	3.841686	3.875459
Cocaine	3.887734	0.008661	2021	ASR	3.881896	3.893571	3.88045	3.895017	3.878761	3.896706	3.87663	3.898837	3.870758	3.904709
Cocaine	3.90879	0.08003	2022	ASR	3.85485	3.962731	3.841485	3.976096	3.825879	3.991702	3.806192	4.011389	3.751931	4.06565
Cocaine	3.902557	0.095711	2023	ASR	3.838048	3.967066	3.822064	3.983049	3.8034	4.001713	3.779856	4.025258	3.714964	4.09015

Cause	Val	Sd	Time	Group	Low_50	Up_50	Low_60	Up_60	Low_70	Up_70	Low_80	Up_80	Low_95	Up_95
Cocaine	3.896135	0.114966	2024	ASR	3.818647	3.973622	3.799448	3.992821	3.77703	4.01524	3.748748	4.043521	3.670801	4.121468
Cocaine	3.889753	0.137535	2025	ASR	3.797054	3.982452	3.774086	4.00542	3.747266	4.03224	3.713432	4.066073	3.620183	4.159323
Cocaine	3.883934	0.163192	2026	ASR	3.773942	3.993925	3.746689	4.021178	3.714867	4.053	3.674722	4.093145	3.564078	4.203789
Cocaine	3.878542	0.191718	2027	ASR	3.749324	4.00776	3.717307	4.039777	3.679922	4.077162	3.632759	4.124325	3.502774	4.25431
Cocaine	3.873185	0.222828	2028	ASR	3.722999	4.023371	3.685786	4.060583	3.642335	4.104035	3.587519	4.158851	3.436441	4.309928
Cocaine	3.867744	0.256324	2029	ASR	3.694982	4.040507	3.652176	4.083313	3.602193	4.133296	3.539137	4.196352	3.365349	4.37014
Cocaine	3.862315	0.292073	2030	ASR	3.665458	4.059172	3.616682	4.107948	3.559728	4.164902	3.487878	4.236752	3.289853	4.434777
Cocaine	3.857164	0.329978	2031	ASR	3.634759	4.079569	3.579652	4.134675	3.515307	4.199021	3.434132	4.280196	3.210407	4.503921
Cocaine	3.852215	0.36995	2032	ASR	3.602869	4.101562	3.541087	4.163344	3.468947	4.235484	3.377939	4.326492	3.127113	4.577318
Cocaine	3.847264	0.411863	2033	ASR	3.569668	4.124859	3.500887	4.19364	3.420574	4.273953	3.319256	4.375271	3.040013	4.654514
Cocaine	3.842265	0.455619	2034	ASR	3.535177	4.149352	3.459089	4.225441	3.370243	4.314287	3.258161	4.426369	2.949251	4.735279
Cocaine	3.837299	0.501149	2035	ASR	3.499525	4.175073	3.415833	4.258765	3.318109	4.356489	3.194826	4.479772	2.855047	4.81955
Opioid	32.81558	0.029049	1990	ASR	32.796	32.83516	32.79115	32.84001	32.78548	32.84567	32.77834	32.85282	32.75864	32.87251
Opioid	33.31993	0.028966	1991	ASR	33.30041	33.33946	33.29557	33.3443	33.28993	33.34994	33.2828	33.35707	33.26316	33.37671
Opioid	33.78207	0.028931	1992	ASR	33.76257	33.80157	33.75774	33.8064	33.7521	33.81204	33.74498	33.81916	33.72536	33.83877
Opioid	34.17803	0.028885	1993	ASR	34.15856	34.1975	34.15374	34.20232	34.14811	34.20796	34.141	34.21506	34.12142	34.23465
Opioid	34.49509	0.028818	1994	ASR	34.47567	34.51452	34.47086	34.51933	34.46524	34.52495	34.45815	34.53204	34.43861	34.55158
Opioid	34.7179	0.02872	1995	ASR	34.69854	34.73726	34.69375	34.74205	34.68815	34.74766	34.68108	34.75472	34.66161	34.77419
Opioid	34.96179	0.028628	1996	ASR	34.9425	34.98109	34.93771	34.98587	34.93213	34.99145	34.92509	34.99849	34.90568	35.0179
Opioid	35.30827	0.028576	1997	ASR	35.28901	35.32753	35.28424	35.33231	35.27867	35.33788	35.27164	35.34491	35.25226	35.36428
Opioid	35.67982	0.028526	1998	ASR	35.66059	35.69905	35.65583	35.70381	35.65027	35.70937	35.64325	35.71639	35.62391	35.73573
Opioid	35.96763	0.028432	1999	ASR	35.94847	35.9868	35.94372	35.99154	35.93818	35.99709	35.93118	36.00408	35.91191	36.02336
Opioid	36.06245	0.028252	2000	ASR	36.04341	36.08149	36.03869	36.08621	36.03318	36.09172	36.02623	36.09867	36.00708	36.11783
Opioid	35.95387	0.027988	2001	ASR	35.93501	35.97273	35.93033	35.97741	35.92487	35.98286	35.91799	35.98975	35.89901	36.00873
Opioid	35.71887	0.027672	2002	ASR	35.70022	35.73752	35.6956	35.74214	35.6902	35.74754	35.6834	35.75435	35.66463	35.77311
Opioid	35.39646	0.027324	2003	ASR	35.37805	35.41488	35.37348	35.41944	35.36816	35.42477	35.36143	35.43149	35.34291	35.45002
Opioid	35.01789	0.026954	2004	ASR	34.99972	35.03606	34.99522	35.04056	34.98996	35.04581	34.98333	35.05244	34.96506	35.07072
Opioid	34.61896	0.02658	2005	ASR	34.60105	34.63688	34.59661	34.64132	34.59143	34.6465	34.58489	34.65304	34.56687	34.67106
Opioid	34.04045	0.026146	2006	ASR	34.02283	34.05807	34.01846	34.06244	34.01336	34.06754	34.00693	34.07397	33.9892	34.0917
Opioid	33.21412	0.02563	2007	ASR	33.19684	33.23139	33.19256	33.23567	33.18756	33.24067	33.18126	33.24698	33.16388	33.26435
Opioid	32.33777	0.025107	2008	ASR	32.32085	32.35469	32.31666	32.35889	32.31176	32.36378	32.30558	32.36996	32.28856	32.38698
Opioid	31.60874	0.024656	2009	ASR	31.59212	31.62535	31.588	31.62947	31.58319	31.63428	31.57713	31.64035	31.56041	31.65706

Cause	Val	Sd	Time	Group	Low_50	Up_50	Low_60	Up_60	Low_70	Up_70	Low_80	Up_80	Low_95	Up_95
Opioid	31.20197	0.024355	2010	ASR	31.18555	31.21838	31.18148	31.22245	31.17673	31.2272	31.17074	31.23319	31.15423	31.2497
Opioid	31.1413	0.024211	2011	ASR	31.12498	31.15762	31.12094	31.16166	31.11622	31.16638	31.11026	31.17234	31.09385	31.18875
Opioid	31.27517	0.024155	2012	ASR	31.25889	31.29145	31.25485	31.29548	31.25014	31.30019	31.2442	31.30613	31.22782	31.32251
Opioid	31.55132	0.024168	2013	ASR	31.53503	31.56761	31.531	31.57165	31.52628	31.57636	31.52034	31.58231	31.50395	31.59869
Opioid	31.912	0.024227	2014	ASR	31.89567	31.92833	31.89162	31.93237	31.8869	31.9371	31.88094	31.94306	31.86451	31.95948
Opioid	32.27248	0.024294	2015	ASR	32.25611	32.28886	32.25205	32.29291	32.24731	32.29765	32.24134	32.30363	32.22486	32.3201
Opioid	32.87865	0.02446	2016	ASR	32.86216	32.89513	32.85808	32.89922	32.85331	32.90399	32.84729	32.91	32.8307	32.92659
Opioid	33.78114	0.024737	2017	ASR	33.76447	33.79781	33.76034	33.80194	33.75551	33.80677	33.74943	33.81285	33.73265	33.82962
Opioid	34.5939	0.024978	2018	ASR	34.57706	34.61073	34.57289	34.6149	34.56802	34.61977	34.56187	34.62592	34.54494	34.64285
Opioid	34.92052	0.025034	2019	ASR	34.90364	34.93739	34.89946	34.94157	34.89458	34.94645	34.88842	34.95261	34.87145	34.96958
Opioid	34.21111	0.024709	2020	ASR	34.19445	34.22776	34.19033	34.23189	34.18551	34.23671	34.17943	34.24278	34.16268	34.25954
Opioid	34.47807	0.024748	2021	ASR	34.46139	34.49475	34.45726	34.49888	34.45243	34.50371	34.44634	34.5098	34.42956	34.52658
Opioid	34.86793	0.610996	2022	ASR	34.45612	35.27974	34.35408	35.38178	34.23494	35.50092	34.08463	35.65123	33.67038	36.06548
Opioid	35.23752	0.749389	2023	ASR	34.73243	35.74261	34.60728	35.86776	34.46115	36.01389	34.2768	36.19824	33.76872	36.70632
Opioid	35.62206	0.885951	2024	ASR	35.02493	36.21919	34.87698	36.36715	34.70422	36.53991	34.48627	36.75785	33.8856	37.35853
Opioid	36.02092	1.027429	2025	ASR	35.32843	36.7134	35.15685	36.88498	34.9565	37.08533	34.70375	37.33808	34.00716	38.03468
Opioid	36.44207	1.179632	2026	ASR	35.647	37.23714	35.45	37.43414	35.21997	37.66417	34.92978	37.95436	34.12999	38.75415
Opioid	36.88692	1.347258	2027	ASR	35.97887	37.79497	35.75387	38.01996	35.49116	38.28268	35.15973	38.6141	34.24629	39.52755
Opioid	37.34936	1.532679	2028	ASR	36.31633	38.38239	36.06038	38.63834	35.7615	38.93722	35.38447	39.31425	34.34531	40.35341
Opioid	37.82435	1.738277	2029	ASR	36.65275	38.99595	36.36246	39.28624	36.0235	39.62521	35.59588	40.05282	34.41733	41.23137
Opioid	38.312	1.966571	2030	ASR	36.98653	39.63747	36.65812	39.96589	36.27464	40.34937	35.79086	40.83315	34.45752	42.16648
Opioid	38.81783	2.220107	2031	ASR	37.32148	40.31418	36.95072	40.68494	36.5178	41.11786	35.97165	41.66401	34.46642	43.16924
Opioid	39.34275	2.500877	2032	ASR	37.65716	41.02834	37.23951	41.44599	36.75184	41.93366	36.13662	42.54887	34.44103	44.24447
Opioid	39.88299	2.809615	2033	ASR	37.98931	41.77667	37.5201	42.24587	36.97223	42.79375	36.28106	43.48491	34.37614	45.38983
Opioid	40.43568	3.147227	2034	ASR	38.31445	42.55691	37.78887	43.0825	37.17516	43.69621	36.40094	44.47043	34.26712	46.60425
Opioid	41.00136	3.51484	2035	ASR	38.63236	43.37036	38.04538	43.95734	37.35999	44.64274	36.49534	45.50739	34.11227	47.89045
Other drug	121.3405	0.058318	1990	ASR	121.3012	121.3798	121.2915	121.3896	121.2801	121.4009	121.2657	121.4153	121.2262	121.4548
Other drug	121.3143	0.057459	1991	ASR	121.2756	121.353	121.266	121.3626	121.2548	121.3738	121.2406	121.3879	121.2017	121.4269
Other drug	121.273	0.056784	1992	ASR	121.2348	121.3113	121.2253	121.3208	121.2142	121.3319	121.2002	121.3458	121.1617	121.3843
Other drug	121.209	0.056138	1993	ASR	121.1712	121.2468	121.1618	121.2562	121.1508	121.2672	121.137	121.281	121.099	121.319
Other drug	121.107	0.055515	1994	ASR	121.0696	121.1445	121.0604	121.1537	121.0495	121.1646	121.0359	121.1782	120.9982	121.2158
Other drug	120.9679	0.054913	1995	ASR	120.9309	121.0049	120.9217	121.0141	120.911	121.0248	120.8975	121.0383	120.8603	121.0755

Cause	Val	Sd	Time	Group	Low_50	Up_50	Low_60	Up_60	Low_70	Up_70	Low_80	Up_80	Low_95	Up_95
Other drug	120.8553	0.054336	1996	ASR	120.8186	120.8919	120.8096	120.901	120.799	120.9116	120.7856	120.9249	120.7488	120.9618
Other drug	120.7989	0.053784	1997	ASR	120.7626	120.8351	120.7536	120.8441	120.7431	120.8546	120.7299	120.8678	120.6934	120.9043
Other drug	120.7453	0.053237	1998	ASR	120.7095	120.7812	120.7006	120.7901	120.6902	120.8005	120.6771	120.8136	120.641	120.8497
Other drug	120.622	0.052695	1999	ASR	120.5865	120.6575	120.5777	120.6663	120.5674	120.6766	120.5545	120.6896	120.5187	120.7253
Other drug	120.3748	0.052139	2000	ASR	120.3396	120.4099	120.3309	120.4186	120.3207	120.4288	120.3079	120.4416	120.2726	120.477
Other drug	119.8837	0.051556	2001	ASR	119.849	119.9185	119.8404	119.9271	119.8303	119.9371	119.8176	119.9498	119.7827	119.9848
Other drug	119.1519	0.050943	2002	ASR	119.1176	119.1863	119.1091	119.1948	119.0992	119.2047	119.0866	119.2172	119.0521	119.2518
Other drug	118.3383	0.050318	2003	ASR	118.3044	118.3722	118.296	118.3806	118.2861	118.3904	118.2738	118.4028	118.2397	118.4369
Other drug	117.5858	0.049725	2004	ASR	117.5523	117.6193	117.544	117.6276	117.5343	117.6373	117.5221	117.6496	117.4884	117.6833
Other drug	117.0361	0.049178	2005	ASR	117.0029	117.0692	116.9947	117.0774	116.9851	117.087	116.973	117.0991	116.9397	117.1325
Other drug	116.7494	0.048711	2006	ASR	116.7166	116.7823	116.7085	116.7904	116.699	116.7999	116.687	116.8119	116.6539	116.8449
Other drug	116.61	0.048291	2007	ASR	116.5775	116.6426	116.5694	116.6507	116.56	116.6601	116.5481	116.672	116.5154	116.7047
Other drug	116.5252	0.047885	2008	ASR	116.4929	116.5575	116.4849	116.5655	116.4756	116.5748	116.4638	116.5866	116.4313	116.619
Other drug	116.4005	0.047482	2009	ASR	116.3685	116.4325	116.3606	116.4404	116.3513	116.4497	116.3396	116.4614	116.3074	116.4936
Other drug	116.1547	0.047071	2010	ASR	116.1229	116.1864	116.1151	116.1943	116.1059	116.2034	116.0943	116.215	116.0624	116.2469
Other drug	115.8742	0.04668	2011	ASR	115.8428	115.9057	115.835	115.9135	115.8259	115.9226	115.8144	115.9341	115.7827	115.9657
Other drug	115.6512	0.046312	2012	ASR	115.62	115.6824	115.6122	115.6901	115.6032	115.6992	115.5918	115.7106	115.5604	115.742
Other drug	115.4478	0.045957	2013	ASR	115.4169	115.4788	115.4092	115.4865	115.4002	115.4955	115.3889	115.5068	115.3578	115.5379
Other drug	115.2238	0.04561	2014	ASR	115.1931	115.2545	115.1854	115.2622	115.1765	115.271	115.1653	115.2823	115.1344	115.3132
Other drug	114.9272	0.045263	2015	ASR	114.8967	114.9577	114.8892	114.9653	114.8803	114.9741	114.8692	114.9853	114.8385	115.016
Other drug	114.5756	0.044921	2016	ASR	114.5453	114.6058	114.5378	114.6133	114.529	114.6221	114.518	114.6332	114.4875	114.6636
Other drug	114.2498	0.044604	2017	ASR	114.2197	114.2799	114.2123	114.2873	114.2036	114.296	114.1926	114.307	114.1624	114.3372
Other drug	113.9888	0.044315	2018	ASR	113.9589	114.0186	113.9515	114.026	113.9429	114.0347	113.932	114.0456	113.9019	114.0756
Other drug	113.8271	0.044057	2019	ASR	113.7974	113.8568	113.7901	113.8642	113.7815	113.8728	113.7707	113.8836	113.7408	113.9135
Other drug	114.0377	0.043894	2020	ASR	114.0081	114.0672	114.0007	114.0746	113.9922	114.0831	113.9814	114.0939	113.9516	114.1237
Other drug	114.0507	0.043789	2021	ASR	114.0212	114.0802	114.0139	114.0875	114.0053	114.096	113.9945	114.1068	113.9649	114.1365
Other drug	112.6403	0.579843	2022	ASR	112.2495	113.0311	112.1526	113.1279	112.0396	113.241	111.8969	113.3836	111.5038	113.7768
Other drug	112.088	0.690893	2023	ASR	111.6223	112.5537	111.507	112.669	111.3722	112.8038	111.2023	112.9737	110.7338	113.4421
Other drug	111.5392	0.788823	2024	ASR	111.0076	112.0709	110.8758	112.2026	110.722	112.3564	110.5279	112.5505	109.9931	113.0853
Other drug	110.985	0.878344	2025	ASR	110.393	111.577	110.2464	111.7237	110.0751	111.895	109.859	112.1111	109.2635	112.7066
Other drug	110.4098	0.96272	2026	ASR	109.7609	111.0586	109.6001	111.2194	109.4124	111.4071	109.1755	111.644	108.5228	112.2967
Other drug	109.8106	1.044294	2027	ASR	109.1067	110.5144	108.9323	110.6888	108.7287	110.8925	108.4718	111.1494	107.7638	111.8574

Cause	Val	Sd	Time	Group	Low_50	Up_50	Low_60	Up_60	Low_70	Up_70	Low_80	Up_80	Low_95	Up_95
Other drug	109.2107	1.122556	2028	ASR	108.4541	109.9674	108.2667	110.1548	108.0478	110.3737	107.7716	110.6499	107.0105	111.411
Other drug	108.6186	1.198344	2029	ASR	107.8109	109.4263	107.6108	109.6264	107.3771	109.8601	107.0824	110.1549	106.2699	110.9674
Other drug	108.0283	1.273296	2030	ASR	107.1701	108.8865	106.9574	109.0991	106.7091	109.3474	106.3959	109.6606	105.5326	110.5239
Other drug	107.4234	1.349528	2031	ASR	106.5138	108.333	106.2884	108.5583	106.0253	108.8215	105.6933	109.1535	104.7783	110.0685
Other drug	106.7999	1.429365	2032	ASR	105.8365	107.7633	105.5978	108.002	105.319	108.2807	104.9674	108.6323	103.9983	109.6014
Other drug	106.1743	1.513269	2033	ASR	105.1544	107.1943	104.9017	107.447	104.6066	107.7421	104.2343	108.1143	103.2083	109.1403
Other drug	105.5558	1.603006	2034	ASR	104.4754	106.6362	104.2077	106.9039	103.8951	107.2165	103.5007	107.6108	102.4139	108.6977
Other drug	104.9418	1.70128	2035	ASR	103.7951	106.0885	103.511	106.3726	103.1793	106.7043	102.7608	107.1228	101.6073	108.2763

Table S11. Decomposition analysis of change in DALYs, incidence and death

Location name	Cause	Measure	Overall Difference	Aging	Population	Epidemiological Change	Aging Percentage	Population Percentage	Epidemiological change Percentage
Andean Latin America	Amphetamine	DALYs	4284.44	1223.58	2127.87	932.99	28.56	49.67	21.78
Australasia	Amphetamine	DALYs	9707.46	-1969.05	7261.25	4415.25	-20.28	74.8	45.48
Caribbean	Amphetamine	DALYs	1540.88	-444.3	1309.19	675.99	-28.83	84.96	43.87
Central Asia	Amphetamine	DALYs	8300.42	-1198.03	6071.55	3426.89	-14.43	73.15	41.29
Central Europe	Amphetamine	DALYs	-291.38	-16958.1	10719.08	5947.62	5819.87	-3678.7	-2041.17
Central Latin America	Amphetamine	DALYs	10802.45	1286.05	6850.88	2665.53	11.91	63.42	24.68
Central Sub-Saharan Africa	Amphetamine	DALYs	4619.44	2550.8	1799.05	269.59	55.22	38.95	5.84
East Asia	Amphetamine	DALYs	-522601	-452031	341117.6	-411687	86.5	-65.27	78.78
Eastern Europe	Amphetamine	DALYs	6286.61	-45981.4	27747.96	24520.05	-731.42	441.38	390.04
Eastern Sub-Saharan Africa	Amphetamine	DALYs	13656.1	7434.31	5685.19	536.6	54.44	41.63	3.93
Global	Amphetamine	DALYs	-27990.8	-93807.5	673013	-607196	335.14	-2404.41	2169.27
High SDI	Amphetamine	DALYs	300432.7	-121633	154035.8	268030.3	-40.49	51.27	89.21
High-income Asia Pacific	Amphetamine	DALYs	-4686.27	-15034.4	10231.6	116.55	320.82	-218.33	-2.49
High-income North America	Amphetamine	DALYs	262988.6	-52436.7	84756.15	230669.2	-19.94	32.23	87.71
High-middle SDI	Amphetamine	DALYs	-142764	-171675	172158.2	-143247	120.25	-120.59	100.34
Low SDI	Amphetamine	DALYs	29190.52	15457.65	12295.56	1437.31	52.95	42.12	4.92
Low-middle SDI	Amphetamine	DALYs	52494.97	20266.32	38596.28	-6367.63	38.61	73.52	-12.13
Middle SDI	Amphetamine	DALYs	-267550	-115195	294141	-446496	43.06	-109.94	166.88
North Africa and Middle East	Amphetamine	DALYs	26517.75	9203.82	11534.51	5779.41	34.71	43.5	21.79
Oceania	Amphetamine	DALYs	1461.14	700.91	747.91	12.32	47.97	51.19	0.84
South Asia	Amphetamine	DALYs	26644.99	8948.81	12491.49	5204.7	33.59	46.88	19.53
Southeast Asia	Amphetamine	DALYs	59514.55	-5630.14	65352.28	-207.59	-9.46	109.81	-0.35
Southern Latin America	Amphetamine	DALYs	2378.26	-304.14	2275.53	406.86	-12.79	95.68	17.11
Southern Sub-Saharan Africa	Amphetamine	DALYs	5430.23	1510.31	4719.94	-800.02	27.81	86.92	-14.73
Tropical Latin America	Amphetamine	DALYs	15207.58	-1715.3	18598	-1675.12	-11.28	122.29	-11.02
Western Europe	Amphetamine	DALYs	26991.84	-51556.4	42983.91	35564.36	-191.01	159.25	131.76
Western Sub-Saharan Africa	Amphetamine	DALYs	13255.33	8041.08	5504.09	-289.83	60.66	41.52	-2.19
Andean Latin America	Cannabis	DALYs	2005.81	673.34	1463.22	-130.76	33.57	72.95	-6.52
Australasia	Cannabis	DALYs	-1745.7	-973.18	2654.67	-3427.19	55.75	-152.07	196.32
Caribbean	Cannabis	DALYs	1455.6	-1160.81	2308.79	307.62	-79.75	158.61	21.13

Central Asia	Cannabis	DALYs	1391.22	-662.13	1869.59	183.75	-47.59	134.39	13.21
Central Europe	Cannabis	DALYs	-3505.25	-7588.19	4450.71	-367.76	216.48	-126.97	10.49
Central Latin America	Cannabis	DALYs	10043.07	332.84	6176.73	3533.5	3.31	61.5	35.18
Central Sub-Saharan Africa	Cannabis	DALYs	4508	2612.73	1818.05	77.22	57.96	40.33	1.71
East Asia	Cannabis	DALYs	12077.19	-34861.5	30413.96	16524.69	-288.66	251.83	136.83
Eastern Europe	Cannabis	DALYs	-3735.2	-12216.9	7417.09	1064.6	327.07	-198.57	-28.5
Eastern Sub-Saharan Africa	Cannabis	DALYs	16970.66	9733.79	7571	-334.12	57.36	44.61	-1.97
Global	Cannabis	DALYs	161849.8	-36928.6	223698.5	-24920.1	-22.82	138.21	-15.4
High SDI	Cannabis	DALYs	1182.15	-63980	69181.73	-4019.56	-5412.18	5852.2	-340.02
High-income Asia Pacific	Cannabis	DALYs	-5474.92	-14334.3	9125.81	-266.43	261.82	-166.68	4.87
High-income North America	Cannabis	DALYs	8346.48	-21856.6	34671.83	-4468.76	-261.87	415.41	-53.54
High-middle SDI	Cannabis	DALYs	4430.73	-35600.1	35101.54	4929.26	-803.48	792.23	111.25
Low SDI	Cannabis	DALYs	39912.89	23042.09	18289.59	-1418.79	57.73	45.82	-3.55
Low-middle SDI	Cannabis	DALYs	57658.26	20923.13	40650.16	-3915.03	36.29	70.5	-6.79
Middle SDI	Cannabis	DALYs	58600.02	-21515.7	60866.8	19248.93	-36.72	103.87	32.85
North Africa and Middle East	Cannabis	DALYs	13532.6	4106.87	6987.32	2438.4	30.35	51.63	18.02
Oceania	Cannabis	DALYs	1029.92	476.02	540.51	13.4	46.22	52.48	1.3
South Asia	Cannabis	DALYs	70441.84	27901.35	44204.3	-1663.8	39.61	62.75	-2.36
Southeast Asia	Cannabis	DALYs	19939.52	-807.74	18426.94	2320.32	-4.05	92.41	11.64
Southern Latin America	Cannabis	DALYs	2869.28	-681.91	2196.89	1354.3	-23.77	76.57	47.2
Southern Sub-Saharan Africa	Cannabis	DALYs	3110.67	194.99	2156.94	758.74	6.27	69.34	24.39
Tropical Latin America	Cannabis	DALYs	4813.41	-1607.2	10116.29	-3695.68	-33.39	210.17	-76.78
Western Europe	Cannabis	DALYs	-7247.69	-31461.4	25491.74	-1278.04	434.09	-351.72	17.63
Western Sub-Saharan Africa	Cannabis	DALYs	11023.27	6424.94	4340.81	257.52	58.29	39.38	2.34
Andean Latin America	Cocaine	DALYs	8601.81	2292.75	3879.34	2429.72	26.65	45.1	28.25
Australasia	Cocaine	DALYs	3500.87	-582.12	3075.61	1007.38	-16.63	87.85	28.78
Caribbean	Cocaine	DALYs	4835.64	-1011.8	3661.33	2186.11	-20.92	75.72	45.21
Central Asia	Cocaine	DALYs	4706.39	117.35	2112.03	2477.02	2.49	44.88	52.63
Central Europe	Cocaine	DALYs	-2950.23	-4566.4	3458.36	-1842.2	154.78	-117.22	62.44
Central Latin America	Cocaine	DALYs	26650.17	4949.06	21216.83	484.28	18.57	79.61	1.82
Central Sub-Saharan Africa	Cocaine	DALYs	1753.73	910.11	625.2	218.42	51.9	35.65	12.45
East Asia	Cocaine	DALYs	-17350.6	-9079.98	12825.96	-21096.5	52.33	-73.92	121.59
Eastern Europe	Cocaine	DALYs	-12725.3	-17819	13311.82	-8218.12	140.03	-104.61	64.58

Eastern Sub-Saharan Africa	Cocaine	DALYs	4610.15	2843.6	2084.77	-318.21	61.68	45.22	-6.9
Global	Cocaine	DALYs	549832.9	32987.21	321702.9	195142.8	6	58.51	35.49
High SDI	Cocaine	DALYs	373352.2	-119658	185542.3	307467.5	-32.05	49.7	82.35
High-income Asia Pacific	Cocaine	DALYs	-2046.71	-12803.5	10858.69	-101.87	625.57	-530.54	4.98
High-income North America	Cocaine	DALYs	355063.1	-72936.2	141673.1	286326.1	-20.54	39.9	80.64
High-middle SDI	Cocaine	DALYs	1097.98	-25474	41546.82	-14974.9	-2320.08	3783.94	-1363.86
Low SDI	Cocaine	DALYs	13670.1	8101.9	6494.03	-925.84	59.27	47.51	-6.77
Low-middle SDI	Cocaine	DALYs	62717.95	16853.25	26581.01	19283.69	26.87	42.38	30.75
Middle SDI	Cocaine	DALYs	98821.86	-6214.31	67043.61	37992.56	-6.29	67.84	38.45
North Africa and Middle East	Cocaine	DALYs	22507.23	13962.28	13211.79	-4666.83	62.03	58.7	-20.73
Oceania	Cocaine	DALYs	57.74	38.16	36.84	-17.27	66.1	63.81	-29.91
South Asia	Cocaine	DALYs	28651.13	12265.27	14992.86	1393	42.81	52.33	4.86
Southeast Asia	Cocaine	DALYs	3988.55	1077.21	2417.87	493.47	27.01	60.62	12.37
Southern Latin America	Cocaine	DALYs	8652.02	-917.79	7868.67	1701.13	-10.61	90.95	19.66
Southern Sub-Saharan Africa	Cocaine	DALYs	6442.02	2168.7	4003	270.32	33.66	62.14	4.2
Tropical Latin America	Cocaine	DALYs	84188.92	1357.65	27029.38	55801.89	1.61	32.11	66.28
Western Europe	Cocaine	DALYs	17960.74	-35724	36623.4	17061.38	-198.9	203.91	94.99
Western Sub-Saharan Africa	Cocaine	DALYs	2735.52	1475.87	1077.8	181.85	53.95	39.4	6.65
Andean Latin America	Opioid	DALYs	17063.18	6833.28	8966.78	1263.11	40.05	52.55	7.4
Australasia	Opioid	DALYs	22962.29	-2581.58	20575.61	4968.26	-11.24	89.61	21.64
Caribbean	Opioid	DALYs	3048.12	-1043.78	7384	-3292.1	-34.24	242.25	-108
Central Asia	Opioid	DALYs	47533.38	614.17	35940.82	10978.38	1.29	75.61	23.1
Central Europe	Opioid	DALYs	7280.58	-36443.6	28866.57	14857.61	-500.56	396.49	204.07
Central Latin America	Opioid	DALYs	42424.48	14783.02	33475.69	-5834.23	34.85	78.91	-13.75
Central Sub-Saharan Africa	Opioid	DALYs	35055.06	18169.93	12386.17	4498.97	51.83	35.33	12.83
East Asia	Opioid	DALYs	-1071701	-205271	602997.8	-1469427	19.15	-56.27	137.11
Eastern Europe	Opioid	DALYs	56879.45	-342479	255662.8	143695.2	-602.11	449.48	252.63
Eastern Sub-Saharan Africa	Opioid	DALYs	115872.3	59360.92	43160.55	13350.85	51.23	37.25	11.52
Global	Opioid	DALYs	5803269	473235.2	3106157	2223877	8.15	53.52	38.32
High SDI	Opioid	DALYs	5292294	-955801	1436366	4811729	-18.06	27.14	90.92
High-income Asia Pacific	Opioid	DALYs	-2076.31	-35107.5	32261.15	770	1690.86	-1553.77	-37.08
High-income North America	Opioid	DALYs	4921835	-654163	1118175	4457824	-13.29	22.72	90.57
High-middle SDI	Opioid	DALYs	-182730	-277661	593179.8	-498249	151.95	-324.62	272.67

Low SDI	Opioid	DALYs	301769.8	150298.6	119780	31691.25	49.81	39.69	10.5
Low-middle SDI	Opioid	DALYs	595632.4	214728.3	314494.4	66409.69	36.05	52.8	11.15
Middle SDI	Opioid	DALYs	-205028	112322.6	727435.7	-1044786	-54.78	-354.8	509.58
North Africa and Middle East	Opioid	DALYs	481374.1	210463.4	221741.6	49169.11	43.72	46.06	10.21
Oceania	Opioid	DALYs	2434.41	1362.87	1272.04	-200.5	55.98	52.25	-8.24
South Asia	Opioid	DALYs	659130.6	250855.7	318776.5	89498.38	38.06	48.36	13.58
Southeast Asia	Opioid	DALYs	110108.1	25551.48	71806.08	12750.52	23.21	65.21	11.58
Southern Latin America	Opioid	DALYs	11999.79	-35.97	12155.88	-120.12	-0.3	101.3	-1
Southern Sub-Saharan Africa	Opioid	DALYs	15596.22	13034.46	25335.62	-22773.9	83.57	162.45	-146.02
Tropical Latin America	Opioid	DALYs	32473.15	7481.56	30588.85	-5597.25	23.04	94.2	-17.24
Western Europe	Opioid	DALYs	227287.4	-248946	260243.7	215989.4	-109.53	114.5	95.03
Western Sub-Saharan Africa	Opioid	DALYs	66688.64	40620.99	28176.36	-2108.71	60.91	42.25	-3.16
Andean Latin America	Other drug	DALYs	1863.06	817.86	1231.99	-186.79	43.9	66.13	-10.03
Australasia	Other drug	DALYs	32134.29	287.57	8766.27	23080.44	0.89	27.28	71.82
Caribbean	Other drug	DALYs	1378.28	18.86	638.36	721.06	1.37	46.32	52.32
Central Asia	Other drug	DALYs	4277.39	287.54	1804.18	2185.67	6.72	42.18	51.1
Central Europe	Other drug	DALYs	-594.98	-3818.32	3057.11	166.23	641.76	-513.82	-27.94
Central Latin America	Other drug	DALYs	8343.98	1514.67	4158.12	2671.18	18.15	49.83	32.01
Central Sub-Saharan Africa	Other drug	DALYs	908.12	502.04	327.89	78.18	55.28	36.11	8.61
East Asia	Other drug	DALYs	-278705	-69725.6	129835	-338815	25.02	-46.59	121.57
Eastern Europe	Other drug	DALYs	19040.92	-28833.9	21876.83	25998.01	-151.43	114.89	136.54
Eastern Sub-Saharan Africa	Other drug	DALYs	2441.56	1181.15	853.48	406.92	48.38	34.96	16.67
Global	Other drug	DALYs	164596.7	51096.21	312451.8	-198951	31.04	189.83	-120.87
High SDI	Other drug	DALYs	262560.7	-54118.7	91858.13	224821.2	-20.61	34.99	85.63
High-income Asia Pacific	Other drug	DALYs	82.88	-2078.02	2281.16	-120.26	-2507.38	2752.49	-145.11
High-income North America	Other drug	DALYs	181189.6	-26140.1	49370.87	157958.8	-14.43	27.25	87.18
High-middle SDI	Other drug	DALYs	-46321.1	-29582.4	67809.19	-84547.9	63.86	-146.39	182.53
Low SDI	Other drug	DALYs	16763.92	6707.78	5342.14	4714	40.01	31.87	28.12
Low-middle SDI	Other drug	DALYs	53243.95	15559.48	21324.68	16359.79	29.22	40.05	30.73
Middle SDI	Other drug	DALYs	-121819	11858.4	128390.5	-262068	-9.73	-105.39	215.13
North Africa and Middle East	Other drug	DALYs	61884.65	26672.79	25419.15	9792.71	43.1	41.08	15.82
Oceania	Other drug	DALYs	133.73	82.64	72.12	-21.02	61.8	53.93	-15.72
South Asia	Other drug	DALYs	49538.49	16157.91	18683.07	14697.51	32.62	37.71	29.67

Southeast Asia	Other drug	DALYs	9308.44	2696.15	5079.7	1532.59	28.96	54.57	16.46
Southern Latin America	Other drug	DALYs	851.65	78.01	622.89	150.75	9.16	73.14	17.7
Southern Sub-Saharan Africa	Other drug	DALYs	3595.45	1380.59	2766.26	-551.4	38.4	76.94	-15.34
Tropical Latin America	Other drug	DALYs	13811.5	694.77	3163.93	9952.8	5.03	22.91	72.06
Western Europe	Other drug	DALYs	50798.33	-28244.6	30270.97	48772	-55.6	59.59	96.01
Western Sub-Saharan Africa	Other drug	DALYs	2314.67	1192.52	819.97	302.18	51.52	35.42	13.05
Andean Latin America	Amphetamine	Death	23.34	2.07	6.02	15.25	8.86	25.79	65.35
Australasia	Amphetamine	Death	81.5	-4.39	17.81	68.09	-5.39	21.85	83.54
Caribbean	Amphetamine	Death	16.39	0.23	3.91	12.24	1.4	23.89	74.71
Central Asia	Amphetamine	Death	61.62	1.42	15.47	44.73	2.3	25.11	72.59
Central Europe	Amphetamine	Death	27.56	-26.11	18.49	35.18	-94.75	67.08	127.66
Central Latin America	Amphetamine	Death	90	9.5	25.67	54.83	10.55	28.52	60.93
Central Sub-Saharan Africa	Amphetamine	Death	5.22	1.47	1.32	2.43	28.24	25.21	46.55
East Asia	Amphetamine	Death	-1341.97	-813.14	1652.48	-2181.3	60.59	-123.14	162.55
Eastern Europe	Amphetamine	Death	308.95	-209.3	146.69	371.56	-67.75	47.48	120.27
Eastern Sub-Saharan Africa	Amphetamine	Death	21.73	6.72	6.37	8.65	30.91	29.3	39.78
Global	Amphetamine	Death	5027.48	255.95	3190.32	1581.21	5.09	63.46	31.45
High SDI	Amphetamine	Death	5160.55	-628.56	1223.76	4565.34	-12.18	23.71	88.47
High-income Asia Pacific	Amphetamine	Death	18.23	-5.56	6.39	17.4	-30.49	35.03	95.46
High-income North America	Amphetamine	Death	4562.94	-391.99	984.69	3970.25	-8.59	21.58	87.01
High-middle SDI	Amphetamine	Death	-25.34	-323.17	637.97	-340.14	1275.14	-2517.25	1342.11
Low SDI	Amphetamine	Death	41.42	16.64	18.29	6.48	40.18	44.16	15.66
Low-middle SDI	Amphetamine	Death	180.55	40.34	80.31	59.9	22.34	44.48	33.18
Middle SDI	Amphetamine	Death	-331.64	50.51	1322.64	-1704.79	-15.23	-398.82	514.05
North Africa and Middle East	Amphetamine	Death	194.46	58.43	63.22	72.81	30.05	32.51	37.44
Oceania	Amphetamine	Death	0.68	0.23	0.24	0.21	33.67	35.46	30.87
South Asia	Amphetamine	Death	216.16	50.84	82.85	82.47	23.52	38.33	38.15
Southeast Asia	Amphetamine	Death	203.35	16.39	57.5	129.46	8.06	28.28	63.66
Southern Latin America	Amphetamine	Death	8.64	0.06	1.98	6.61	0.69	22.85	76.46
Southern Sub-Saharan Africa	Amphetamine	Death	35.3	6.1	12.59	16.61	17.28	35.66	47.06
Tropical Latin America	Amphetamine	Death	19.65	0.65	5.72	13.28	3.32	29.09	67.59
Western Europe	Amphetamine	Death	469.98	-161.31	160.79	470.5	-34.32	34.21	100.11
Western Sub-Saharan Africa	Amphetamine	Death	3.72	1.11	1	1.61	29.91	26.87	43.21

Andean Latin America	Cocaine	Death	120.09	29.79	47.82	42.48	24.81	39.82	35.37
Australasia	Cocaine	Death	11.47	-0.67	2.71	9.43	-5.87	23.64	82.23
Caribbean	Cocaine	Death	67.81	-1.67	28.24	41.24	-2.46	41.64	60.82
Central Asia	Cocaine	Death	74.76	3.4	27.5	43.86	4.54	36.79	58.67
Central Europe	Cocaine	Death	-42.83	-27.22	27.99	-43.6	63.55	-65.34	101.8
Central Latin America	Cocaine	Death	284.29	58.24	187.39	38.66	20.49	65.91	13.6
Central Sub-Saharan Africa	Cocaine	Death	12.94	5.82	5.15	1.97	44.95	39.8	15.24
East Asia	Cocaine	Death	-265.32	-36.01	173.26	-402.58	13.57	-65.3	151.73
Eastern Europe	Cocaine	Death	-261.06	-213.75	201.82	-249.12	81.88	-77.31	95.43
Eastern Sub-Saharan Africa	Cocaine	Death	46.36	29.46	27.7	-10.8	63.55	59.76	-23.3
Global	Cocaine	Death	9017.33	624.25	3308.2	5084.87	6.92	36.69	56.39
High SDI	Cocaine	Death	6910.48	-852.76	1852.36	5910.88	-12.34	26.81	85.53
High-income Asia Pacific	Cocaine	Death	20.41	-9.36	13.26	16.51	-45.84	64.97	80.87
High-income North America	Cocaine	Death	6645.94	-630.67	1637.89	5638.71	-9.49	24.65	84.84
High-middle SDI	Cocaine	Death	-163.59	-89.13	348.4	-422.85	54.49	-212.97	258.48
Low SDI	Cocaine	Death	138.92	83.38	91.85	-36.31	60.02	66.12	-26.14
Low-middle SDI	Cocaine	Death	866.32	209.82	375.69	280.81	24.22	43.37	32.41
Middle SDI	Cocaine	Death	1262.69	127.99	758.75	375.95	10.14	60.09	29.77
North Africa and Middle East	Cocaine	Death	327.16	216.7	220	-109.54	66.24	67.25	-33.48
Oceania	Cocaine	Death	0.68	0.44	0.51	-0.27	64.15	75.86	-40.01
South Asia	Cocaine	Death	614.92	233.35	322.41	59.16	37.95	52.43	9.62
Southeast Asia	Cocaine	Death	88.88	27.52	45.73	15.63	30.96	51.45	17.58
Southern Latin America	Cocaine	Death	17.48	0.82	5.18	11.48	4.69	29.63	65.68
Southern Sub-Saharan Africa	Cocaine	Death	45.58	17.4	32.69	-4.51	38.18	71.71	-9.89
Tropical Latin America	Cocaine	Death	953.48	30.31	218.14	705.03	3.18	22.88	73.94
Western Europe	Cocaine	Death	250.98	-152.59	176.21	227.37	-60.8	70.21	90.59
Western Sub-Saharan Africa	Cocaine	Death	3.3	2.04	1.84	-0.58	61.93	55.63	-17.55
Andean Latin America	Opioid	Death	56.33	19.85	17.02	19.46	35.24	30.22	34.54
Australasia	Opioid	Death	250.82	-3.13	171.52	82.43	-1.25	68.38	32.86
Caribbean	Opioid	Death	24.14	3.21	12.79	8.15	13.28	52.98	33.74
Central Asia	Opioid	Death	379.56	36.56	150.79	192.21	9.63	39.73	50.64
Central Europe	Opioid	Death	180.45	-253.24	272.7	160.99	-140.34	151.12	89.22
Central Latin America	Opioid	Death	113.72	65.31	69.42	-21.01	57.43	61.04	-18.47

Central Sub-Saharan Africa	Opioid	Death	249.44	124.82	83.44	41.19	50.04	33.45	16.51
East Asia	Opioid	Death	-13298.6	1104.72	5711.17	-20114.5	-8.31	-42.95	151.25
Eastern Europe	Opioid	Death	1564.82	-2378.78	2102.47	1841.14	-152.02	134.36	117.66
Eastern Sub-Saharan Africa	Opioid	Death	1235.32	597.65	413.35	224.32	48.38	33.46	18.16
Global	Opioid	Death	57988.92	10276.23	25862.9	21849.78	17.72	44.6	37.68
High SDI	Opioid	Death	58845.59	-5717.56	13576.51	50986.63	-9.72	23.07	86.64
High-income Asia Pacific	Opioid	Death	152.93	-82.55	86.02	149.46	-53.98	56.25	97.73
High-income North America	Opioid	Death	53655.76	-4059.09	10912.26	46802.59	-7.57	20.34	87.23
High-middle SDI	Opioid	Death	-1458.15	-487.35	4477.41	-5448.21	33.42	-307.06	373.64
Low SDI	Opioid	Death	2097.94	989.63	787.22	321.1	47.17	37.52	15.31
Low-middle SDI	Opioid	Death	4044.76	1697.87	1872.46	474.43	41.98	46.29	11.73
Middle SDI	Opioid	Death	-5554.8	3515.07	6065.96	-15135.8	-63.28	-109.2	272.48
North Africa and Middle East	Opioid	Death	2882.22	1566.37	1278.38	37.48	54.35	44.35	1.3
Oceania	Opioid	Death	4.97	5.44	4.88	-5.35	109.45	98.17	-107.62
South Asia	Opioid	Death	4580.76	2266.9	2137.77	176.08	49.49	46.67	3.84
Southeast Asia	Opioid	Death	925.51	306.76	451.83	166.93	33.14	48.82	18.04
Southern Latin America	Opioid	Death	84.41	6.65	22.39	55.37	7.88	26.53	65.59
Southern Sub-Saharan Africa	Opioid	Death	266.63	130.37	184.97	-48.71	48.9	69.37	-18.27
Tropical Latin America	Opioid	Death	75.33	13.8	15.91	45.62	18.32	21.12	60.56
Western Europe	Opioid	Death	4556.76	-1575.74	2497.51	3634.99	-34.58	54.81	79.77
Western Sub-Saharan Africa	Opioid	Death	47.64	44.66	37.34	-34.37	93.76	78.39	-72.14
Andean Latin America	Other drug	Death	32.99	12.4	22	-1.4	37.57	66.67	-4.24
Australasia	Other drug	Death	701.47	5.51	194.31	501.66	0.79	27.7	71.51
Caribbean	Other drug	Death	26.75	0.45	9.83	16.47	1.67	36.76	61.56
Central Asia	Other drug	Death	73.75	-0.03	31.37	42.41	-0.05	42.54	57.51
Central Europe	Other drug	Death	-4.34	-53.9	53.21	-3.65	1241.33	-1225.42	84.09
Central Latin America	Other drug	Death	133.03	14.42	65.32	53.28	10.84	49.1	40.05
Central Sub-Saharan Africa	Other drug	Death	3.3	1.21	1.08	1.01	36.77	32.65	30.58
East Asia	Other drug	Death	-4934.44	-794.27	2826.88	-6967.05	16.1	-57.29	141.19
Eastern Europe	Other drug	Death	370.05	-527.14	466.86	430.33	-142.45	126.16	116.29
Eastern Sub-Saharan Africa	Other drug	Death	16.46	5.53	5.15	5.78	33.6	31.29	35.11
Global	Other drug	Death	3469.71	748.93	6258.22	-3537.45	21.58	180.37	-101.95
High SDI	Other drug	Death	5105.65	-934.26	1776.92	4262.99	-18.3	34.8	83.5

High-income Asia Pacific	Other drug	Death	23.82	-3.77	23.51	4.09	-15.83	98.68	17.15
High-income North America	Other drug	Death	3461.64	-485.3	985.17	2961.76	-14.02	28.46	85.56
High-middle SDI	Other drug	Death	-821.24	-444.2	1382.86	-1759.9	54.09	-168.39	214.3
Low SDI	Other drug	Death	235.23	71.18	77.44	86.61	30.26	32.92	36.82
Low-middle SDI	Other drug	Death	951.68	227.37	383.62	340.69	23.89	40.31	35.8
Middle SDI	Other drug	Death	-2004.8	411.04	2718.18	-5134.01	-20.5	-135.58	256.09
North Africa and Middle East	Other drug	Death	1123.26	420.98	506.95	195.33	37.48	45.13	17.39
Oceania	Other drug	Death	0.74	0.51	0.64	-0.41	68.67	86.73	-55.4
South Asia	Other drug	Death	968.83	288.25	360.59	320	29.75	37.22	33.03
Southeast Asia	Other drug	Death	149.49	44.95	76.74	27.8	30.07	51.34	18.59
Southern Latin America	Other drug	Death	10.62	0.94	5.09	4.59	8.82	47.95	43.23
Southern Sub-Saharan Africa	Other drug	Death	75.66	19.83	57.45	-1.61	26.2	75.93	-2.13
Tropical Latin America	Other drug	Death	239.26	9.04	52.67	177.55	3.78	22.01	74.21
Western Europe	Other drug	Death	997.31	-433.64	536.3	894.66	-43.48	53.77	89.71
Western Sub-Saharan Africa	Other drug	Death	0.07	0.02	0.02	0.03	27.18	25.27	47.54
Andean Latin America	Amphetamine	Incidence	2962.86	717.02	1954.83	291.01	24.2	65.98	9.82
Australasia	Amphetamine	Incidence	2292.63	-2394.79	5301.7	-614.29	-104.46	231.25	-26.79
Caribbean	Amphetamine	Incidence	683.12	-623.17	1188.44	117.85	-91.22	173.97	17.25
Central Asia	Amphetamine	Incidence	3892.21	-2232.7	5368.31	756.6	-57.36	137.92	19.44
Central Europe	Amphetamine	Incidence	-4034.34	-16668.5	9456.86	3177.3	413.17	-234.41	-78.76
Central Latin America	Amphetamine	Incidence	5808.43	-148.14	5895.65	60.92	-2.55	101.5	1.05
Central Sub-Saharan Africa	Amphetamine	Incidence	4698	2708.69	1914.08	75.23	57.66	40.74	1.6
East Asia	Amphetamine	Incidence	-446869	-428707	238335.1	-256497	95.94	-53.33	57.4
Eastern Europe	Amphetamine	Incidence	-11880.4	-33774.9	19281.2	2613.27	284.29	-162.29	-22
Eastern Sub-Saharan Africa	Amphetamine	Incidence	13653.15	7675.86	5943.84	33.44	56.22	43.53	0.24
Global	Amphetamine	Incidence	-295367	-160522	493211.3	-628056	54.35	-166.98	212.64
High SDI	Amphetamine	Incidence	40784.48	-100218	99564.11	41438.59	-245.73	244.12	101.6
High-income Asia Pacific	Amphetamine	Incidence	-6726.52	-16299.1	10116.36	-543.76	242.31	-150.4	8.08
High-income North America	Amphetamine	Incidence	45283.47	-31009	43630.11	32662.32	-68.48	96.35	72.13
High-middle SDI	Amphetamine	Incidence	-146255	-164897	129025.9	-110384	112.75	-88.22	75.47
Low SDI	Amphetamine	Incidence	29550.32	15971.52	12659.83	918.97	54.05	42.84	3.11
Low-middle SDI	Amphetamine	Incidence	40374.57	14707.57	35232.2	-9565.2	36.43	87.26	-23.69
Middle SDI	Amphetamine	Incidence	-259866	-154061	213088.2	-318894	59.28	-82	122.71

North Africa and Middle East	Amphetamine	Incidence	14877.37	4513.73	8848.17	1515.47	30.34	59.47	10.19
Oceania	Amphetamine	Incidence	1344.22	614.06	727.72	2.44	45.68	54.14	0.18
South Asia	Amphetamine	Incidence	17388.83	6515.76	9785.62	1087.45	37.47	56.28	6.25
Southeast Asia	Amphetamine	Incidence	35567.81	-16569.1	57004.84	-4867.96	-46.58	160.27	-13.69
Southern Latin America	Amphetamine	Incidence	1931.98	-613.45	2481.23	64.2	-31.75	128.43	3.32
Southern Sub-Saharan Africa	Amphetamine	Incidence	2756	109.37	3363.51	-716.89	3.97	122.04	-26.01
Tropical Latin America	Amphetamine	Incidence	9275.68	-5054.12	15482.16	-1152.36	-54.49	166.91	-12.42
Western Europe	Amphetamine	Incidence	-2612.96	-46012.9	35680.37	7719.59	1760.95	-1365.52	-295.43
Western Sub-Saharan Africa	Amphetamine	Incidence	14340.61	8806.75	5960.44	-426.59	61.41	41.56	-2.97
Andean Latin America	Cannabis	Incidence	9901.87	1716.36	8707.31	-521.8	17.33	87.94	-5.27
Australasia	Cannabis	Incidence	-8114.3	-6666.6	13121.92	-14569.6	82.16	-161.71	179.55
Caribbean	Cannabis	Incidence	5962.49	-8407.12	12513.1	1856.51	-141	209.86	31.14
Central Asia	Cannabis	Incidence	6098.63	-5709.08	10966.69	841.03	-93.61	179.82	13.79
Central Europe	Cannabis	Incidence	-25425.2	-45771.2	23923.39	-3577.43	180.02	-94.09	14.07
Central Latin America	Cannabis	Incidence	42139.68	-7570.68	34749.04	14961.32	-17.97	82.46	35.5
Central Sub-Saharan Africa	Cannabis	Incidence	29377.99	17292.75	12024	61.24	58.86	40.93	0.21
East Asia	Cannabis	Incidence	-624.3	-268948	179290.6	89033.14	43080.13	-28718.8	-14261.3
Eastern Europe	Cannabis	Incidence	-22087.2	-67272.6	39514.42	5670.96	304.58	-178.9	-25.68
Eastern Sub-Saharan Africa	Cannabis	Incidence	107495.2	60294.61	49182.2	-1981.62	56.09	45.75	-1.84
Global	Cannabis	Incidence	775811	-378020	1269592	-115761	-48.73	163.65	-14.92
High SDI	Cannabis	Incidence	-19024.7	-360040	349706.1	-8691.17	1892.48	-1838.17	45.68
High-income Asia Pacific	Cannabis	Incidence	-44456.7	-92776.4	49903.02	-1583.38	208.69	-112.25	3.56
High-income North America	Cannabis	Incidence	49673.11	-101358	170265.8	-19235	-204.05	342.77	-38.72
High-middle SDI	Cannabis	Incidence	-19882.1	-236306	195330.9	21092.58	1188.53	-982.44	-106.09
Low SDI	Cannabis	Incidence	254107.6	147534.5	118708.4	-12135.3	58.06	46.72	-4.78
Low-middle SDI	Cannabis	Incidence	321448.7	91591.75	254278	-24421.1	28.49	79.1	-7.6
Middle SDI	Cannabis	Incidence	238991.8	-209562	354545.5	94008.06	-87.69	148.35	39.34
North Africa and Middle East	Cannabis	Incidence	70704.1	15465.64	42859.86	12378.6	21.87	60.62	17.51
Oceania	Cannabis	Incidence	5595.68	2393.09	3123.76	78.82	42.77	55.82	1.41
South Asia	Cannabis	Incidence	398241.7	132640.5	274920.6	-9319.38	33.31	69.03	-2.34
Southeast Asia	Cannabis	Incidence	93179.43	-30681.3	110677.2	13183.45	-32.93	118.78	14.15
Southern Latin America	Cannabis	Incidence	11409.73	-6441.09	12352.25	5498.57	-56.45	108.26	48.19
Southern Sub-Saharan Africa	Cannabis	Incidence	14977.18	-1388.21	12518.97	3846.42	-9.27	83.59	25.68

Tropical Latin America	Cannabis	Incidence	10178.36	-26647.6	53595.34	-16769.4	-261.81	526.56	-164.76
Western Europe	Cannabis	Incidence	-52197.7	-159755	131034.3	-23477.2	306.06	-251.03	44.98
Western Sub-Saharan Africa	Cannabis	Incidence	73781.29	43473.77	28974.4	1333.12	58.92	39.27	1.81
Andean Latin America	Cocaine	Incidence	1199.19	-101.17	1148.43	151.92	-8.44	95.77	12.67
Australasia	Cocaine	Incidence	514	-579.27	1031.39	61.88	-112.7	200.66	12.04
Caribbean	Cocaine	Incidence	282.49	-993.97	1346.7	-70.24	-351.86	476.73	-24.86
Central Asia	Cocaine	Incidence	448.75	-346.14	666.96	127.93	-77.13	148.63	28.51
Central Europe	Cocaine	Incidence	-955.27	-2380.23	1264.73	160.23	249.17	-132.39	-16.77
Central Latin America	Cocaine	Incidence	3084.79	-1816.58	7028.72	-2127.35	-58.89	227.85	-68.96
Central Sub-Saharan Africa	Cocaine	Incidence	773.34	536.97	291.3	-54.94	69.44	37.67	-7.1
East Asia	Cocaine	Incidence	-4992.31	-7201.83	4459.63	-2250.11	144.26	-89.33	45.07
Eastern Europe	Cocaine	Incidence	-1492.56	-5296.2	2956.12	847.52	354.84	-198.06	-56.78
Eastern Sub-Saharan Africa	Cocaine	Incidence	1370	906.11	530.92	-67.03	66.14	38.75	-4.89
Global	Cocaine	Incidence	32645.23	-19198.2	79826.08	-27982.6	-58.81	244.53	-85.72
High SDI	Cocaine	Incidence	12493.28	-46940.6	39099.88	20333.97	-375.73	312.97	162.76
High-income Asia Pacific	Cocaine	Incidence	-4368.81	-7810.53	4062.12	-620.4	178.78	-92.98	14.2
High-income North America	Cocaine	Incidence	15585.3	-16786.3	24381.46	7990.19	-107.71	156.44	51.27
High-middle SDI	Cocaine	Incidence	-3160.66	-18542	13794.72	1586.63	586.65	-436.45	-50.2
Low SDI	Cocaine	Incidence	4598.35	3192.65	1939.57	-533.87	69.43	42.18	-11.61
Low-middle SDI	Cocaine	Incidence	10051.66	3661.67	6926.54	-536.55	36.43	68.91	-5.34
Middle SDI	Cocaine	Incidence	8663.15	-12801.1	19279.77	2184.51	-147.77	222.55	25.22
North Africa and Middle East	Cocaine	Incidence	4022.09	2070.16	3233.79	-1281.86	51.47	80.4	-31.87
Oceania	Cocaine	Incidence	20.27	9.77	12.07	-1.57	48.2	59.55	-7.75
South Asia	Cocaine	Incidence	3331.82	1743.29	2213.92	-625.38	52.32	66.45	-18.77
Southeast Asia	Cocaine	Incidence	255.34	-170.65	621.34	-195.36	-66.83	243.34	-76.51
Southern Latin America	Cocaine	Incidence	1493.84	-1612.22	3225.72	-119.66	-107.92	215.93	-8.01
Southern Sub-Saharan Africa	Cocaine	Incidence	1660.06	358.94	1467.68	-166.56	21.62	88.41	-10.03
Tropical Latin America	Cocaine	Incidence	9847.76	-4630.26	7435.32	7042.7	-47.02	75.5	71.52
Western Europe	Cocaine	Incidence	-1374.23	-16712.7	12234.34	3104.11	1216.15	-890.27	-225.88
Western Sub-Saharan Africa	Cocaine	Incidence	1939.38	938.56	663.67	337.15	48.39	34.22	17.38
Andean Latin America	Opioid	Incidence	5665.13	1772.13	3548.29	344.7	31.28	62.63	6.08
Australasia	Opioid	Incidence	3757.13	-1264.72	4296.75	725.1	-33.66	114.36	19.3
Caribbean	Opioid	Incidence	679.14	-1140.61	2840.97	-1021.22	-167.95	418.32	-150.37

Central Asia	Opioid	Incidence	9165.87	-3190.88	11774.55	582.19	-34.81	128.46	6.35
Central Europe	Opioid	Incidence	-14.89	-9865.75	6765.1	3085.76	66270.47	-45442.7	-20727.8
Central Latin America	Opioid	Incidence	13434.38	1777.19	13009.78	-1352.58	13.23	96.84	-10.07
Central Sub-Saharan Africa	Opioid	Incidence	10456.55	5535.71	3855.5	1065.34	52.94	36.87	10.19
East Asia	Opioid	Incidence	-174884	-108660	134290.2	-200515	62.13	-76.79	114.66
Eastern Europe	Opioid	Incidence	-23798.7	-92236.6	58787.52	9650.3	387.57	-247.02	-40.55
Eastern Sub-Saharan Africa	Opioid	Incidence	25367.94	14237.78	10976.73	153.43	56.13	43.27	0.6
Global	Opioid	Incidence	640974.6	-22823	628760.9	35036.67	-3.56	98.09	5.47
High SDI	Opioid	Incidence	402016.3	-160795	159544	403267.8	-40	39.69	100.31
High-income Asia Pacific	Opioid	Incidence	-4095.07	-13814.9	10436.57	-716.73	337.35	-254.86	17.5
High-income North America	Opioid	Incidence	369472.5	-71179.5	100715.4	339936.6	-19.27	27.26	92.01
High-middle SDI	Opioid	Incidence	-54897.2	-120460	145301.1	-79738.2	219.43	-264.68	145.25
Low SDI	Opioid	Incidence	94454.61	61992.19	40970.75	-8508.32	65.63	43.38	-9.01
Low-middle SDI	Opioid	Incidence	179959.3	55586.25	104861.4	19511.63	30.89	58.27	10.84
Middle SDI	Opioid	Incidence	19177.65	-11978.2	183607.9	-152452	-62.46	957.41	-794.95
North Africa and Middle East	Opioid	Incidence	123780.9	39367.86	67821.7	16591.29	31.8	54.79	13.4
Oceania	Opioid	Incidence	970.04	509.26	470.17	-9.4	52.5	48.47	-0.97
South Asia	Opioid	Incidence	203426	65338.42	102595.1	35492.43	32.12	50.43	17.45
Southeast Asia	Opioid	Incidence	25028.64	3472.51	22091.5	-535.37	13.87	88.26	-2.14
Southern Latin America	Opioid	Incidence	3429.7	-649.33	4156.86	-77.83	-18.93	121.2	-2.27
Southern Sub-Saharan Africa	Opioid	Incidence	2054.52	1668.11	7319.97	-6933.56	81.19	356.29	-337.48
Tropical Latin America	Opioid	Incidence	9713.42	-735.61	12490.22	-2041.19	-7.57	128.59	-21.01
Western Europe	Opioid	Incidence	6628.93	-41254	35271.7	12611.21	-622.33	532.09	190.24
Western Sub-Saharan Africa	Opioid	Incidence	30736.85	19089.39	13229.46	-1582	62.11	43.04	-5.15
Andean Latin America	Other drug	Incidence	28929.7	13938.03	13952.38	1039.29	48.18	48.23	3.59
Australasia	Other drug	Incidence	24964.03	-129.68	19677.83	5415.88	-0.52	78.82	21.69
Caribbean	Other drug	Incidence	12190.86	1278.2	12073.53	-1160.87	10.48	99.04	-9.52
Central Asia	Other drug	Incidence	33664.69	7712.65	24983.23	968.81	22.91	74.21	2.88
Central Europe	Other drug	Incidence	3951.65	-47947.1	43557.74	8341.01	-1213.34	1102.27	211.08
Central Latin America	Other drug	Incidence	93531.85	40464.67	54647	-1579.81	43.26	58.43	-1.69
Central Sub-Saharan Africa	Other drug	Incidence	42286.9	24719.03	16105.36	1462.51	58.46	38.09	3.46
East Asia	Other drug	Incidence	167285	-51008.5	546988.8	-328695	-30.49	326.98	-196.49
Eastern Europe	Other drug	Incidence	-3508.49	-117599	108001.5	6089.41	3351.85	-3078.29	-173.56

Eastern Sub-Saharan Africa	Other drug	Incidence	102410.3	55731.82	40460.61	6217.85	54.42	39.51	6.07
Global	Other drug	Incidence	2411842	1080780	2125914	-794851	44.81	88.14	-32.96
High SDI	Other drug	Incidence	415072.2	-269674	515482	169264.4	-64.97	124.19	40.78
High-income Asia Pacific	Other drug	Incidence	-3572.01	-69390.3	70714.84	-4896.57	1942.61	-1979.69	137.08
High-income North America	Other drug	Incidence	211902.9	-101433	188267.1	125068.3	-47.87	88.85	59.02
High-middle SDI	Other drug	Incidence	260071.4	-117420	478952.4	-101461	-45.15	184.16	-39.01
Low SDI	Other drug	Incidence	310249.8	158855.6	124520.6	26873.68	51.2	40.14	8.66
Low-middle SDI	Other drug	Incidence	707966.6	275112.4	334006.7	98847.51	38.86	47.18	13.96
Middle SDI	Other drug	Incidence	717074.7	230784.4	631802.2	-145512	32.18	88.11	-20.29
North Africa and Middle East	Other drug	Incidence	318195.1	167639.1	127479.5	23076.53	52.68	40.06	7.25
Oceania	Other drug	Incidence	5423.67	2981.45	2438.9	3.31	54.97	44.97	0.06
South Asia	Other drug	Incidence	735539.7	287694.3	318136.8	129708.6	39.11	43.25	17.63
Southeast Asia	Other drug	Incidence	236561.1	117318.4	136967.4	-17724.7	49.59	57.9	-7.49
Southern Latin America	Other drug	Incidence	24400.74	2468.06	21900.64	32.05	10.11	89.75	0.13
Southern Sub-Saharan Africa	Other drug	Incidence	32451.69	12198.69	16925.79	3327.21	37.59	52.16	10.25
Tropical Latin America	Other drug	Incidence	97879.04	27550.66	46217.64	24110.74	28.15	47.22	24.63
Western Europe	Other drug	Incidence	111591.1	-196156	237870	69876.96	-175.78	213.16	62.62
Western Sub-Saharan Africa	Other drug	Incidence	135763	72817.06	49976.1	12969.86	53.64	36.81	9.55

Figure S1. The EAPC of ASPR for 5 drug use disorders in global and 21 regions

Opioid

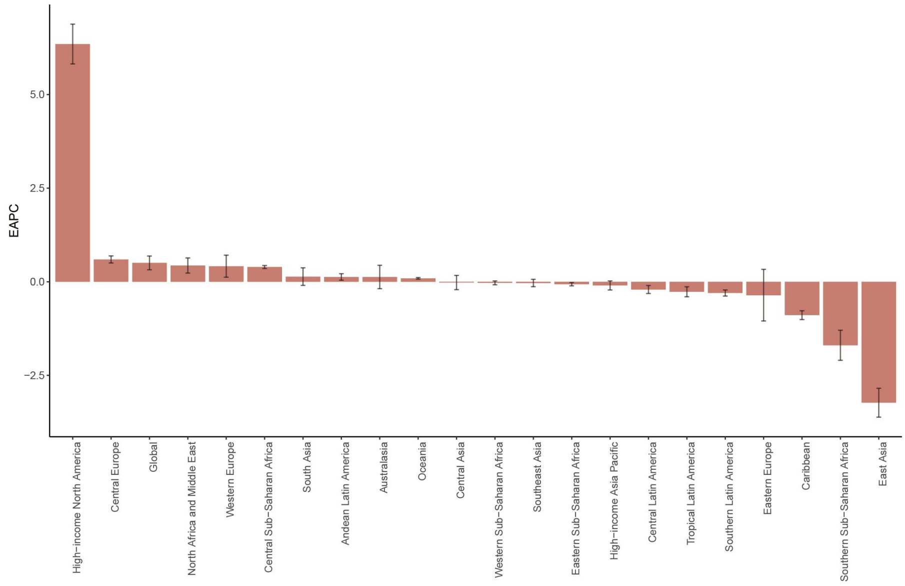

Other drug

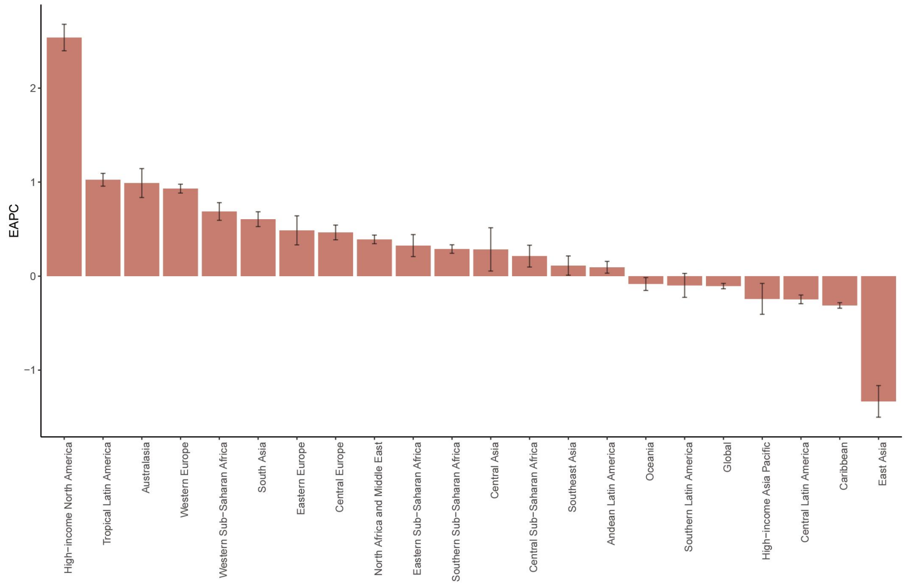

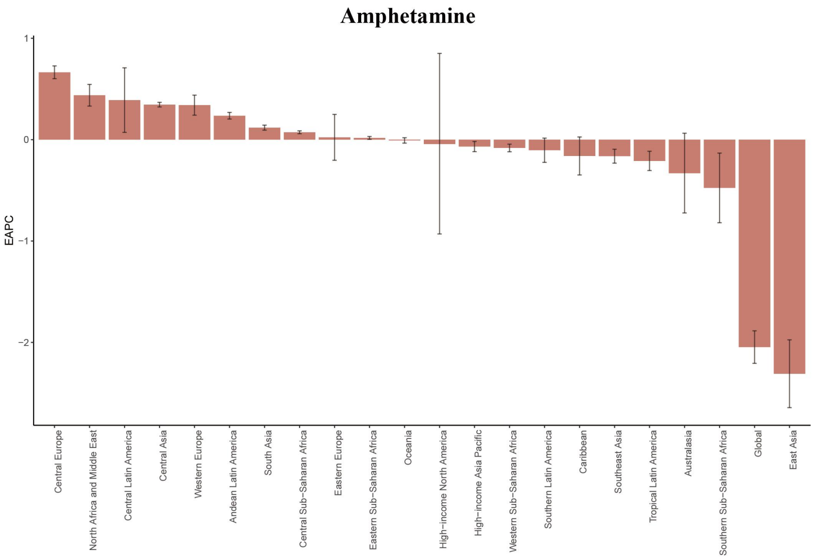
Amphetamine
EAPC
1
0
−1
−2
Central Europe
North Africa and Middle East
Central Latin America
Central Asia
Western Europe
Andean Latin America
South Asia
Central Sub−Saharan Africa
Eastern Europe
Eastern Sub−Saharan Africa
Oceania
High−income North America
High−income Asia Pacific
Western Sub−Saharan Africa
Southern Latin America
Caribbean
Southeast Asia
Tropical Latin America
Australasia
Southern Sub−Saharan Africa
Global
East Asia

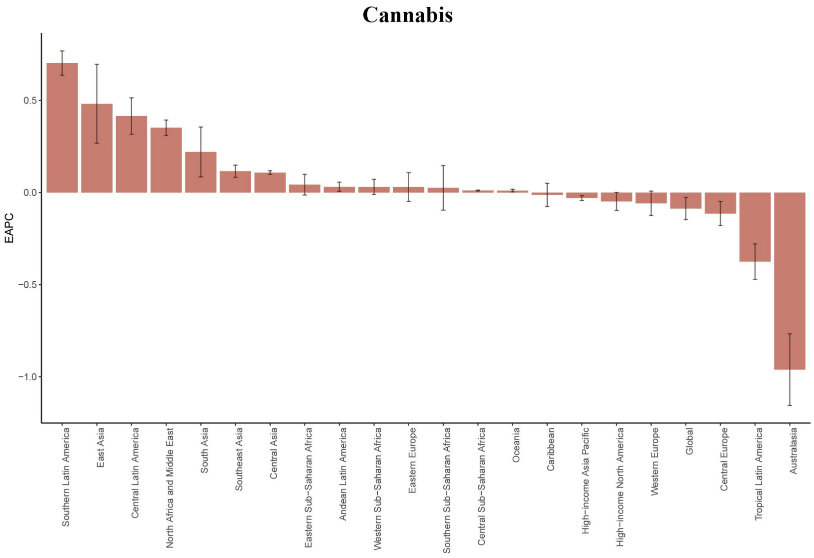
Cannabis
EAPC
0.5
0.0
−0.5
−1.0
Southern Latin America
East Asia
Central Latin America
North Africa and Middle East
South Asia
Southeast Asia
Central Asia
Eastern Sub−Saharan Africa
Andean Latin America
Western Sub−Saharan Africa
Eastern Europe
Southern Sub−Saharan Africa
Central Sub−Saharan Africa
Oceania
Caribbean
High−income Asia Pacific
High−income North America
Western Europe
Global
Central Europe
Tropical Latin America
Australasia

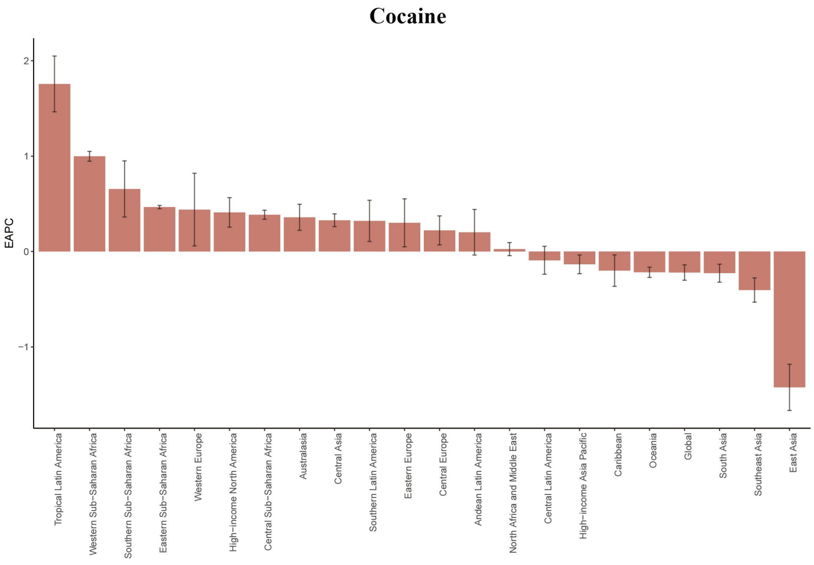
Cocaine
EAPC
2
1
0
−1
Tropical Latin America
Western Sub−Saharan Africa
Southern Sub−Saharan Africa
Eastern Sub−Saharan Africa
Western Europe
High−income North America
Central Sub−Saharan Africa
Australasia
Central Asia
Southern Latin America
Eastern Europe
Central Europe
Andean Latin America
North Africa and Middle East
Central Latin America
High−income Asia Pacific
Caribbean
Oceania
Global
South Asia
Southeast Asia
East Asia

Figure S2. The EAPC of ASMR for 5 drug use disorders in global and 21 regions

Amphetamine

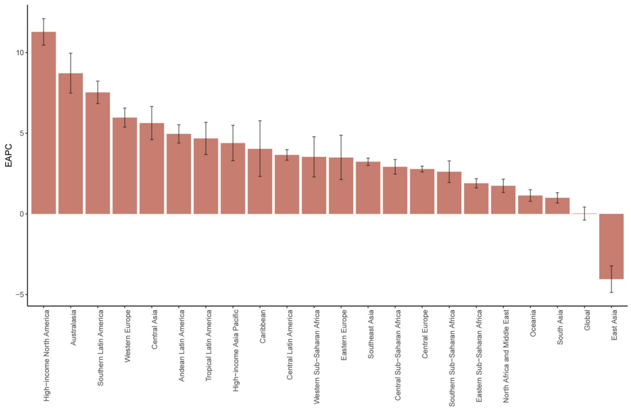

Cocaine

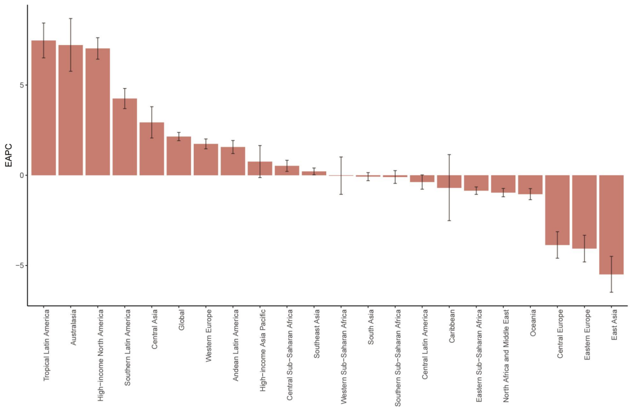

Opioid

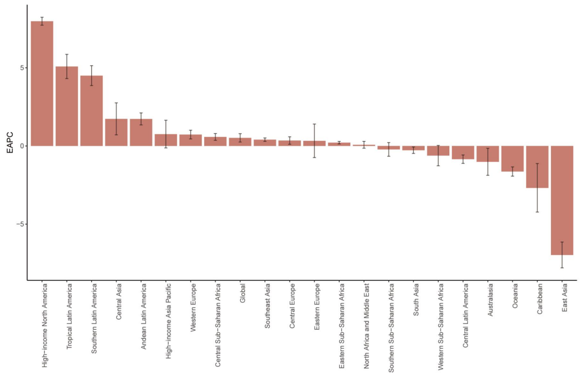

Other drug

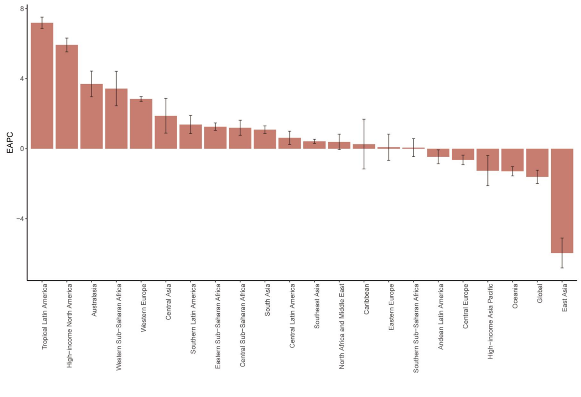

Figure S3. The EAPC of ASDR for 5 drug use disorders in global and 21 regions

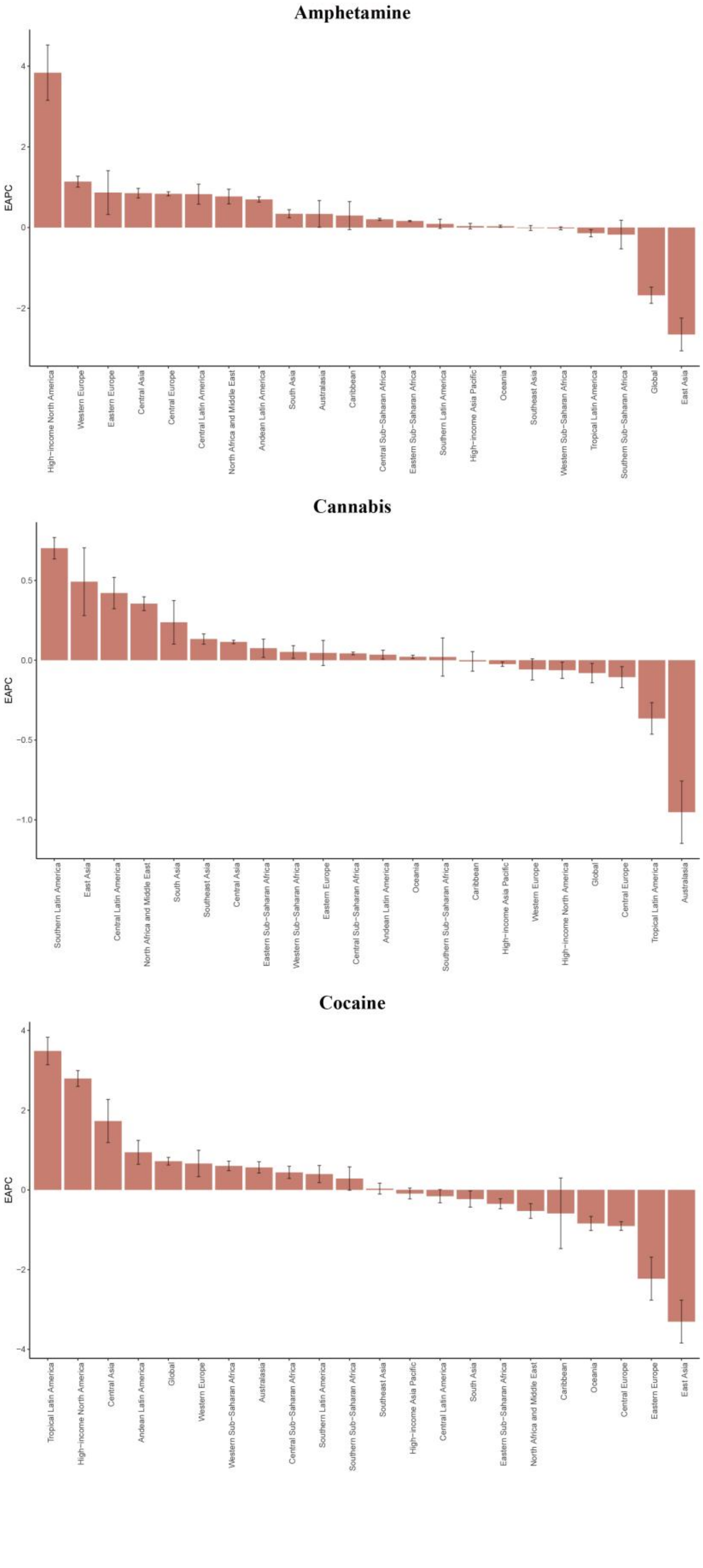

Opioid

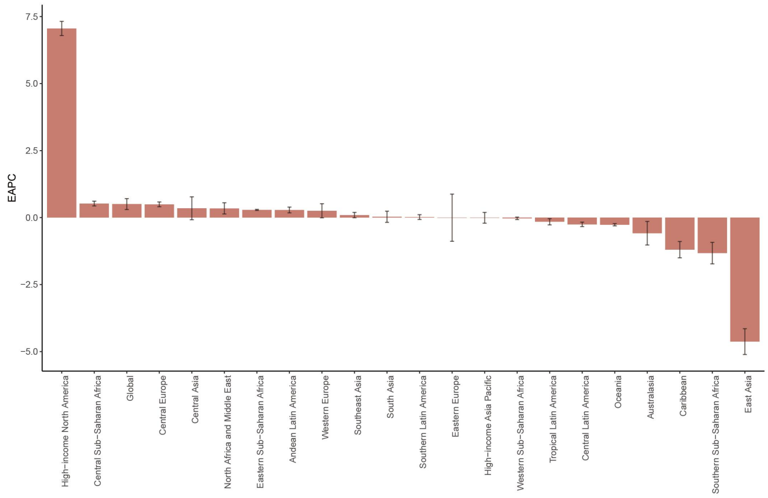

Other drug

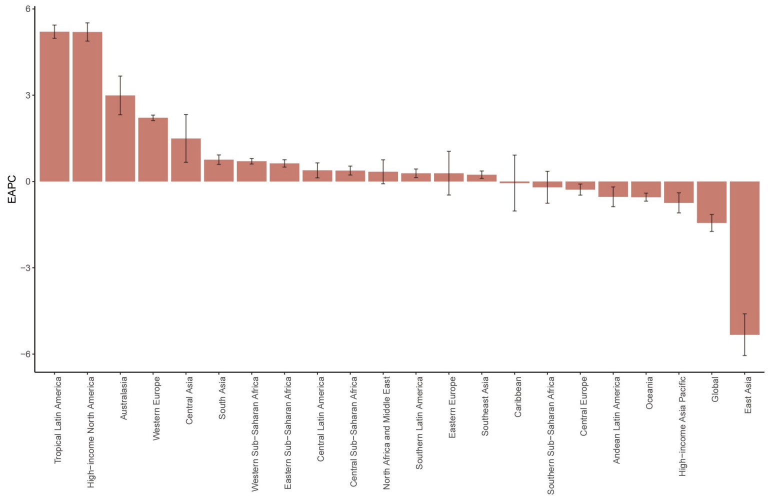

Figure S4. The global DALYs ,deaths and prevalence of 5 drug use disorders in 204 countries or territories in 2021

Amphetamine

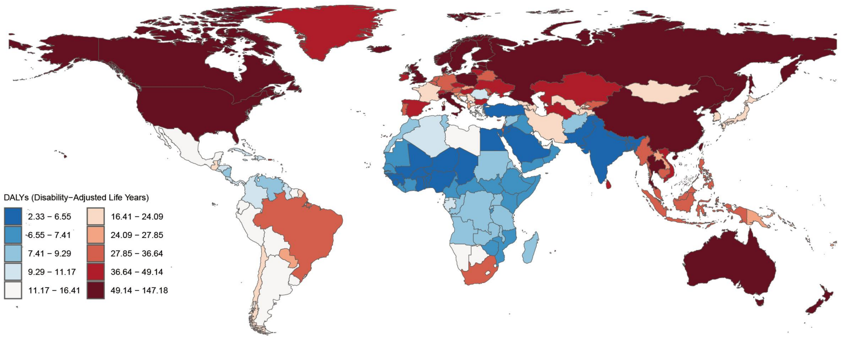

Cannabis

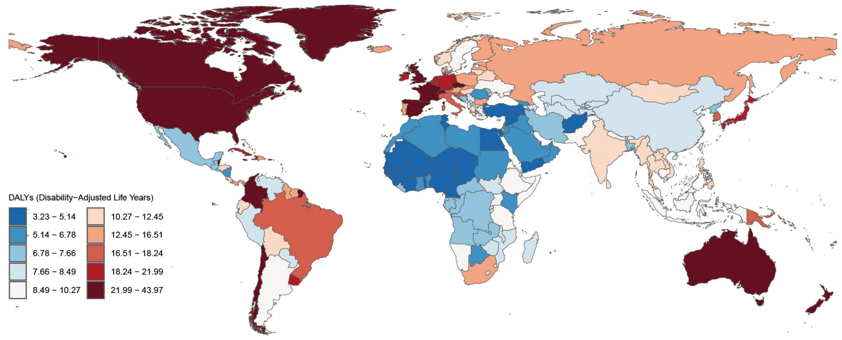

Cocaine

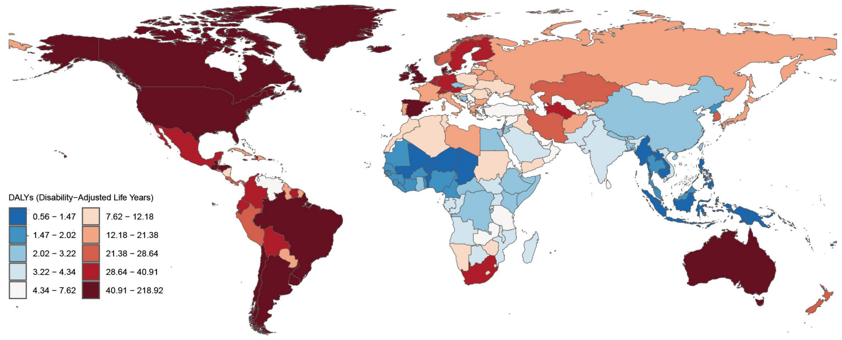

Opioid

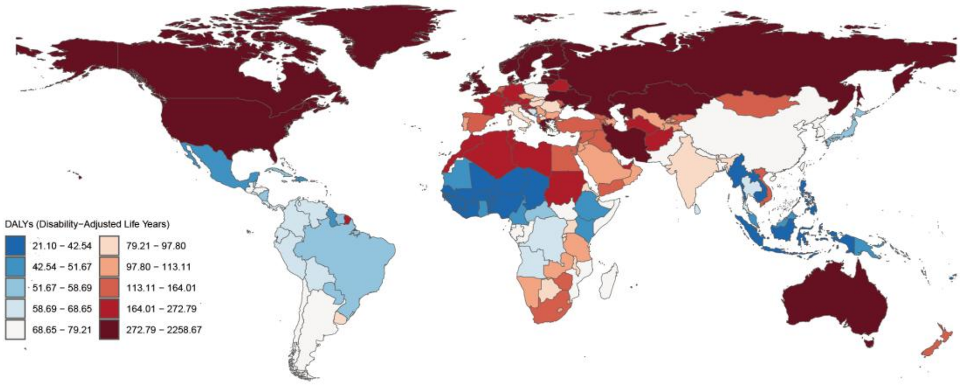

Other drug

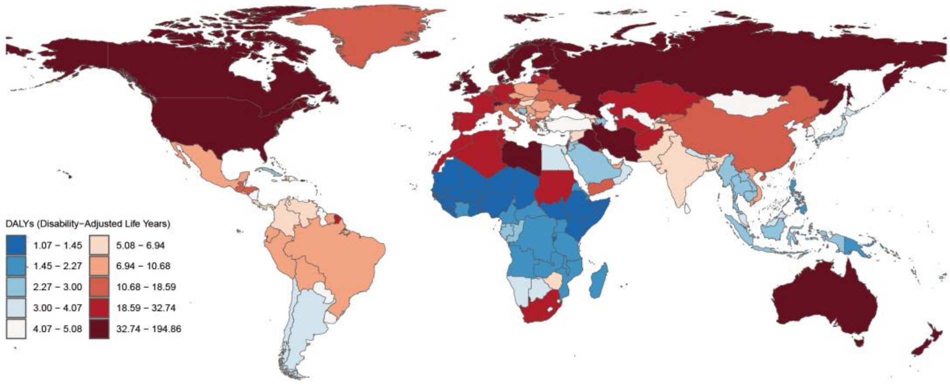

Amphetamine

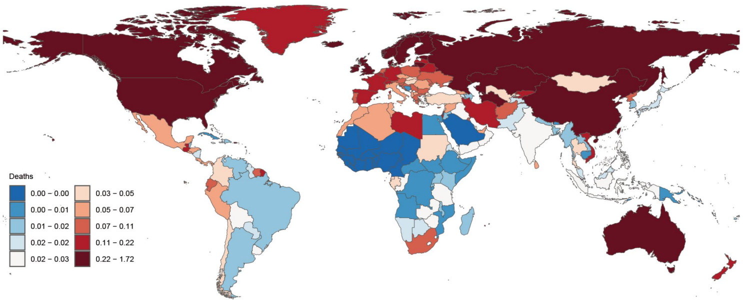

Cocaine

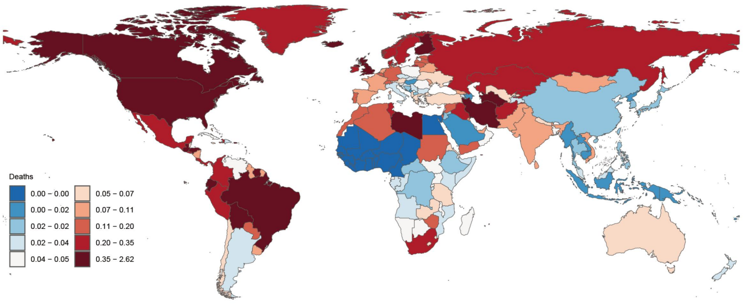

Opioid

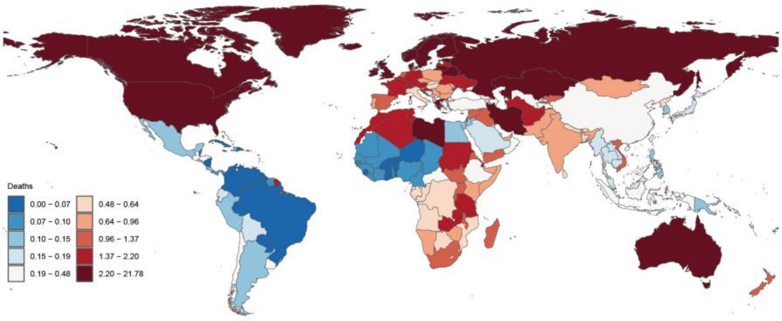

Other drug

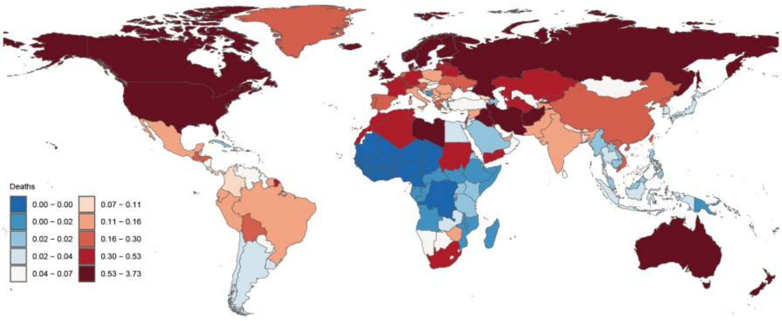

Amphetamine

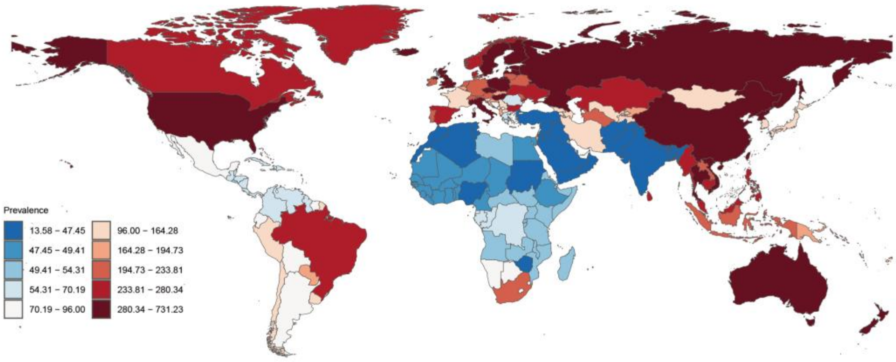

Cannabis

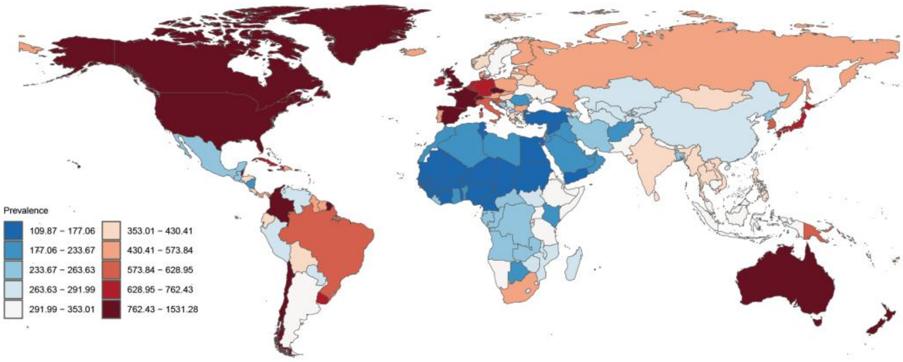

Cocaine

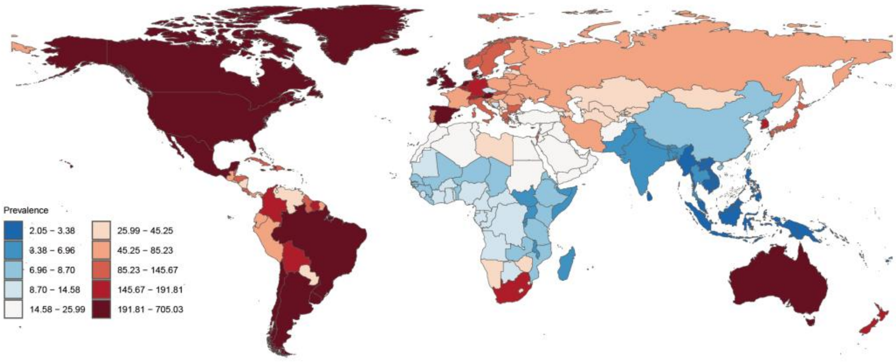

Opioid

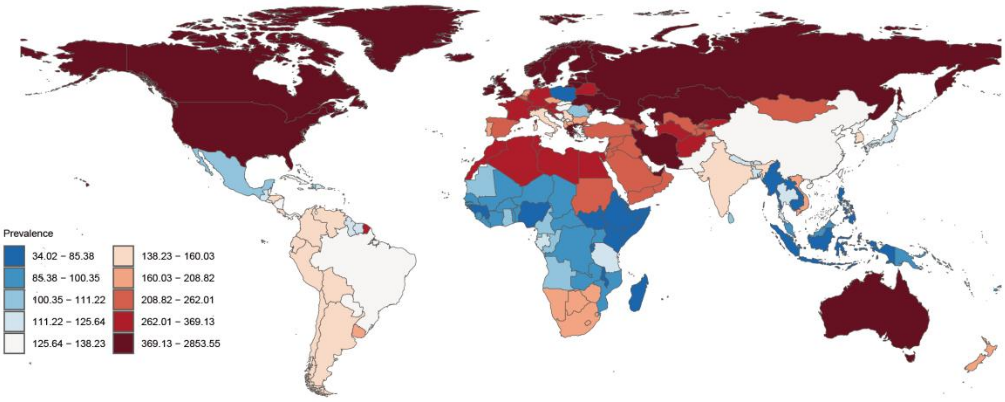

Other drug

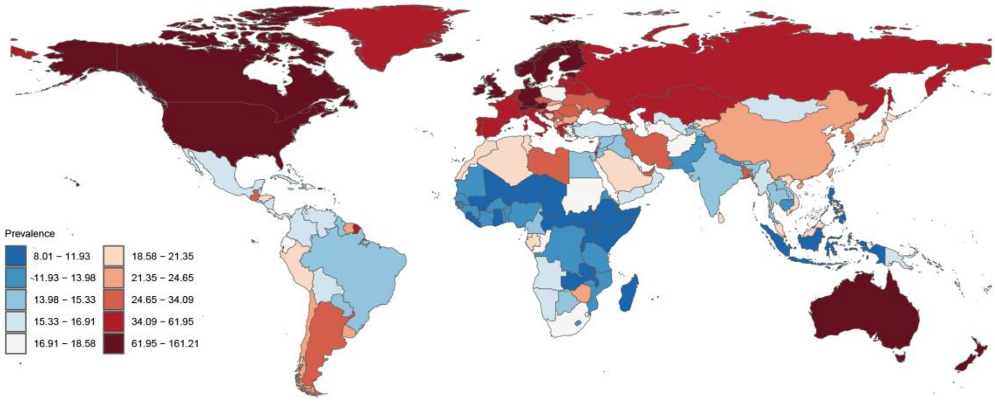

Figure S5. ASPR of 5 drug use disorders for 204 countries and territories by SDI

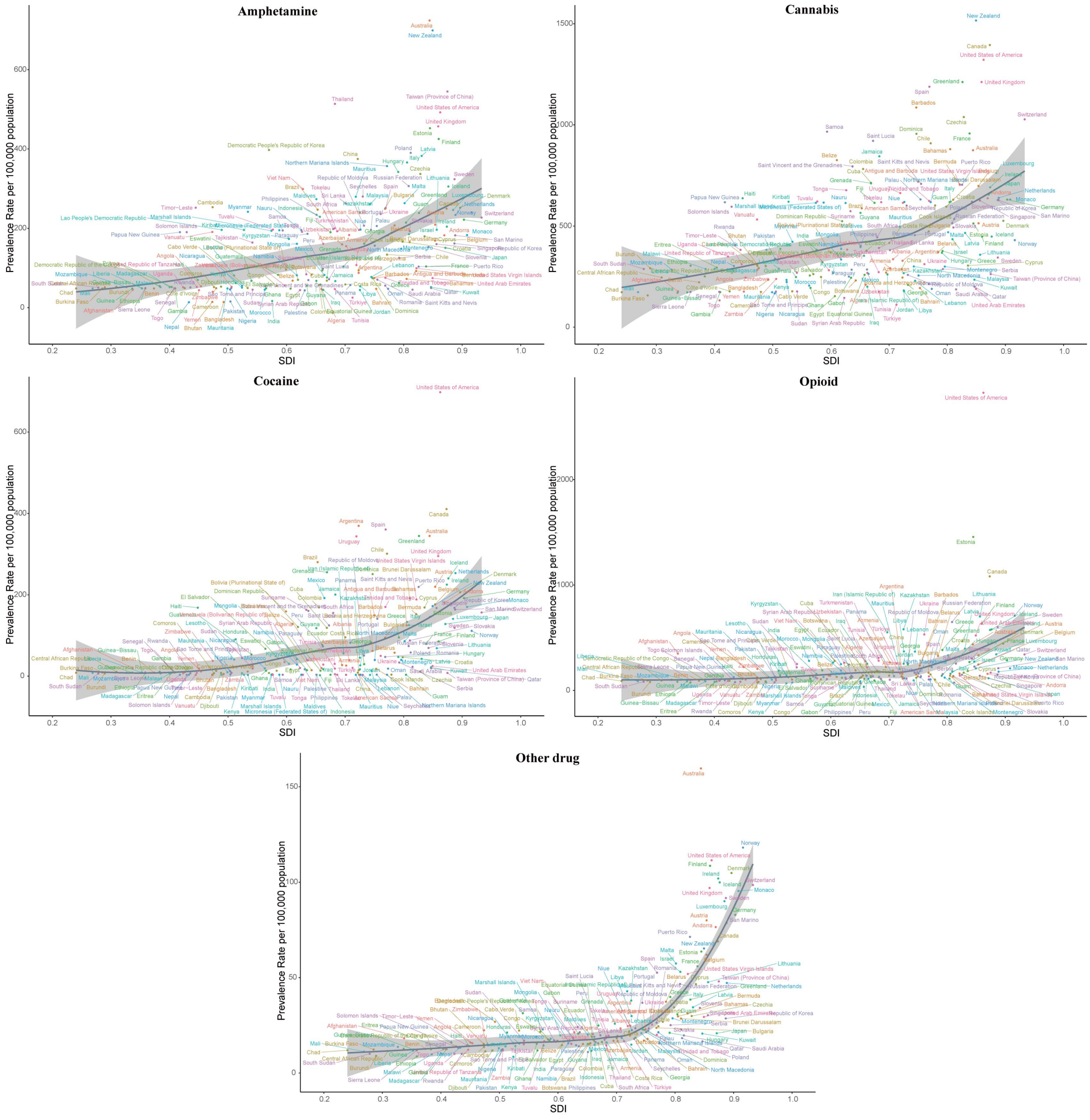

Figure S6. ASMR of 5 drug use disorders for 204 countries and territories by SDI

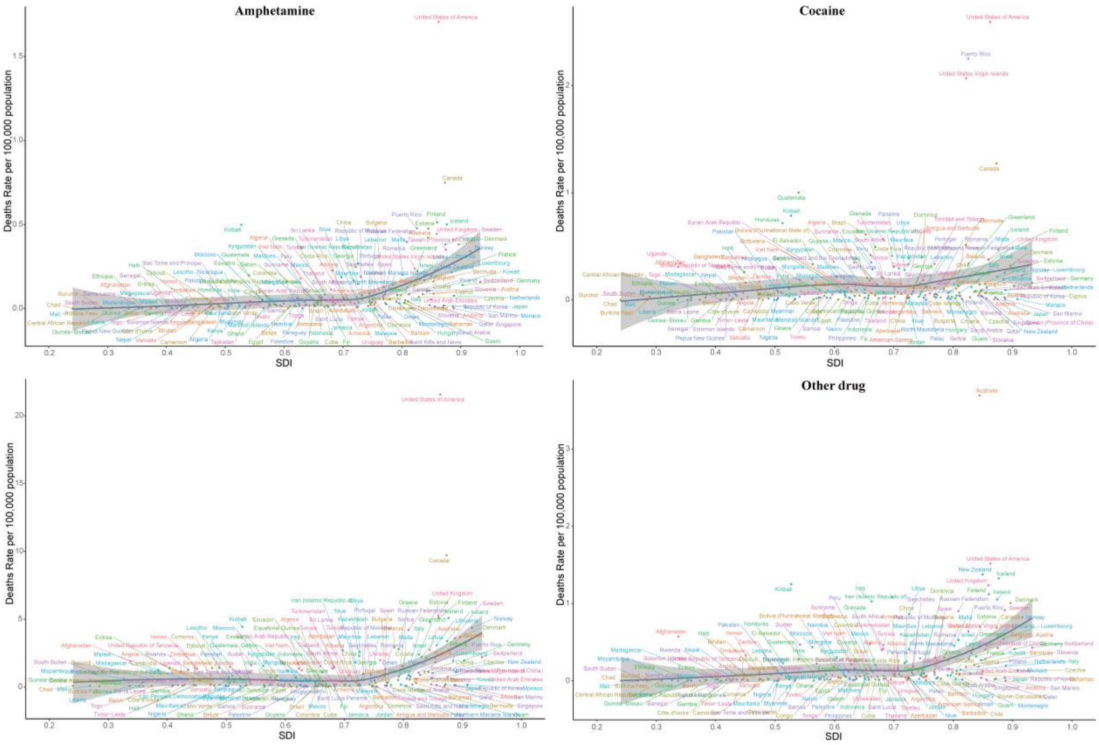

Figure S7. ASDR of 5 drug use disorders for 204 countries and territories by SDI

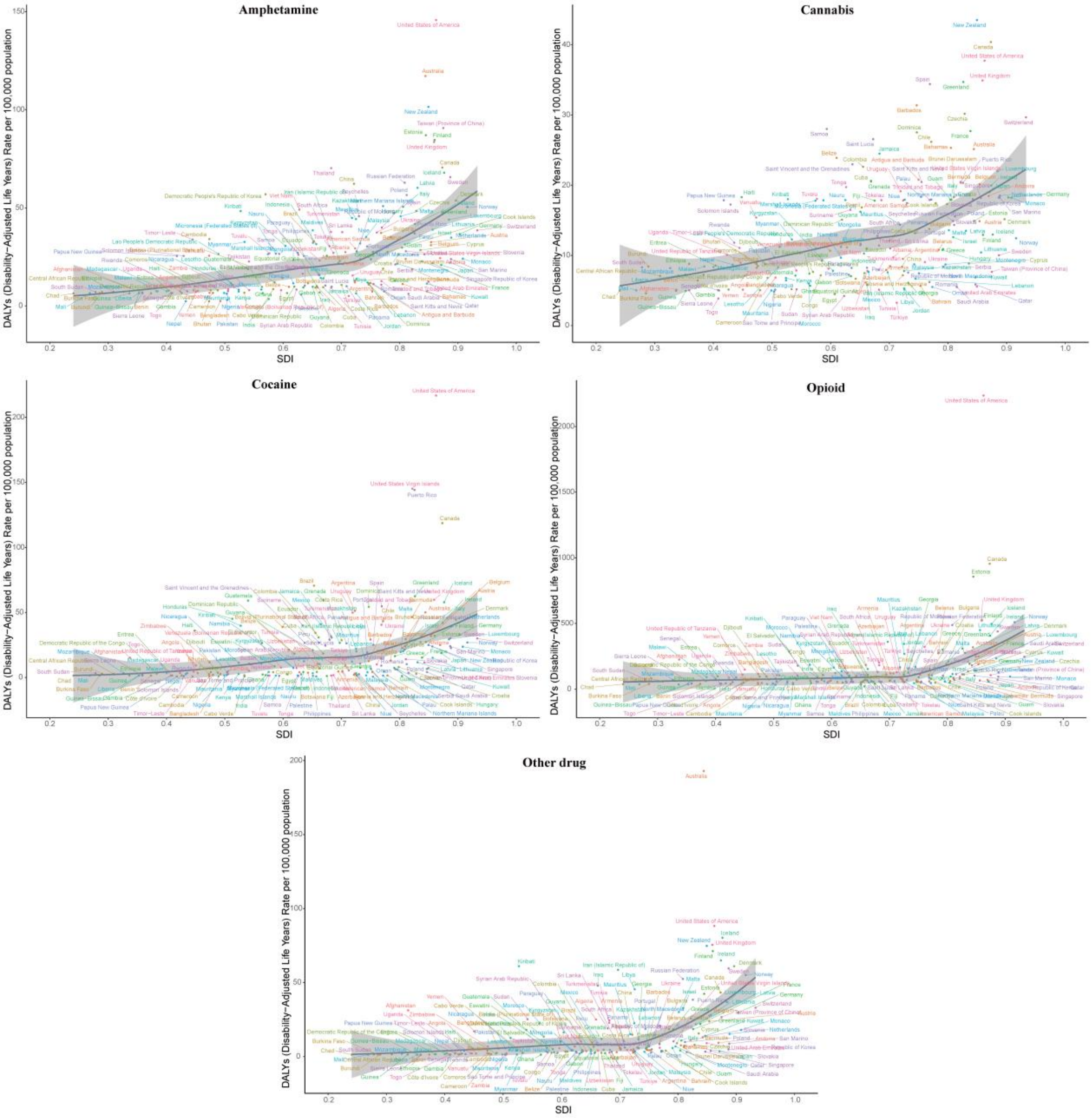

Figure S8. Global prevalence of 5 drug use disorders by age and sex in 2021

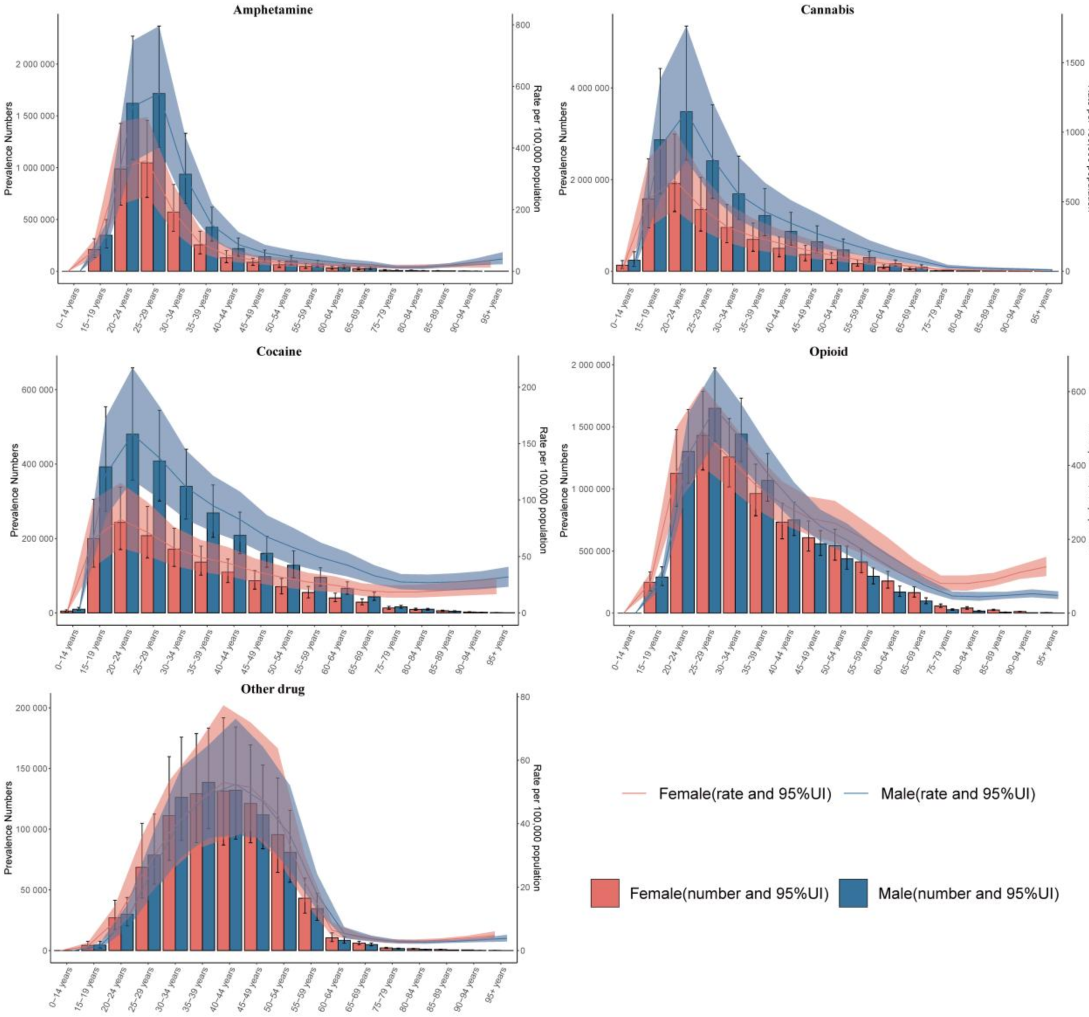

Figure S9. Global deaths of 5 drug use disorders by age and sex in 2021

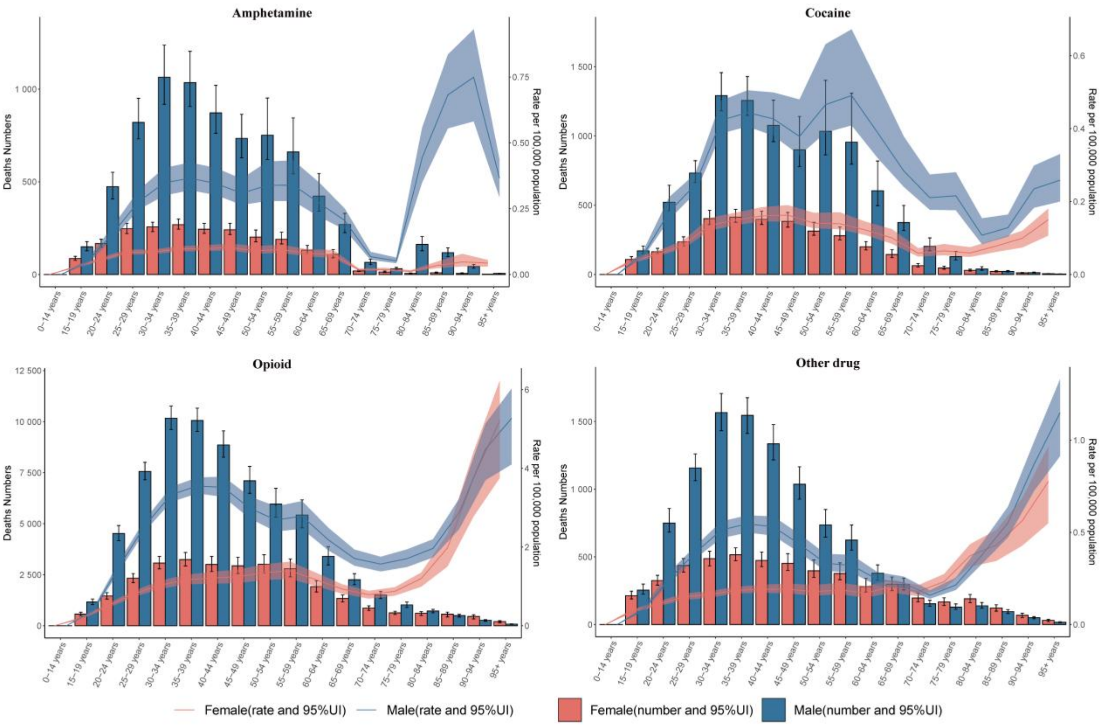

Figure S10. Global DALYs of 5 drug use disorders by age and sex in 2021

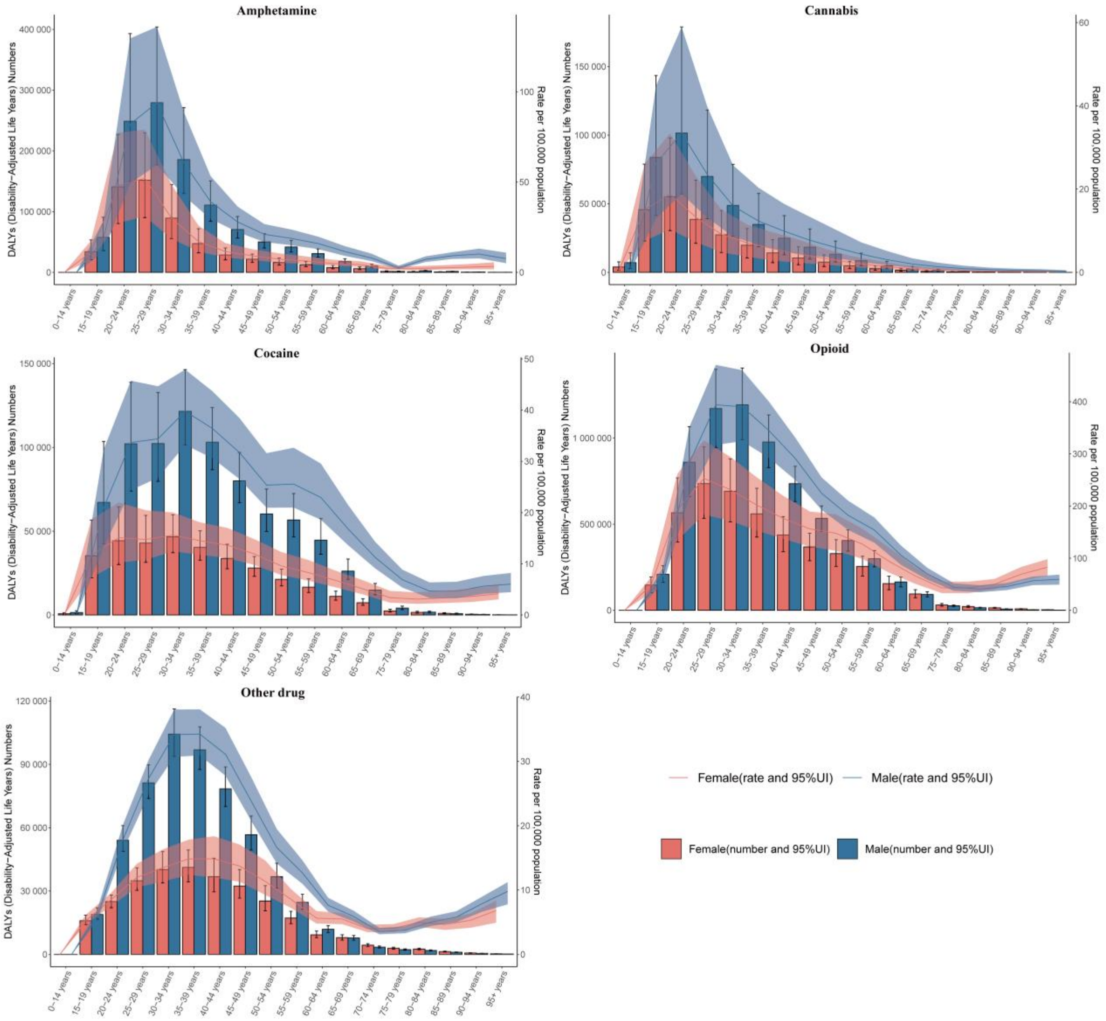

Figure S11. Change in DALYs of 5 drug use disorders decomposed by three population-level determinants: aging, population and epidemiological change at the global level and various regions

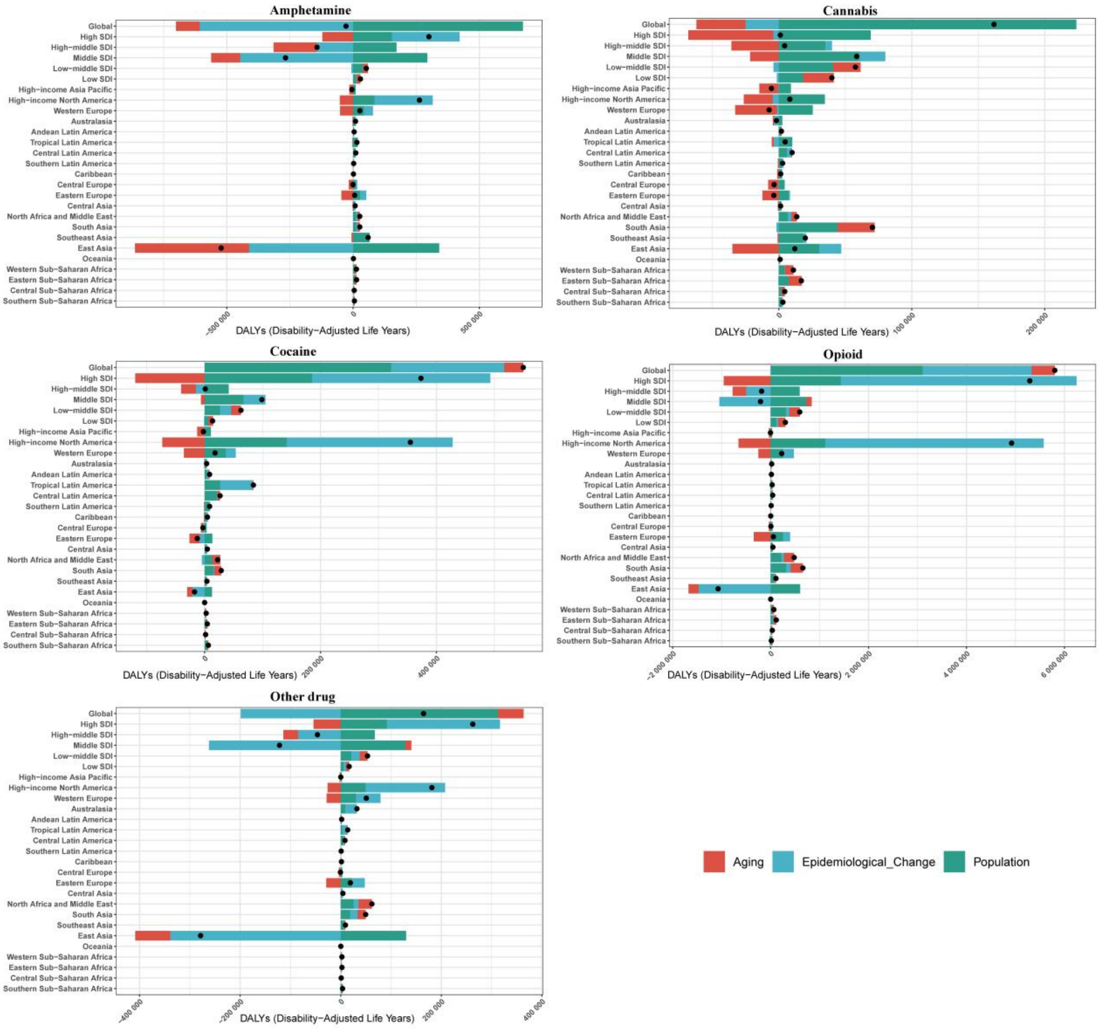

Figure S12. Change in deaths of 5 drug use disorders decomposed by three population-level determinants: aging, population and epidemiological change at the global level and various regions

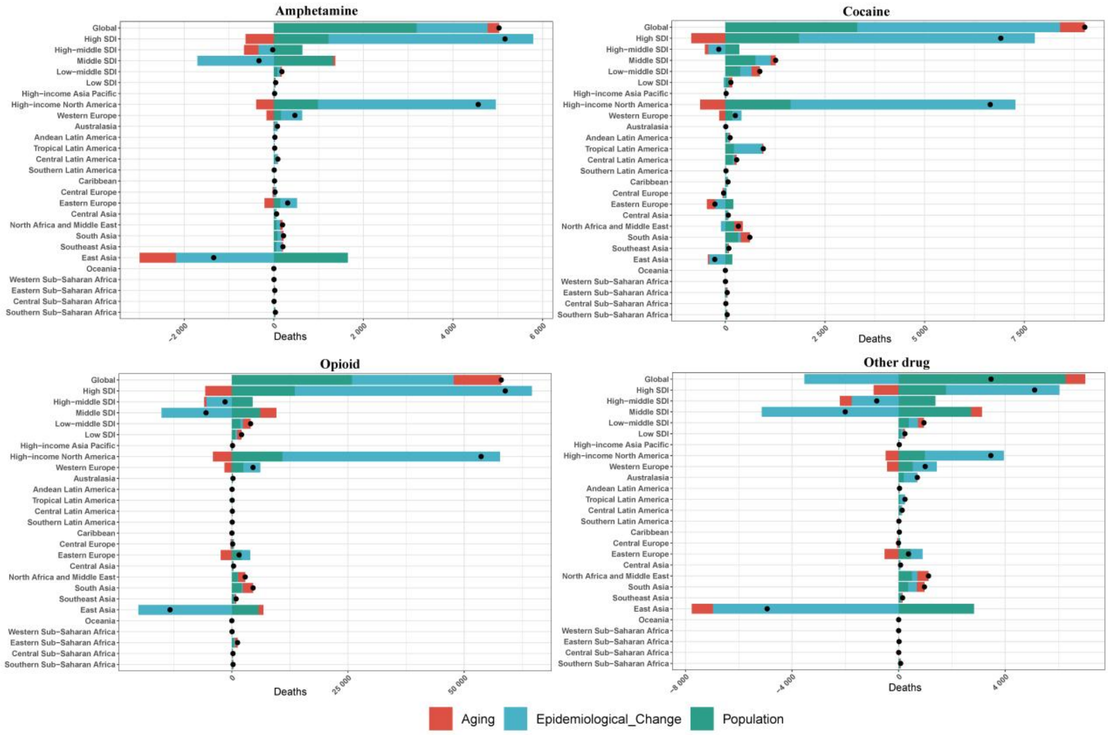

Supplement: Supplementary file 1 [file Data_Sheet_1.pdf]
